# Iron–digermanium cluster: synthesis, Lewis-base-induced isomerization, dihydrogen activation, and alkene cycloaddition

**DOI:** 10.1039/d6sc04230a

**Published:** 2026-08-17

**Authors:** Yang Liu, Jan C. Kruse, Andreas A. Lang, Gábor Balázs, Robert Wolf

**Affiliations:** a University of Regensburg, Institute of Inorganic Chemistry 93040 Regensburg Germany robert.wolf@ur.de; b University of Göttingen, Institute of Inorganic Chemistry 37077 Göttingen Germany

## Abstract

Heterometallic complexes containing multiple heavier tetrylidyne ligands (RE; E = Si, Ge, Sn, Pb) are promising for cooperative bond activation but are rare because RE units tend to oligomerize. We present the synthesis, electronic structure, and reactivity of an iron–digermanium complex, [Fe(GeAr′)_2_] (1, Ar′ = 2,6-Dipp_2_-C_6_H_3_, Dipp = 2,6-^*i*^Pr_2_-C_6_H_3_), featuring adjacent Ar′Ge units bonded to Fe through double and single Fe–Ge bonds, respectively. Complex 1 reacts readily with ^*t*^BuNC, H_2_, and styrene *via* coordination, bond activation, and cycloaddition pathways, producing diverse structural outcomes. Coordination of ^*t*^BuNC to Fe induces a formal reductive elimination of the two Ar′Ge units, forming an iron–digermyne complex (2) with a rare σ,σ-type double-bonding mode. Under mild conditions, H_2_ is activated, forming a germyl(iv)–iron–germylidyne complex (3). The reaction with styrene involves a [3 + 2]-cycloaddition, yielding a bis(germylene)–iron complex (4). Notably, the reaction with 2,3-dimethyl-1,3-butadiene (DMB) proceeds *via* an unusual kinetic [4 + 3]-cycloaddition pathway to produce a bis(germylene)–iron species (5), which then irreversibly rearranges into the thermodynamically favored [4 + 1] product (6). In contrast, the heavier iron–tin analogue exhibits distinct reactivity towards these substrates, highlighting fundamental differences between Fe–Ge and Fe–Sn bonding and reactivity.

## Abstract 2

A heterometallic FeGe_2_ cluster that combines germylidyne and metallogermylene sites exhibits flexible Fe–Ge bonding and diverse reactivity, including Lewis-base-triggered isomerization, H_2_ activation, and selective alkene cycloadditions.

## Graphical abstract

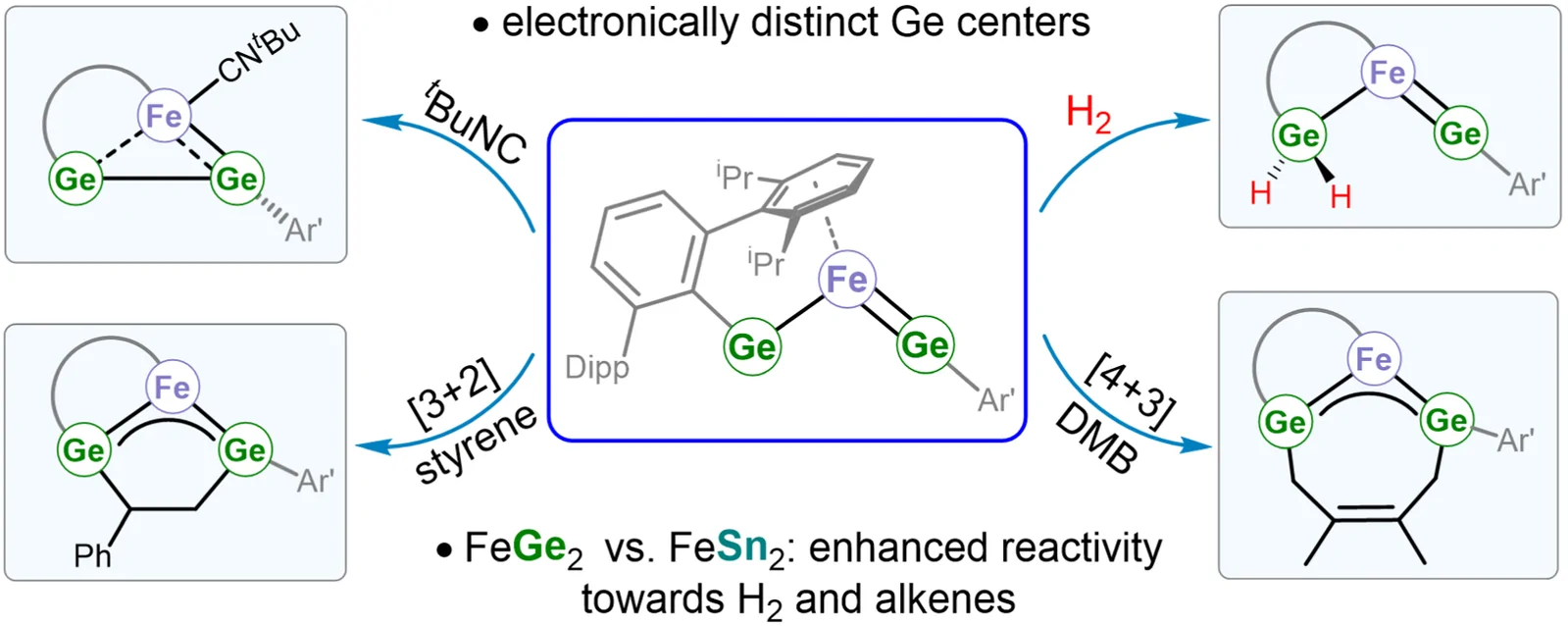

## Introduction

Recent years have seen growing interest in combining d-block metals (M) with heavier p-block elements (E) in cooperative chemistry.^1–5^ This approach offers unique opportunities for small-molecule activation and the discovery of new reactivity patterns. Low-valent p-block element ligands, which have small HOMO–LUMO gaps and ambiphilic properties, frequently participate in bond activation.^6,7^ In this field, heavier tetrylidynes (RE, E = Si, Ge, Sn, Pb) are appealing because of their unconventional M–E bonds in low-coordinate RE units, which can be harnessed for cooperative small-molecule activation.^8–10^

**Fig. 1:**
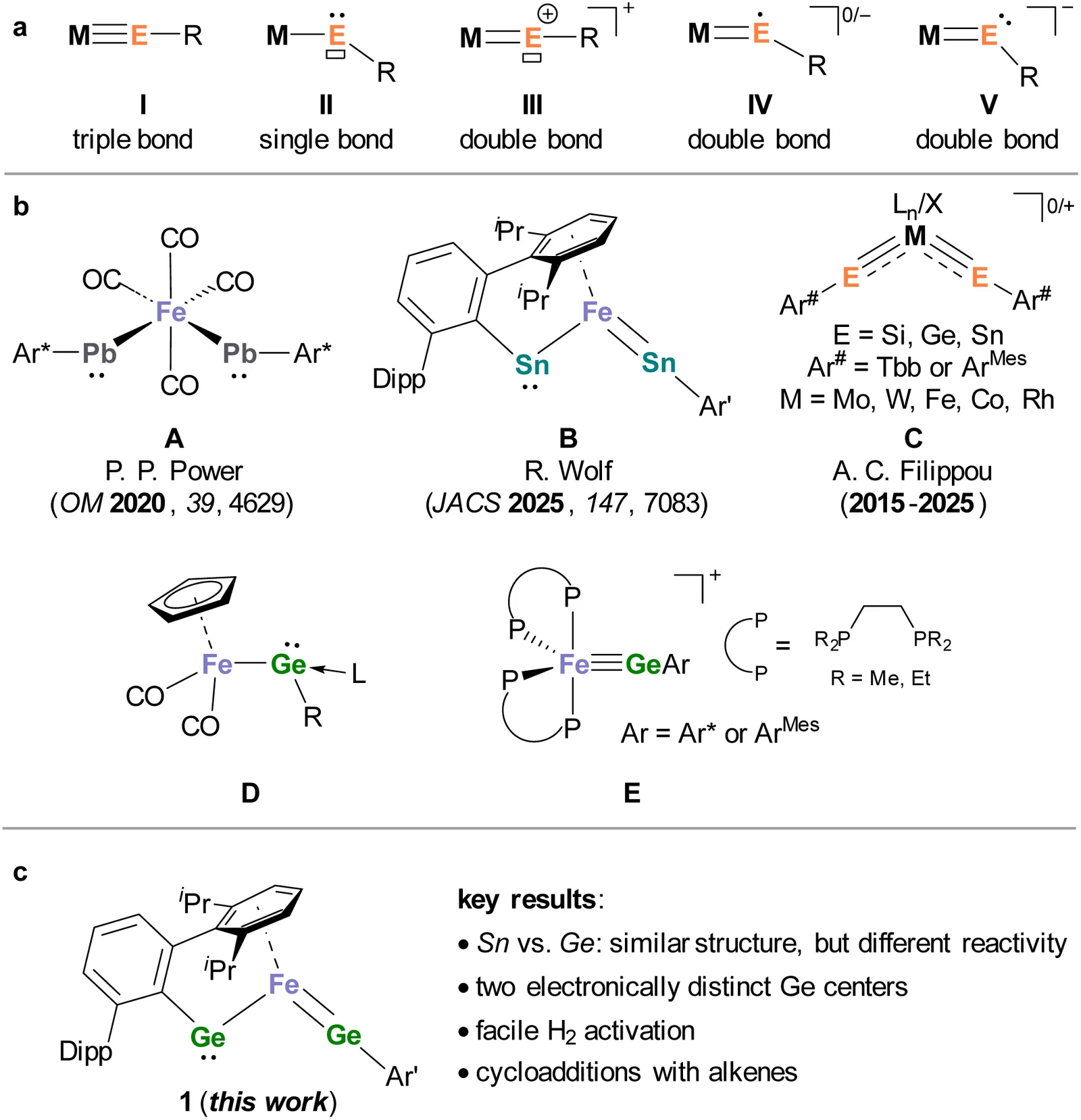
(a) Bonding modes of the tetrylidyne ligand. (b) Complexes featuring two transition–metal-bridged RE units and iron–germanium complexes (C: L = Me_3_P, *n* = 1–3, X = Cp or halide; D: L = Lewis base ligand, R = anionic substituent). (c) FeGe_2_ cluster 1 featuring versatile reactivity (this work).

Compared to carbyne ligands (RC), heavier tetrylidynes (E = Si–Pb) are generally less efficient π-acceptors, resulting in weaker M–E π-interactions.^11–14^ As a result, two M-ER bonding modes are feasible: triply bonded tetrylidynes (M≡ER, Fig. 1a, I) and singly bonded metallotetrylenes (M–ER; Fig. 1a, II).^8,15,16^ Additionally, a few metal complexes featuring M=E double bonds have been reported by the Wesemann, Tilley, Hadlington and Filippou groups, including cationic complexes with the motif [M=ER]^+^ (M = Ti, Ir, Fe, Co; E = Ge, Sn; Fig. 1a, III),^17–22^ open-shell (*S* = 1/2) complexes [M=EAr^Mes^]^0/−^ (M = Mo, Mn; E = Ge, Sn; Ar^Mes^ = 2,6-Mes_2_-C_6_H_3_, Mes = 2,4,6-Me_3_-C_6_H_2_) featuring tetrel-centered unpaired electron density (Fig. 1a, IV),^23^ and an anionic ruthenagermylene complex [(Ph_3_P)Ru=GeAr*]^−^ (Ar* = 2,6-Tripp_2_-C_6_H_3_, Tripp = 2,4,6-^*i*^Pr_3_-C_6_H_2_; Fig. 1a, V).^24^

The synthesis of complexes with neighboring RE ligands remains highly challenging.^25–27^ As a result, well-defined [M(ER)_2_] complexes are very rare. Power and co-workers prepared the metalla–diplumbylene complex [(CO)_4_Fe(PbAr*)_2_] (A) featuring two ferrioplumbylene units bonding to iron through Fe–Pb single bonds by reaction of the diplumbyne Ar*PbPbAr* with Fe_2_(CO)_9_ (Fig. 1b).^28^ We recently reported the iron–tin cluster [Fe(SnAr′)_2_] (B, Fig. 1b; Ar′ = 2,6-Dipp_2_-C_6_H_3_, Dipp = 2,6-^*i*^Pr_2_-C_6_H_3_), which contains an Fe–Sn single bond and a Fe=Sn double bond.^29^ Notably, Filippou and co-workers have synthesized several metal-bis(tetrylidyne) complexes [(L_*n*_(X)M(EAr^#^)_2_)]^0/+^ (C, Fig. 1b, M = Mo, W, Fe, Co, Rh; E = Si, Ge or Sn; Ar^#^ = Tbb or Ar^Mes^; Tbb = 2,6-[(SiMe_3_)_2_CH]_2_-4-^*t*^Bu-C_6_H_2_), in which both M–E bonds show multiple bond character.^30–33^ Among these ME_2_ clusters, complex B and the molybdenum–bis(germylidyne) complex [(Me_3_P)_3_Mo(GeAr^Mes^)_2_]^31^ (type C, Fig. 1b) have shown diverse reactivity towards small molecules, including P_4_, CS_2_, and CO_2_, highlighting the potential of complexes of this type as versatile reagents for cooperative small-molecule activation involving both the transition metal and a p-block element.

Although germylidyne and metallogermylene transition–metal complexes are generally well established (especially with group 6, 9 and 10 metals), complexes with RGe ligands and group 8 metals are scarce.^8,13,34^ The few known iron complexes typically exhibit a ferriogermylene structure containing an Fe–Ge single bond (D, Fig. 1b).^35–40^ In contrast, the complexes [L_2_FeGeAr]^+^ (E, Fig. 1b, Ar = Tbb, Ar^Mes^; L = diphosphine ligand) synthesized by the Filippou group feature linear Fe–Ge–Ar linkages and short iron–germanium distances, indicating Fe≡Ge triple bonds.^41^ Recently, Hadlington and co-workers reported an iron–germanium complex [(η^6^-arene)FeGeR(NHC)]^+^ (NHC = N-heterocyclic carbene), displaying an Fe=Ge double bond, in which the NHC-stabilized GeR unit was described as a cationic germylene.^21^ To date, the complexes [(Me_3_P)_2_Fe(GeTbb)_2_]^0/+^ appear to be the only iron complexes containing two RGe units (type C, Fig. 1b).^32^ Structural and computational data indicate that these complexes adopt delocalized Fe–Ge multiple bonds.

Here, we report the synthesis of the trinuclear iron–germanium complex [Fe(GeAr′)_2_] (1) showing ferriogermylene and germylidyne moieties in the same molecule. We examine the electronic structure and reactivity of 1 towards Lewis base (^*t*^BuNC), H_2_, styrene, and 2,3-dimethyl-1,3-butadiene (DMB), leading to structurally diverse products and demonstrating the markedly distinct reactivity of 1 in comparison to the iron–tin complex B. Changes in Fe–E bonding upon transitioning from Sn to Ge unlock H_2_ activation and alkene/diene cycloaddition chemistry. These studies reveal that the FeGe_2_ unit in 1 behaves as an iron-activated digermyne with tunable germylidyne/germylene character.

## Results and discussion

### Synthesis and electronic structure of complex 1

**Scheme 1:**
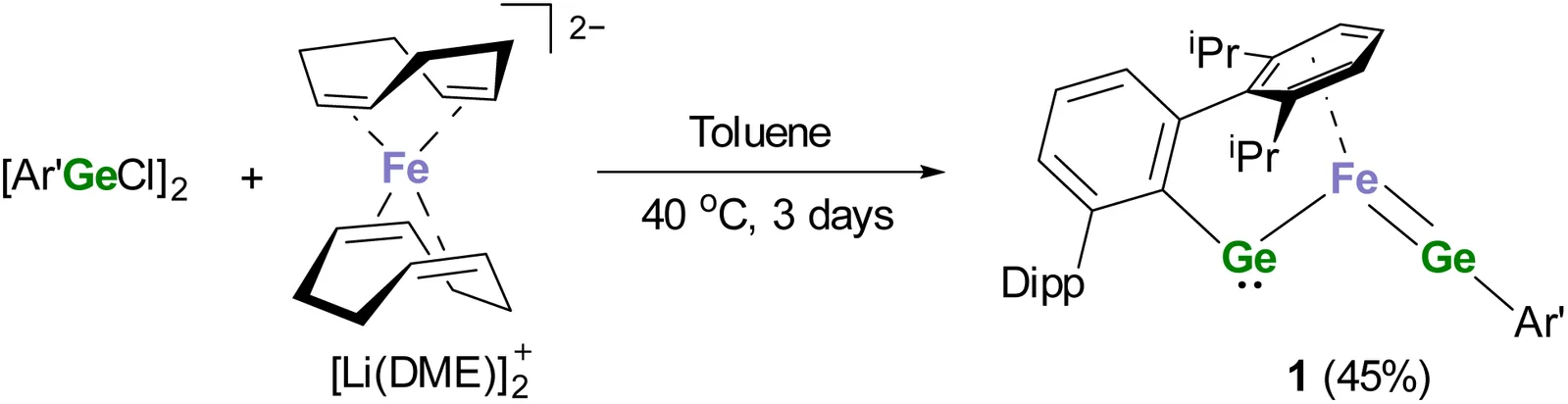
Synthesis of complex 1.

**Fig. 2:**
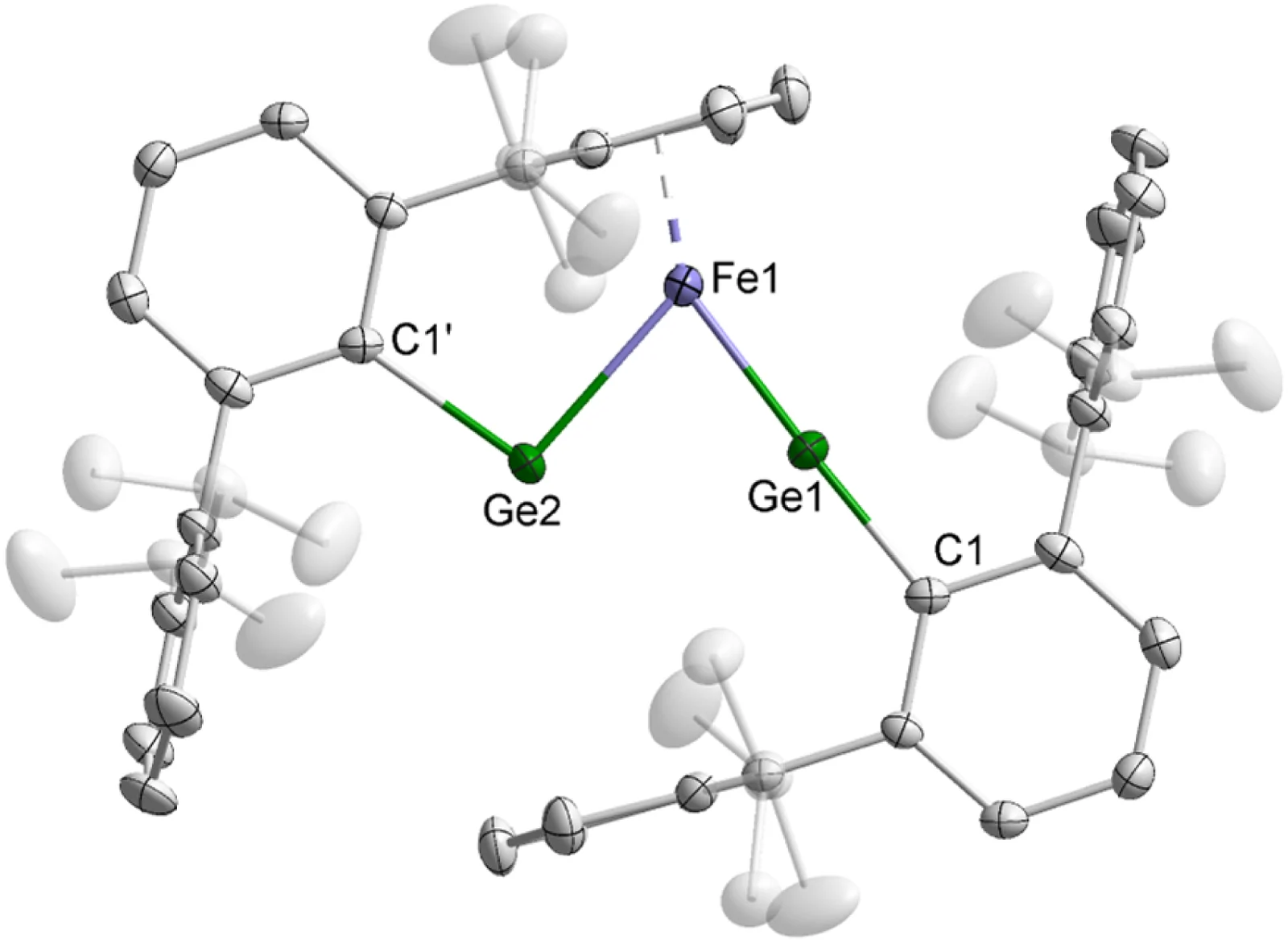
Molecular structure of 1 with 50% probability thermal ellipsoids. Hydrogen atoms are omitted for clarity. Selected bond lengths (Å) and angles (°): Fe1–Ge1 2.1061(9), Fe1–Ge2 2.4194(9), Ge1–C1 1.928(2), Ge2–C1′ 2.084(2), Ge1⋯Ge2 2.8738(9), Ge1–Fe1–Ge2 78.51(3), Fe1–Ge1–C1 176.86(7), Fe1–Ge2–C1′ 95.77(6).

Complex 1 was accessed through the reaction of the Fe^2−^ synthon [Li(DME)]_2_[Fe(COD)_2_] (DME = 1,2-dimethoxyethane, COD = 1,5-cyclooctadiene) with the terphenyl germanium(ii) dimer [Ar′GeCl]_2_ (Scheme 1). To suppress the formation of the digermyne [Ar′GeGeAr′] as a side product, the concentration and temperature must be strictly controlled. Complex 1 was isolated as dark brown crystals in 45% yield and characterized by multinuclear NMR spectroscopy and single-crystal X-ray diffraction (scXRD) analysis. Its solid-state molecular structure reveals an FeGe_2_ core in which the iron atom is coordinated by two Ar′Ge moieties and a flanking Dipp-aryl ring of one Ar′ substituent in η^6^-fashion (Fig. 2). Three sets of signals for the Dipp flanking groups were observed in the ^1^H NMR spectrum of 1 (C_6_D_6_, 298 K), consistent with its asymmetric coordination pattern (Fig. S1). Moreover, variable-temperature (VT) NMR experiments did not reveal any dynamic behavior of 1 in solution (*d*_8_-toluene). Complex 1 is thermally stable in an inert atmosphere, with no significant changes observed in the ^1^H NMR spectrum even after heating to 80 °C for 24 hours. The zero-field ^57^Fe Mössbauer spectrum of 1 (80 K, solid, Fig. S31) displays a doublet with an isomer shift value of *δ* = 0.51 mm s^−1^ and quadrupole splitting value of |Δ*E*_Q_| = 0.69 mm s^−1^. The lower isomer shift of 1 compared to that of B (0.61 mm s^−1^) indicates a relatively less electron-rich iron center. Given the closely similar geometries of 1 and B (*vide infra*), this difference points to a change in the Fe–E interactions (E = Ge *vs.* Sn).

The molecular structure of 1 features the shortest Fe–Ge bond reported to date (Fe1–Ge1 2.1061(9) Å), which is significantly shorter than those observed in iron–germylene complexes bearing Fe=Ge double bonds (2.19–2.37 Å),^21,42–45^ and comparable to those of iron–germylidyne complexes E (2.1198(4)–2.1342(4) Å) and [(Me_3_P)_2_Fe(GeTbb)_2_] (2.1137(7)/2.1153(8) Å) with Fe≡Ge triple bonds.^32,41^ The Ge1 center adopts an essentially linear geometry (∠Fe1–Ge1–C1 176.86(7)°). In contrast, the Fe1–Ge2 bond length (2.4194(9) Å) is much longer than the Fe1–Ge1 bond length, and falls within the typical range reported for ferriogermylene species (2.37–2.50 Å).^36–40^ The Fe1–Ge2–C1′ angle of 95.77(6)° is comparatively acute due to the geometric constraints. The Ge1⋯Ge2 distance of 2.8738(9) Å is substantially longer than a typical Ge–Ge single bond (the sum of the covalent radii of two Ge atoms is 2.42 Å),^46^ but shorter than the sum of the van der Waals radii (4.58 Å).^47^ In comparison, the Ge–Ge bond lengths in digermynes, such as [Ar′GeGeAr′] (2.2850(8) Å)^48^ featuring a Ge–Ge multiple bond, and [(Ar*(SiMe_3_)N)Ge]_2_ (2.7093(7) Å; Ar* = 2,6-Tripp_2_-C_6_H_3_, Tripp = 2,4,6-^*i*^Pr_3_-C_6_H_2_)^49^ and [(Ar^NEt2^)Ge]_2_ (2.6066(5) Å; Ar^NEt2^ = 2,6-(Et_2_NCH_2_)_2_–C_6_H_3_)^50^ containing Ge–Ge single bonds are shorter. In contrast to η^2^-coordinating ditetrylyne metal complexes, [M(η^2^-REER)] (M = Ni, Pd, Pt, Ag; E = Si, Ge),^51–54^ which typically display a near-perpendicular arrangement of the ME_2_ plane and the C–E–E–C array, the FeGe_2_ plane in 1 is coplanar with the C1′–Ge1⋯Ge2–C1 array. This configuration has also been observed for B.^29^ Such a configuration, combined with the long Ge1⋯Ge2 distance, indicates the absence of a Ge–Ge bond in 1.

**Fig. 3:**
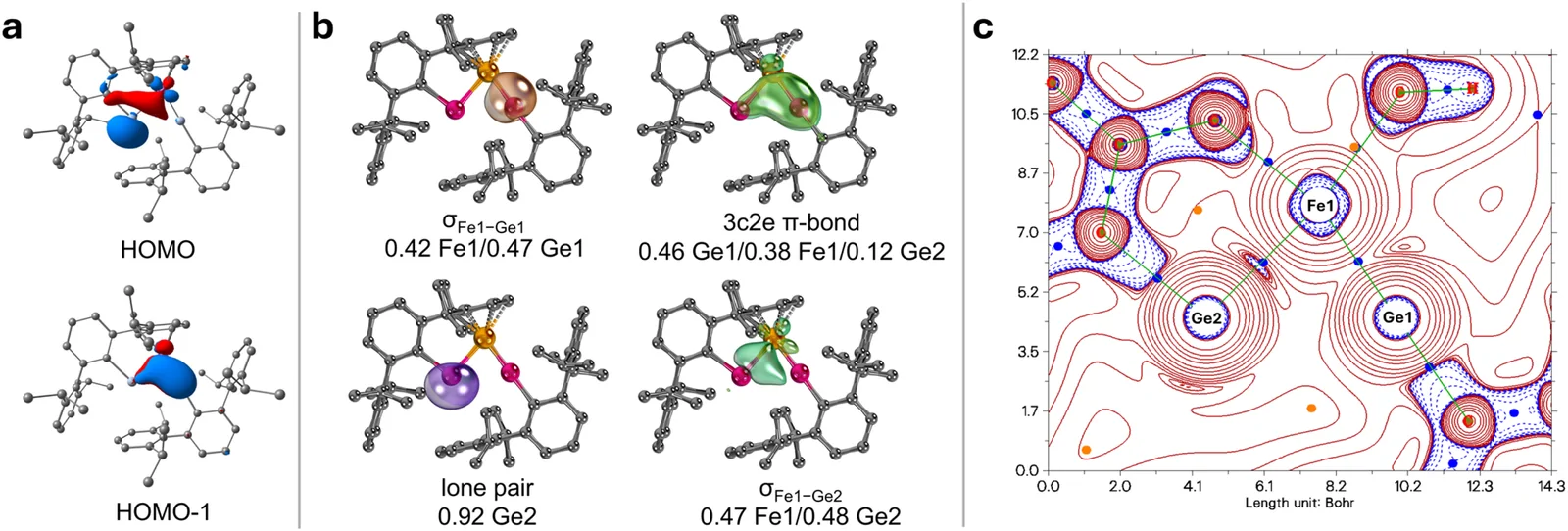
(a) HOMO and HOMO−1 of 1 (isovalue = 0.04). (b) Selected IBOs of 1. (c) Plot of the Laplacian of the electron density on the FeGe_2_ plane of 1 with bond critical points (blue dots), ring critical points (brown dots) and bond paths (green lines).

The electronic structure of 1 was analyzed using quantum chemical calculations with density functional theory (DFT) at the BP86-D3BJ/def2-TZVP//ωB97X-D3/def2-TZVP level of theory (see the SI for details). The optimized structure is in excellent agreement with the scXRD data (Fig. S39 and Table S3). The highest occupied molecular orbital (HOMO, −6.18 eV) corresponds to the Fe1–Ge2 σ-bond in combination with a Ge2 lone pair lying in the FeGe_2_ plane (Fig. 3a). HOMO−1 (−6.73 eV) represents the Fe1–Ge1 out-of-plane π-bond, which slightly delocalizes towards Ge2, forming a weak Fe1–Ge2 π-bonding interaction (Fig. 3a). The Fe1–Ge1 σ-bond lies much lower in energy (HOMO−18; Fig. S41). The lowest unoccupied molecular orbital (LUMO, 0.08 eV) and LUMO+1 (0.54 eV) consist mainly of out-of-plane p orbitals of the two Ge atoms with only minor contributions from Fe. LUMO+2 (0.73 eV) is dominated by an in-plane Ge1-centered p orbital (Fig. S41). Intrinsic bond orbital (IBO)^55^ analysis (Fig. 3b) discloses that both the σ and π interactions of the Fe1–Ge1 bond are slightly polarized towards Ge1. The Fe1–Ge2 σ bond is rather covalent with nearly equal contributions at the Fe1 and Ge2 atoms. This contrasts with the corresponding Fe–Sn bonds in B, which are highly polarized.^29^ A lone pair centered predominantly on Ge2 further confirms its germylene character. The more covalent nature of the FeGe_2_ core in 1 compared to the FeSn_2_ core in B is also reflected in the natural charge distribution (Fe1: −0.45; Ge1: +0.85; Ge2: +0.52), which is less separated compared to that calculated for the FeSn_2_ core in B (Fe1: −0.71; Sn1: +1.05; Sn2: +0.97). Additionally, the less negatively charged Fe center in 1 agrees well with the lower isomer shift value obtained from the ^57^Fe Mössbauer measurement of 1 compared to B (*vide supra*). The Mayer bond orders (MBOs) for Fe1–Ge1 (1.65) and Fe1–Ge2 (1.15) are larger relative to those of the Fe–Sn bonds in B (1.58 and 0.92), consistent with their more covalent nature. The MBO for the Ge1⋯Ge2 interaction is merely 0.52, indicating a weak Ge1⋯Ge2 orbital overlap. Quantum theory of atoms in molecules (QTAIM)^56,57^ analysis of 1 revealed bond critical points (BCPs) along the Fe1–Ge1 and Fe1–Ge2 bonds, while no BCP was located between Ge1 and Ge2 atoms (Fig. 3c). Based on the orbital images and metrical data, complex 1 is best characterized as containing both germylidyne (Ar′Ge1) and ferriogermylene (Ar′Ge2) groups, which are doubly and singly bonded to the iron center, respectively. The interactions of Fe1–Ge1 and Fe1–Ge2 exhibit greater covalent character than the corresponding Fe–Sn bonds in compound B.

### Reactivity studies of 1

**Scheme 2:**
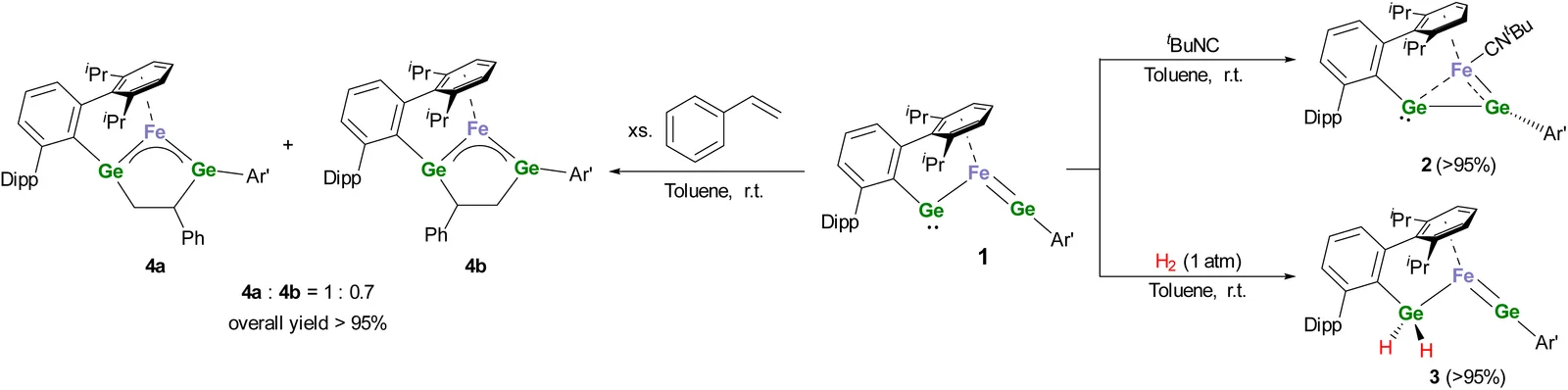
Reaction of 1 with ^*t*^BuNC, H_2_, and styrene to yield 2, 3, and 4a/b.

**Fig. 4:**
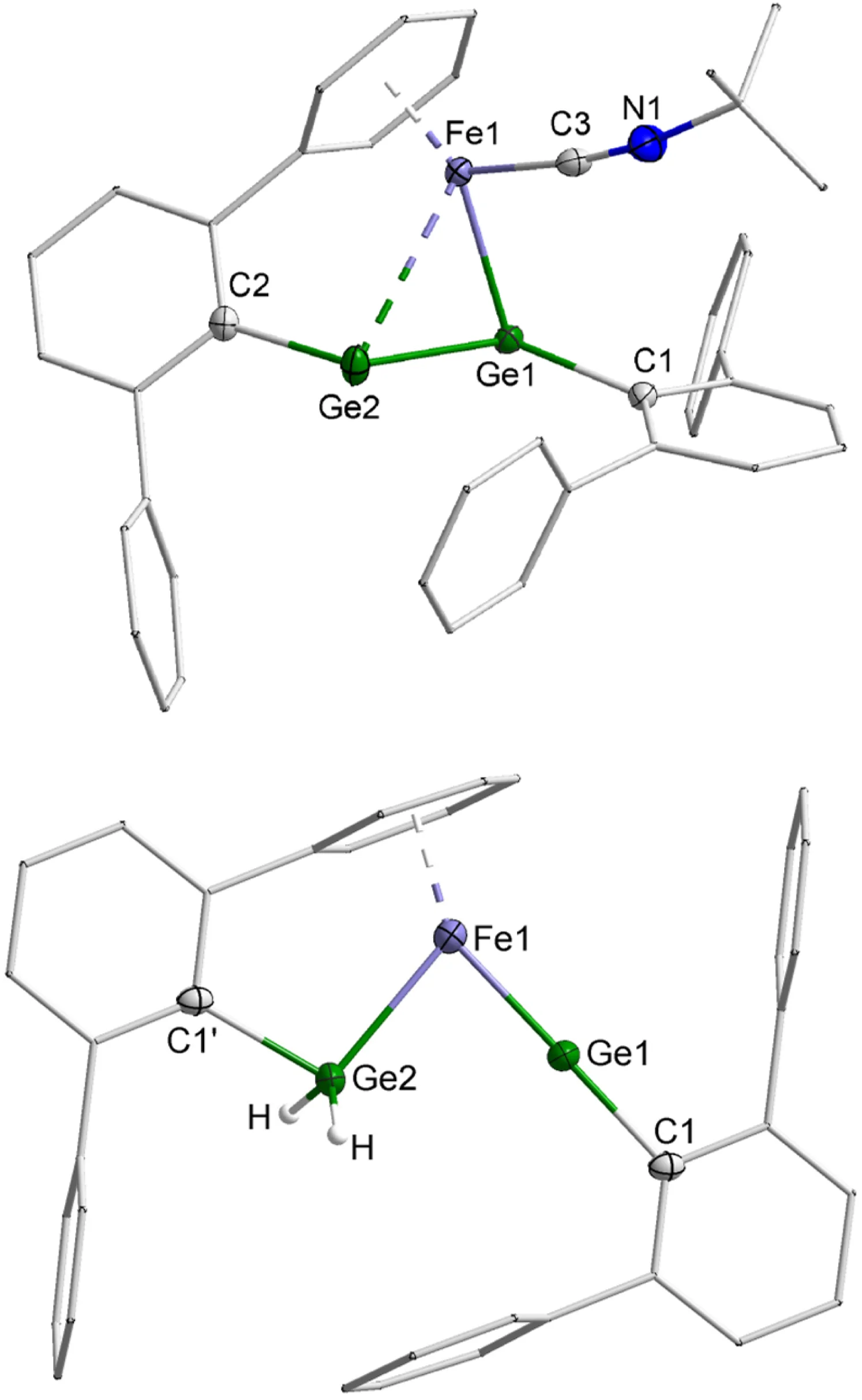
Molecular structure of 2 (top) and 3 (bottom) with 50% probability thermal ellipsoids. Hydrogen atoms attached to carbon atoms, solvent molecules, and the ^*i*^Pr substituents are omitted for clarity. Parts of the organic substituents are shown in wireframe. Selected bond lengths (Å) and angles (°) of 2: Fe1–Ge1 2.2916(5), Fe1–Ge2 2.9513(6), Fe1–C3 1.831(3), Ge1–C1 1.993(3), Ge2–C2 2.018(3), Ge1–Ge2 2.3787(5), Ge1–Fe1–Ge2 52.14(1), Fe1–Ge1–C1 121.39(8), Fe1–Ge2–C2 91.03(8), C1–Ge1–Ge2 119.09(8), C2–Ge2–Ge1 113.66(8). For 3: Fe1–Ge1 2.0994(6), Fe1–Ge2 2.3968(7), Ge1–C1 1.930(2), Ge2–C1′ 2.006(2), Ge1⋯Ge2 3.0161(6), Ge1–Fe1–Ge2 83.98(2), Fe1–Ge1–C1 176.03(6), Fe1–Ge2–C1′ 101.32(5).

Complex 1 reacts readily with ^*t*^BuNC, converting cleanly to complex 2, which was isolated as green crystals in near-quantitative yield (Scheme 2). An scXRD analysis of 2 reveals a formal iron–digermyne complex in which ^*t*^BuNC is bonded to the iron center (Fig. 4). The Ar′GeGeAr′ ligand coordinates to iron with both Ge atoms in an asymmetric fashion, with additional η^6^-coordination from a flanking Dipp-aryl ring. This contrasts with the reaction of B with PMe_3_, which induces cleavage of the Fe=Sn double bond and formation of a Sn–Sn single bond, yielding a distannyne ligand that coordinates to only one of its tin atoms.^29^ Unlike B, complex 1 does not react with PMe_3_. Additionally, the Power group has reported the reaction of the digermyne [Ar′GeGeAr′] with ^*t*^BuNC, in which ^*t*^BuNC binds to one Ge center.^58^

Compared to 1, the FeGe_2_ plane in 2 is no longer coplanar with the C–Ge⋯Ge–C array but is tilted by 66°. This angle is notably smaller than those observed in typical metal η^2^-ditetrylyne complexes (around 90°).^51–54^ The Ge1–Ge2 (2.3787(5) Å; MBO = 1.28) bond length is shorter than the sum of covalent radii of two Ge atoms (*ca.* 2.42 Å),^46^ but longer than the Ge–Ge bond length in the silver–digermyne complex [Ag(η^2^-Ar′GeGeAr′)] (2.303(2) Å)^53^ and the cobalt–digermyne complex [Cp(CO)Co(η^2^-TbbGeGeTbb)] (2.3456(3) Å),^32^ indicating the presence of a Ge–Ge bond with multiple bond character in 2. The Ge1 center adopts a pyramidal geometry with the sum of angles around Ge (∑_∠Ge1_) of 318.8°. The Fe1–Ge1 (2.2916(5) Å; MBO = 1.25) bond length in 2 is much shorter than the Fe–Ge single bonds in the formal iron–(η^2^-digermyne) complex [(NHC^*i*Pr_2_Me_2_^)GeFe(CO)_4_]_2_ (2.5970(3) and 2.6614(3) Å),^59^ and is within the range reported for iron–germylene complexes containing Fe=Ge double bonds (2.19–2.37 Å).^21,42–45^ The Fe1–Ge2 (2.9513(6) Å) bond is significantly longer than the Fe1–Ge1 bond, but shorter than the sum of the van der Waals radii of Fe and Ge atoms (∑_rvdW_ = 4.73 Å).^47^ This elongated distance, together with the MBO value of 0.53 and QTAIM analysis of the FeGe_2_ plane showing no BCP between Fe1 and Ge2 atoms (Fig. S43), suggests a notably weak Fe–Ge interaction. Consistently, IBO analysis supports the presence of an Fe1–Ge2 orbital interaction and a nonbonding lone pair localized on the Ge2 atom. The Fe1–Ge1 bond exhibits σ,σ-type double bonding (Fig. S44). This is consistent with its short bond length and the pyramidal geometry of the Ge1 center. Similar bonding modes have been observed in dihalotetrel-*d*^10^ metal complexes reported by the Braunschweig group.^60,61^ So far, four formal transition metal–digermyne complexes have been reported, and all adopt metallacyclic MGe_2_ motifs.^32,51,53,59^ The unprecedented Fe–Ge_2_ bonding mode observed in 2 can be attributed to the asymmetric coordination between Fe and the two Ge atoms due to the strong η^6^-arene-iron interaction of one of the flanking aryl ligands.

Dihydrogen (H_2_) is among the most challenging small molecules to activate due to its strong (BDE = 104 kcal mol^−1^) and nonpolar H–H bond. Nevertheless, a small number of metallogermylene complexes [M(GeR)] (M = W, Mo, Zn) have been shown to activate H_2_, and all were proposed to proceed *via* metal–germanium cooperativity based on quantum-chemical calculations.^62–64^ Given that both the HOMO and LUMO of 1 have contributions from the Fe 3d and Ge 4p orbitals, a potential cooperativity in the hydrogenation of 1 might be expected. Therefore, we investigated the reactivity of 1 towards H_2_. Exposure of a toluene solution of 1 to H_2_ (1 atm) resulted in a color change from dark brown to green over the course of 10 min, after which green crystals of complex 3 were isolated in near-quantitative yield (Scheme 2). This is in stark contrast to complex B, which shows no reactivity towards H_2_ even at an elevated temperature (80 °C) for 3 days.

The solid-state molecular structure of 3 reveals that only one equivalent of H_2_ is oxidatively added to the Ge atom of the ferriogermylene moiety in 1, forming a germyl(IV) ligand (Fig. 4). In comparison, Power's digermyne compound [Ar′GeGeAr′] can react with up to three equivalents of H_2_,^65^ while complex 3 appears to be inert towards H_2_. The ^1^H NMR spectrum of 3 (C_6_D_6_, 298 K) displays a singlet at 3.14 ppm (2H), which corresponds to the GeH_2_ unit (*cf.* 3.21 ppm for [Ar′(H_2_)Ge]_2_ and 3.78 ppm for [Cp*W(CO)_3_Ge(H_2_)NHC^Dipp^][BAr^F^_4_]).^64,65^ In the IR spectrum of 3, one Ge–H stretching band was detected at 2002 cm^−1^ (*cf.* 2100, 2060 cm^−1^ for [Ar′(H_2_)Ge]_2_ ^65^ and 2054, 1995 cm^−1^ for [(TBoN)Ge(H_2_)ZnL*]^62^). No obvious changes were observed in the ^1^H NMR (*d*_8_-toluene) spectra of 3 at low temperatures (213–253 K), providing no evidence for dynamic behavior on the NMR timescale, such as hydride migration in solution. The Fe1–Ge2 (2.3968(7) Å; MBO = 0.68) bond length in 3 lies in the expected range for an Fe–Ge(iv) single bond. The Fe1–Ge1 (2.0994(6) Å; MBO = 1.74) bond length and Fe1–Ge1–C1 bond angle (176.03(6)°) are almost identical to those in 1, suggesting a similar germylidyne motif with double bond character. This is also supported by the IBO analysis, which shows σ and π interactions between Fe1 and Ge1 (Fig. S46). The Ge1⋯Ge2 distance (3.0161(6) Å) is much longer than that in 1 (2.8738(9) Å), indicating no interaction between the two Ge atoms.

DFT calculations were performed to elucidate the reaction mechanism and support a cooperative dihydrogen activation pathway involving simultaneous interaction of H_2_ with Fe and Ge2 at the ferriogermylene site (see the SI for details). This pathway is energetically favored, with an activation barrier much lower (ΔΔ*G*^‡^ = 7.3 kcal mol^−1^) than an alternative oxidative addition process occurring solely at Ge2, which is commonly proposed for other germylenes.^6,39,66^

**Fig. 5:**
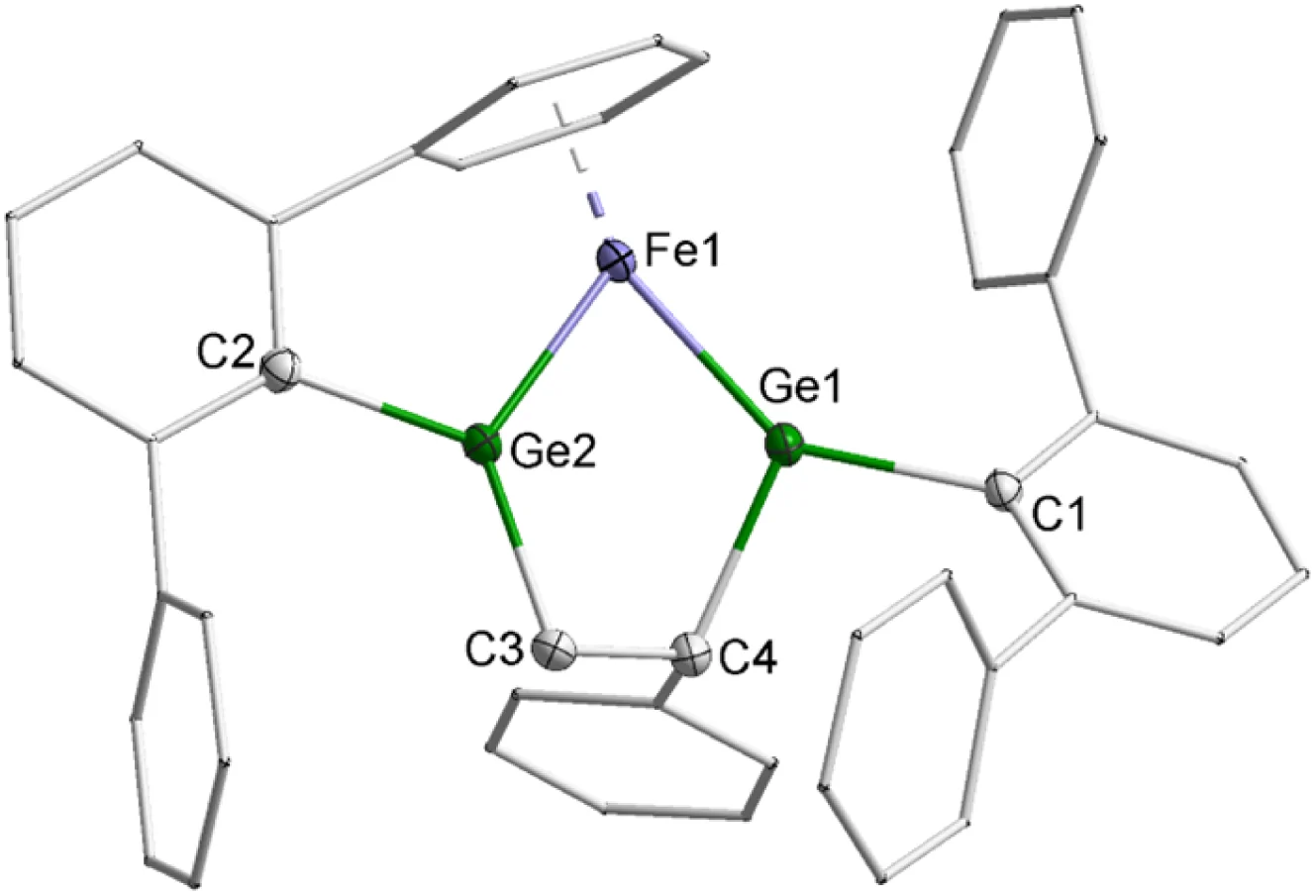
Molecular structure of 4a with 50% probability thermal ellipsoids. Hydrogen atoms, solvent molecules, and the ^*i*^Pr substituents are omitted for clarity. Parts of the organic substituents are shown in wireframe. Selected bond lengths (Å) and angles (°) of 4a: Fe1–Ge1 2.2441(4), Fe1–Ge2 2.1857(4), Ge1–C1 2.020(2), Ge1–C4 2.074(2), Ge2–C2 1.955(2), Ge2–C3 1.970(2), C3–C4 1.534(3), Ge1⋯Ge2 2.8442(3), Ge1–Fe1–Ge2 79.88(1), C1–Ge1–C4 102.54(8), C2–Ge2–C3 128.02(9).

Intrigued by the cycloaddition chemistry of digermynes with alkenes,^67–70^ we explored the reactions of 1 with styrene and 2,3-dimethyl-1,3-butadiene (DMB). Complex 1 reacts smoothly with styrene at ambient temperature, affording complex 4 as a mixture of two regioisomers (4a/4b = 1 : 0.7, determined by ^1^H NMR spectroscopy; Scheme 2 and Fig. S11), which were isolated as green crystals in near-quantitative overall yield. In contrast, complex B does not react with styrene even at 80 °C. Both 4a and 4b have been characterized by scXRD (Fig. 5 and S35) and exhibit similar FeGe_2_ core structures, differing only in the position of the phenyl substituent. The solid-state molecular structure of 4a reveals a bis(germylene)–iron complex with an FeGe_2_C_2_ five-membered ring, which is likely formed *via* a formal [3 + 2]-cycloaddition of the C=C double bond of styrene with the FeGe_2_ core of 1. The FeGe_2_C_2_ ring has an envelope shape, with the C4 atom slightly tilted from the ring plane. The two Ge atoms have nearly planar geometries (∑_∠Ge_ = 360°). The Fe1–Ge1 (2.2441(4) Å; MBO = 1.46) and Fe1–Ge2 (2.1857(4) Å; MBO = 1.51) bond lengths fall within the range reported for iron–germylene complexes featuring Fe=Ge double bonds (2.19–2.37 Å).^21,42–45^ Consistent with these structural data, IBO analysis shows two Ge → Fe σ-bonding interactions and a delocalized three-center, two-electron (3c2e) Ge–Fe–Ge π-bonding interaction (Fig. S49). These results confirm the bis(germylene)-iron structural motif of 4, with considerable Fe=Ge double-bond character. The C3–C4 (1.534(3) Å) bond length is indicative of a C–C single bond.

Bis(germylene) transition–metal complexes are rare and are typically synthesized from the reaction of free bis(germylene) ligands with metal sources.^71–73^ Notably, the Filippou group reported the synthesis of bis(germylene)cobalt complexes structurally related to 4, obtained by reacting the cobalt–digermyne complexes [Cp(CO)_n_Co(η^2^-TbbGeGeTbb)] (*n* = 0, 1) with alkynes.^32^ The formation of 4 represents a new approach to access bis(germylene)–metal complexes. We propose that the reaction of 1 with styrene proceeds *via* an initial [2 + 1]-cycloaddition between the C=C bond of styrene and the ferriogermylene Ge2, followed by a C-migration (or Ge insertion) to yield 4. Such a pathway has been theoretically studied for the formal [2 + 2]-cycloaddition of ditetrylynes with alkenes.^69,74,75^

**Fig. 6:**
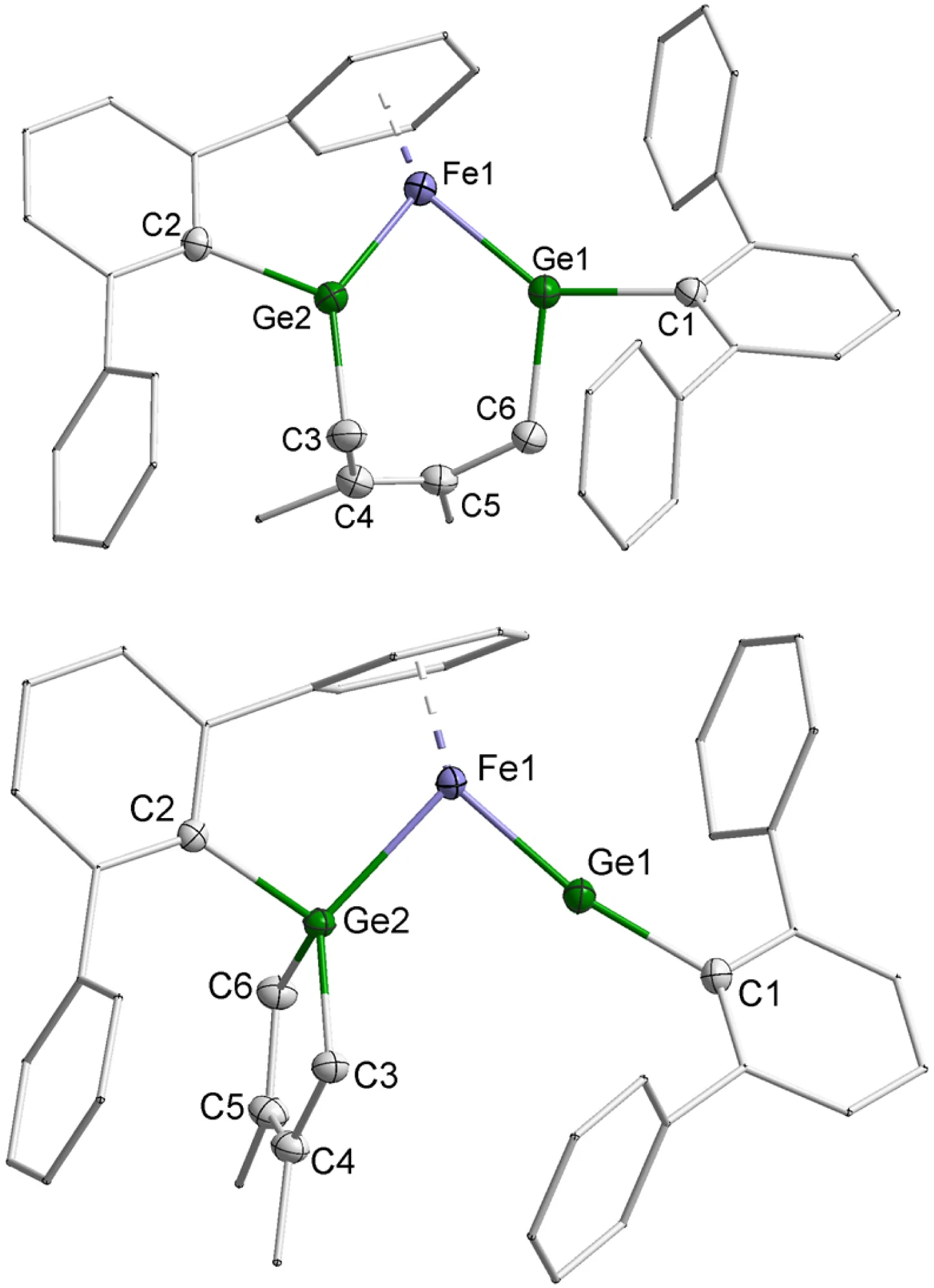
Molecular structure of 5 (top) and 6 (bottom) with 50% probability thermal ellipsoids. Hydrogen atoms, solvent molecules, and the ^*i*^Pr substituents are omitted for clarity. Parts of the organic substituents are shown in wireframe. Selected bond lengths (Å) and angles (°) of 5: Fe1–Ge1 2.2260(5), Fe1–Ge2 2.2150(5), Ge1–C1 2.030(3), Ge1–C6 2.012(3), Ge2–C2 1.990(3), Ge2–C3 1.987(3), C4–C5 1.344(4), Ge1⋯Ge2 3.0663(6), Ge1–Fe1–Ge2 87.33(2), C1–Ge1–C6 96.05(11), C2–Ge2–C3 116.60(12). For 6: Fe1–Ge1 2.1069(2), Fe1–Ge2 2.4021(2), Ge1–C1 1.956(1), Ge2–C2 2.010(1), Ge2–C3 1.982(1), Ge2–C6 1.993(1), C4–C5 1.337(2), Ge1⋯Ge2 3.2368(8), Fe1–Ge1–C1 168.39(4), Ge1–Fe1–Ge2 91.514(8), Fe1–Ge2–C2 101.03(3), C3–Ge2–C6 90.70(5).

**Scheme 3:**
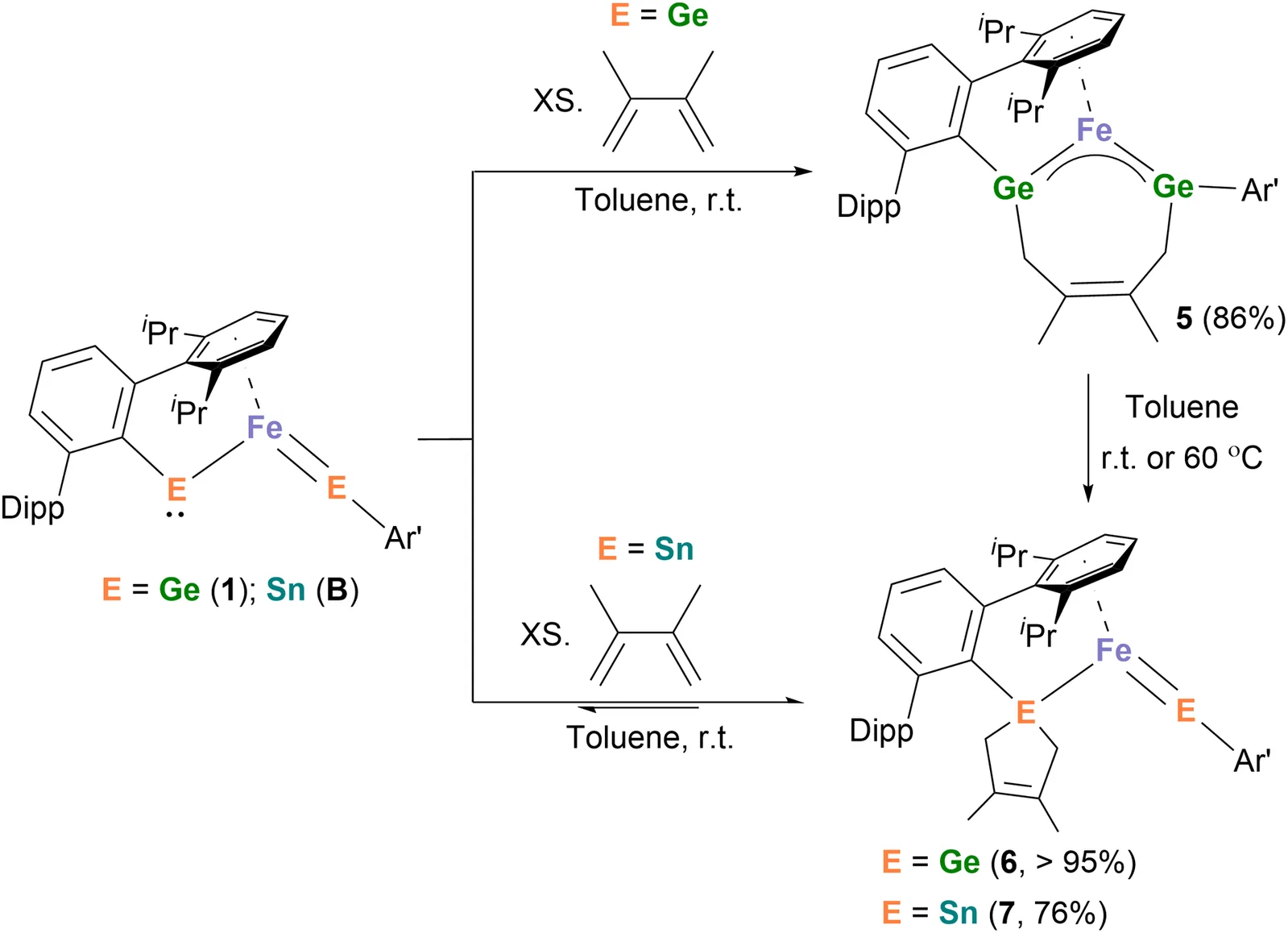
Reaction of 1 or B with DMB to yield 5, 6, or 7.

DMB is commonly used as a trapping reagent for germylenes, which generally undergo [4 + 1]-cycloaddition with DMB to form germacyclopentenes.^67,76,77^ Intriguingly, complex 1 reacts *via* a formal [4 + 3]-cycloaddition with DMB, resulting in the bis(germylene)–iron complex 5 with an FeGe_2_C_4_ seven-membered ring as revealed by its solid-state molecular structure (Fig. 6). 5 was isolated as brown crystals in 86% yield (Scheme 3). The geometries of the two Ge centers, which are planar, and Fe1–Ge1 (2.2260(5) Å; MBO = 1.41) and Fe1–Ge2 (2.2150(5) Å; MBO = 1.48) bond lengths of 5 are quite similar to those of 4a, indicating a bis(germylene)–iron structural motif. This was further supported by IBO analysis (Fig. S52). The C4–C5 (1.344(4) Å) bond length corresponds to a C=C double bond.

Subsequent studies showed that complex 5 converts very slowly to the ferriogermolene 6 at ambient temperature (24% conversion after 5 days), while complete conversion was achieved within 48 h at 60 °C (Scheme 3). This transformation is irreversible, and 6 was isolated as green crystals in near-quantitative yield. These observations indicate that 5 is a kinetic product in the reaction of 1 with DMB, whereas 6 represents the thermodynamically favored species. DFT calculations support these experimental results. 5 and 6 are nearly isoenergetic, with 6 stabilized by 0.61 kcal mol^−1^.

The scXRD structure of 6 shows that DMB is [4 + 1] cycloadded to the Ge atom of the ferriogermylene unit, resulting in a distorted tetrahedral Ge(iv) center (Fig. 6). The Fe1–Ge2 (2.4021(2) Å; MBO = 0.80) and Fe1–Ge1 (2.1069(2) Å; MBO = 1.90) bond lengths in 6 are comparable to the corresponding Fe–Ge bond lengths in 1 and 3, indicating their single and double bond character, respectively. This assignment is further corroborated by IBO analysis (Fig. S55). Notably, the MBO of the Fe1–Ge1 (1.90) bond in 6 is significantly larger than that of the corresponding Fe1–Ge1 (1.65) bond in 1, indicating enhanced double bond character. This difference can be attributed to the absence of π-electron delocalization towards the Ge2 atom in 6. The C4–C5 (1.337(2) Å) bond can be assigned as a C=C double bond.

To compare this reactivity of 1 with that of its heavier analogue B, the reaction of B with excess DMB was investigated. Interestingly, the [4 + 1]-cycloaddition product 7 was formed instead of a [4 + 3]-cycloaddition product (Scheme 3). Monitoring the reaction by ^1^H NMR spectroscopy (298 K, C_6_D_6_) shows that 7 is the only new species detected. Notably, this reaction is reversible. Upon dissolving single crystals of 7 in C_6_D_6_ (298 K), ^1^H NMR signals corresponding to the starting material B and DMB gradually appear, and an equilibrium is established with a 7 : B ratio of 2 : 1. Although the observed reversibility suggests a low-energy barrier for the conversion of B to 7, the involvement of a transient [4 + 3]-cycloaddition intermediate, analogous to 5, cannot be ruled out. Complex 7 was isolated as dark brown crystals in 76% yield and was characterized by scXRD. Its solid-state molecular structure closely resembles that of 6 (Fig. S38). The bond lengths and angles, together with DFT calculations (Fig. S58), indicate a stannyl(iv)–iron–stannylidyne complex with Fe–Sn single and double bonds.

Power and Tokitoh have reported reactions of digermynes [Ar*GeGeAr*]^68^ and [BbtGeGeBbt] (Bbt = 2,6-[(Me_3_Si)_2_CH]_2_-4-[(Me_3_Si)_3_C]-C_6_H_2_)^67^ with DMB, respectively, in which the DMB undergoes [4 + 1]-cycloaddition to the Ge centers. Direct [4 + 2]-cycloaddition, followed by a ring contraction, has been proposed as one possible pathway for these reactions. Based on the observations described herein, the conversion of 1 to 5 may proceed *via* a direct [4 + 3]-cycloaddition of DMB and the Ge–Fe–Ge unit. The LUMO of 1 is primarily composed of the out-of-plane 4p orbitals with π* symmetry, which can effectively interact with the HOMO of DMB (Fig. S61). Alternatively, an initial [4 + 2]-cycloaddition between DMB and the Fe=Ge unit, followed by a C-migration from Fe to Ge, could also account for the formation of the kinetic product 5.

## Conclusions

In conclusion, we have synthesized the first iron–germanium complex [Fe(GeAr′)_2_] (1) featuring adjacent, electronically differentiated Ar′Ge units with germylidyne and germylene character, respectively. Detailed structural and computational analyses reveal that 1 contains covalent Fe–Ge single (σ) and Fe=Ge double (σ + π) bonds. Complex 1 was shown to react with the Lewis base ^*t*^BuNC, H_2_, and alkenes (styrene and DMB) *via* coordination, H–H bond activation, and cycloadditions, respectively, to form iron–digermyne (2), germyl–iron–germylidyne (3), and iron–bis(germylene) (4 and 5) complexes. The iron–bis(germylene) complex 5 slowly transforms into a thermodynamically favored germyl–iron–germylidyne product (6) *via* a C-migration process. The reactivity of 1 is markedly distinct from its heavier iron–tin counterpart (B) and the digermyne compound [Ar′GeGeAr′].

In these reactions, the Fe center is involved in variable bonding interactions with both Ge atoms, ranging from Fe–Ge single bonds to Fe=Ge double bonds. This bonding flexibility may modulate the electronic properties of the Ge centers and contribute to the diverse reactivity observed for complex 1. More broadly, complex 1 can be regarded as an iron-activated digermynein which the Ge≡Ge triple bond has been broken, and the resulting two Ar′Ge groups are bridged by an Fe atom that adjusts their electronic properties. This behavior is demonstrated by the reactions of 1 with H_2_ and DMB, which resemble those of free digermynes but proceed *via* distinct and selective pathways. These results highlight the potential of heterometallic systems that combine transition metals with multiple low-coordinate, low-valent tetryl centers to achieve unusual reactivity. Ongoing studies in our laboratory explore broader applications of this system for cooperative bond activation and aim to diversify the heterotrimetallic framework with other transition metals.

## Author contributions

Conceptualization: Y. L. and R. W.; all synthetic experiments and writing – original draft: Y. L.; computational work: Y. L. and G. B.; X-ray crystallography: Y. L. and A. A. L.; Mössbauer spectroscopy: J. C. K.; review and editing: all authors; supervision, project administration and funding acquisition: R. W.

## Conflicts of interest

There are no conflicts to declare.

## Supplementary Material

SC-OLF-D6SC04230A-s001

SC-OLF-D6SC04230A-s002

## Data Availability

The data supporting this article are included as part of the supplementary information (SI). Supplementary information: experimental procedures, characterization data of new complexes and computational details. See DOI: https://doi.org/10.1039/d6sc04230a. CCDC 2553713–2553720 contain the supplementary crystallographic data for this paper.^78*a*–*h*^
