# Supplementary material for: Iron–digermanium cluster: synthesis, Lewis-base-induced isomerization, dihydrogen activation, and alkene cycloaddition
Source: Chem Sci. 2026 Aug 17;17(39):18961–9. doi: 10.1039/d6sc04230a (PMC13504319; doi:10.1039/d6sc04230a)
Supplement: Supplementary file 1 [file SC-017-D6SC04230A-s001.pdf]

Supplementary Information (SI) for Chemical Science.
This journal is © The Royal Society of Chemistry 2026

Electronic Supplementary Information

Table of Contents

Experimental Procedures 2
General Considerations 2
Synthesis of Complexes **1**-**7** 2
NMR Spectra 8
UV-vis Spectra 18
IR Spectra 22
Zero-field ^{57}Fe Mössbauer Measurements 23
Crystallographic Data 23
DFT Calculations 30
References 57

Experimental Procedures

General Considerations

All manipulations of air- and moisture-sensitive materials were performed under an atmosphere of dry dinitrogen with rigorous exclusion of air and moisture using standard Schlenk techniques, or in an argon-filled glovebox. Solvents were purified, dried, and degassed with an MBraun SPS800 solvent purification system. All dry solvents except *n*-hexane and *n*-pentane were stored under argon over activated 3 Å molecular sieves in gas-tight Schlenk tubes. *n*-Hexane and *n*-pentane were stored over a potassium mirror. d_6-Benzene (C_6D_6) and d_8-tetrahydrofuran (d_8-THF) were dried over activated 3 Å molecular sieves. [Ar'GeCl]$_2$ (Ar' = 2,6-Dipp$_2$-C_6H_3, Dipp = 2,6-^{i}Pr$_2$-C_6H_3)[1] and [Li(DME)]$_2$[Fe(COD)$_2$] (DME = 1,2-dimethoxyethane, COD = 1,5-cyclooctadiene)[2] were synthesized according to literature procedures. All other chemicals were purchased from vendors. ^{t}BuNC, PMe_3, and 2,3-dimethyl-1,3-butadiene (DMB) were dried over activated 3 Å molecular sieves. Styrene was dried over CaH_2 and distilled. All these chemicals were stored under an inert atmosphere at −30 °C before use. Dihydrogen (H_2) gas (grade 4.8) was purchased from Linde-Gas and used as received. NMR spectra were recorded with Bruker Avance 400 MHz or 500 MHz spectrometers. All chemical shifts are reported in units of ppm and referenced to the residual ^{1}H NMR signals of the deuterated solvents for proton chemical shifts and to the ^{13}C{^{1}H} NMR signals of deuterated solvents for carbon chemical shifts. The assignments of the ^{1}H and ^{13}C{^{1}H} NMR signals were confirmed by two-dimensional (COSY, NOESY, HSQC, HMBC) and DEPT experiments. UV-vis spectra were recorded on an Ocean Optics DH-2000-BAL Spectrometer. Elemental analyses were determined by the analytical department of the University of Regensburg with a vario MICRO cube instrument. IR spectra were recorded with a Bruker ALPHA spectrometer equipped with a diamond ATR unit.

Synthesis of Complexes 1-7

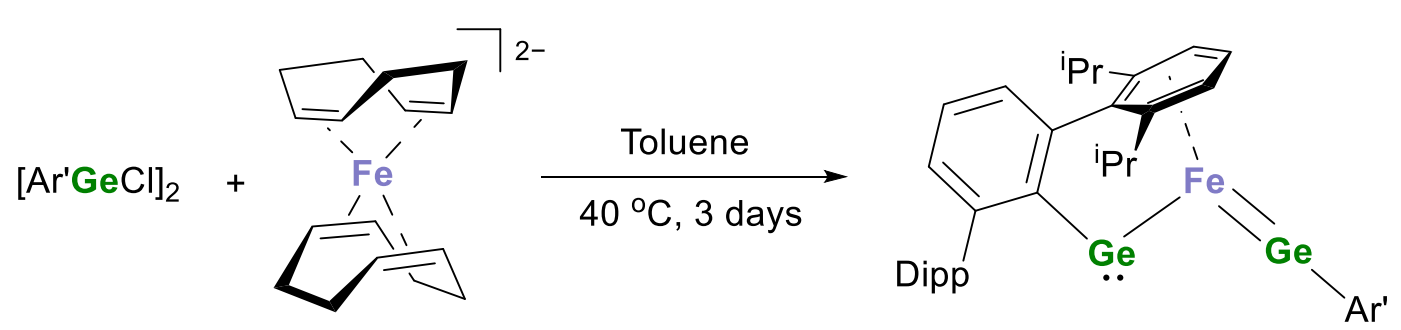

Synthesis of [Fe(GeAr')$_2$] (1). To a pre-cooled (−30 °C) toluene (40 mL) solution of [Ar'GeCl]$_2$ (405 mg, 0.4 mmol) in a glass vial was added [Li(DME)]$_2$[Fe(COD)$_2$] (200 mg, 0.43 mmol) portionwise over 10 minutes. The resulting dark green solution was allowed to warm to room temperature and was stirred for 2 h. The mixture was then transferred to a Schlenk tube and heated to 40 °C, where it was stirred for 3 days. During this period, the color gradually changed from dark green to dark brown. The solvent was removed under reduced pressure to afford a black residue, which was washed with *n*-pentane (3 × 2 mL) and then extracted with benzene (3 × 3 mL). Slow evaporation of the combined benzene solution at room temperature afforded the product **1** as dark brown crystals. The isolated crystals were washed with *n*-hexane (3 × 1 mL) and dried under vacuum. Yield: 180 mg, 45%. Notes: The digermyne side product, [Ar'GeGeAr'], may co-crystallize with complex **1**. Its presence and relative amount can be determined by ^{1}H NMR spectroscopy. This impurity can be reduced or removed by repeated recrystallization from benzene. **^{1}H NMR** (500 MHz, C_6D_6, 298 K): δ (ppm) 0.44 (d, *J* = 6.9 Hz, 6H, −CH(C*H*$_3$)$_2$ of η^6-Dipp), 0.95 (d, *J* = 6.9 Hz, 6H, −CH(C*H*$_3$)$_2$), 1.05 (d, *J* = 6.8 Hz, 6H, −CH(C*H*$_3$)$_2$ of η^6-Dipp), 1.12 (d, *J* = 6.8 Hz, 12H, −CH(C*H*$_3$)$_2$), 1.21 (d, *J* = 6.9 Hz, 6H, −CH(C*H*$_3$)$_2$), 1.27 (d, *J* = 7.0 Hz, 12H, −CH(C*H*$_3$)$_2$), 2.27 (sept, *J* = 7.0 Hz, 2H, −C*H*(CH$_3$)$_2$ of η^6-Dipp), 2.96 (sept, *J* = 7.0 Hz, 2H, −C*H*(CH$_3$)$_2$), 3.01 (sept, *J* = 7.0 Hz, 4H, −C*H*(CH$_3$)$_2$), 4.56 (d, *J* = 6.5 Hz, 2H, *m*-C*H*-η^6-Dipp), 5.32 (t, *J* = 6.5 Hz, 1H, *p*-C*H*-η^6-Dipp), 7.02–7.06 (m, 3H), 7.10–7.13 (m, 1H), 7.14 (d, *J* = 7.5 Hz, 2H), 7.23 (d, *J* = 8.0 Hz, 2H), 7.26 (t, *J* = 7.5 Hz, 1H), 7.32 (t, *J* = 7.0 Hz, 1H), 7.36 (t, *J* = 7.5 Hz, 1H), 7.40 (dd, *J* = 7.5, 1.5 Hz, 1H), 7.78 (dd, *J* = 7.5, 1.5 Hz, 1H). Due to overlapping signals in the aromatic region, the full assignment of the signals of the aryl rings was not possible. Signals of two

H atoms on aryl rings overlap with the residual solvent peak of C_6D_6. **$^{13}C\{^1H\}$ NMR** (125 MHz, C_6D_6, 298 K): δ (ppm) 169.6 (C_{ipso}–Ge), 158.0 (C_{ipso}–Ge), 147.3 (*o-C*-Dipp), 146.6 (*o-C*-Dipp), 145.8 (*o-C*-Dipp), 144.7 (*o-C*-Dipp), 143.4 (*o-C*-Dipp), 142.2 (*i-C*-Dipp), 138.4 (Dipp-*C*-Ar), 129.4, 129.3, 129.2, 129.0, 128.5, 128.0, 126.5, 123.5 (*m-C*-Dipp), 122.6 (*m-C*-Dipp), 115.8 (*i-C-*η^6-Dipp), 111.4 (*o-C-*η^6-Dipp), 84.2 (*p-C-*η^6-Dipp), 78.6 (*m-C-*η^6-Dipp), 31.4 (−*C*H(CH_3)$_2$), 31.1 (−*C*H(CH_3)$_2$), 28.6 (−*C*H(CH_3)$_2$), 25.8 (−CH($\mathit{C}H_3$)$_2$), 25.7 (−CH($\mathit{C}H_3$)$_2$), 24.8 (−CH($\mathit{C}H_3$)$_2$), 24.7 (−CH($\mathit{C}H_3$)$_2$), 24.5 (−CH($\mathit{C}H_3$)$_2$), 23.8 (−CH($\mathit{C}H_3$)$_2$). **Elemental Analysis**: Calcd. for $C_{60}H_{74}Ge_2Fe\cdot0.5C_6H_6$: C 73.08, H 7.50; Found: C 73.03, H 7.62. **UV-vis** (toluene, 298 K): λ_{max}, nm (ε, M^{-1} cm^{-1}) 380 (10700), 500 (shoulder, 1780), 600 (broad, 750), 780 (broad, 250). **^{57}Fe Mössbauer** (solid state, 80 K): δ = 0.51 mm s^{-1}, $|\Delta E_Q|$ = 0.69 mm s^{-1}.

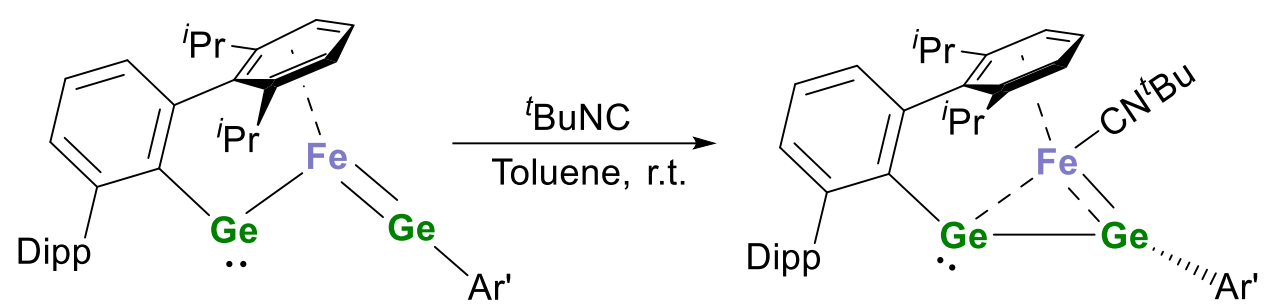

1 2

Synthesis of [(^{t}BuNC)Fe(GeAr')$_2$] (2). To a dark brown toluene (5 mL) solution of **1** (50 mg, 0.05 mmol) in a glass vial was added ^{t}BuNC (6.3 mg, 0.08 mmol) at room temperature. The solution immediately turned dark green and was further stirred at room temperature for 12 h. The volatiles were then removed under reduced pressure to afford **2** as a green powder in near-quantitative yield. Green crystals suitable for single-crystal X-ray diffraction were obtained by slow evaporation of an *n*-pentane solution of **2** at room temperature. **^{1}H NMR** (500 MHz, C_6D_6, 298 K): δ (ppm) 0.44 (d, *J* = 6.9 Hz, 6H, −CH(CH_3)$_2$ of η^6-Dipp), 0.89 (d, *J* = 6.7 Hz, 6H, −CH(CH_3)$_2$), 1.09 (d, *J* = 6.7 Hz, 6H, −CH(CH_3)$_2$ of η^6-Dipp), 1.12 (d, *J* = 6.9 Hz, 6H, −CH(CH_3)$_2$), 1.17 (d, *J* = 6.8 Hz, 6H, −CH(CH_3)$_2$), 1.23 (d, *J* = 6.9 Hz, 6H, −CH(CH_3)$_2$), 1.27 (s, 9H, −C(CH_3)$_3$), 1.40 (d, *J* = 6.7 Hz, 6H, −CH(CH_3)$_2$), 1.51 (d, *J* = 6.7 Hz, 6H, −CH(CH_3)$_2$), 2.73 (sept, *J* = 7.0 Hz, 2H, −C*H*(CH_3)$_2$ of η^6-Dipp), 2.85 (sept, *J* = 7.0 Hz, 2H, −C*H*(CH_3)$_2$), 2.98 (sept, *J* = 7.0 Hz, 2H, −C*H*(CH_3)$_2$), 3.55 (sept, *J* = 7.0 Hz, 2H, −C*H*(CH_3)$_2$), 4.35 (d, *J* = 6.5 Hz, 2H, *m-CH-*η^6-Dipp), 4.76 (t, *J* = 6.5 Hz, 1H, *p-CH-*η^6-Dipp), 6.91 (dd, *J* = 7.5, 1.5 Hz, 2H), 7.02–7.06 (m, 3H), 7.15 (dd, *J* = 7.5, 1.5 Hz, 2H), 7.21 (d, *J* = 8.0 Hz, 3H), 7.28 (dd, *J* = 7.5, 1.5 Hz, 1H), 7.36–7.44 (m, 3H). Due to overlapping signals in the aromatic region, the full assignment of the signals of the aryl rings was not possible. Signal of one H atom on an aryl ring overlaps with the residual solvent peak of C_6D_6. **$^{13}C\{^1H\}$ NMR** (125 MHz, C_6D_6, 298 K): δ (ppm) 169.0, 164.3, 153.6, 147.5, 146.8, 146.7, 145.5, 145.4, 141.5, 140.6, 140.1, 129.6, 129.3, 128.3, 128.0, 126.7, 125.9, 123.6, 123.4, 123.1, 109.9 (*i-C-*η^6-Dipp), 107.9 (*o-C-*η^6-Dipp), 89.2 (*p-C-*η^6-Dipp), 85.6 (*m-C-*η^6-Dipp), 57.1 (^{t}BuN*C*), 31.4 (−C(CH_3)$_3$), 31.3 (−*C*H(CH_3)$_2$), 30.8 (−*C*H(CH_3)$_2$), 30.1 (−*C*H(CH_3)$_2$), 29.7 (−*C*H(CH_3)$_2$), 27.0 (−CH($\mathit{C}H_3$)$_2$), 26.1 (−CH($\mathit{C}H_3$)$_2$), 25.7 (−CH($\mathit{C}H_3$)$_2$), 25.5 (−CH($\mathit{C}H_3$)$_2$), 25.1 (−CH($\mathit{C}H_3$)$_2$), 24.0 (−CH($\mathit{C}H_3$)$_2$), 23.9 (−CH($\mathit{C}H_3$)$_2$), 19.8 (−CH($\mathit{C}H_3$)$_2$). **Elemental Analysis**: Calcd. for $C_{65}H_{83}NGe_2Fe$: C 72.32, H 7.75, N 1.30; Found: C 72.44, H 7.59, N 1.19. **UV-vis** (toluene, 298 K): λ_{max}, nm (ε, M^{-1} cm^{-1}) 370 (shoulder, 19400), 420 (shoulder, 12460), 470 (shoulder, 6950), 590 (1460), 790 (2200). **ATR–IR** (powder, cm^{-1}): v = 2025 (*NC*).

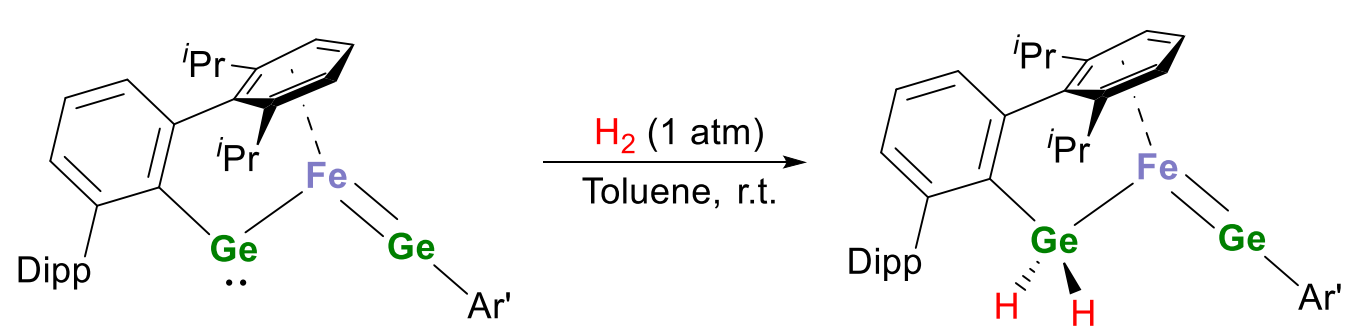

1 3

Synthesis of [(Ar'GeH$_2$)Fe(GeAr')] (3). To a dark brown benzene (3 mL) solution of **1** (50 mg, 0.05 mmol) in a Schlenk tube (equipped with a high vacuum valve plug) was injected H_2 gas (1 atm). The solution was shaken vigorously several times. The color of the solution gradually changed to dark green over a period of *ca.* 10 min. The reaction mixture was then kept at room temperature without stirring for 2 days, during which dark green crystals of **3** formed on the bottom and walls of the Schlenk tube. The crystals

were isolated by decanting the supernatant. After removing the solvent from the supernatant, the resulting green residue was extracted with toluene (3 × 1 mL). Storage of the combined toluene extracts at −30 °C for 2 days afforded a second crop of crystals. Total yield: 48 mg, 96%. **^{1}H NMR** (500 MHz, C_6D_6, 298 K): *δ* (ppm) 0.97 (d, *J* = 6.8 Hz, 6H, −CH(C*H*$_3$)$_2$ of *η*^{6}-Dipp-Fe), 1.07 (d, *J* = 6.8 Hz, 12H, −CH(C*H*$_3$)$_2$), 1.13 (d, *J* = 6.9 Hz, 6H, −CH(C*H*$_3$)$_2$ of *η*^{6}-Dipp-Fe), 1.18 (d, *J* = 6.8 Hz, 6H, −CH(C*H*$_3$)$_2$), 1.45 (d, *J* = 7.2 Hz, 12H, −CH(C*H*$_3$)$_2$), 1.46 (d, *J* = 7.5 Hz, 6H, −CH(C*H*$_3$)$_2$), 2.23 (sept, *J* = 7.0 Hz, 2H, −C*H*(CH$_3$)$_2$ of *η*^{6}-Dipp-Fe), 2.81 (sept, *J* = 7.0 Hz, 4H, −C*H*(CH$_3$)$_2$), 2.88 (sept, *J* = 7.0 Hz, 2H, −C*H*(CH$_3$)$_2$), 3.14 (s, 2H, −Ge*H*), 5.34–5.45 (m, 3H, *m*- and *p*-C*H*-*η*^{6}-Dipp-Fe), 6.81 (d, *J* = 7.5 Hz, 2H, *m*-Ar'*H*), 7.18–7.23 (m, 6H), 7.38 (d, *J* = 7.5 Hz, 2H, *m*-Ar'*H*), 7.50 (t, *J* = 7.5 Hz, 1H). Due to overlapping signals in the aromatic region, the full assignment of the signals of the aryl rings was not possible. Signals of four H atoms on aryl rings overlap with the residual solvent peak of C_6D_6. **^{13}C{^{1}H} NMR** (125 MHz, C_6D_6, 298 K): *δ* (ppm) 163.0 (*i-C* of ArGe=Fe), 154.7(*i-C* of ArGe–Fe), 147.9 (*o-C* of Dipp-ArGe=Fe), 147.0 (*o-C* of Dipp-ArGe–Fe), 146.9 (*o-C* of ArGe–Fe), 145.0 (*o-C* of ArGe–Fe), 142.8 (*i-C* of ArGe=Fe), 140.5 (*i-C* of Dipp-ArGe–Fe), 136.1 (*o-C* of ArGe=Fe), 130.5 (*p-C* of Dipp-ArGe=Fe), 128.9 (*m-C* of ArGe=Fe), 128.3 (*p-C* of Dipp-ArGe–Fe), 128.1 (*p-C* of ArGe=Fe), 127.8 (*m-C* of ArGe–Fe), 127.2 (*m-C* of ArGe–Fe), 125.5 (*p-C* of ArGe–Fe), 124.1 (*m-C* of Dipp-ArGe=Fe), 122.7 (*m-C* of Dipp-ArGe–Fe), 115.6 (*i-C-η*^{6}-Dipp), 107.8 (*o*-Ar of *η*^{6}-Dipp), 78.3 (*m-C-η*^{6}-Dipp), 76.7 (*p-C-η*^{6}-Dipp), 31.2 (−*C*H(CH$_3$)$_2$), 31.1 (−*C*H(CH$_3$)$_2$), 28.9 (−*C*H(CH$_3$)$_2$), 25.9 (−CH(*C*H$_3$)$_2$ of *η*^{6}-Dipp), 25.8 (−CH(*C*H$_3$)$_2$), 25.3 (−CH(*C*H$_3$)$_2$), 23.63 (−CH(*C*H$_3$)$_2$), 23.60 (−CH(*C*H$_3$)$_2$), 23.5 (−CH(*C*H$_3$)$_2$ of *η*^{6}-Dipp). **Elemental Analysis**: Calcd. for $C_{60}H_{76}Ge_2Fe{\cdot}C_6H_6$: C 73.64, H 7.68; Found: C 73.34, H 7.33. **UV-vis** (toluene, 298 K): λ_{max} (nm) 420 (strong), 530, 630 (broad). The poor solubility of **3** in toluene prevented accurate determination of its concentration; therefore, the absorption coefficient was not calculated. **ATR–IR** (powder, cm^{-1}): *ν* = 2002 (Ge*H*).

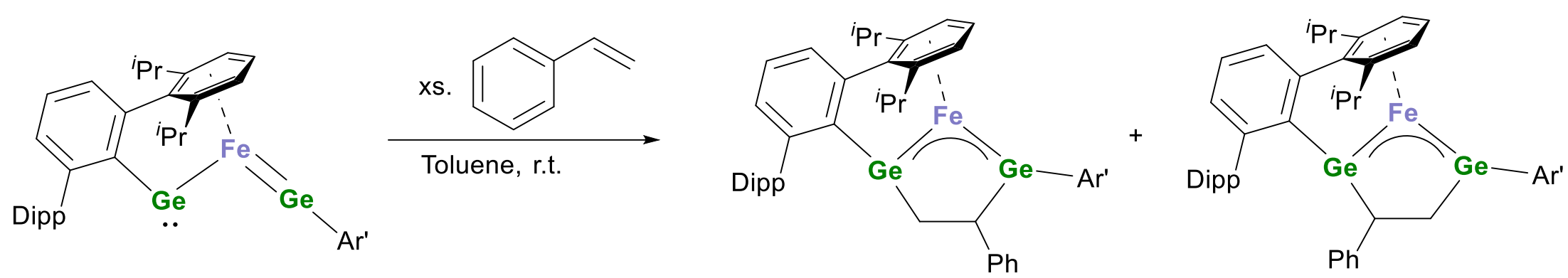

1 **4a** **4a** : **4b** = 1 : 0.7 **4b**

Synthesis of [(Ar'GeCH$_2$(Ph)CHGeAr')Fe] (4).

NMR-scale: To a dark brown C_6D_6 (0.5 mL) solution of **1** (6.0 mg, 0.006 mmol) in a *J. Young* NMR tube was added an excess of styrene (4.2 mg, 0.04 mmol). The solution gradually turned dark green over a period of *ca.* 10 min. The reaction was monitored by ^{1}H NMR spectroscopy, which showed the clean conversion of **1** to **4a** and **4b** in an approximate ratio of 1:0.7 within 8 h (Figure S11).

Preparative-scale: A toluene solution (3 mL) of **1** (50 mg, 0.05 mmol) was treated with styrene (10.4 mg, 0.10 mmol) and stirred at room temperature overnight. The solvent was removed under vacuum, and the resulting green solid was extracted with *n*-pentane (2 × 1 mL). Slow evaporation of the *n*-pentane solution at room temperature afforded green crystals containing both **4a** and **4b**, as confirmed by single-crystal X-ray diffraction analysis, in near-quantitative overall yield. The green crystals were further washed with cold *n*-pentane (−30 °C, 2 × 0.1 mL), and then were dried under vacuum. The ^{1}H NMR spectrum of the final crystalline material revealed **4a** as the major component (**4a**/**4b** = 100:7). Yield: 32 mg, 58%. **^{1}H NMR** (400 MHz, d_8-THF, 268 K) of **4a**: *δ* (ppm) −0.60 (dd, *J* = 13.9, 8.2 Hz, 1H, PhC*H*), 0.00 (d, *J* = 6.7 Hz, 3H, −CH(C*H*$_3$)$_2$), 0.34 (d, *J* = 6.9 Hz, 3H, −CH(C*H*$_3$)$_2$), 0.38 (d, *J* = 14.0 Hz, 1H, −C*H*$_2$CHPh), 0.59 (d, *J* = 6.7 Hz, 3H, −CH(C*H*$_3$)$_2$), 0.65 (d, *J* = 7.0 Hz, 3H, −CH(C*H*$_3$)$_2$), 0.77 (d, *J* = 6.7 Hz, 3H, −CH(C*H*$_3$)$_2$), 0.85 (d, *J* = 6.9 Hz, 3H, −CH(C*H*$_3$)$_2$), 0.88 (d, *J* = 6.9 Hz, 6H, −CH(C*H*$_3$)$_2$), 0.96 (d, *J* = 6.8 Hz, 6H, −CH(C*H*$_3$)$_2$), 1.10 (d, *J* = 6.7 Hz, 3H, −CH(C*H*$_3$)$_2$), 1.14 (d, *J* = 7.0 Hz, 3H, −CH(C*H*$_3$)$_2$), 1.16 (d, *J* = 7.0 Hz, 3H, −CH(C*H*$_3$)$_2$), 1.19 (d, *J* = 6.9 Hz, 3H, −CH(C*H*$_3$)$_2$), 1.35 (d, *J* = 6.9 Hz, 3H, −CH(C*H*$_3$)$_2$),

1.51 (d, J = 6.9 Hz, 3H, −CH(CH_3)$_2$), 1.82 (sept, J = 6.8 Hz, 1H, −CH(CH$_3$)$_2$), 2.19 (sept, J = 6.8 Hz, 1H, −CH(CH$_3$)$_2$), 2.30 (sept, J = 6.8 Hz, 1H, −CH(CH$_3$)$_2$), 2.58 (sept, J = 6.8 Hz, 1H, −CH(CH$_3$)$_2$), 2.64 (sept, J = 6.8 Hz, 1H, −CH(CH$_3$)$_2$), 2.95 (sept, J = 6.8 Hz, 1H, −CH(CH$_3$)$_2$), 3.35 (sept, J = 6.8 Hz, 1H, −CH(CH$_3$)$_2$), 3.48 (sept, J = 6.8 Hz, 1H, −CH(CH$_3$)$_2$), 3.58 (d, J = 13.9 Hz, 1H, −CH_2CHPh), 3.64 (d, J = 5.9 Hz, 1H, *m*-C*H*-η^6-Dipp), 4.56 (d, J = 5.8 Hz, 1H, *m*-C*H*-η^6-Dipp), 4.97 (t, J = 5.9 Hz, 1H, *p*-C*H*-η^6-Dipp), 6.18 (d, J = 7.2 Hz, 2H), 6.60–6.66 (m, 1H), 6.66–6.74 (m, 3H), 6.78 (d, J = 7.6 Hz, 1H), 7.01–7.08 (m, 2H), 7.08–7.17 (m, 3H), 7.17–7.26 (m, 2H), 7.30 (d, J = 8.0 Hz, 1H), 7.33–7.40 (m, 2H), 7.46 (t, J = 7.5 Hz, 1H), 7.50 (d, J = 7.7 Hz, 1H), 7.55 (t, J = 7.5 Hz, 1H). Due to overlapping signals in the aromatic region, the full assignment of the signals of the aryl rings was not possible. **^{13}C{^{1}H} NMR** (100 MHz, d_8-THF, 268 K): δ (ppm) 168.2, 161.0, 149.1, 148.2, 147.8, 147.5, 146.9, 146.8, 146.4, 145.6, 143.4, 142.4, 142.0, 141.6, 139.5, 131.5, 131.0, 129.2, 129.0, 128.8, 128.7, 127.9, 127.9, 127.5, 127.3, 124.2, 124.1, 123.5, 123.3, 123.2, 122.9, 122.8, 117.1, 117.0, 104.5, 82.9, 79.7, 75.7, 57.7, 37.1, 32.4, 31.9, 31.4, 31.3, 30.8, 30.8, 30.8, 28.0, 27.1, 26.5, 25.8, 25.4, 25.6, 25.5, 25.4, 25.0, 24.9, 24.5, 24.3, 24.1, 23.8, 23.3, 22.8, 21.1. **Elemental Analysis**: Calcd. for $C_{68}H_{82}Ge_2Fe$: C 74.22, H 7.51; Found: C 74.35, H 7.16. **UV-vis** (toluene, 298 K): λ_{max}, nm (ε, M^{-1} cm^{-1}) 400 (shoulder, 8630), 450 (14700), 600 (broad, 1830). Attempts to isolate **4b** in pure form were unsuccessful and consistently afforded mixtures of **4a** and **4b**. Figure S11 shows a comparison of the ^{1}H NMR spectrum of **4a** and **4a**/**b**.

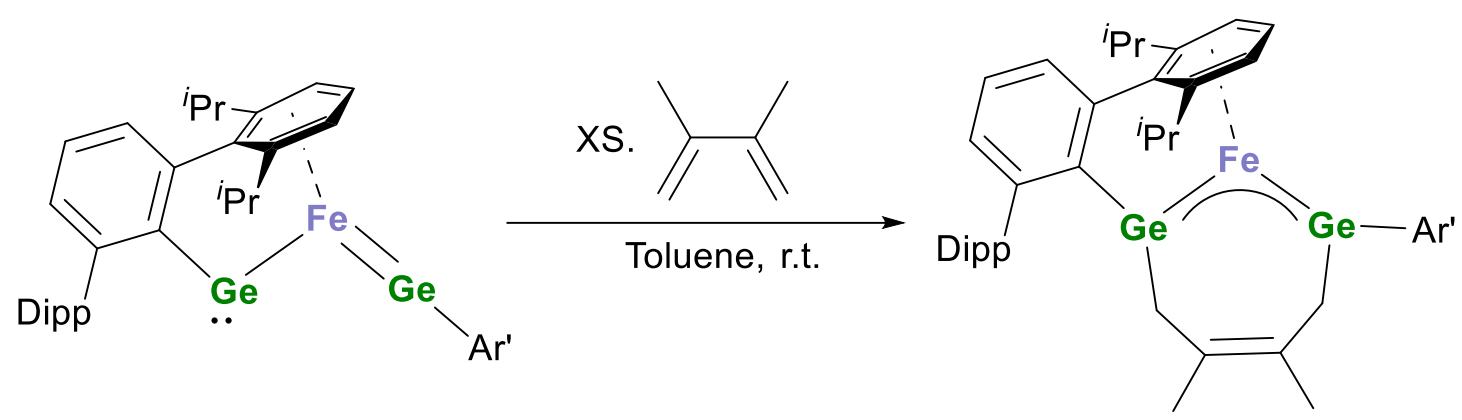

1 **5**

Synthesis of [(Ar'GeCH$_2$(CH$_3$)CC(CH$_3$)CH$_2$GeAr')Fe] (5). To a dark brown toluene (3 mL) solution of **1** (50 mg, 0.05 mmol) in a glass vial was added an excess of DMB (8.2 mg, 0.1 mmol) at room temperature. The solution gradually turned brown, and the reaction mixture was stirred at room temperature overnight. After removing the solvent under vacuum, the resulting brown powder was extracted with *n*-pentane (2 × 0.1 mL). Slow evaporation of this brown *n*-pentane solution at room temperature afforded **5** as brown crystals. Yield: 46 mg, 86%. **^{1}H NMR** (400 MHz, d_8-THF, 223 K): δ (ppm) 0.69 (d, J = 6.8 Hz, 6H, −CH(CH_3)$_2$), 0.75 (d, J = 7.0 Hz, 3H, −CH(CH_3)$_2$), 0.85–1.01 (m, 15H), 1.03 (d, J = 6.6 Hz, 3H, −CH(CH_3)$_2$), 1.11 (d, J = 6.8 Hz, 3H, −CH(CH_3)$_2$), 1.13–1.23 (m, 15H), 1.24–1.29 (d+s, 6H, −CH(CH_3)$_2$ and -C(CH_3)CH$_2$Ge), 1.30–1.38 (m, 4H), 1.54 (d, J = 13.7 Hz, 1H, -C(CH$_3$)CH_2Ge), 1.58 (d, J = 6.9 Hz, 3H, −CH(CH_3)$_2$), 1.62–1.72 (m, 2H), 1.90 (d, J = 12.9 Hz, 1H, -C(CH$_3$)CH_2Ge), 2.07 (sept, J = 6.8 Hz, 1H, −CH(CH$_3$)$_2$), 2.67 (sept, J = 6.8 Hz, 1H, −CH(CH$_3$)$_2$), 2.79 (sept, J = 6.8 Hz, 2H, −CH(CH$_3$)$_2$), 3.00 (sept, J = 6.8 Hz, 1H, −CH(CH$_3$)$_2$), 3.15 (sept, J = 6.8 Hz, 1H, −CH(CH$_3$)$_2$), 3.66 (sept, J = 6.8 Hz, 1H, −CH(CH$_3$)$_2$), 3.82 (d, J = 5.9 Hz, 1H, *m*-C*H*-η^6-Dipp-Fe), 4.41 (t, J = 5.8 Hz, 1H, *p*-C*H*-η^6-Dipp-Fe), 5.04 (d, J = 5.9 Hz, 1H, *m*-C*H*-η^6-Dipp-Fe), 6.98 (dd, J = 6.2, 2.9 Hz, 1H, *m*-C*H* of Ar'), 7.08 (d, J = 7.5 Hz, 1H, *m*-C*H* of Dipp), 7.15–7.31 (m, 7H), 7.31–7.37 (m, 1H), 7.37–7.48 (m, 3H), 7.50 (t, J = 6.9 Hz, 2H, *p*-C*H* of Dipp), 7.90 (d, J = 7.4 Hz, 1H, *m*-C*H* of Ar'). Residual *n*-pentane signals account for approximately three protons by integration overlap with the −CH(CH$_3$)$_2$ signals, resulting in an apparent proton count higher than expected for **5** (Figure S15). Due to signal overlap in the aromatic and alkyl regions, full assignment of the resonances was not possible. **^{13}C{^{1}H} NMR** (100 MHz, d_8-THF, 223 K): δ (ppm) 169.3, 166.1, 148.6, 147.3, 147.2, 147.0, 146.8, 146.8, 144.2, 143.6, 143.1, 143.1, 142.5, 140.9, 139.4, 131.7, 130.9, 129.9, 129.9, 129.1, 128.7, 128.7, 127.3, 127.1, 126.6, 124.5, 123.6, 123.6, 123.4, 122.8, 120.9, 117.8, 116.4, 108.9, 82.5, 78.6, 75.3, 54.9, 41.5, 32.0, 31.9, 31.3, 31.2, 31.1, 31.0, 30.5, 29.9, 27.8, 26.8, 26.4, 26.3, 26.2, 26.0, 24.6, 24.5, 24.2, 23.9, 23.7, 23.5, 22.8, 21.9, 21.6. **Elemental Analysis**: Calcd. for $C_{66}H_{84}Ge_2Fe$: C 73.50, H 7.85; Found: C 73.47, H 7.87. **UV-vis** (toluene, 298 K): λ_{max}, nm (ε, M^{-1} cm^{-1}) 330 (shoulder, 7860), 450 (11680), 600 (broad, 3900).

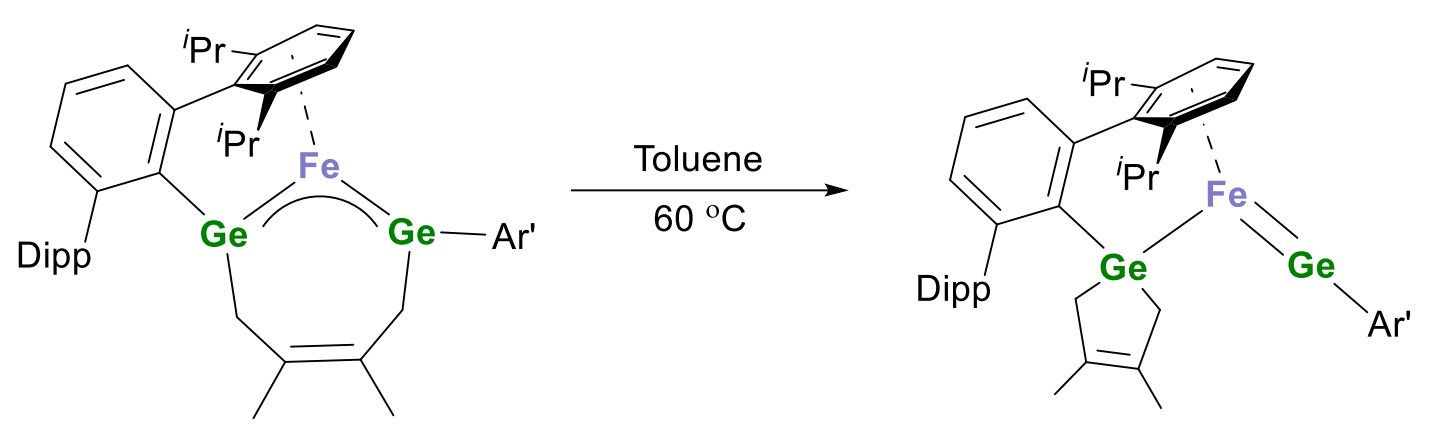

5 6

Synthesis of [(Ar′(CH$_2$(CH$_3$)CC(CH$_3$)CH$_2$)GeFe(GeAr′)] (6). A brown C_6D_6 (3 mL) solution of **5** (22 mg, 0.02 mmol) in a *J. Young* NMR tube was heated to 60 °C for 48 h, during which time, the solution gradually turned green. The ^{1}H NMR spectrum showed clean conversion of **5** to **6**. The volatiles were removed under reduced pressure to afford **6** as a green powder in near-quantitative yield. Green crystals suitable for single-crystal X-ray diffraction were obtained by slow evaporation of an *n*-pentane solution of **6** at room temperature. **^{1}H NMR** (500 MHz, C_6D_6, 298 K): δ (ppm) 0.71 (d, J = 16 Hz, 2H, C(CH$_3$)CH_2Ge), 1.057 (d, J = 7.0 Hz, 6H, −CH(CH_3)$_2$ of η^6-Dipp), 1.063 (d, J = 7.0 Hz, 12H, −CH(CH_3)$_2$), 1.17 (d, J = 7.0 Hz, 6H, −CH(CH_3)$_2$), 1.40 (d, J = 7.0 Hz, 12H, −CH(CH_3)$_2$), 1.42 (d, J = 7.0 Hz, 6H, −CH(CH_3)$_2$), 1.53 (s, 6H, C(CH_3)CH$_2$Ge), 2.04 (d, J = 15.5 Hz, 2H, C(CH$_3$)CH_2Ge), 2.25 (sept, J = 7.0 Hz, 2H, −CH(CH$_3$)$_2$ of η^6-Dipp), 2.97 (sept, J = 7.0 Hz, 4H, −CH(CH$_3$)$_2$), 3.00 (sept, J = 7.0 Hz, 2H, −CH(CH$_3$)$_2$), 5.02 (t, J = 6.0 Hz, 1H, *p*-C*H*-η^6-Dipp), 5.44 (d, J = 6.1 Hz, 2H, *m*-C*H*-η^6-Dipp), 6.76 (d, J = 7.5 Hz, 2H, *m*-C*H* of Ar′), 7.09 (t, J = 7.5 Hz, 1H, *p*-C*H* of Ar′), 7.12–7.15 (m, 3H), 7.21 (d, J = 7.8 Hz, 4H, *m*-C*H* of Dipp), 7.26 (d, J = 7.7 Hz, 2H, *m*-C*H* of Dipp), 7.30–7.37 (m, 3H). **^{13}C{^{1}H} NMR** (125 MHz, C_6D_6, 298 K): δ (ppm) 166.5, 158.2, 147.6, 146.5, 144.4, 143.7, 142.2, 141.0, 138.3, 131.7, 131.6, 129.9, 129.7, 127.93, 127.91, 125.8, 125.7, 123.7, 122.7, 117.0, 108.2, 79.2 (*p-C*-η^6-Dipp), 78.9 (*m-C*-η^6-Dipp), 40.5 (C(CH$_3$)CH$_2$Ge), 31.1 (−CH(CH$_3$)$_2$), 30.9 (−CH(CH$_3$)$_2$), 29.1 (−CH(CH$_3$)$_2$ of η^6-Dipp), 26.3 (−CH(CH$_3$)$_2$), 26.1 (−CH(CH$_3$)$_2$), 24.8 (−CH(CH$_3$)$_2$ of η^6-Dipp), 24.0 (−CH(CH$_3$)$_2$), 23.9 (−CH(CH$_3$)$_2$ and C(CH$_3$)CH$_2$Ge, overlap), 20.3 (−CH(CH$_3$)$_2$). **Elemental Analysis**: Calcd. for $C_{66}H_{84}Ge_2Fe\cdot C_7H_8$: C 74.90, H 7.92; Found: C 74.89, H 7.85. **UV-vis** (toluene, 298 K): λ_{max}, nm (ε, M^{-1} cm^{-1}) 350 (13600), 430 (shoulder, 6360), 600 (broad, 700), 680 (broad, 550), 820 (shoulder, 320).

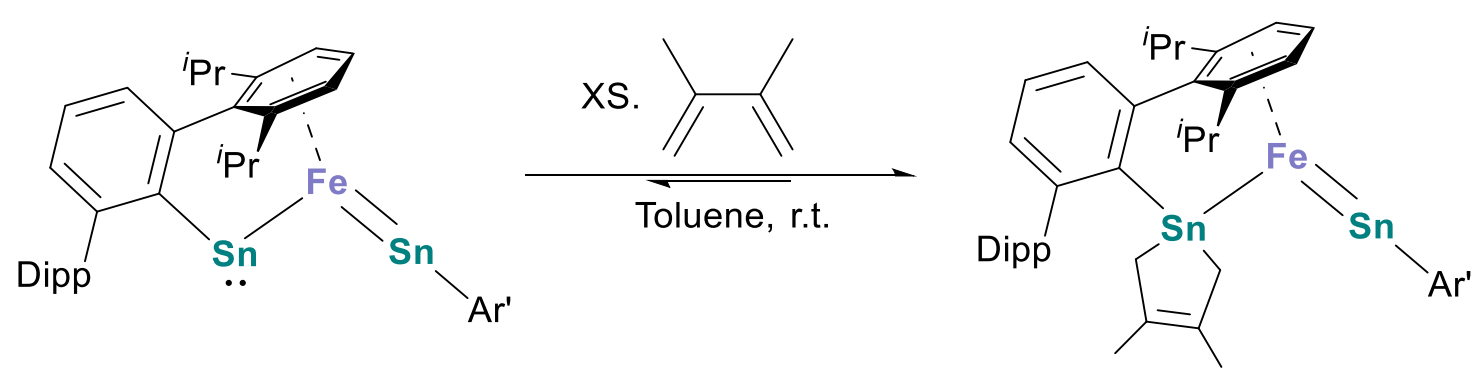

B 7

Synthesis of [(Ar′(CH$_2$(CH$_3$)CC(CH$_3$)CH$_2$)SnFe(SnAr′)] (7). To a dark brown benzene (3 mL) solution of **B** (55 mg, 0.05 mmol) in a glass vial was added an excess of DMB (8.2 mg, 0.1 mmol) at room temperature. The reaction mixture was stirred at room temperature for 12 h, during which time no obvious color change was observed. After removing the solvent under vacuum, the resulting brown solid was extracted with *n*-pentane (2 × 0.2 mL). To prevent the formation of **B**, additional DMB (4.1 mg, 0.05 mmol) was added to the *n*-pentane solution, which was then stored at −30 °C for crystallization. Dark brown crystals of **7** were obtained after two days. Yield: 45 mg, 76%. **^{1}H NMR** (500 MHz, C_6D_6, 298 K): δ (ppm) 0.81 (d, J = 15.7 Hz, 2H, C(CH$_3$)CH_2Sn), 0.99–1.12 (m, 30H, −CH(CH_3)$_2$), 1.31–1.44 (m, 18H, −CH(CH_3)$_2$), 1.65–1.76 (m, 8H, C(CH$_3$)CH_2Sn + C(CH_3)CH$_2$Sn), 2.27 (sept, J = 7.0 Hz, 2H, −CH(CH$_3$)$_2$ of η^6-Dipp), 2.95 (sept, J = 7.0 Hz, 4H, −CH(CH$_3$)$_2$), 3.05 (sept, J = 7.0 Hz, 2H, −CH(CH$_3$)$_2$), 5.51 (d, J = 5.8 Hz, 2H, *m*-C*H*-η^6-Dipp), 5.63 (t, J = 5.8 Hz, 1H, *p*-C*H*-η^6-Dipp), 6.91 (d, J = 7.5 Hz, 2H, *m*-C*H* of Ar′), 7.18–7.30 (m, 10H), 7.33 (t, J = 7.7 Hz, 2H, *p*-C*H* of Dipp), 7.38 (t, J = 7.7 Hz, 1H, *p*-C*H* of Ar′). **^{13}C{^{1}H} NMR** (125 MHz, C_6D_6, 298 K): δ (ppm) 179.2, 156.7, 147.7, 146.7, 146.5, 144.9, 142.7, 141.4, 137.9, 132.3, 130.2, 130.1, 129.9, 128.1, 127.4, 126.2, 125.8, 123.8, 122.6, 116.4, 107.2, 76.9 (*p-C*-η^6-Dipp), 75.7 (*m-C*-η^6-Dipp), 31.3 (C(CH$_3$)CH$_2$)Sn), 30.9 (−CH(CH$_3$)$_2$), 30.8 (−CH(CH$_3$)$_2$), 29.4 (−CH(CH$_3$)$_2$), 26.2 (−CH(CH$_3$)$_2$), 26.0 (−CH(CH$_3$)$_2$), 25.0 (−CH(CH$_3$)$_2$), 24.5

(−CH(CH_3)$_2$), 23.5 (−CH(CH_3)$_2$), 22.7 (C(CH_3)CH_2)Sn), 22.2 (−CH(CH_3)$_2$). **^{119}Sn NMR** (186 MHz, C_6D_6, 298 K): δ (ppm) 688 (s, Sn1), 62 (t, $^2J_{Sn\text{-}H}$ = 40 Hz; coupling to the diastereotopic H atoms of the CH_2 group). **Elemental Analysis**: Calcd. for $C_{66}H_{84}Sn_2Fe$: C 67.72, H 7.23; Found: C 67.63, H 7.02. **UV-vis** (toluene, 298 K): λ_{max}, nm (ε, M^{-1} cm^{-1}) 310 (shoulder, 20190), 380 (20540), 480 (9590), 590 (shoulder, 1520), 690 (shoulder, 960).

NMR Spectra

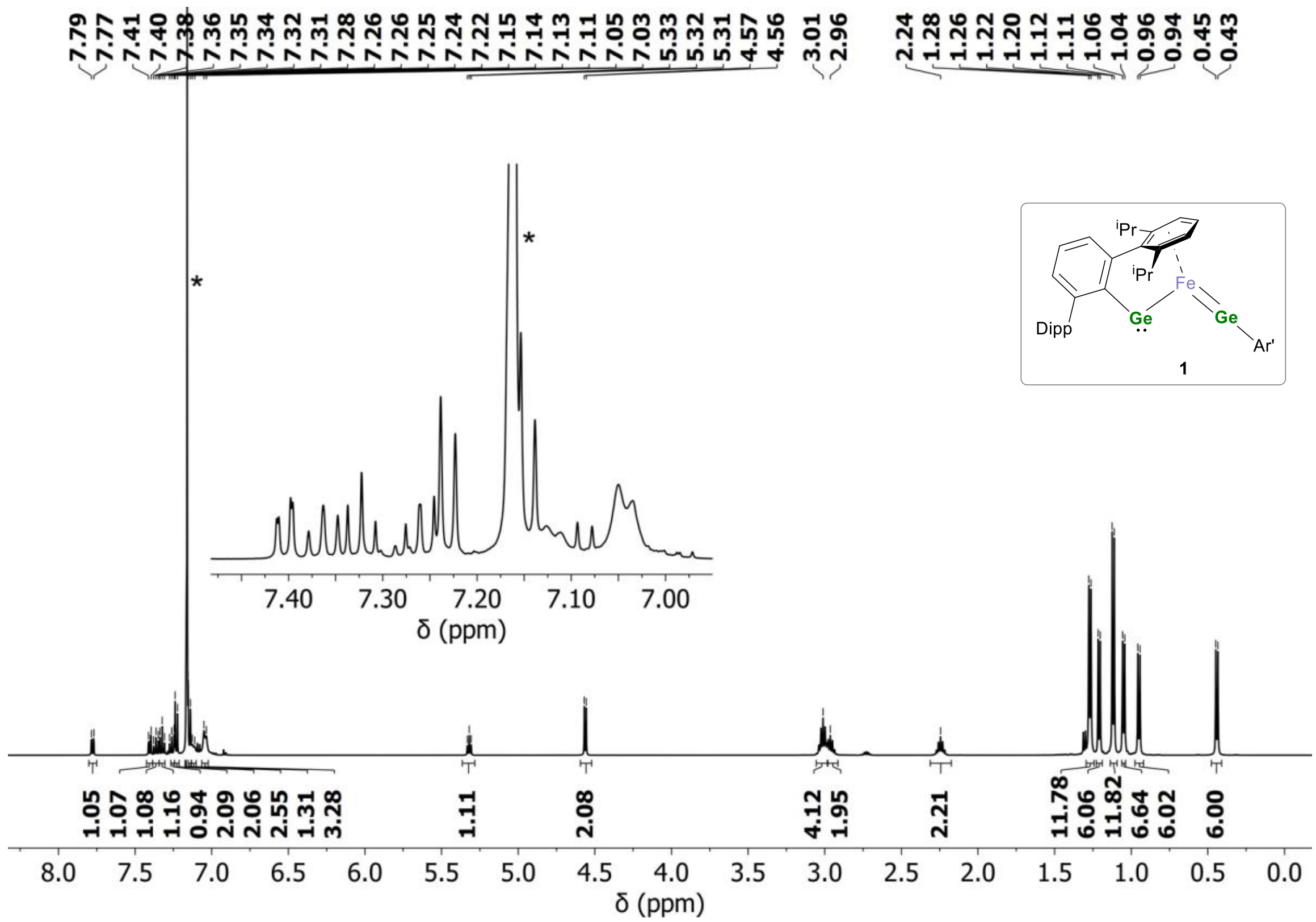

Figure S1. ^{1}H NMR (500 MHz, 298 K) spectrum of [Fe(GeAr')$_2$] (**1**) in C_6D_6. *C_6D_5H.

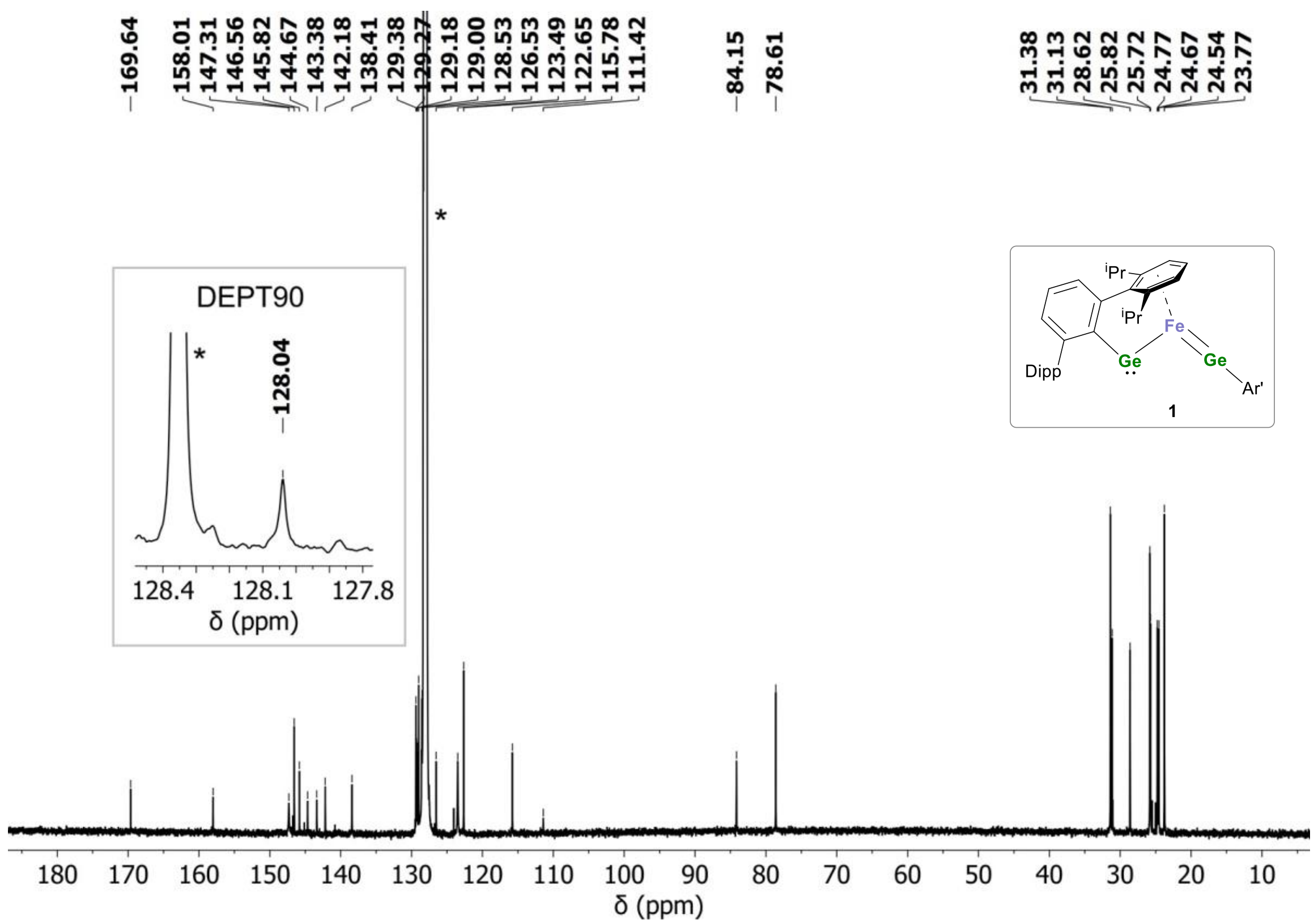

Figure S2. $^{13}C\{^1H\}$ NMR (125 MHz, 298 K) spectrum of [Fe(GeAr')$_2$] (**1**) in C_6D_6. Inset: DEPT90 $^{13}C\{^1H\}$ NMR spectrum of **1** in C_6D_6, showing the signals that overlap with the residual signal of C_6D_6 in the standard $^{13}C\{^1H\}$ NMR spectrum. *C_6D_6.

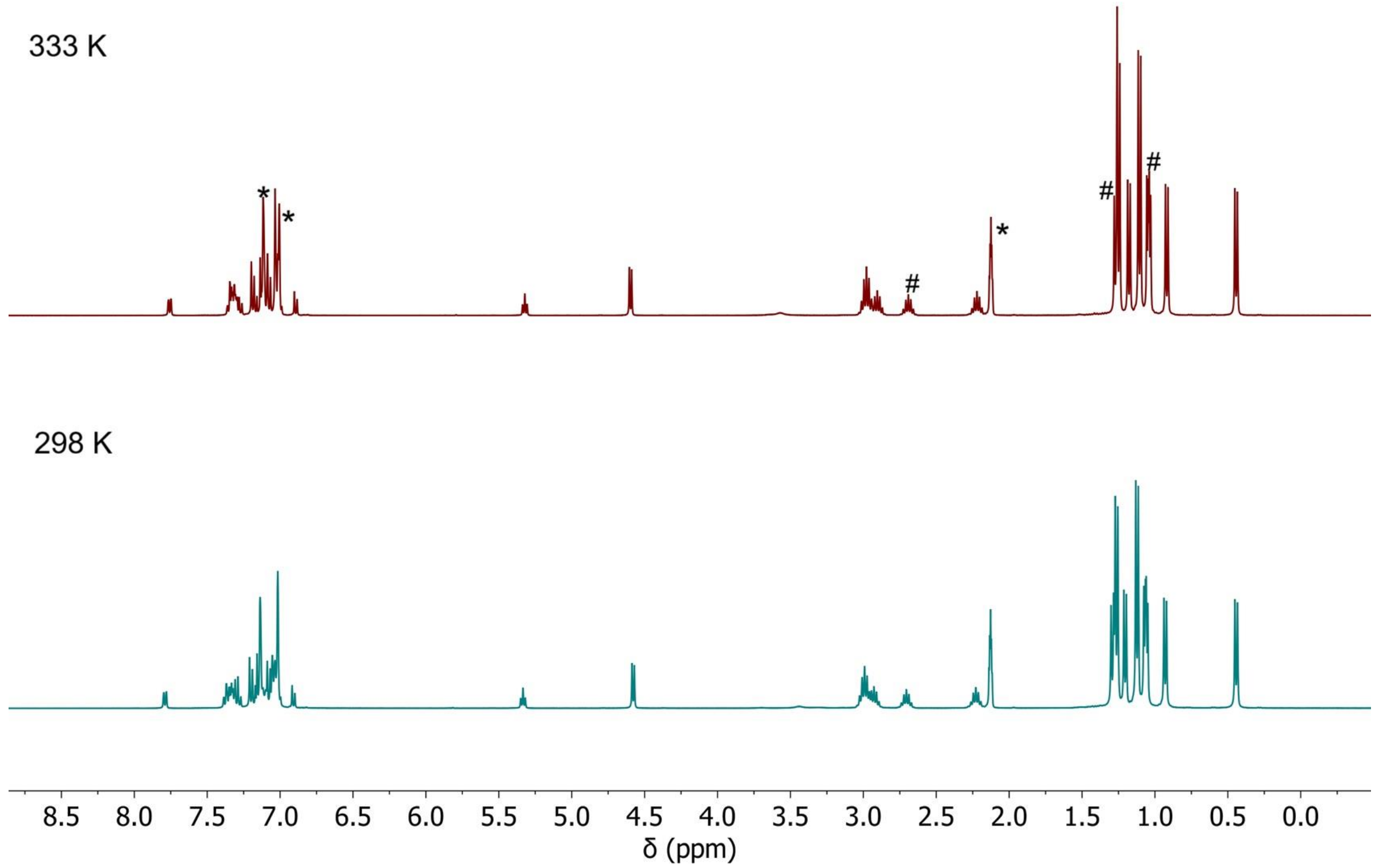

Figure S3. Comparison of ^{1}H NMR (400 MHz) spectra of [Fe(GeAr')2] (**1**) recorded at 298 K and 333 K in d_8-Toluene. *d_8-Toluene, $^{\#}$[Ar'GeGeAr'].

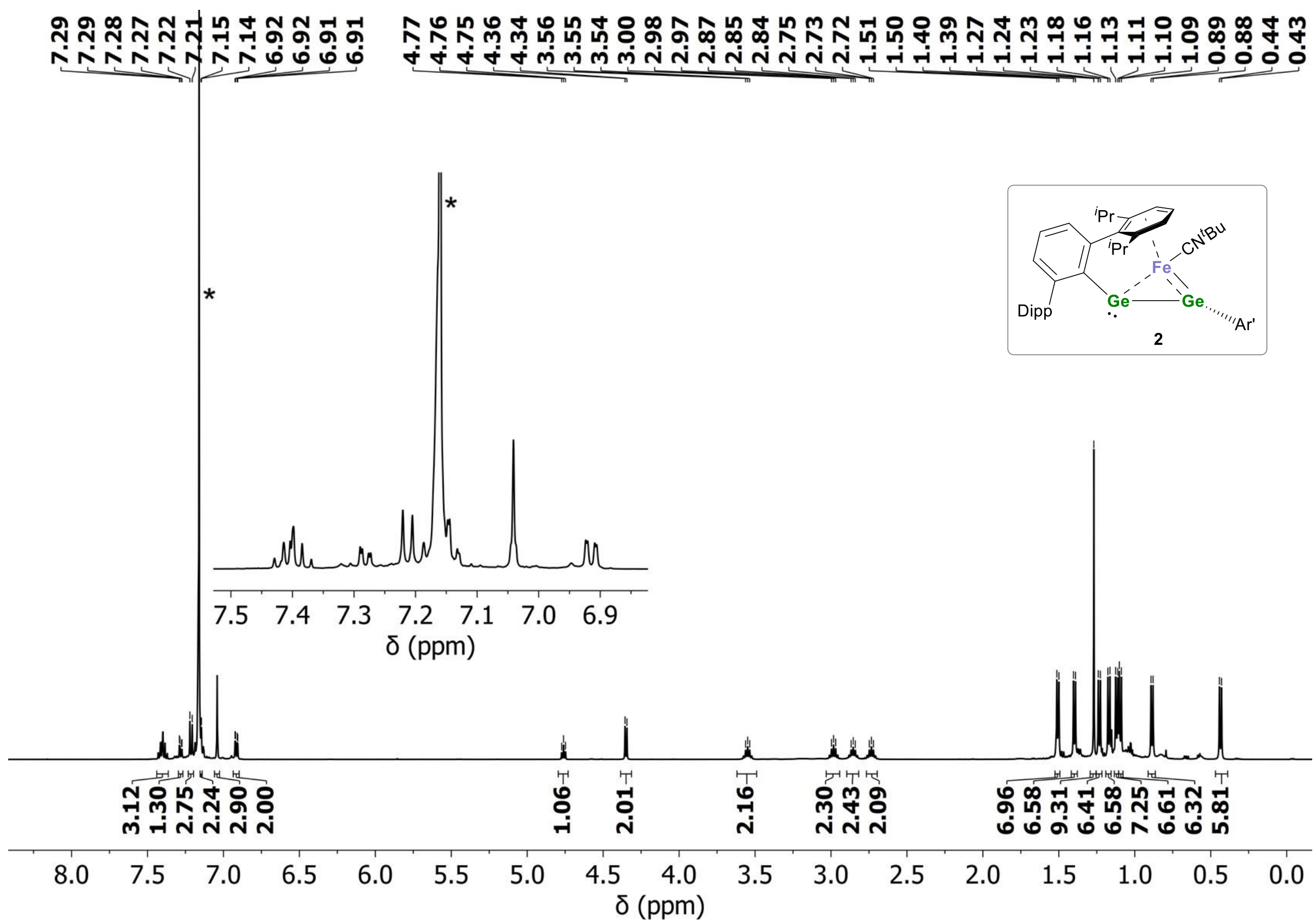

Figure S4. ^{1}H NMR (500 MHz, 298 K) spectrum of [(^{t}BuNC)Fe(GeAr')$_2$] (**2**) in C_6D_6. *C_6D_5H.

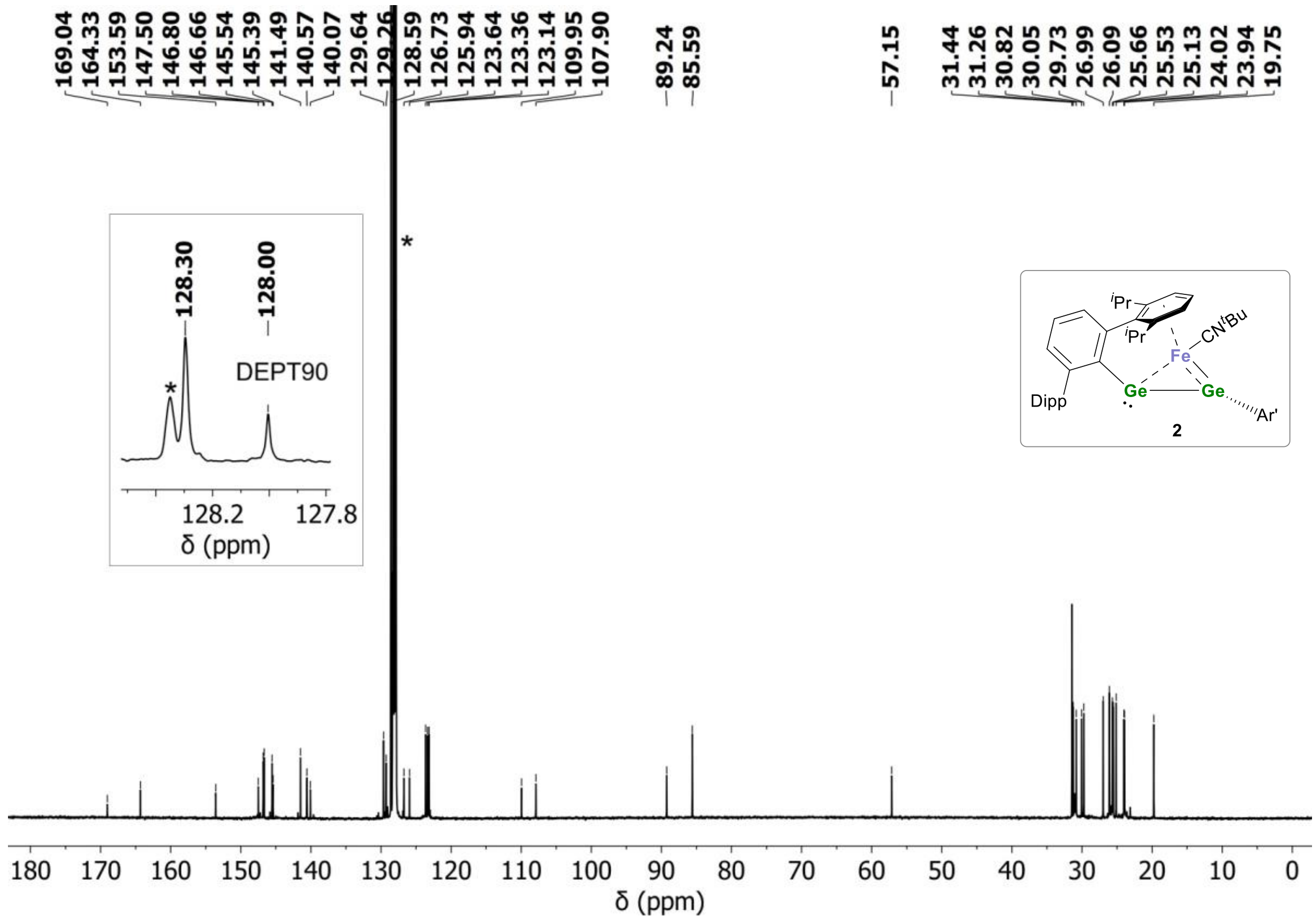

Figure S5. $^{13}C\{^1H\}$ NMR (125 MHz, 298 K) spectrum of $[(^tBuNC)Fe(GeAr')_2]$ (**2**) in C_6D_6. Inset: DEPT90 $^{13}C\{^1H\}$ NMR spectrum of **2** in C_6D_6, showing the signals that overlap with the residual signal of C_6D_6 in the standard $^{13}C\{^1H\}$ NMR spectrum. *C_6D_6.

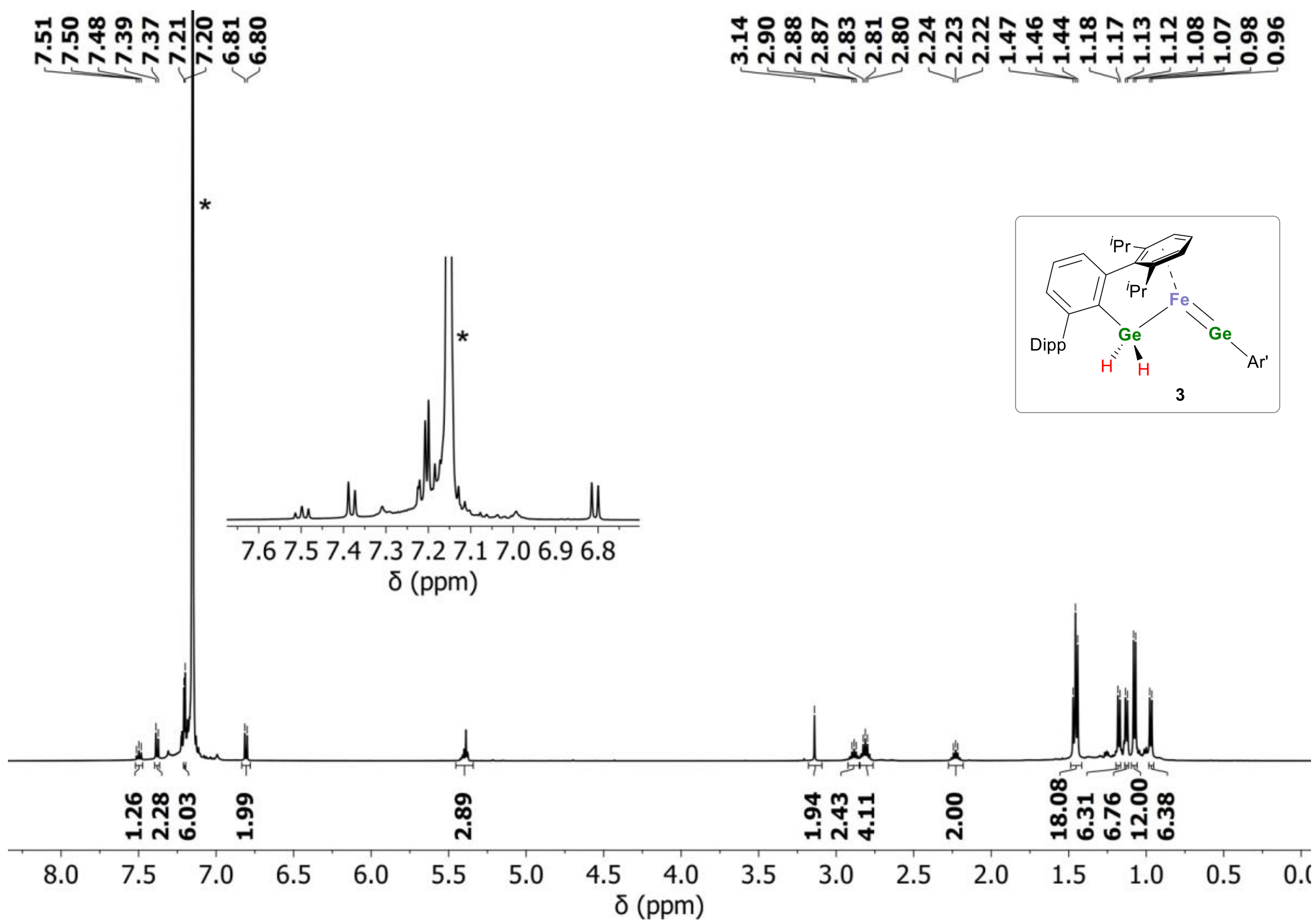

Figure S6. ^1H NMR (500 MHz, 298 K) spectrum of $[(Ar'GeH_2)Fe(GeAr')]$ (**3**) in C_6D_6. *C_6D_5H.

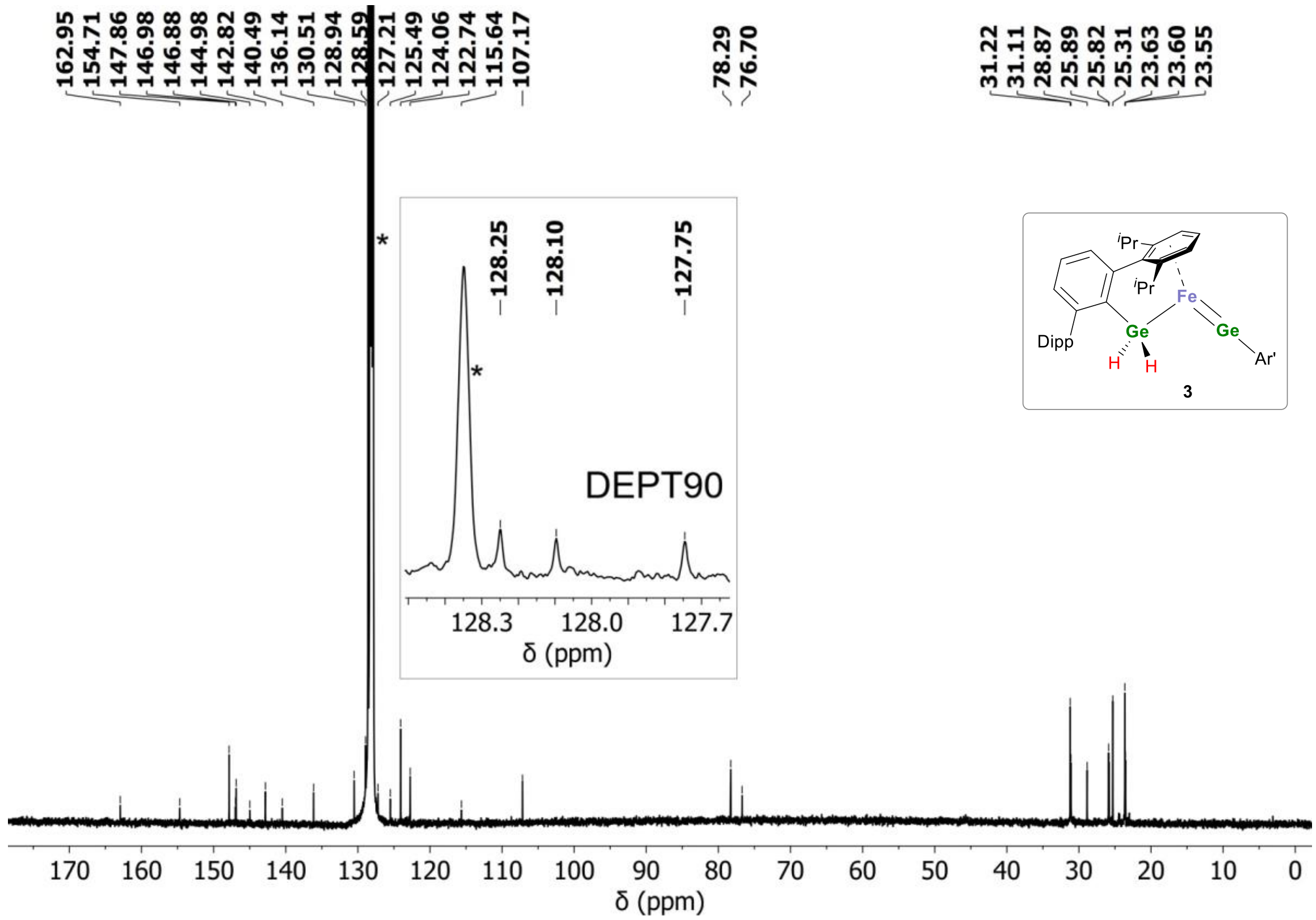

Figure S7. $^{13}C\{^1H\}$ NMR (125 MHz, 298 K) spectrum of [(Ar'GeH$_2$)Fe(GeAr')] (**3**) in C_6D_6. Inset: DEPT90 $^{13}C\{^1H\}$ NMR spectrum of **3** in C_6D_6, showing the signals that overlap with the residual signal of C_6D_6 in the standard $^{13}C\{^1H\}$ NMR spectrum. *C_6D_6.

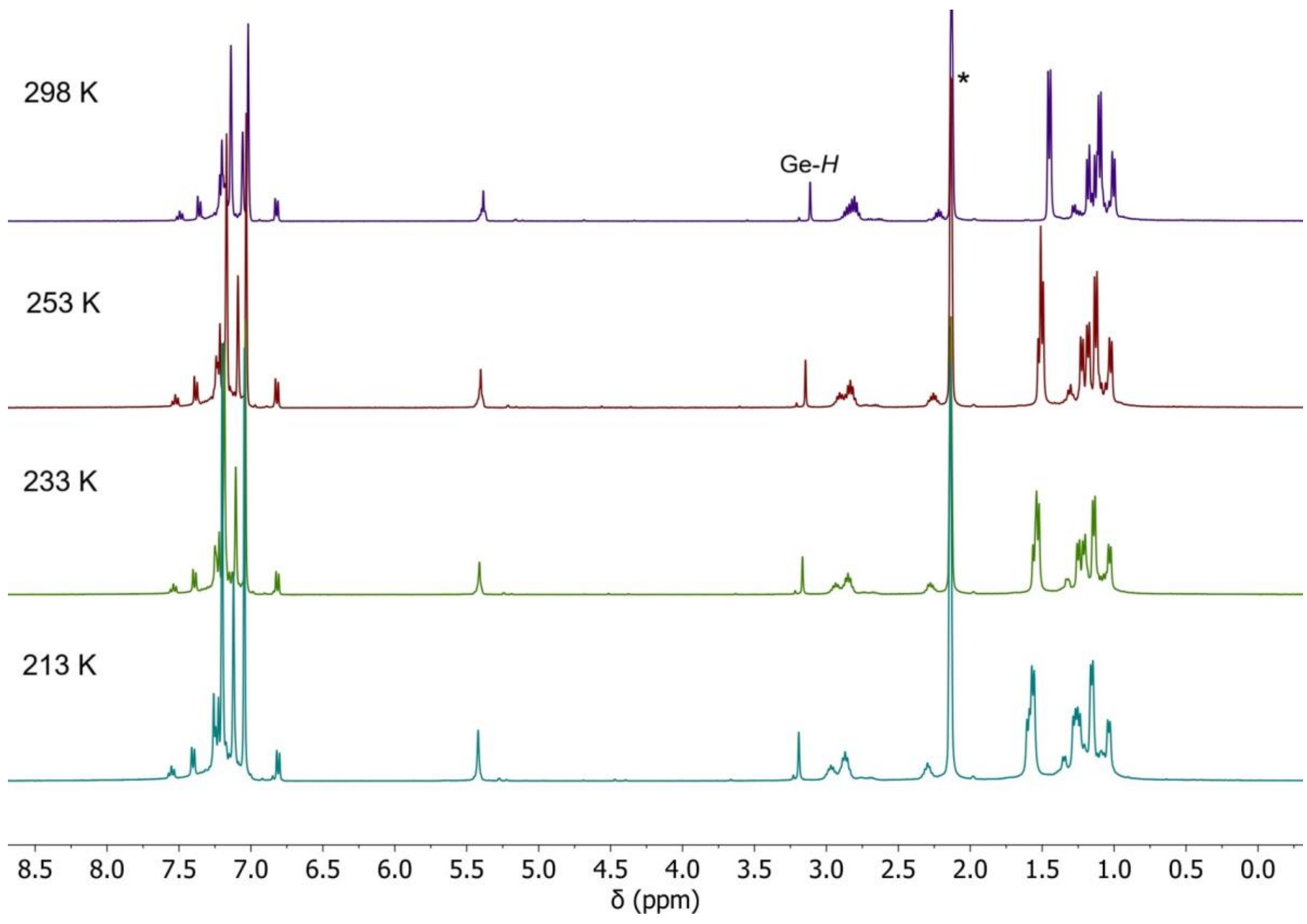

Figure S8. Variable-temperature (VT) ^1H NMR (400 MHz, 213 – 298 K) spectra of [(Ar'GeH$_2$)Fe(GeAr')] (**3**) in d_8-Toluene. *d_8-Toluene.

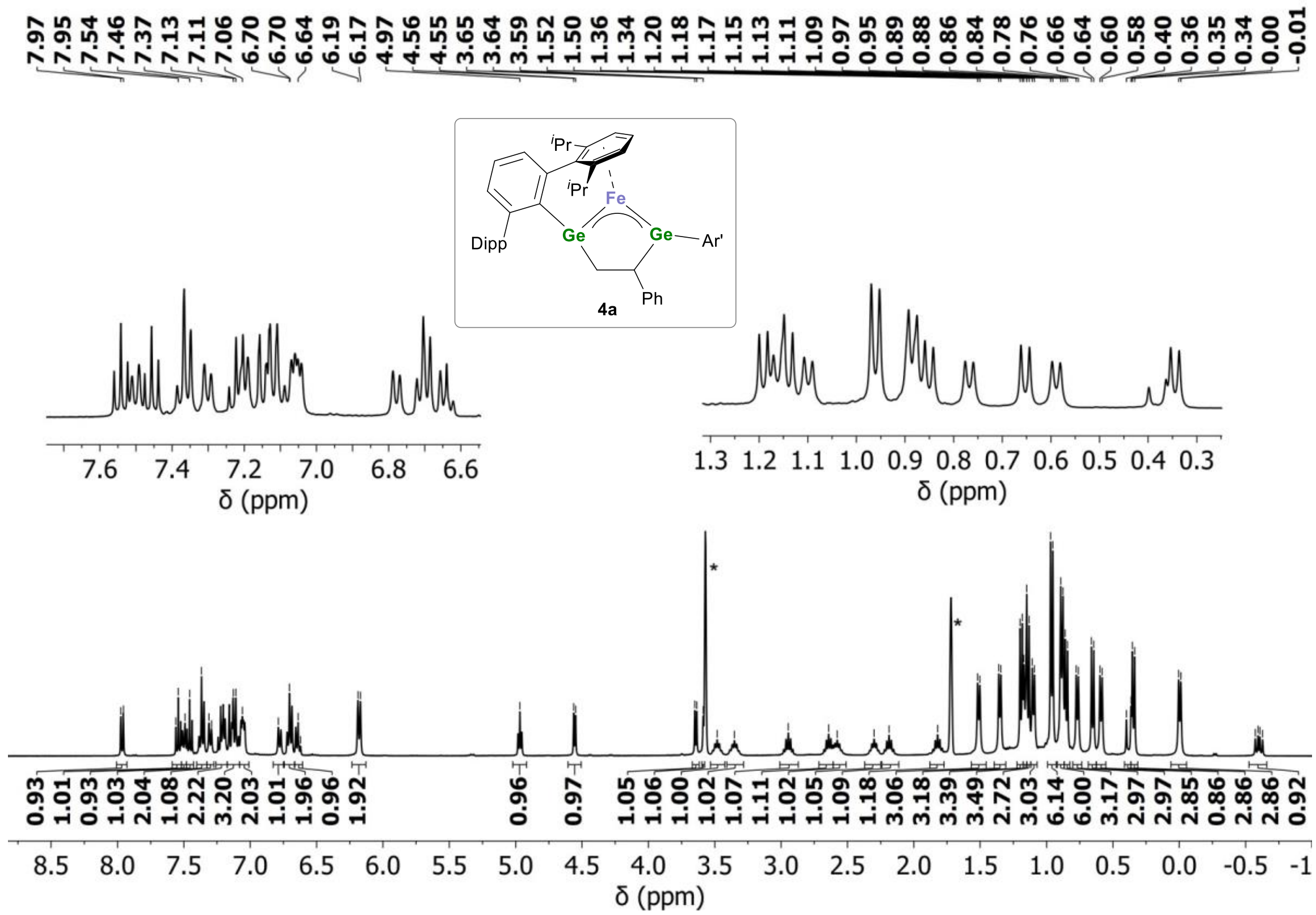

Figure S9. ^{1}H NMR (400 MHz, 268 K) spectrum of [(Ar'GeCH$_2$(Ph)CHGeAr')Fe] (**4a**) in d_8-THF. *d_8-THF.

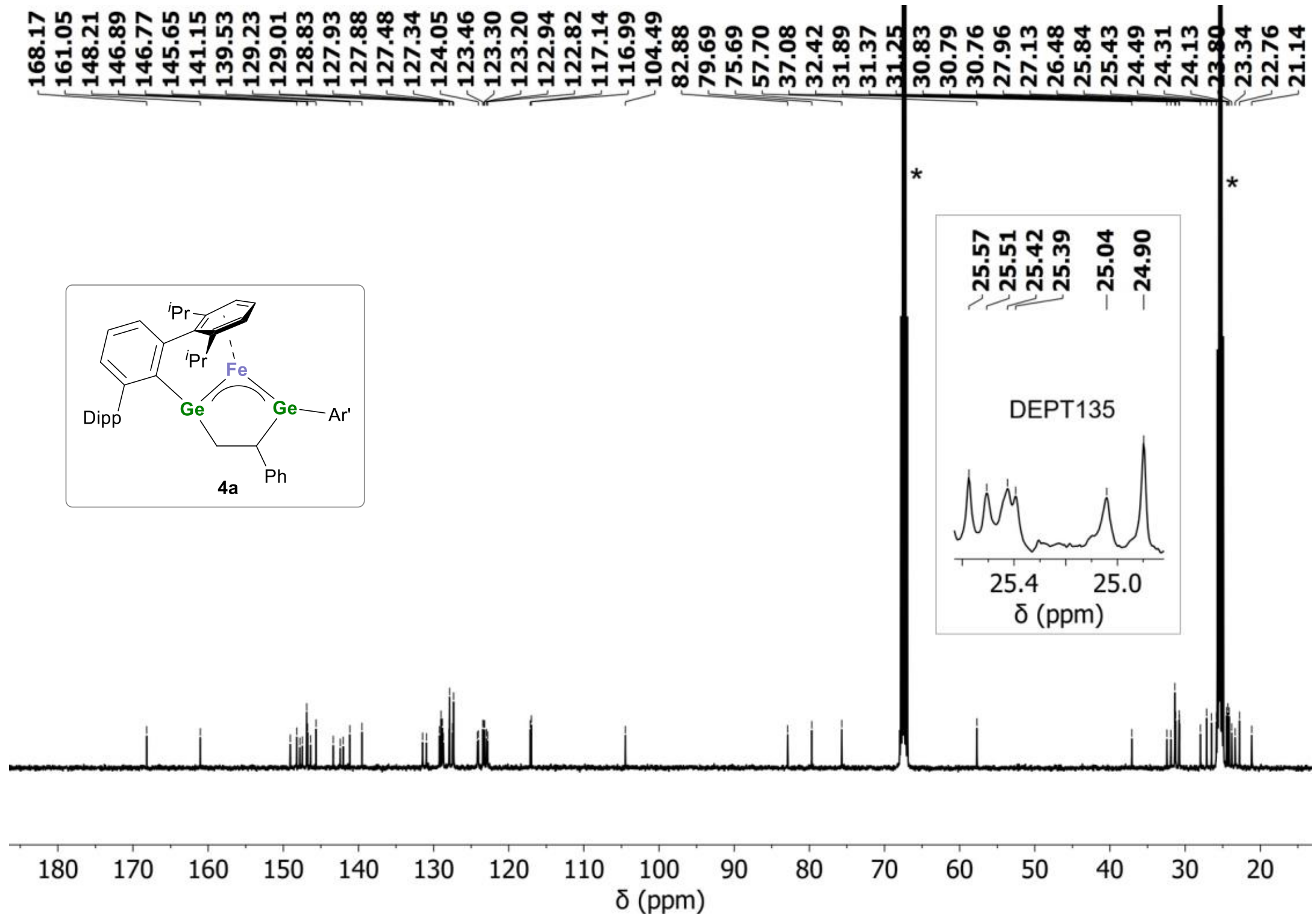

Figure S10. $^{13}C\{^{1}H\}$ NMR (100 MHz, 268 K) spectrum of [(Ar'GeCH$_2$(Ph)CHGeAr')Fe] (**4a**) in d_8-THF. Inset: DEPT135 $^{13}C\{^{1}H\}$ NMR spectrum of **4a** in d_8-THF, showing the signals that overlap with the residual signal of d_8-THF in the standard $^{13}C\{^{1}H\}$ NMR spectrum. *d_8-THF.

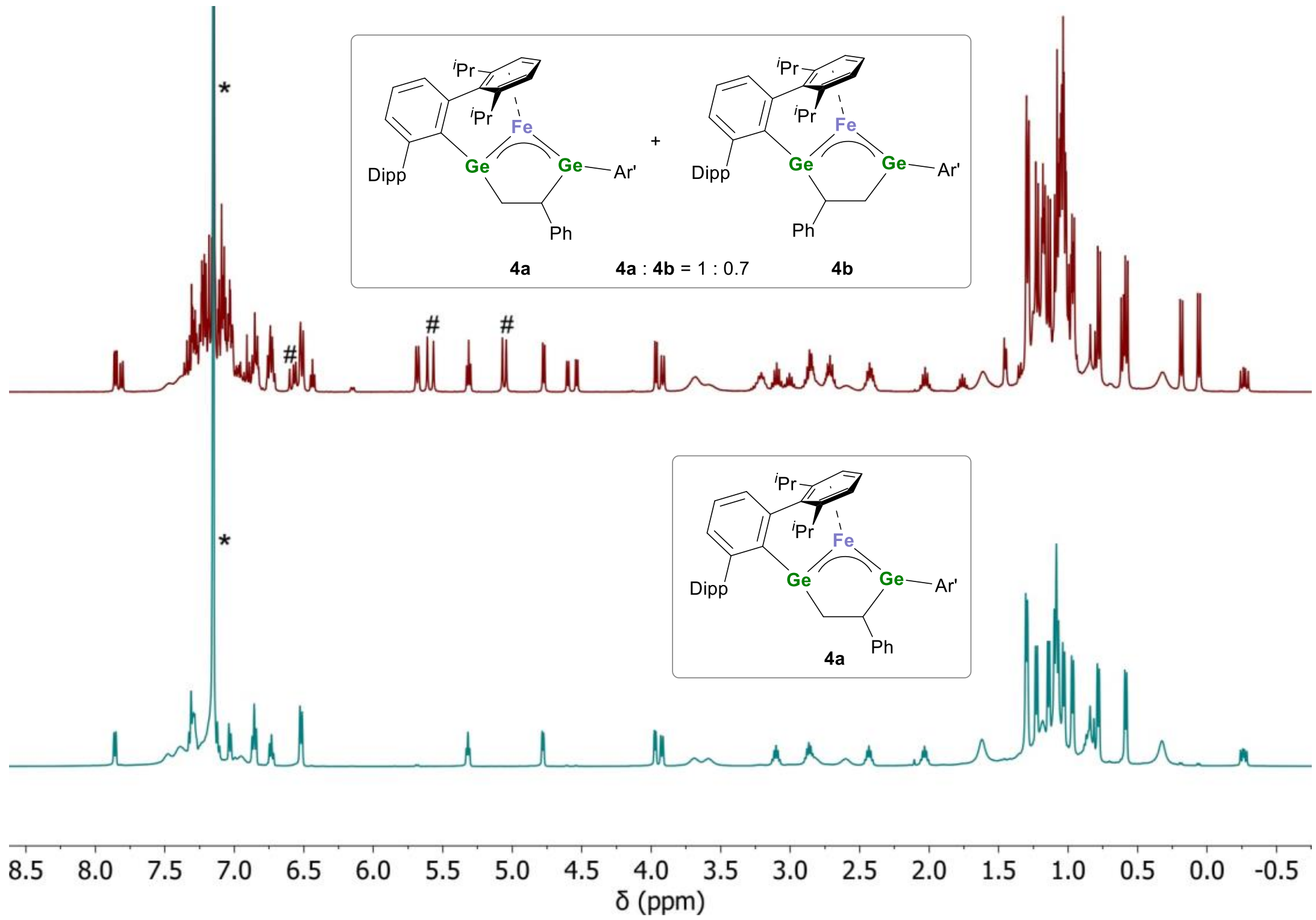

Figure S11. Comparison of ^{1}H NMR (400/500 MHz, 298 K) spectra of [(Ar'GeCH$_2$(Ph)CHGeAr')Fe] (**4a**, bottom) and a mixture of **4a**/**4b** (top) generated from the reaction of **1** with excess styrene in C_6D_6. *C_6D_5H, #Styrene. Note: Broad resonances corresponding to the Ar' substituent not coordinated to the Fe center were observed at room temperature, likely due to slow rotational dynamics on the NMR timescale. At low temperature (268 K), these resonances became well resolved (Figure S9).

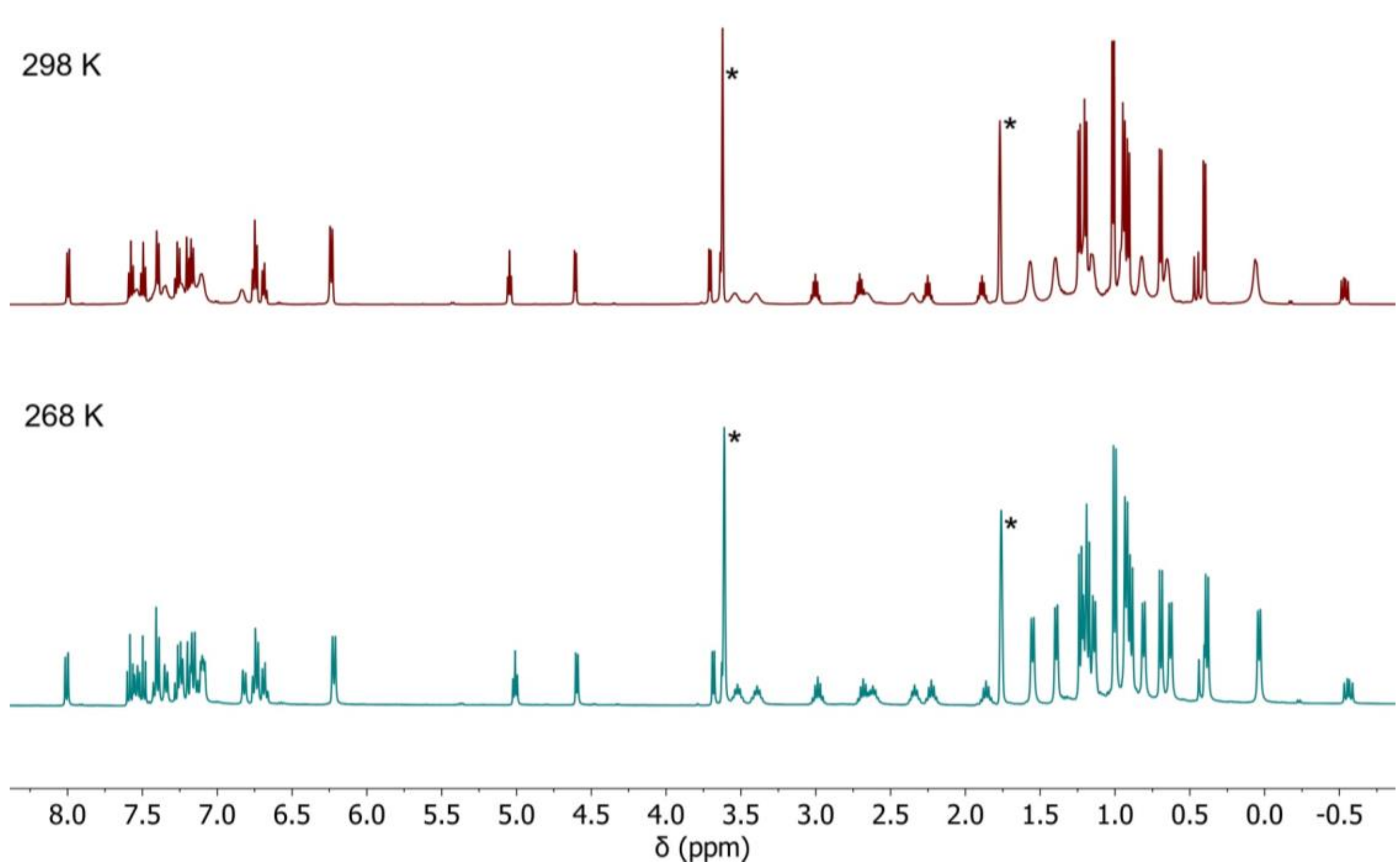

Figure S12. Comparison of ^{1}H NMR (400/500 MHz) spectra of [(Ar'GeCH$_2$(Ph)CHGeAr')Fe] (**4a**) at 298 K (top) and 268 K (bottom) in d_8-THF, showing the dynamic behaviour of **4a** in solution. *d_8-THF.

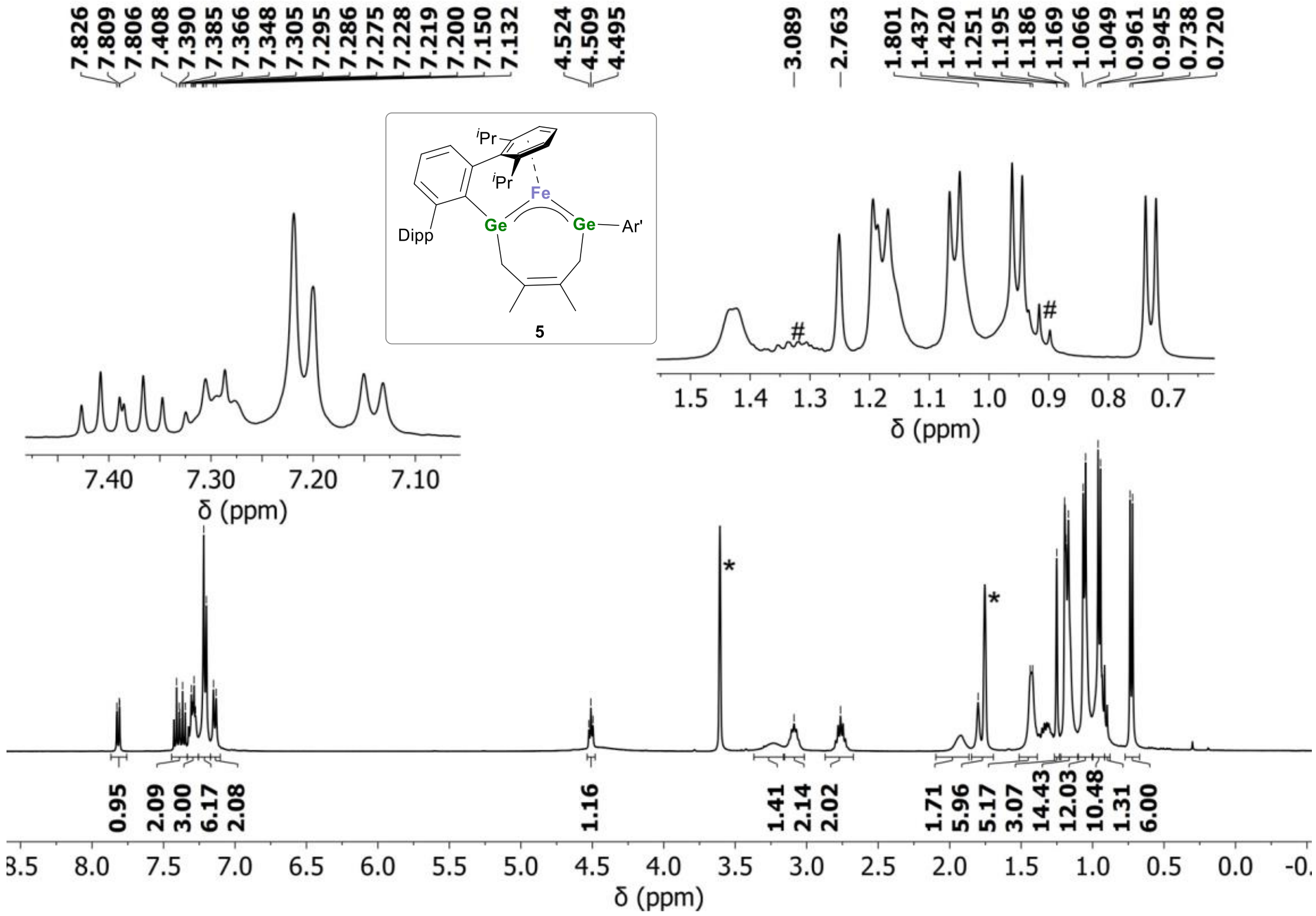

Figure S13. ^{1}H NMR (400 MHz, 298 K) spectrum of [(Ar'GeCH$_2$(CH$_3$)CC(CH$_3$)CH$_2$GeAr')Fe] (**5**) in d_8-THF. *d_8-THF, #*n*-pentane. Broad signals were observed, due to dynamic behavior of **5** in solution. This dynamic process is likely due to the flipping of the FeGe$_2$C$_4$ ring. At low temperature (223 K), these resonances became well resolved (Figures S14 and S15).

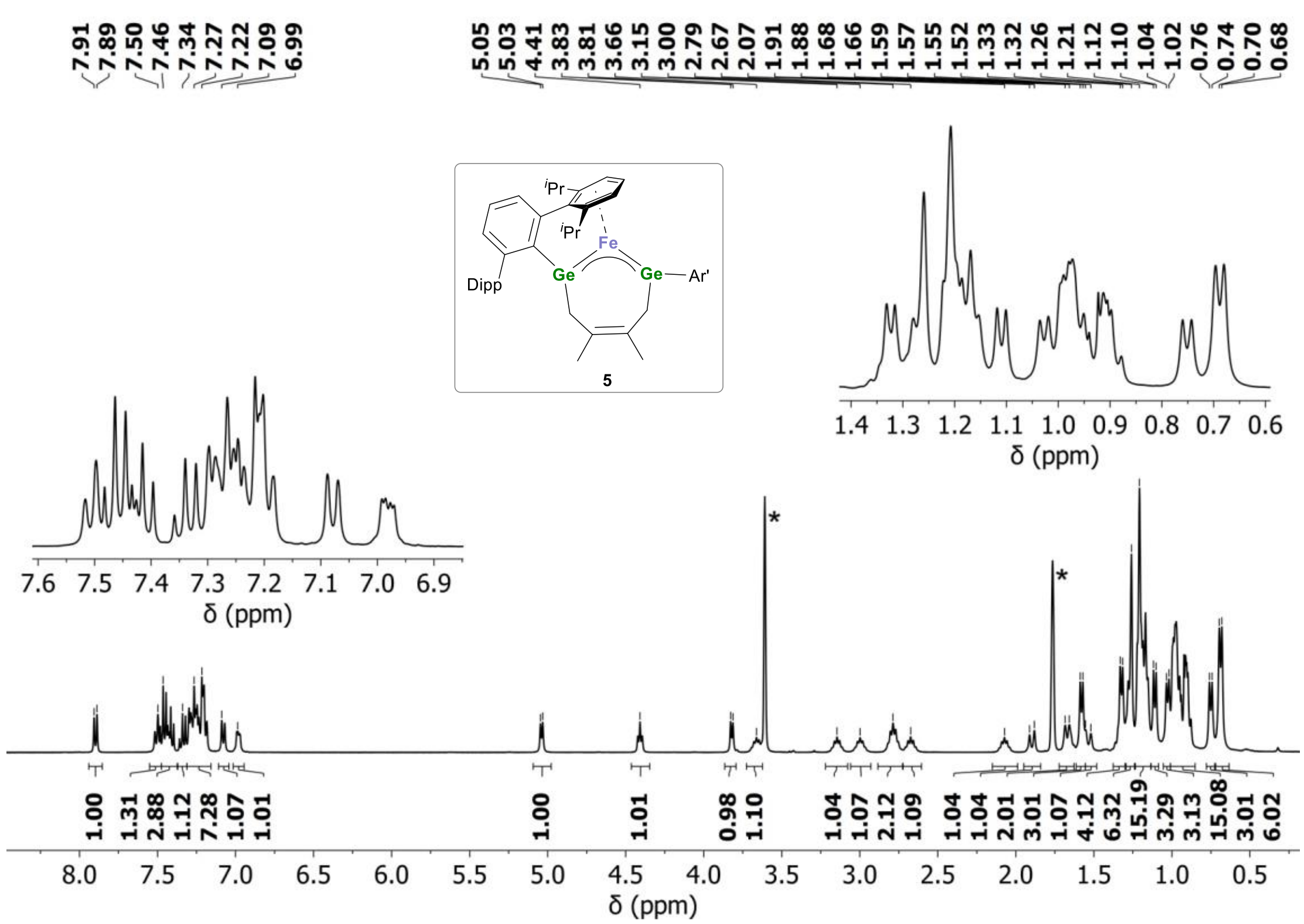

Figure S14. ^{1}H NMR (400 MHz, 223 K) spectrum of [(Ar'GeCH$_2$(CH$_3$)CC(CH$_3$)CH$_2$GeAr')Fe] (**5**) in d_8-THF. *d_8-THF.

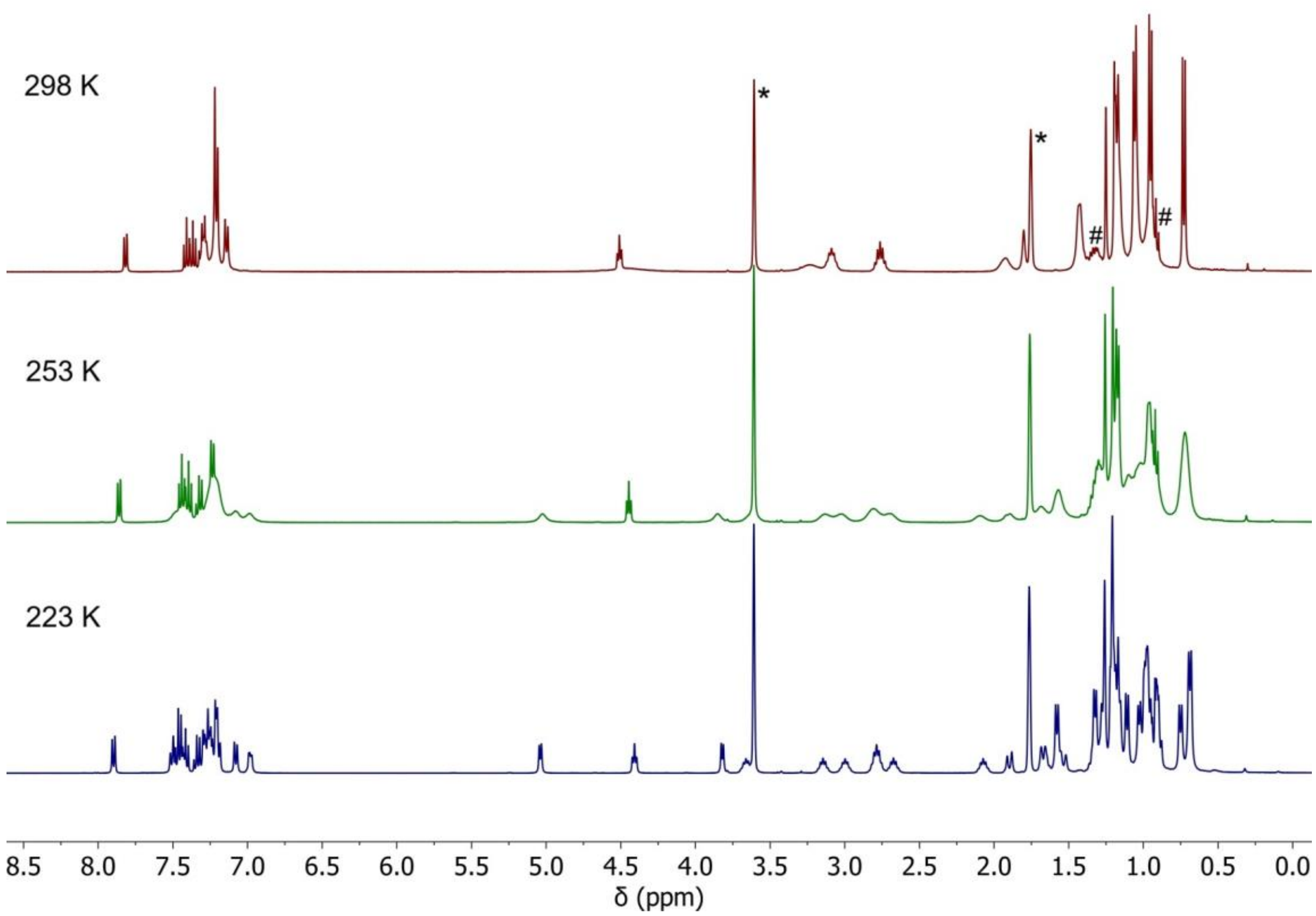

Figure S15. Variable-temperature (VT) ^{1}H NMR (400 MHz) spectra of [(Ar'GeCH$_2$(CH$_3$)CC(CH$_3$)CH$_2$GeAr')Fe] (**5**) in d_8-THF. *d_8-THF, #*n*-pentane.

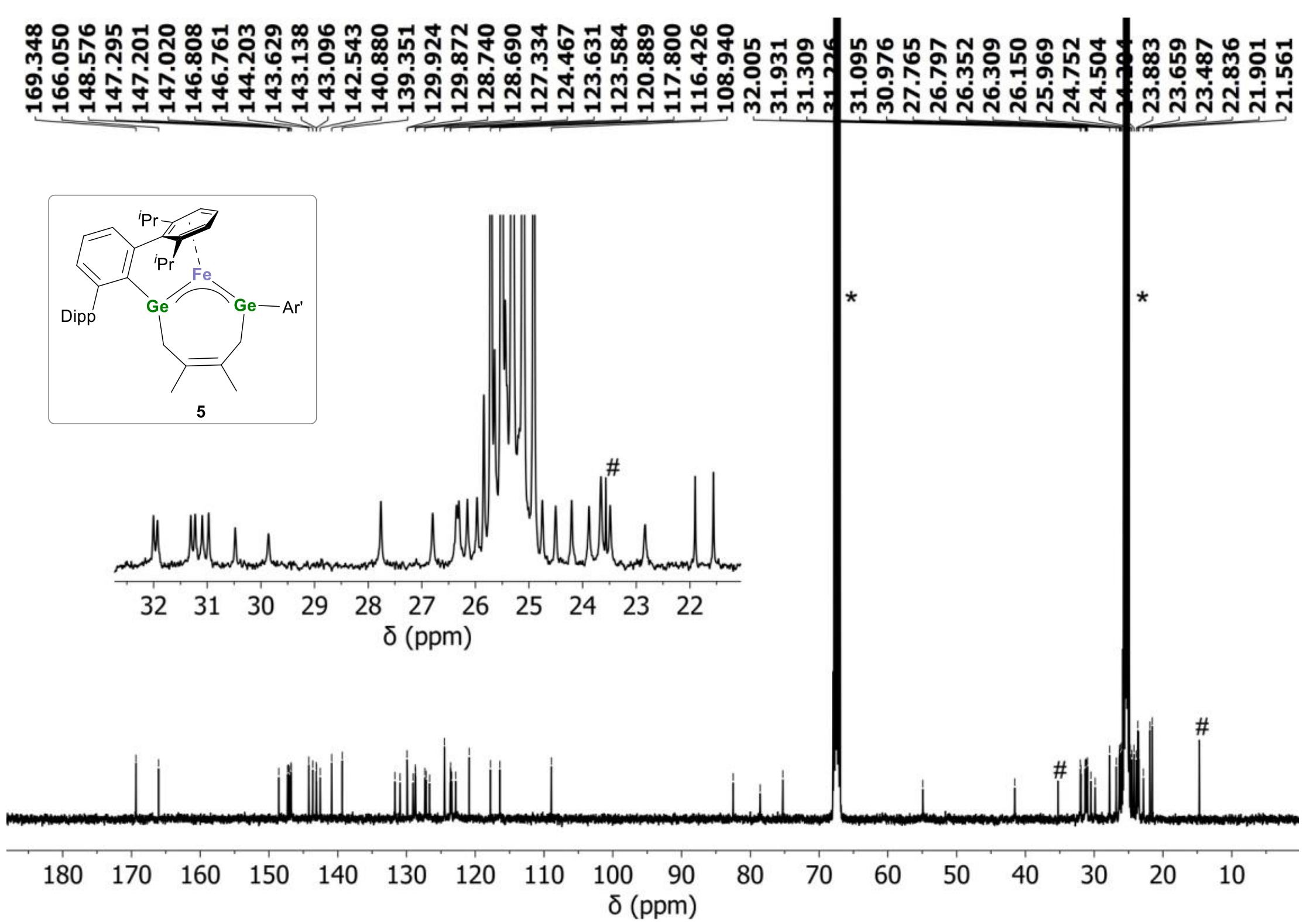

Figure S16. ^{13}C{^{1}H} NMR (100 MHz, 223 K) spectrum of [(Ar'GeCH$_2$(CH$_3$)CC(CH$_3$)CH$_2$GeAr')Fe] (**5**) in d_8-THF. *d_8-THF, #*n*-pentane.

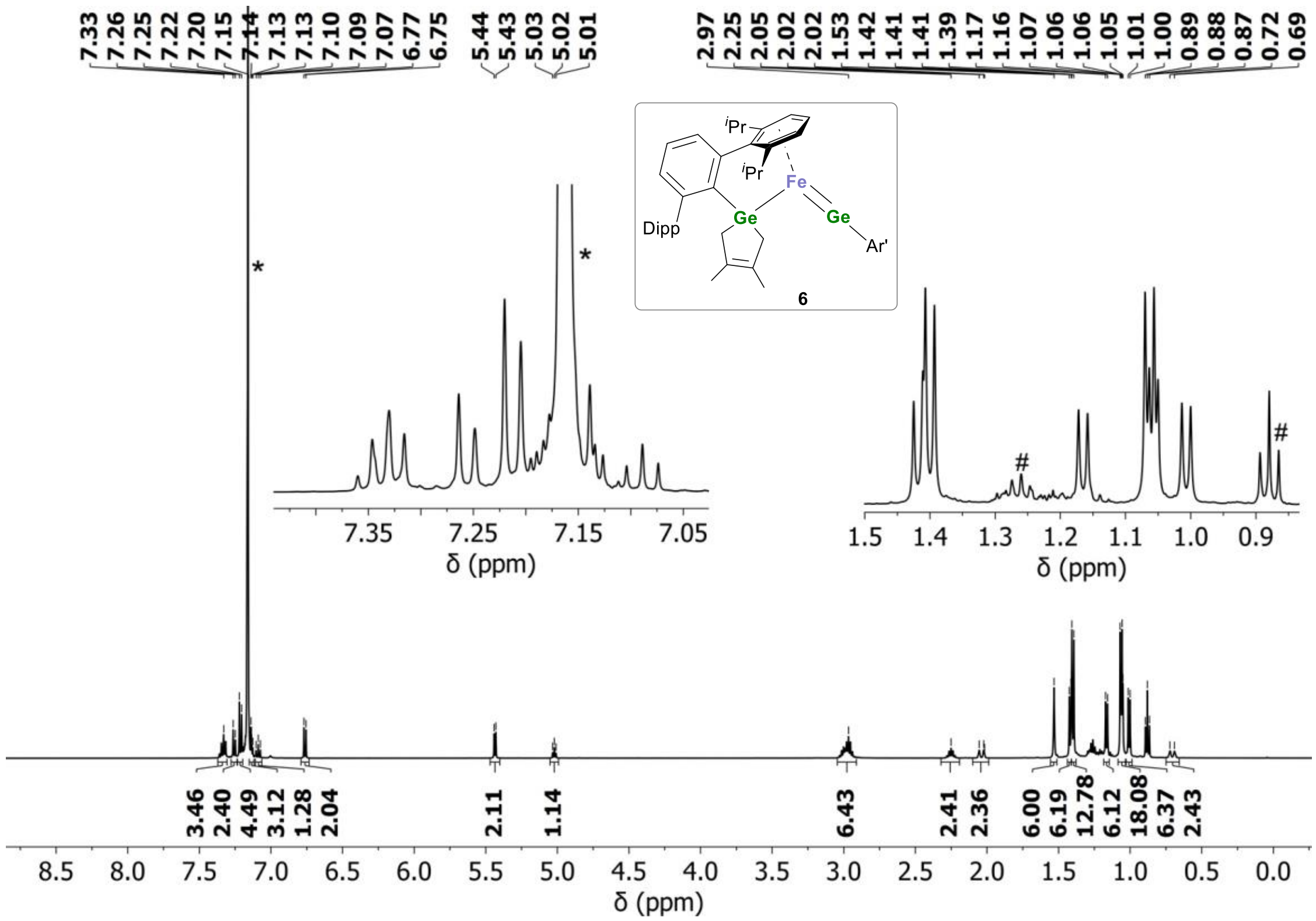

Figure S17. ^{1}H NMR (500 MHz, 298 K) spectrum of [(Ar’(CH$_2$(CH$_3$)CC(CH$_3$)CH$_2$)GeFe(GeAr’)] (**6**) in C_6D_6. *C_6D_5H, #*n*-pentane.

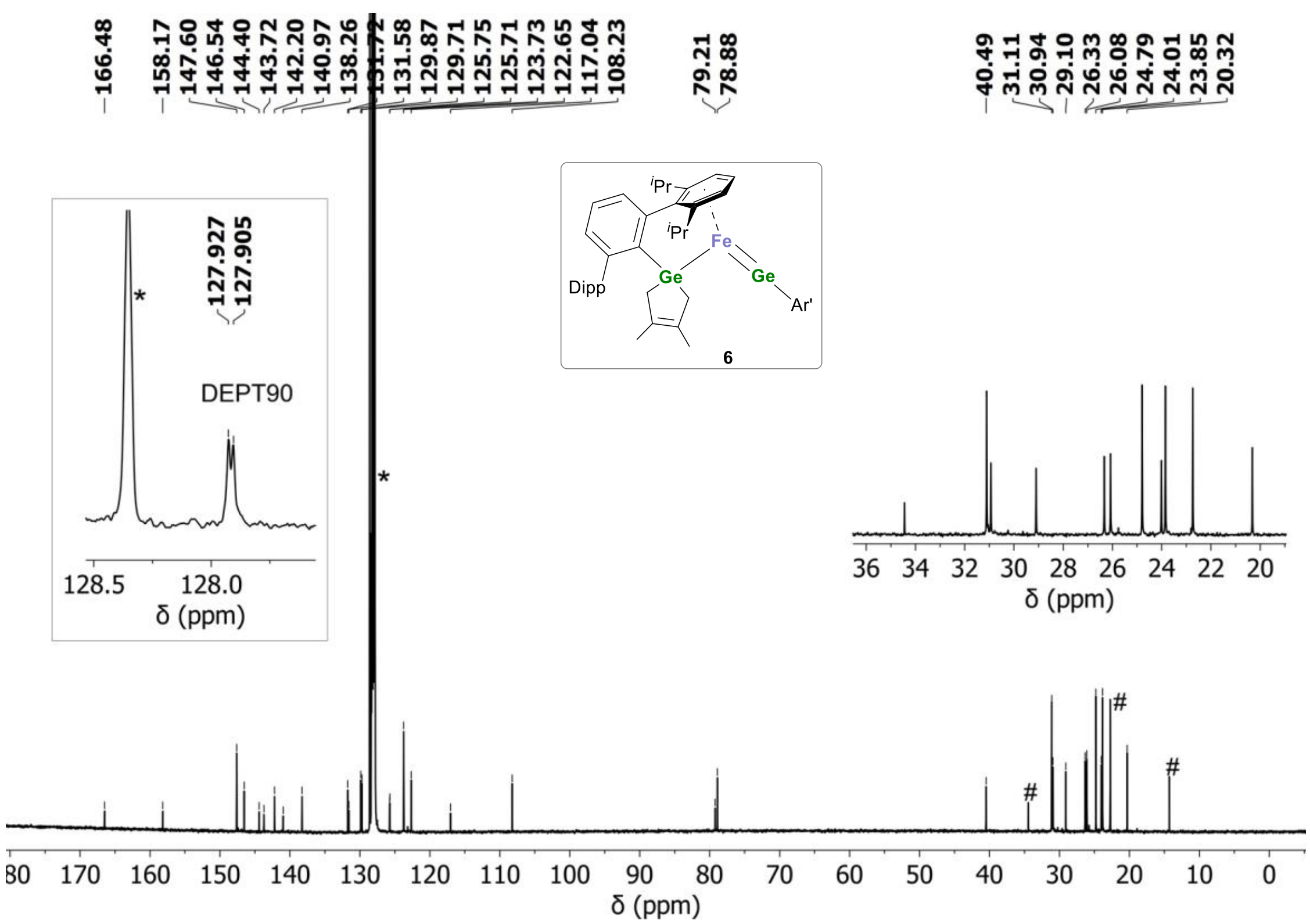

Figure S18. $^{13}C\{^{1}H\}$ NMR (125 MHz, 298 K) spectrum of [(Ar’(CH$_2$(CH$_3$)CC(CH$_3$)CH$_2$)GeFe(GeAr’)] (**6**) in C_6D_6. Inset: DEPT90 $^{13}C\{^{1}H\}$ NMR spectrum of **6** in C_6D_6, showing the signals that overlap with the residual signal of C_6D_6 in the standard $^{13}C\{^{1}H\}$ NMR spectrum. *C_6D_6, #*n*-pentane.

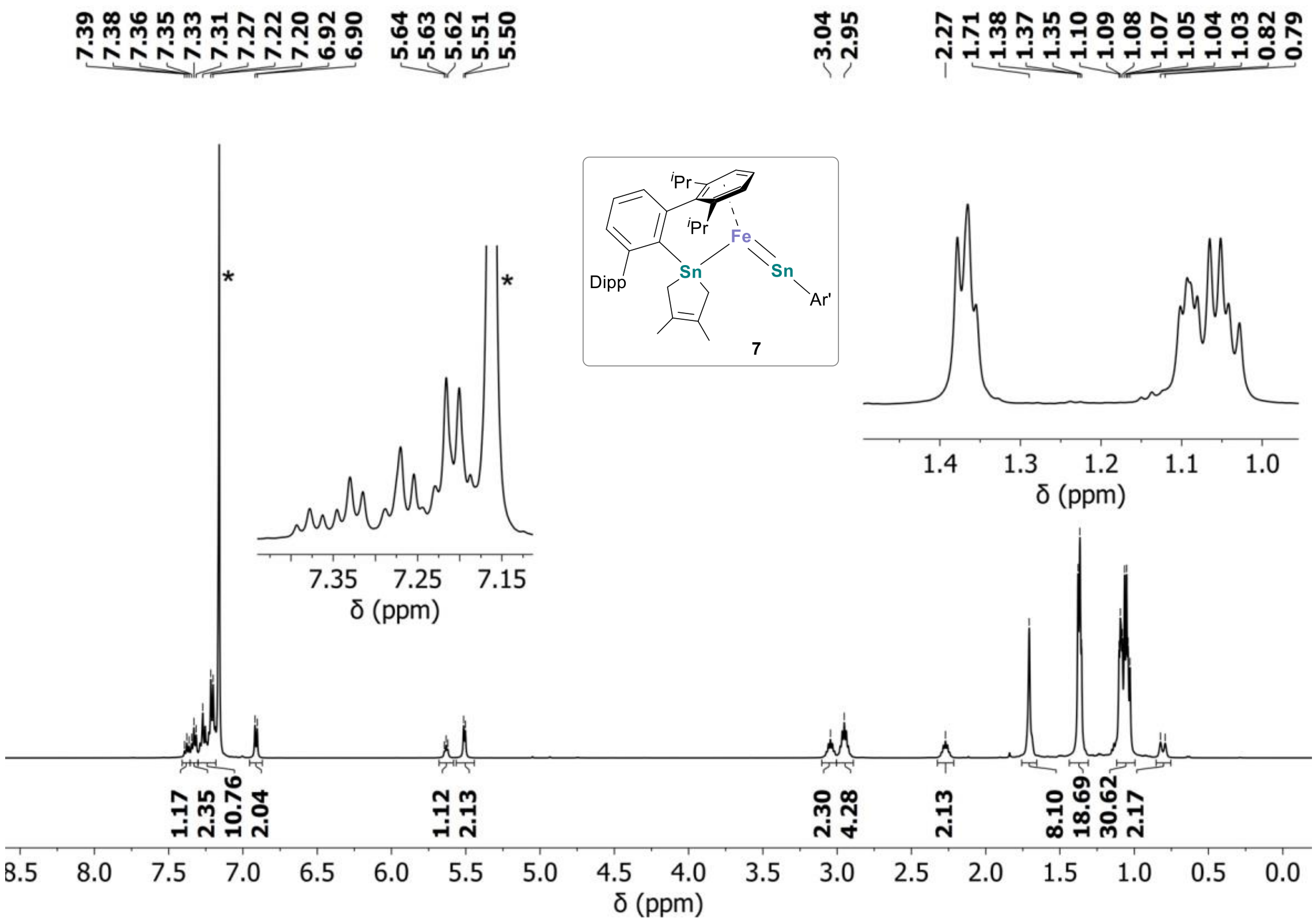

Figure S19. ^{1}H NMR (500 MHz, 298 K) spectrum of [(Ar'($CH_2(CH_3)CC(CH_3)CH_2$)SnFe(SnAr')] (**7**) in C_6D_6. *C_6D_5H.

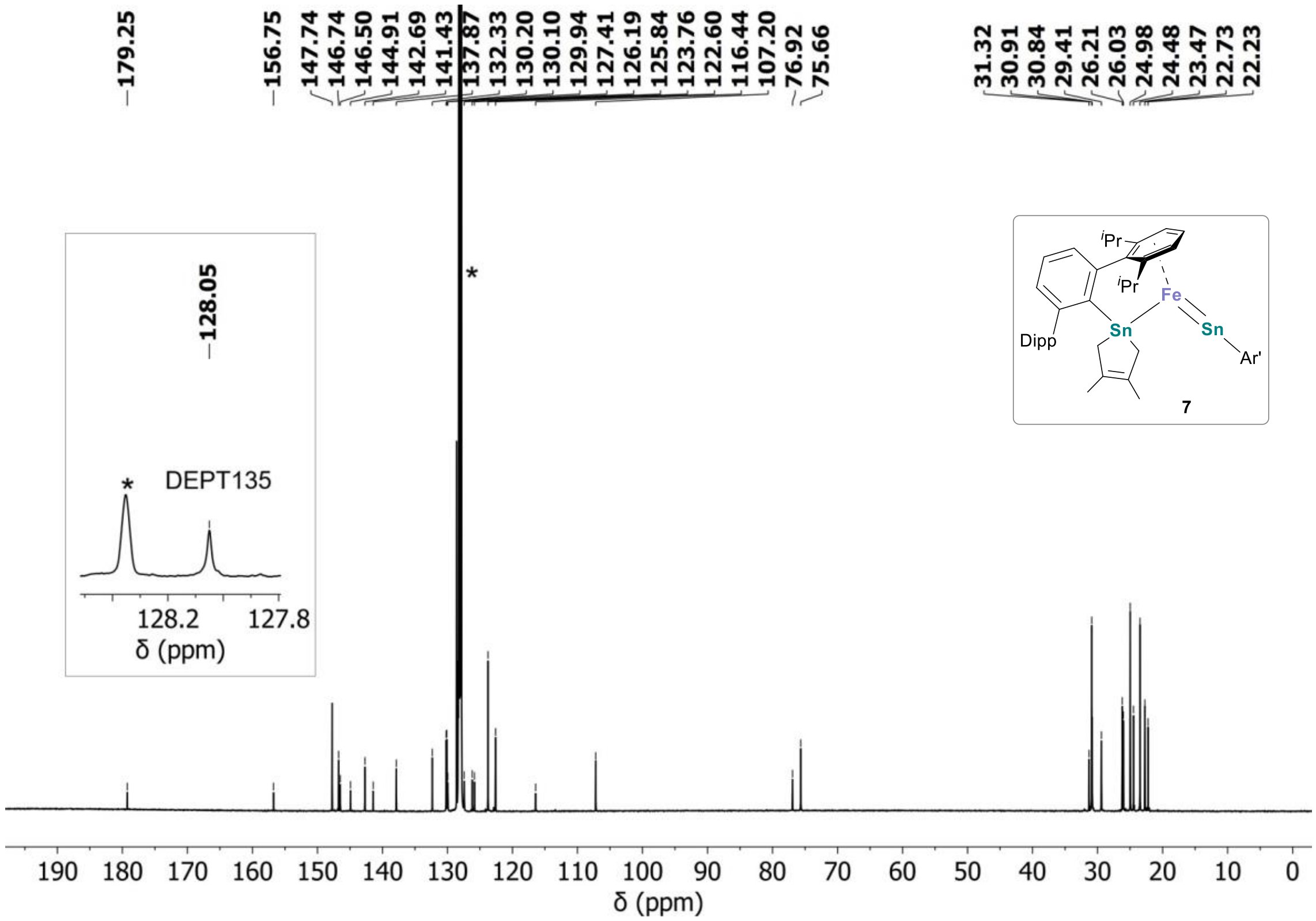

Figure S20. $^{13}C\{^{1}H\}$ NMR (125 MHz, 298 K) spectrum of [(Ar'($CH_2(CH_3)CC(CH_3)CH_2$)SnFe(SnAr')] (**7**) in C_6D_6. Inset: DEPT135 $^{13}C\{^{1}H\}$ NMR spectrum of **7** in C_6D_6, showing the signals that overlap with the residual signal of C_6D_6 in the standard $^{13}C\{^{1}H\}$ NMR spectrum. *C_6D_6.

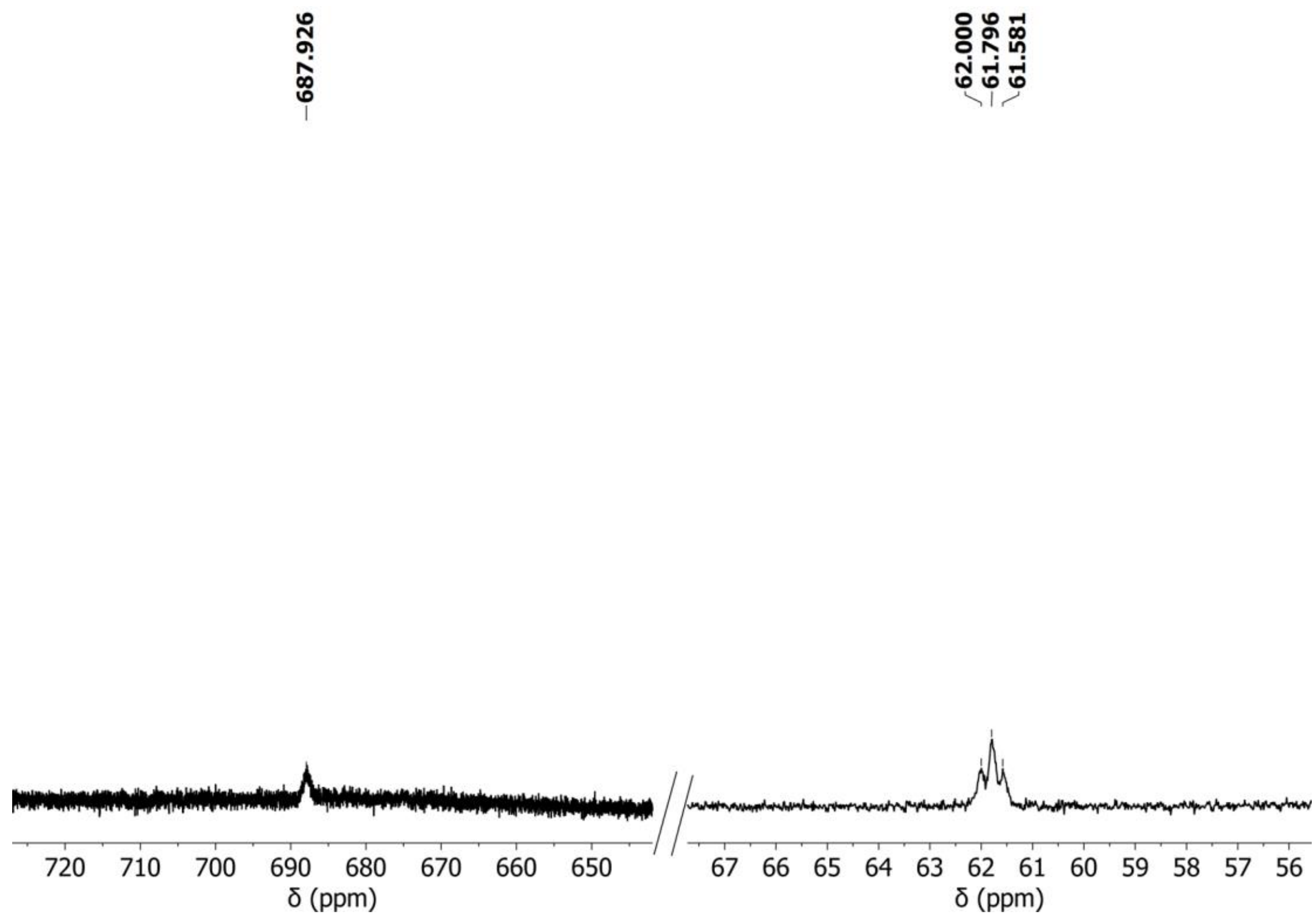

Figure S21. $^{119}Sn\{^1H\}$ NMR (186 MHz, 298 K) spectrum of [(Ar'($CH_2(CH_3)CC(CH_3)CH_2$)SnFe(SnAr')] (**7**) in C_6D_6.

UV-vis Spectra

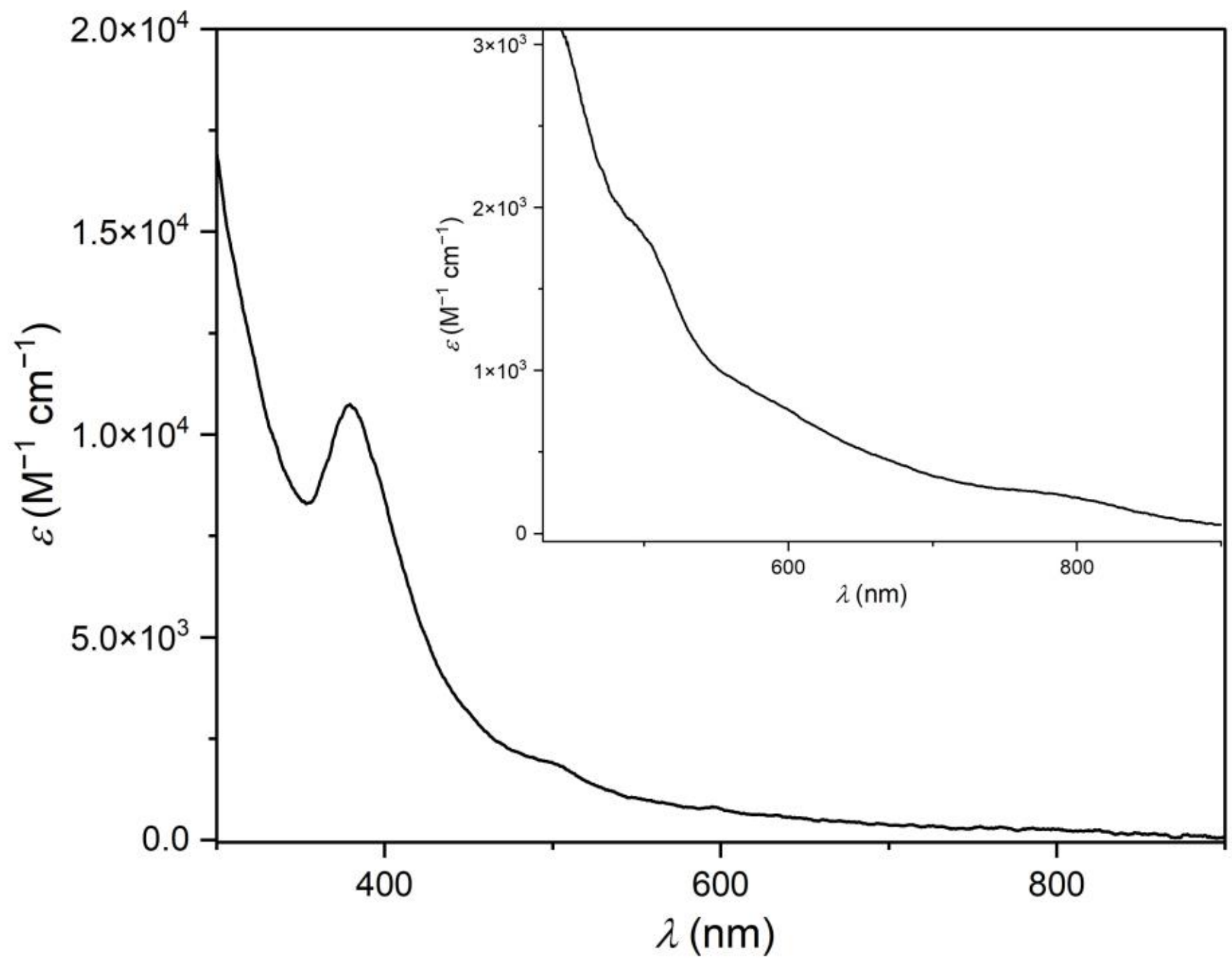

Figure S22. UV-vis spectra of **1** recorded in toluene (*ca.* 0.06 mM, inset: *ca*. 0.6 mM) at 25 °C.

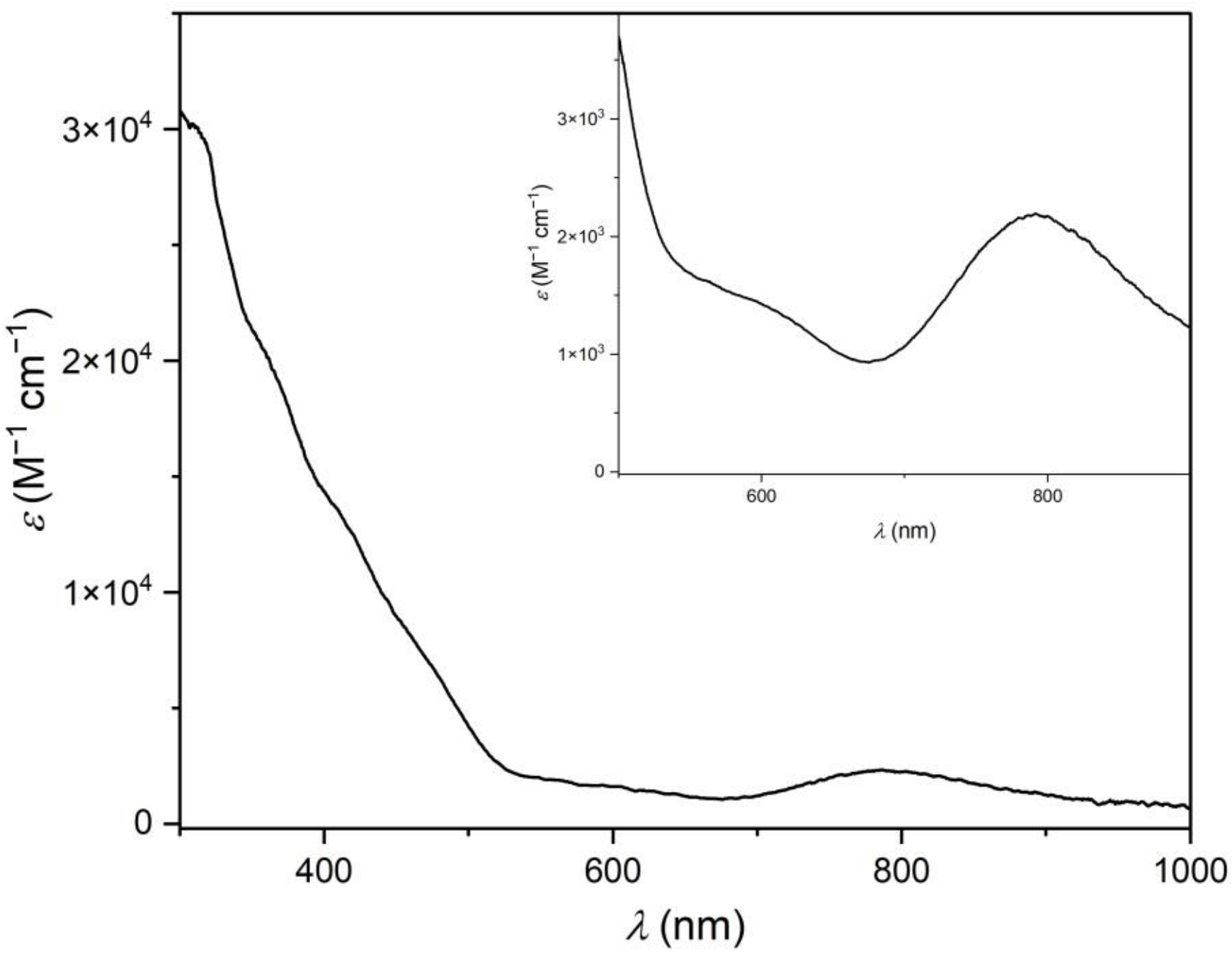

Figure S23. UV-vis spectra of **2** recorded in toluene (*ca.* 0.05 mM, inset: *ca*. 0.5 mM) at 25 °C.

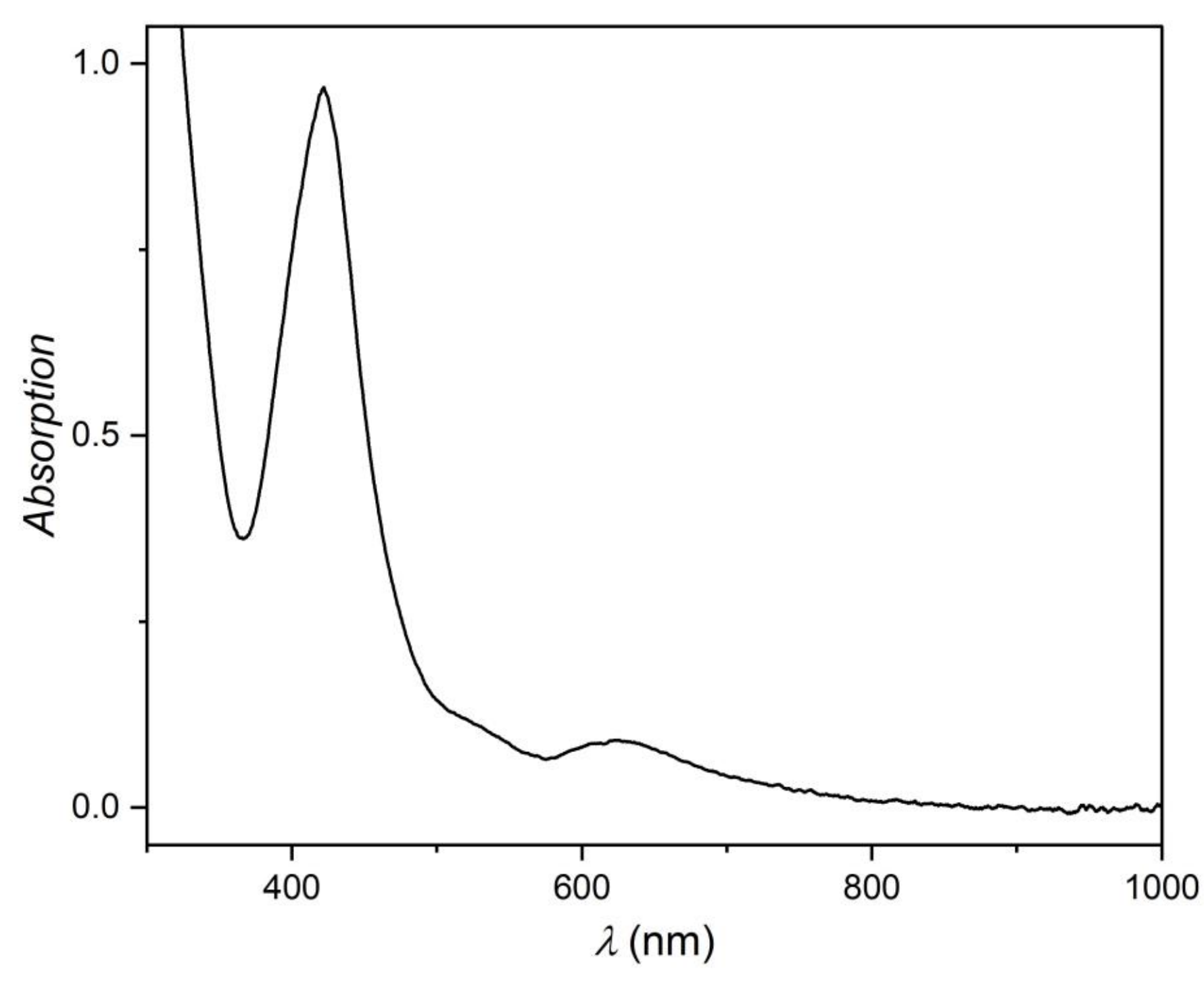

Figure S24. UV-vis spectrum of **3** recorded in toluene at 25 °C. The poor solubility of **3** in toluene prevented accurate determination of its concentration; therefore, the absorption coefficient was not calculated.

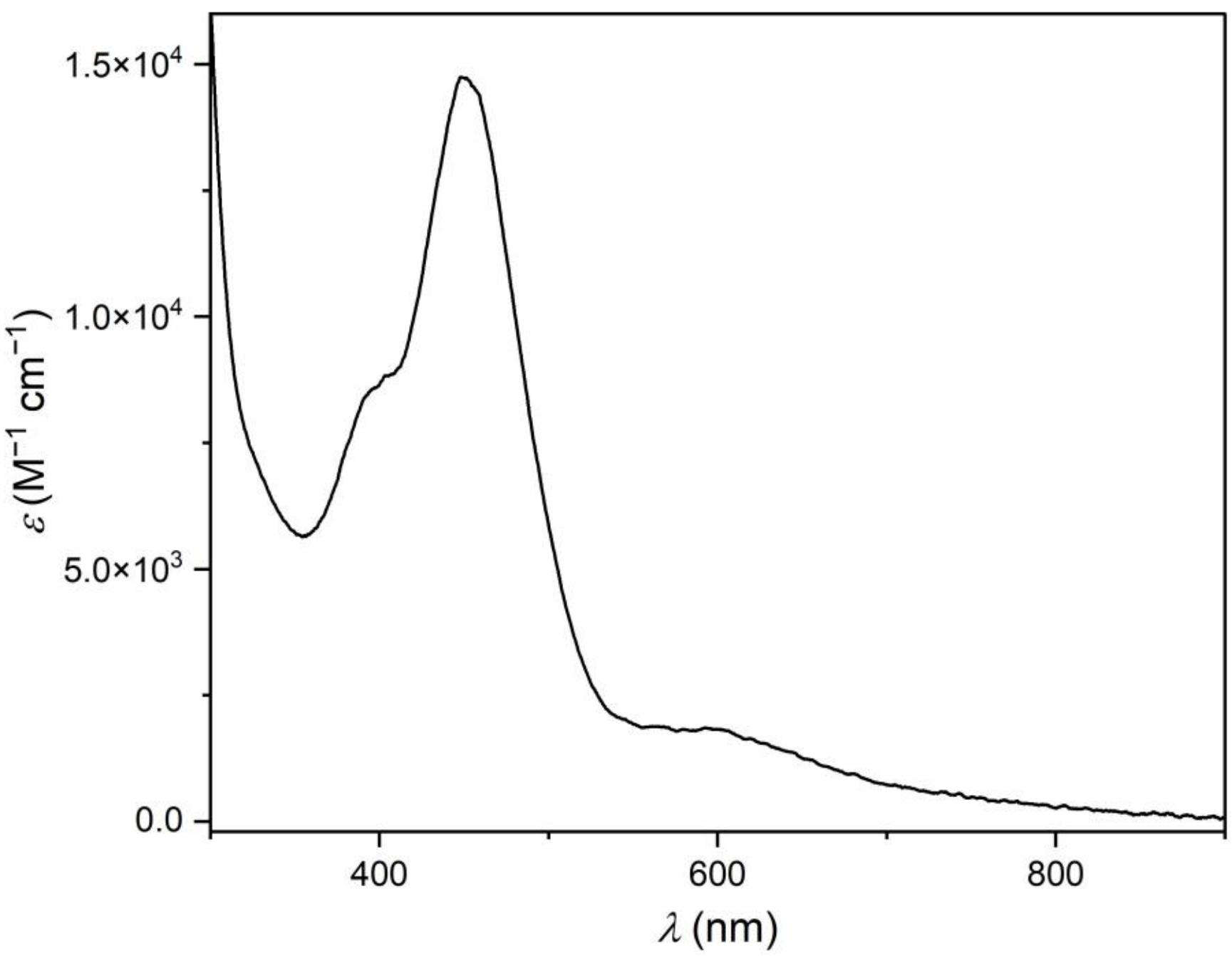

Figure S25. UV-vis spectrum of **4a** recorded in toluene (*ca.* 0.06 mM) at 25 °C.

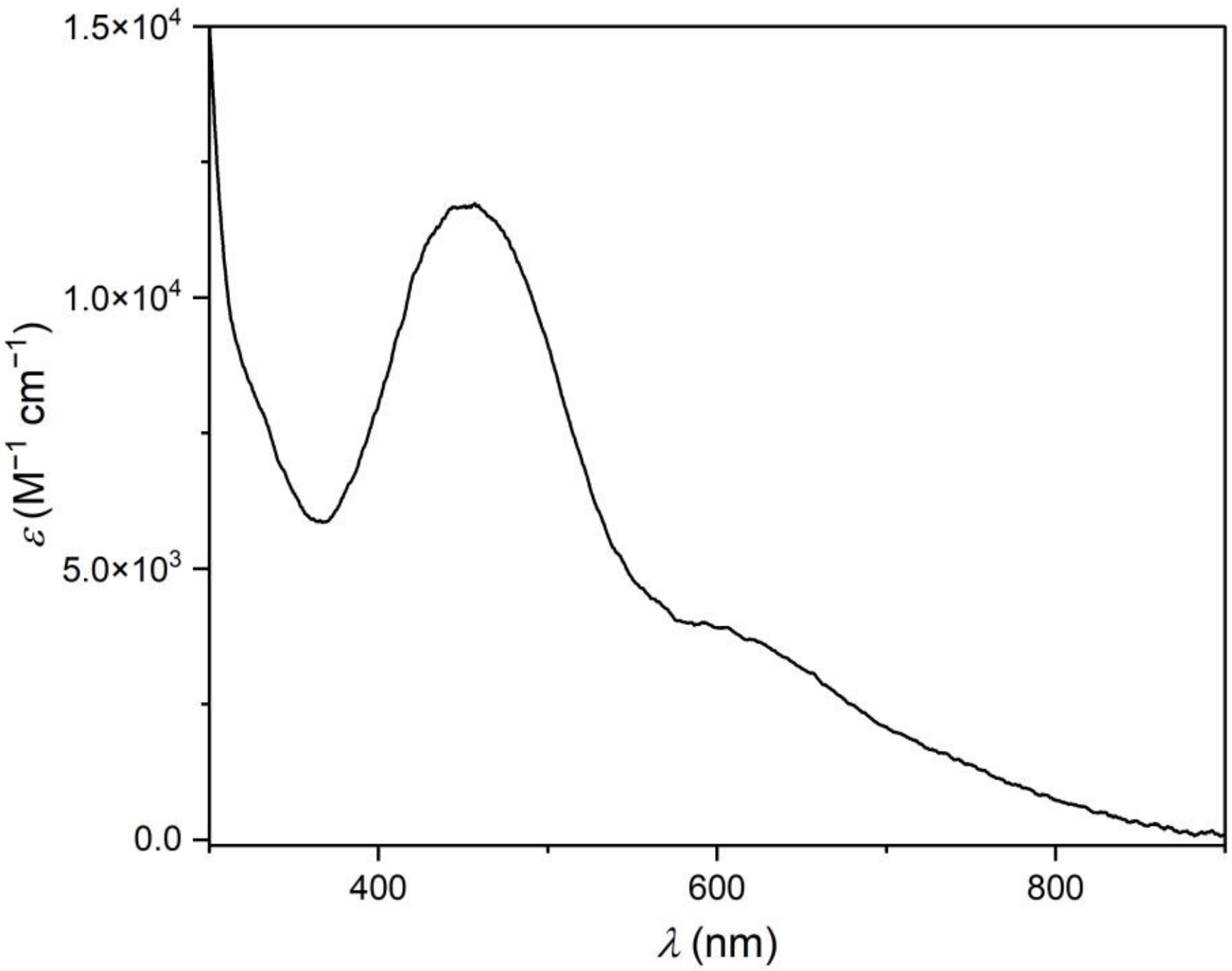

Figure S26. UV-vis spectrum of **5** recorded in toluene (*ca.* 0.06 mM) at 25 °C.

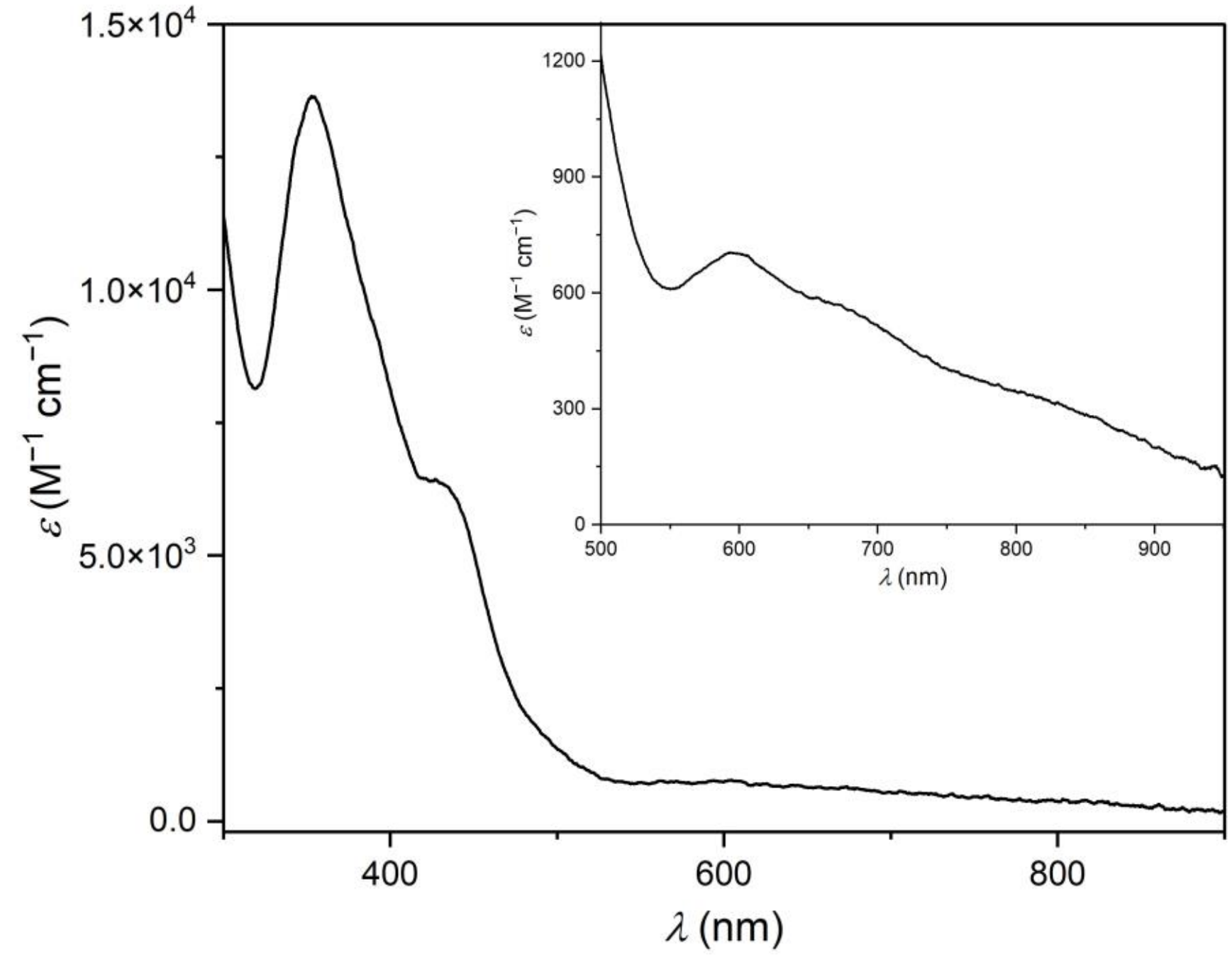

Figure S27. UV-vis spectra of **6** recorded in toluene (*ca.* 0.06 mM, inset: *ca*. 0.6 mM) at 25 °C.

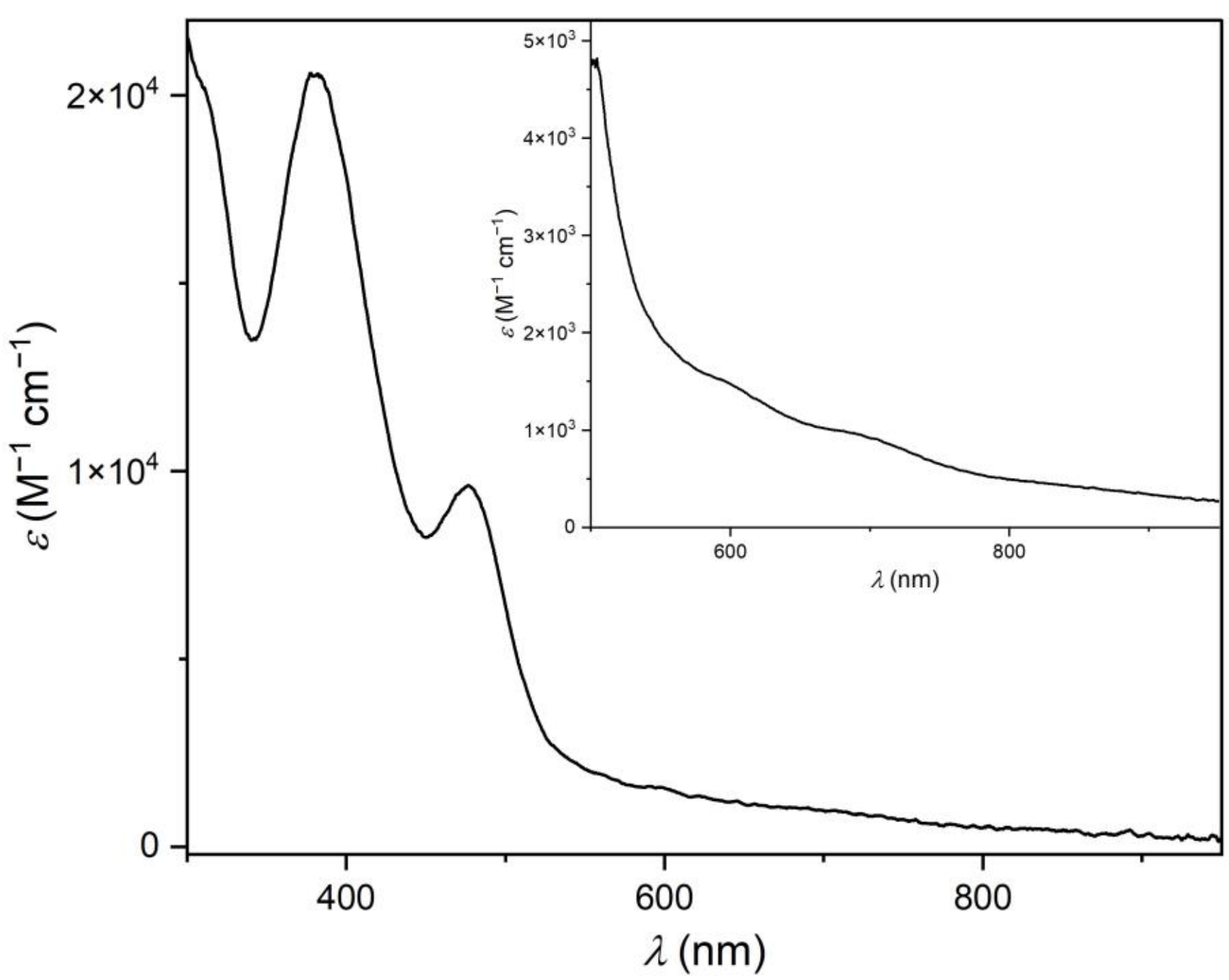

Figure S28. UV-vis spectra of **7** recorded in toluene (*ca.* 0.05 mM, inset: *ca*. 0.5 mM) at 25 °C.

IR Spectra

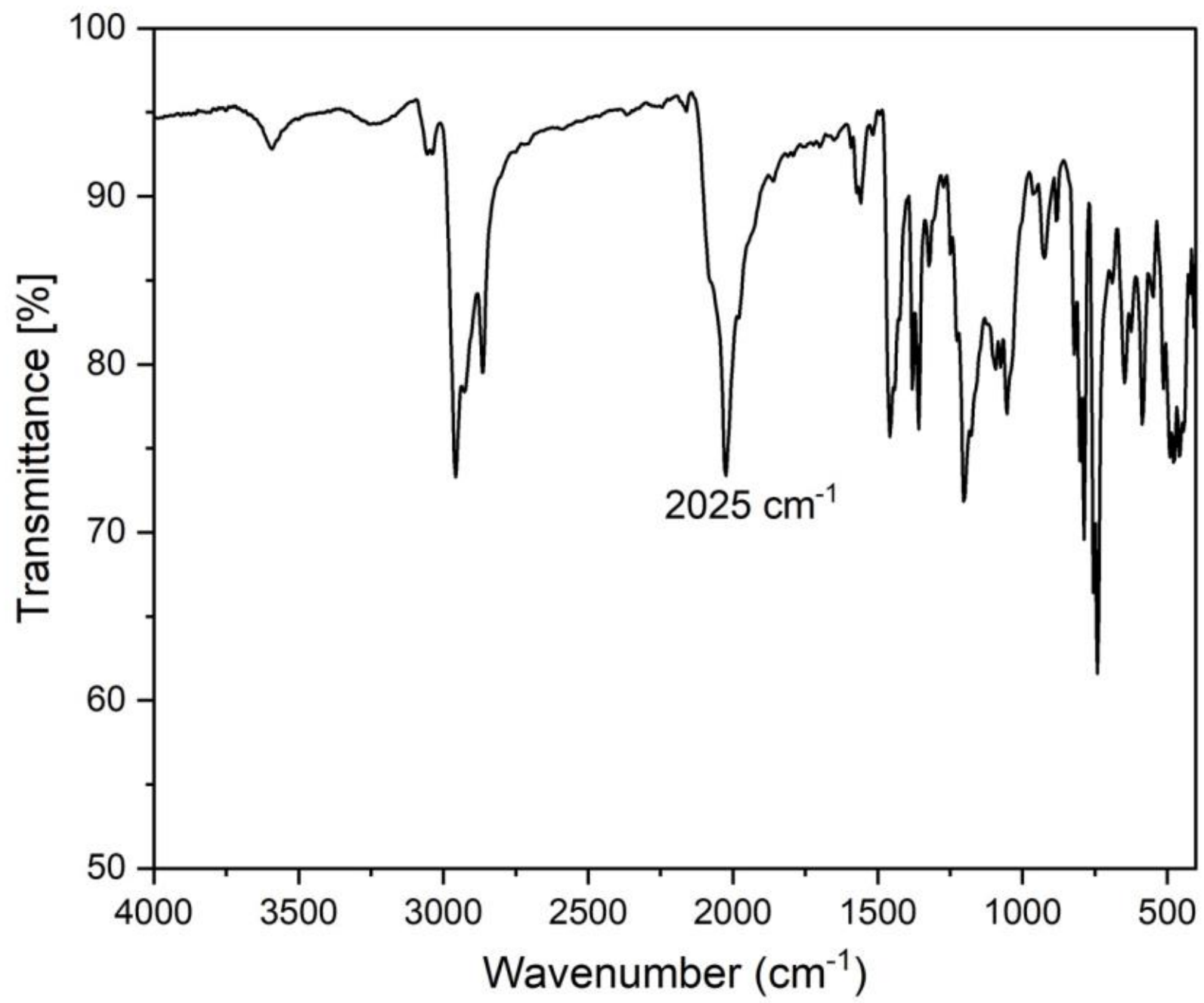

Figure S29. ATR−IR spectrum (solid) of **2** (ν_{NC} = 2025 cm^{-1}).

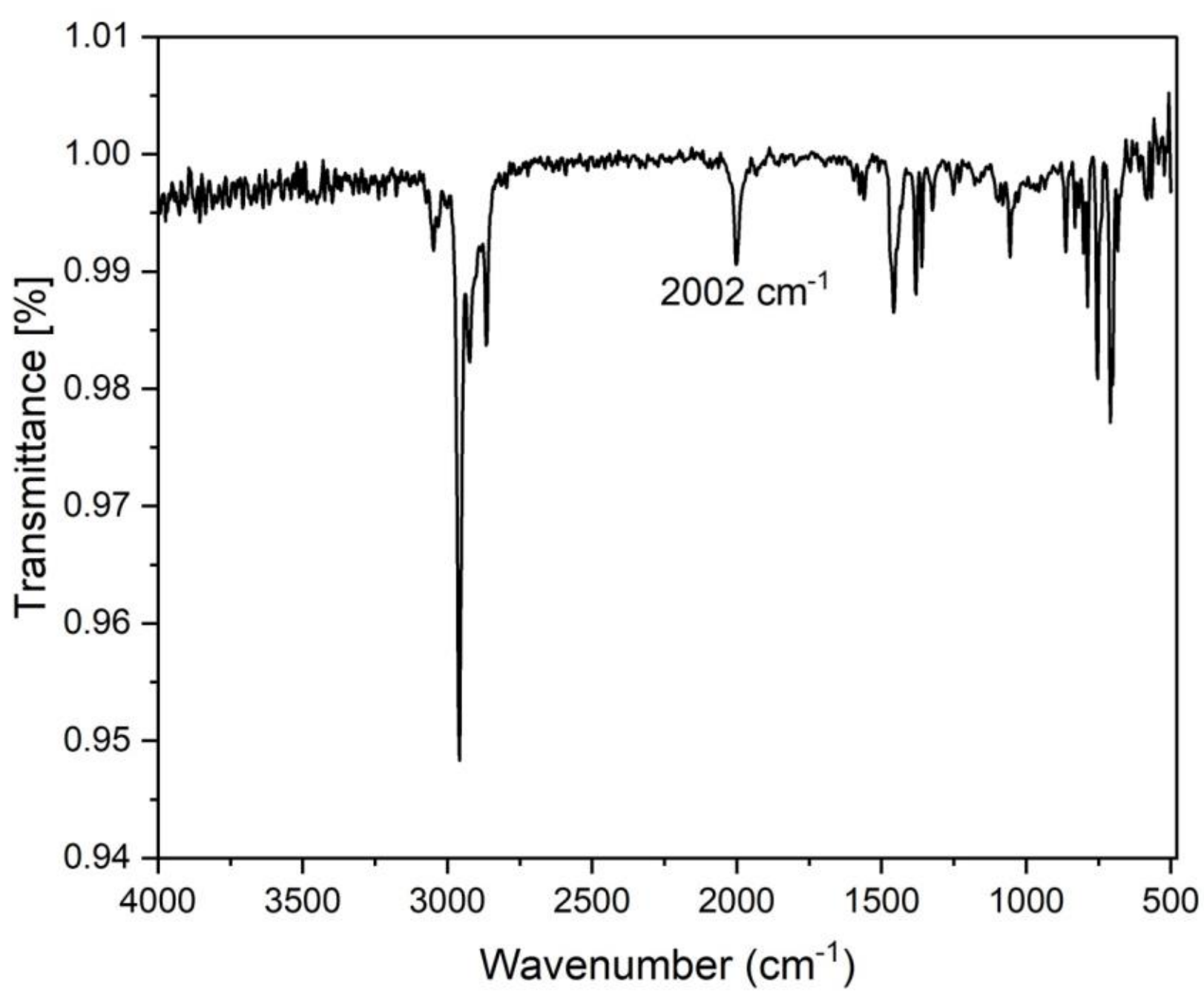

Figure S30. ATR−IR spectrum (solid) of **3** ($\nu_{Ge\text{-}H}$ = 2002 cm^{-1}).

Zero-field ^{57}Fe Mössbauer Measurements

Mössbauer spectra were recorded with a ^{57}Co source in a Rh matrix using an alternating constant acceleration *Wissel* Mössbauer spectrometer operated in transmission mode and equipped with a *Janis* closed-cycle helium cryostat (Göttingen University, Germany). Isomer shifts are given in mm s^{-1} relative to iron metal at ambient temperature. Simulation of the experimental data was performed with the *mf2.SL* program using *Lorentzian* line doublets: E. Bill, Max-Planck Institute for Chemical Energy Conversion, Mülheim/Ruhr, Germany.

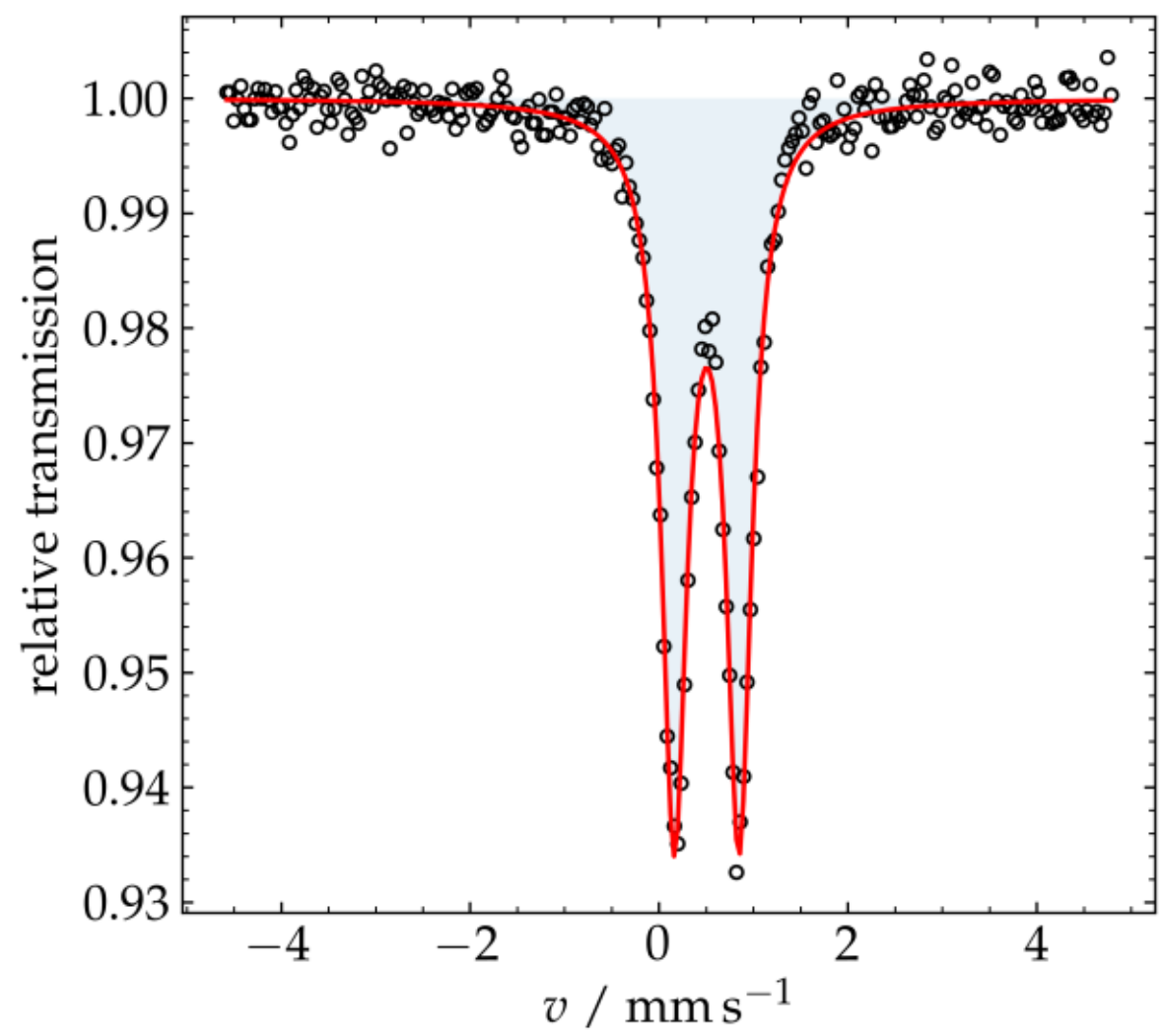

Figure S31. Zero-field ^{57}Fe Mössbauer spectrum of a solid sample of complex **1** (δ = 0.51 mm s^{-1}, $|\Delta E_Q|$ = 0.69 mm s^{-1}, Γ = 0.33 mm s^{-1}) at 80 K.

Crystallographic Data

X-Ray Structure Determination. Crystal data and details of the data collections are given in Tables S1 and S2, molecular structures are shown in Figures S32–S38. Single-crystal X-ray diffraction data were recorded on Rigaku XtaLAB Synergy DW (HyPix-Arc 150) diffractometers with Cu-K_α radiation (λ = 1.54184 Å; for **1**, **2** and **4** – **7**) and Mo-K_α radiation (λ = 0.71073 Å; for **3**). Crystals were selected under mineral oil, mounted on micro mount loops and quench-cooled using an open flow N_2 cooling device. The structures were solved with SHELXT[3] solution program using dual methods and by using Olex2 as the graphical interface.[4] The models were refined with ShelXL[5] using full matrix least squares minimization on F^2. The hydrogen atoms were located in idealized positions and refined isotropically with a riding model. Either semi-empirical multi-scan absorption corrections[6] or analytical ones[7] were applied to the data by using CrysAlisPro (171.43.143a) program.[8]

In **1**, only half of the formula unit is present in the asymmetric unit, with the other half consisting of symmetry equivalent atoms. The Ge1, Ge2 and Fe1 atoms are disordered with respect to a crystallographic inversion center and each atom was refined at half-occupancy at the two disordered sites. SADI (Fe−Ge and Ge−C) restraints were applied.

In **3**, only half of the formula unit is present in the asymmetric unit, with the other half consisting of symmetry equivalent atoms. The Ge1, Ge2 and Fe1 atoms are disordered with respect to a crystallographic inversion center and each atom was refined at half-occupancy at the two disordered sites. DFIX (Ge−H 1.5 Å) restraints were applied.

In **4a**, two independent molecules with similar conformations, bond distances and angles are in one asymmetric unit.

In **4b**, the structure was refined with a twin law of [-1 0 0 0 -1 0 0 0 -1 2] identified by using Olex2, resulting in a BASF twin fraction of 0.453. Three independent molecules with similar conformations, bond distances and angles are in one asymmetric unit. In one molecule, one ^{i}Pr group was found to be disordered over two positions (occupancy factors: 0.39/0.61). It was refined with SADI (C−C) restraints. In another molecule, one Dipp group was found to be disordered over two positions (occupancy factors: 0.58/0.42). It was refined with SADI (C−C), SIMU, RIGU and ISOR restraints.

In **6**, one ^{i}Pr group was found to be disordered over two positions (occupancy factors: 0.68/0.32). One *n*-pentane molecule per asymmetric unit is severely disordered. They were refined by using a solvent mask which was calculated by Olex2, and 166 electrons were found in a volume of 808 Å^3 in one void per unit cell. This is consistent with the presence of 4 *n*-pentane molecules which account for 168 electrons per unit cell. Although these solvent atoms were not included in the refinement model they are included in the formula and in the calculation of the formula weight, density, μ, F(000), etc. As **6** was crystallized from *n*-pentane this serves as evidence for the presence of *n*-pentane in these positions, though they proved to be severely disordered.

In **7**, one *n*-pentane molecule was found to be disordered around a special position and was refined at half-occupancy. It was refined with SADI, DANG (C−C 2.54 Å), DFIX (C−C 1.52 Å), SIMU and RIGU restraints.

Table S1. Crystallographic data and structure refinement details for compounds **1** – **4a**.

**Compound**	**1**	**2**	**3**	**4a**
CCDC	2553713	2553714	2553715	2553716
Empirical formula	$C_{60}H_{74}FeGe_2$	$C_{65}H_{83}FeGe_2N$	$C_{60}H_{76}FeGe_2$	$C_{68}H_{82}FeGe_2$
Formula weight	996.22	1079.35	998.23	1100.36
Temperature/K	123.00(10)	123.00(10)	123.00(10)	123.00(10)
Crystal system	orthorhombic	monoclinic	orthorhombic	monoclinic
Space group	*P*ccn	$P2_1$/n	*P*ccn	$P2_1$
a/Å	15.61120(10)	12.91080(10)	15.6429(2)	12.43830(10)
b/Å	20.63320(10)	22.8398(2)	20.6078(3)	19.30080(10)
c/Å	16.20520(10)	19.6786(2)	16.2435(2)	24.21560(10)
α/°	90	90	90	90
β/°	90	103.2760(10)	90	99.8760(10)
γ/°	90	90	90	90
Volume/$Å^3$	5219.84(5)	5647.75(9)	5236.35(12)	5727.27(6)
Z	4	4	4	4
ρ_{calc} g/cm^3	1.268	1.269	1.266	1.276
μ/mm^{-1}	3.818	3.573	1.45	3.53
F(000)	2096	2280	2104	2320
Crystal size/mm^3	0.152 × 0.124 × 0.085	0.079 × 0.054 × 0.037	0.13 × 0.11 × 0.06	0.17 × 0.16 × 0.07
Radiation	Cu Kα (*λ* = 1.54184)	Cu Kα (*λ* = 1.54184)	Mo Kα (*λ* = 0.71073)	Cu Kα (*λ* = 1.54184)
2Θ range for data collection/°	7.1 to 150.424	6.022 to 150.2	5.016 to 74.016	5.89 to 150.428
Index ranges	-19 ≤ h ≤ 18, -25 ≤ k ≤ 25, -20 ≤ l ≤ 19	-16 ≤ h ≤ 16, -26 ≤ k ≤ 28, -24 ≤ l ≤ 24	-20 ≤ h ≤ 26, -33 ≤ k ≤ 33, -27 ≤ l ≤ 27	-15 ≤ h ≤ 13, -24 ≤ k ≤ 24, -30 ≤ l ≤ 30
Reflections collected	30464	63719	85627	157500
Independent reflections	5319 [R_{int} = 0.0177, R_{sigma} = 0.0130]	11447 [R_{int} = 0.0439, R_{sigma} = 0.0234]	12703 [R_{int} = 0.0395, R_{sigma} = 0.0293]	23022 [R_{int} = 0.0258, R_{sigma} = 0.0201]
Data/restraints/parameters	5319/2/306	11447/0/641	12703/3/314	23022/1/1311
Goodness-of-fit on F^2	1.105	1.089	1.218	1.035
Final R indexes [I>=2σ (I)]	R_1 = 0.0406, wR_2 = 0.1051	R_1 = 0.0461, wR_2 = 0.1410	R_1 = 0.0634, wR_2 = 0.1443	R_1 = 0.0213, wR_2 = 0.0541
Final R indexes [all data]	R_1 = 0.0432, wR_2 = 0.1065	R_1 = 0.0496, wR_2 = 0.1438	R_1 = 0.0860, wR_2 = 0.1514	R_1 = 0.0241, wR_2 = 0.0551
Largest diff. peak/hole / e $Å^{-3}$	0.40/−0.62	1.26/−0.78	0.99/−0.56	0.43/−0.34

Table S2. Crystallographic data and structure refinement details for compounds **4b** – **7**.

**Compound**	**4b**	**5**	**6**	**7**
CCDC	2553717	2553720	2553718	2553719
Empirical formula	$C_{68}H_{82}FeGe_2$	$C_{66}H_{84}FeGe_2$	$C_{66}H_{84}FeGe_2{\cdot}C_5H_{12}$	$C_{66}H_{84}FeSn_2{\cdot}0.5C_5H_{12}$
Formula weight	1100.36	1078.36	1150.5	1206.63
Temperature/K	123.00(10)	123.00(10)	123.00(10)	123.00(10)
Crystal system	monoclinic	monoclinic	monoclinic	triclinic
Space group	Cc	*C*c	*P*2_1/n	P-1
a/Å	57.3332(3)	18.5683(2)	11.18347(4)	12.3021(3)
b/Å	13.08435(6)	17.5342(2)	28.28000(9)	14.0812(2)
c/Å	23.35776(10)	18.1352(2)	20.03699(7)	19.1595(3)
α/°	90	90	90	76.9690(10)
β/°	94.1503(4)	106.0490(10)	96.6501(3)	83.9530(10)
γ/°	90	90	90	74.000(2)
Volume/Å^3	17476.30(14)	5674.34(11)	6294.43(4)	3104.93(11)
Z	12	4	4	2
ρ_{calc}g/cm^3	1.255	1.262	1.214	1.291
μ/mm^{-1}	3.471	3.551	3.23	8.465
F(000)	6960	2280	2448	1254
Crystal size/mm^3	0.123 × 0.104 × 0.046	0.104 × 0.076 × 0.038	0.166 × 0.116 × 0.061	0.239 × 0.163 × 0.104
Radiation	Cu Kα (*λ* = 1.54184)	Cu Kα (*λ* = 1.54184)	Cu Kα (*λ* = 1.54184)	Cu Kα (*λ* = 1.54184)
2Θ range for data collection/°	6.182 to 151.558	7.068 to 150.478	5.43 to 150.522	4.74 to 150.634
Index ranges	-71 ≤ h ≤ 70, -15 ≤ k ≤ 16, -24 ≤ l ≤ 29	-23 ≤ h ≤ 22, -21 ≤ k ≤ 21, -20 ≤ l ≤ 22	-13 ≤ h ≤ 13, -35 ≤ k ≤ 35, -24 ≤ l ≤ 24	-15 ≤ h ≤ 15, -17 ≤ k ≤ 17, -23 ≤ l ≤ 24
Reflections collected	183253	69674	162449	63492
Independent reflections	32225 [R_{int} = 0.0282, R_{sigma} = 0.0258]	10167 [R_{int} = 0.0272, R_{sigma} = 0.0203]	12841 [R_{int} = 0.0226, R_{sigma} = 0.0099]	12613 [R_{int} = 0.0266, R_{sigma} = 0.0162]
Data/restraints/parameters	32225/219/2110	10167/2/640	12841/0/661	12613/61/687
Goodness-of-fit on F^2	1.028	1.02	1.028	1.055
Final R indexes [I>=2σ (I)]	R_1 = 0.0317, wR_2 = 0.0799	R_1 = 0.0220, wR_2 = 0.0561	R_1 = 0.0229, wR_2 = 0.0609	R_1 = 0.0218, wR_2 = 0.0577
Final R indexes [all data]	R_1 = 0.0351, wR_2 = 0.0831	R_1 = 0.0234, wR_2 = 0.0568	R_1 = 0.0238, wR_2 = 0.0613	R_1 = 0.0224, wR_2 = 0.0580
Largest diff. peak/hole / e Å^{-3}	0.55/−0.37	0.54/−0.30	0.85/−0.54	0.62/−0.65

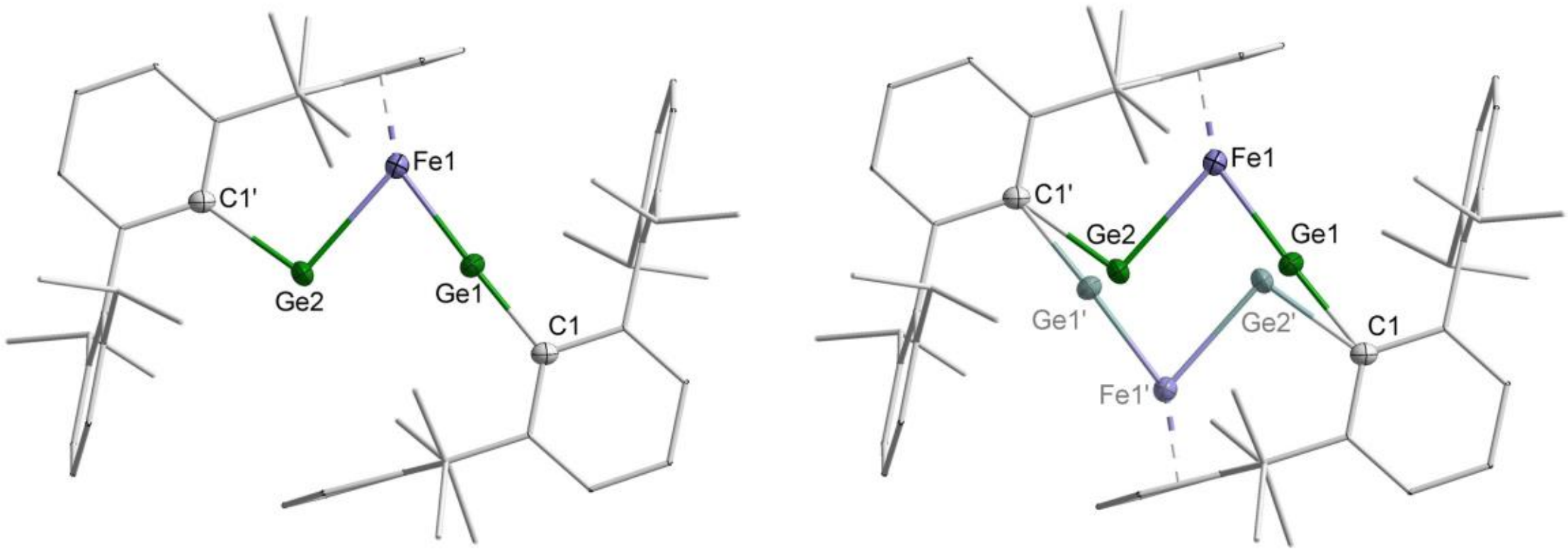

Figure S32. Plot (50% probability thermal ellipsoids, hydrogen atoms omitted) of the solid-state molecular structure of **1** without (left) and with (right) the disordered Fe and Ge atoms. Selected bond lengths [Å] and angles [°]: Fe1−Ge1 2.1061(9), Fe1−Ge2 2.4194(9), Ge1−C1 1.928(2), Ge2−C1' 2.084(2), Ge1···Ge2 2.8738(9), Ge1−Fe1−Ge2 78.51(3), Fe1−Ge1−C1 176.86(7), Fe1−Ge2−C1' 95.77(6).

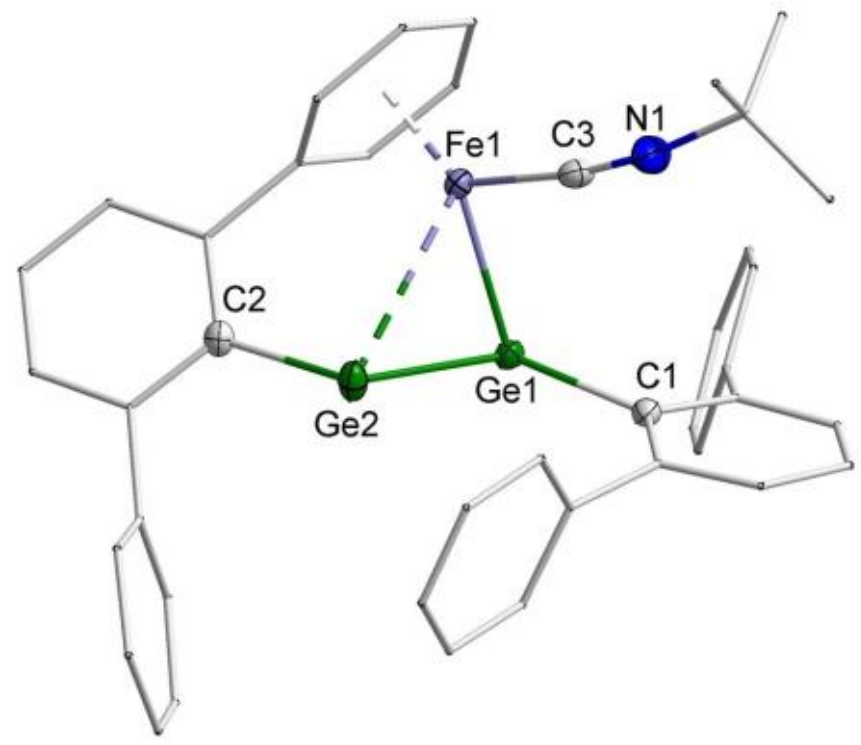

Figure S33. Plot (50% probability thermal ellipsoids, hydrogen atoms omitted) of the solid-state molecular structure of **2**. Selected bond lengths [Å] and angles [°]: Fe1−Ge1 2.2916(5), Fe1−Ge2 2.9513(6), Fe1−C3 1.831(3), Ge1−C1 1.993(3), Ge2−C2 2.018(3), Ge1−Ge2 2.3787(5), Ge1−Fe1−Ge2 52.14(1), Fe1−Ge1−C1 121.39(8), Fe1−Ge2−C2 91.03(8), C1−Ge1−Ge2 119.09(8), C2−Ge2−Ge1 113.66(8).

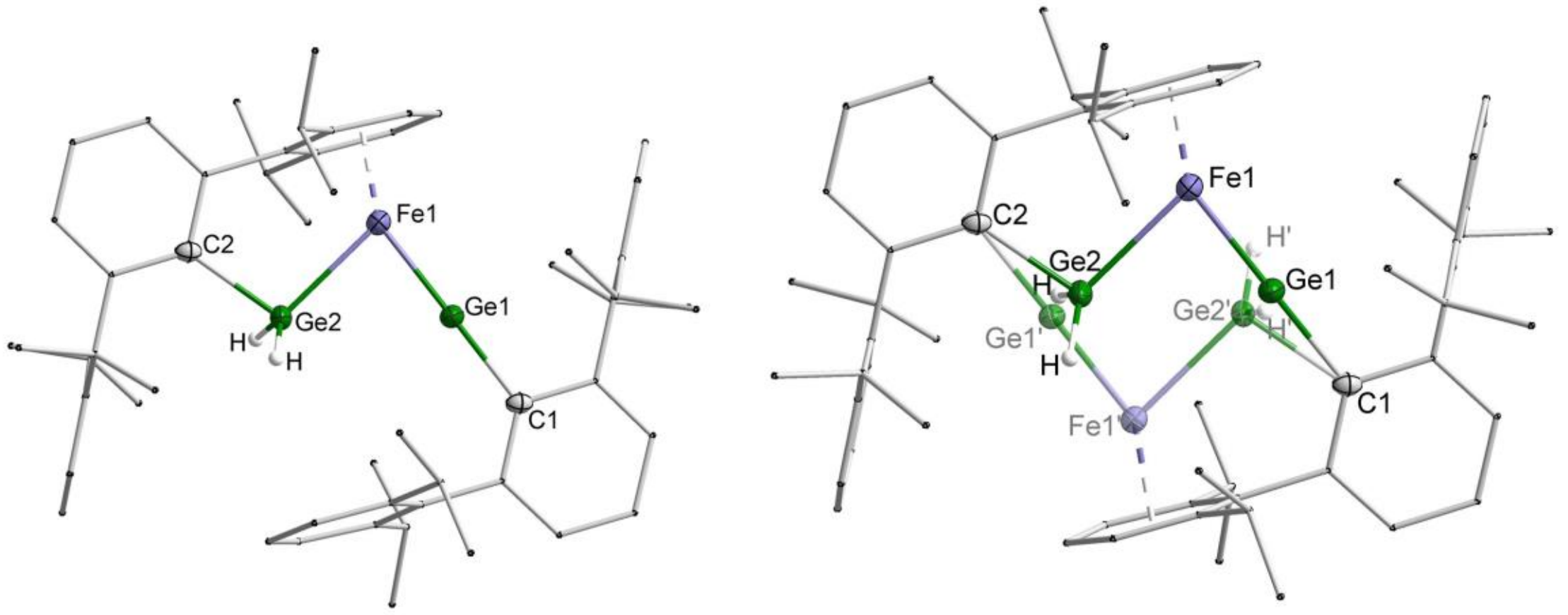

Figure S34. Plot (50% probability thermal ellipsoids, hydrogen atoms except the Ge-bound H atoms omitted) of the solid-state molecular structure of **3** without (left) and with (right) the disordered Fe, Ge and H atoms. Selected bond lengths [Å] and angles [°]: Fe1−Ge1 2.0994(6), Fe1−Ge2 2.3968(7), Ge1−C1 1.930(2), Ge2−C1' 2.006(2), Ge1···Ge2 3.0161(6), Ge1−Fe1−Ge2 83.98(2), Fe1−Ge1−C1 176.03(6), Fe1−Ge2−C1' 101.32(5).

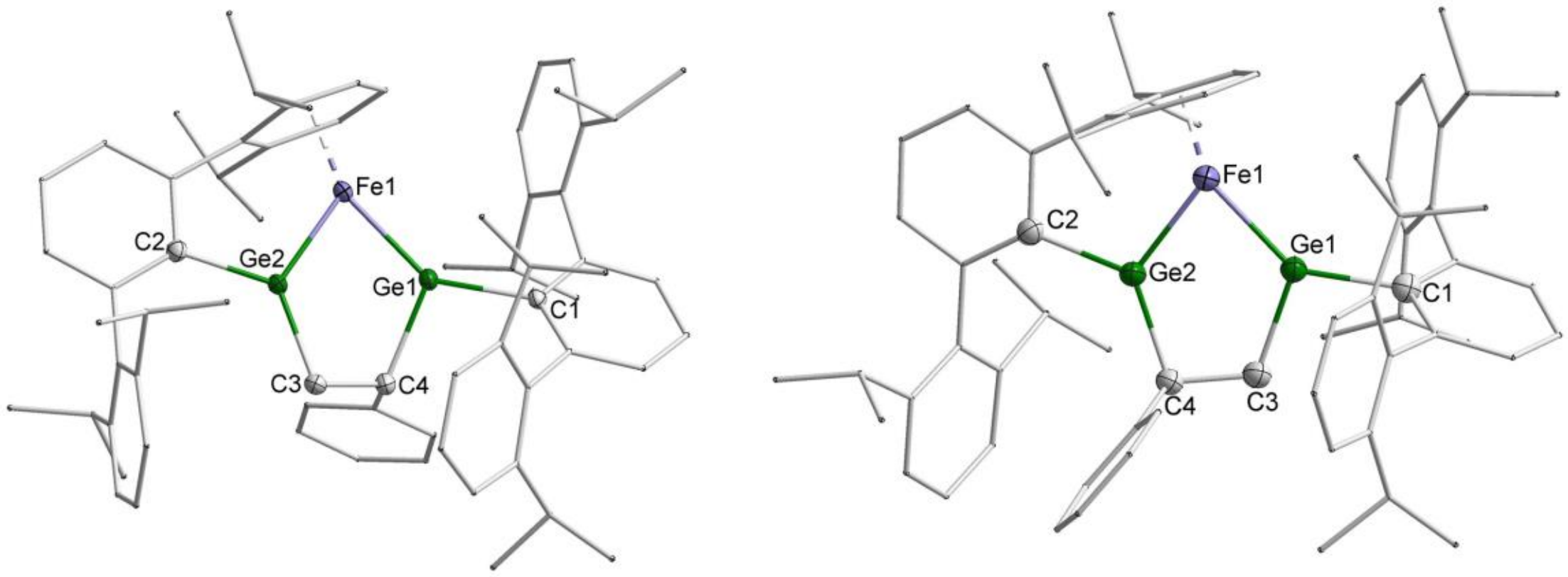

Figure S35. Plot (50% probability thermal ellipsoids, hydrogen atoms omitted) of the solid-state molecular structure of **4a** (left) and **4b** (right). Selected bond lengths [Å] and angles [°] of **4a**: Fe1−Ge1 2.2441(4), Fe1−Ge2 2.1857(4), Ge1−C1 2.020(2), Ge1−C4 2.074(2), Ge2−C2 1.955(2), Ge2−C3 1.970(2), C3−C4 1.534(3), Ge1···Ge2 2.8442(3), Ge1−Fe1−Ge2 79.88(1), C1−Ge1−C4 102.54(8), C2−Ge2−C3 128.02(9). **4b**: Fe1−Ge1 2.2171(8), Fe1−Ge2 2.1833(7), Ge1−C1 2.001(4), Ge1−C3 2.026(4), Ge2−C2 1.976(4), Ge2−C4 2.000(4), C3−C4 1.533(5), Ge1···Ge2 2.8497(6), Ge1−Fe1−Ge2 80.72(3), C1−Ge1−C3 100.42(16), C2−Ge2−C4 129.25(16).

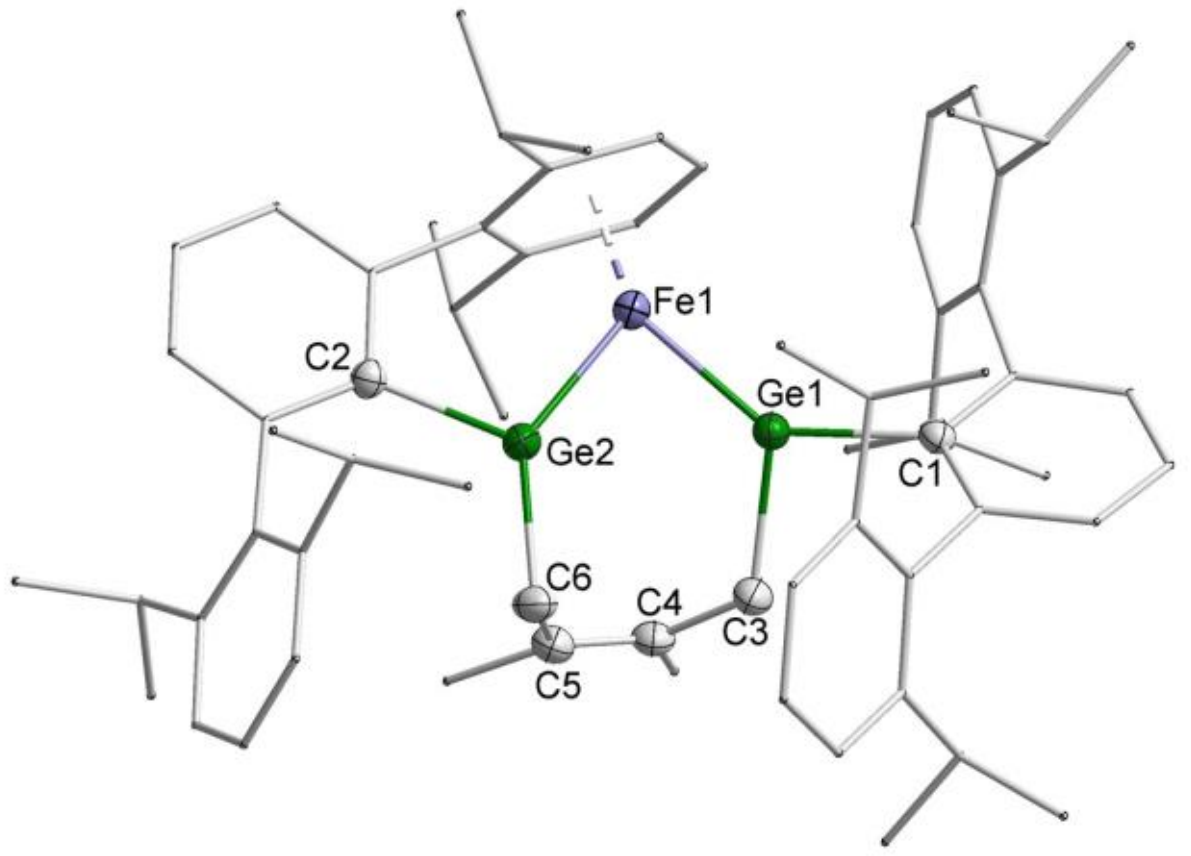

Figure S36. Plot (50% probability thermal ellipsoids, hydrogen atoms omitted) of the solid-state molecular structure of **5**. Selected bond lengths [Å] and angles [°]: Fe1−Ge1 2.2260(5), Fe1−Ge2 2.2150(5), Ge1−C1 2.030(3), Ge1−C6 2.012(3), Ge2−C2 1.990(3), Ge2−C3 1.987(3), C4−C5 1.344(4), Ge1···Ge2 3.0663(6), Ge1−Fe1−Ge2 87.33(2), C1−Ge1−C6 96.05(11), C2−Ge2−C3 116.60(12).

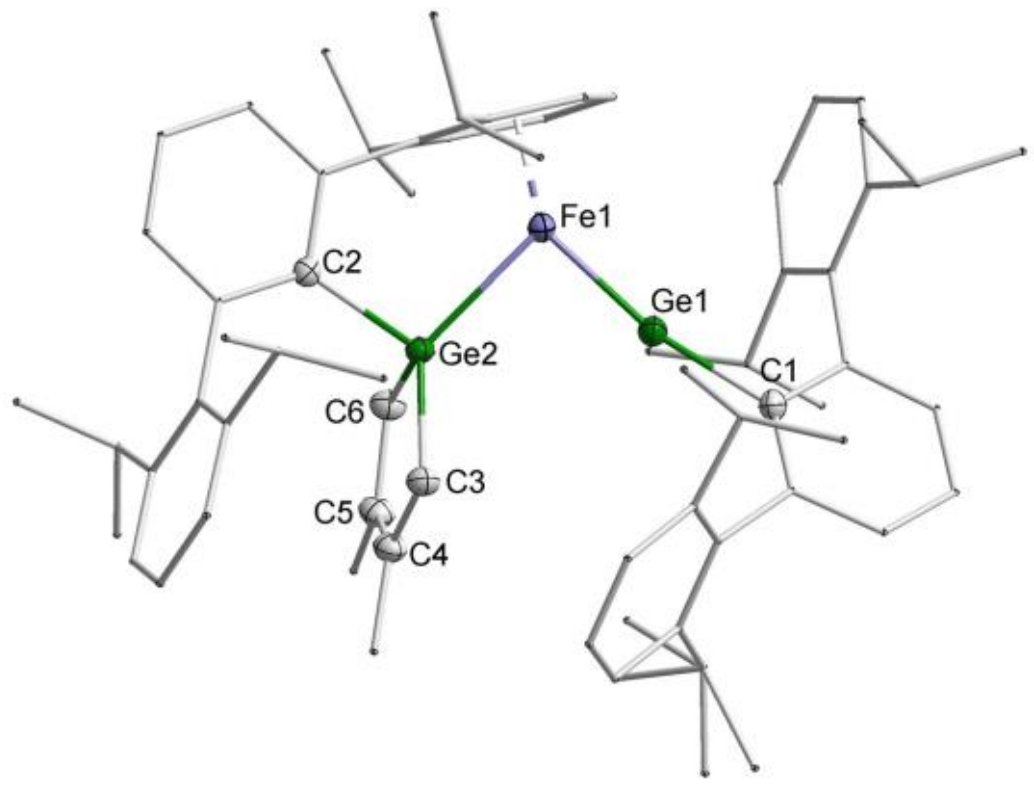

Figure S37. Plot (50% probability thermal ellipsoids, hydrogen atoms and solvent molecules (*n*-pentane) omitted) of the solid-state molecular structure of **6**. Selected bond lengths [Å] and angles [°]: Fe1−Ge1 2.1069(2), Fe1−Ge2 2.4021(2), Ge1−C1 1.956(1), Ge2−C2 2.010(1), Ge2−C3 1.982(1), Ge2−C6 1.993(1), C4−C5 1.337(2), Ge1···Ge2 3.2368(8), Fe1−Ge1−C1 168.39(4), Ge1−Fe1−Ge2 91.514(8), Fe1−Ge2−C2 101.03(3), C3−Ge2−C6 90.70(5).

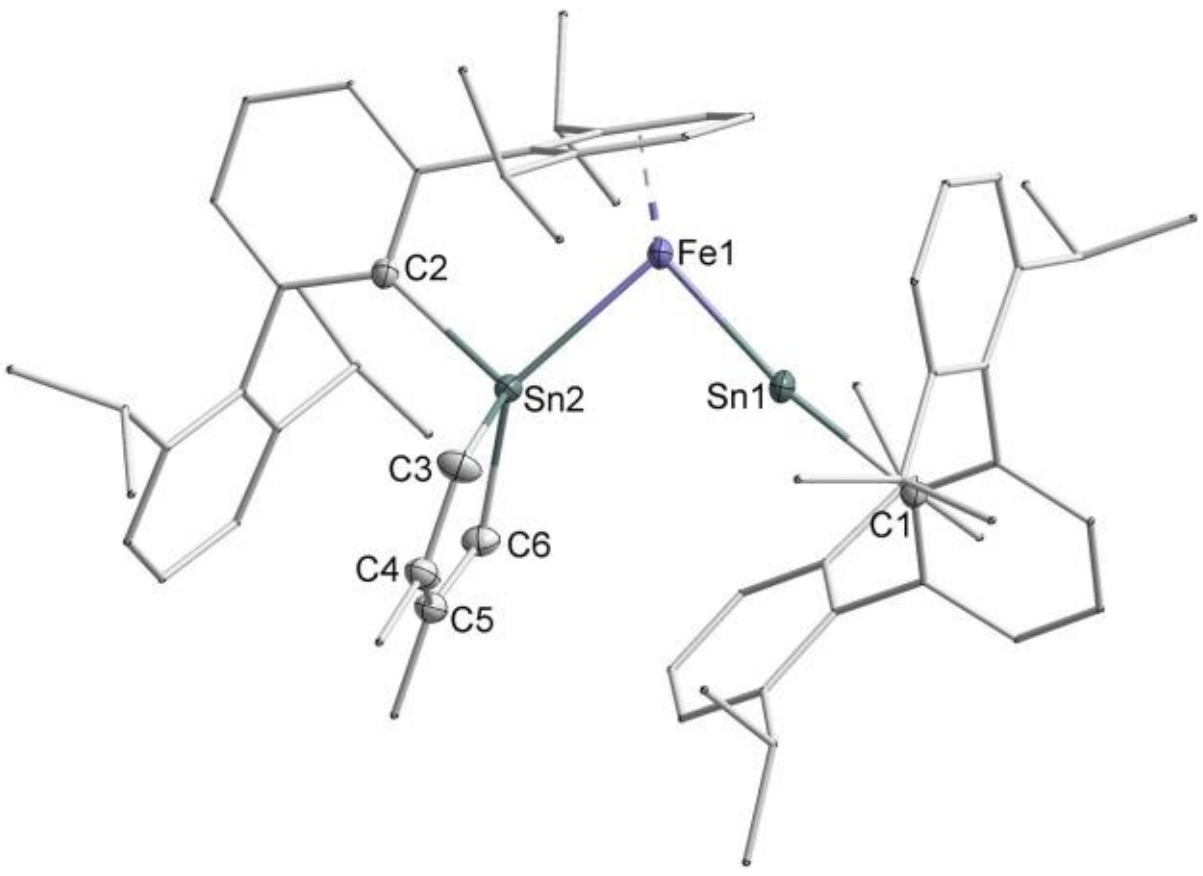

Figure S38. Plot (50% probability thermal ellipsoids, hydrogen atoms and solvent molecules (*n*-pentane) omitted) of the solid-state molecular structure of **7**. Selected bond lengths [Å] and angles [°]: Fe1−Sn1 2.2904(3), Fe1−Sn2 2.5778(3), Sn1−C1 2.1428(16), Sn2−C2 2.2016(15), Sn2−C3 2.1849(17), Sn2−C6 2.1648(16), C4−C5 1.342(2), Sn1···Sn2 3.4730(4), Fe1−Sn1−C1 170.97(4), Sn1−Fe1−Sn2 90.829(9), Fe1−Sn2−C2 95.72(4), C3−Sn2−C6 84.72(6).

DFT Calculations

Computational Details. All calculations were performed using the ORCA quantum-chemical program package (version 5.0.4 or 6.0).[9,10] Geometry optimizations (OPT) were performed with the BP86 functional[11] and the def2-TZVP basis set[12] in combination with the auxiliary basis set def2/J.[13] Effective core potentials (ECP) were used for Sn atoms.[14] The RI approximation was used to accelerate the calculations.[15] The noncovalent interactions were considered via atom-pairwise dispersion corrections with Becke-Johnson (D3BJ) damping.[16] Coordinates from X-ray structural analyses were used as starting coordinates and the optimized coordinates of **1**–**7** are given in Table S10. Numerical frequency calculations at the same level of theory were performed to identify the number of imaginary frequencies (zero for local minimum) and provide the energy difference between Gibbs free energy and electronic energy (G−E(el)). Optimized structures were visualized by the IboView[17] or Chemcraft[18] program. Single-point energy calculations for **1**-**6** were performed using the ωB97X-D3 functional[19] with the same basis set combination and the RIJCOSX approximation.[20] For complex **7**, the ZORA-def2-TZVP basis set[12b] was used for all atoms except Sn, for which the SARC-ZORA-TZVP basis set[21] was employed. For the sake of computational efficiency, the energy profile of the hydrogenation of **1** (Figure S62) was calculated at the BP86-D3BJ/def2-SVP (C, H)/def2-TZVP (Fe, Ge) level of theory. The Intrinsic Reaction Coordinate (IRC)[22] was used to confirm the connectivity between a transition state (TS) with its downhill-nearest intermediates. Time-dependent density functional theory (TD-DFT) calculations (80 roots) were carried out at the same level of theory in gas phase for **1**.[23] The resulting excitation energies and simulated UV-vis spectrum are summarized in Figure S40. Intrinsic bond orbitals (IBO)[17] were calculated using the ORCA program at the ωB97X-D3 level of theory. Natural bond orbital (NBO) calculations were carried out using the NBO 6.0 program.[24] QTAIM analyses were performed using Multiwfn.[25] The relative Gibbs free energies of **5** and **6** were additionally calculated using the r²SCAN-3c composite method at 298 K in the gas phase (Figure S60).[26]

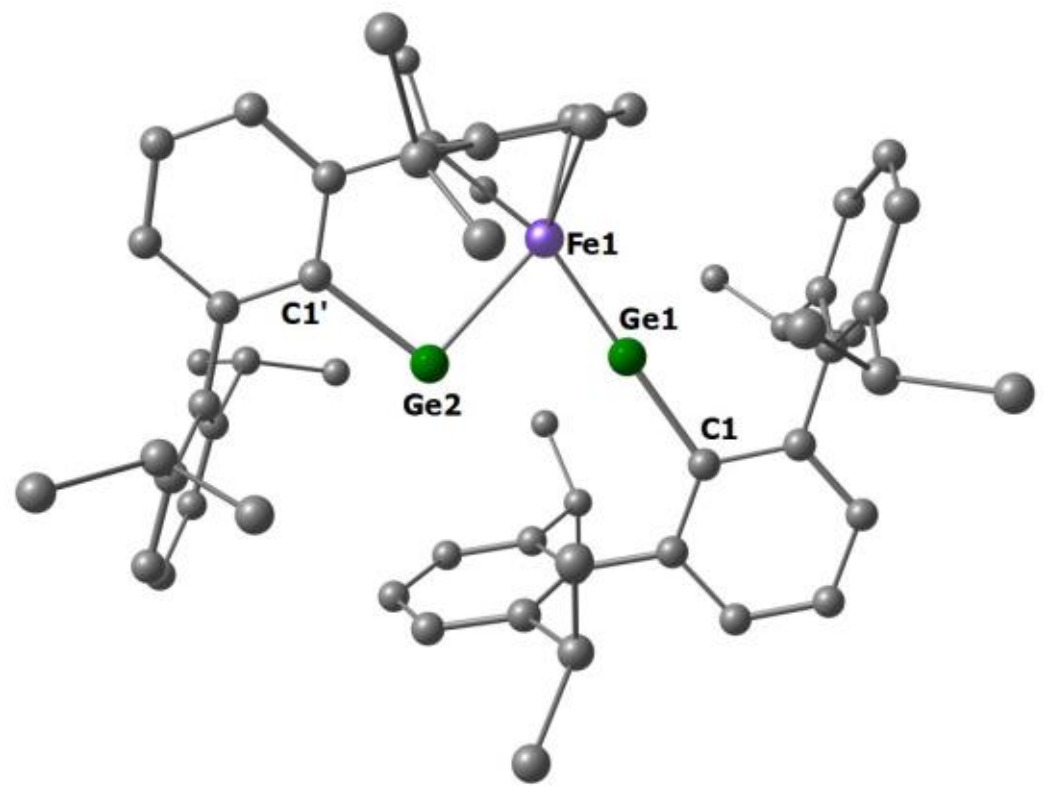

Figure S39. The optimized structure of **1** (BP86/def2-TZVP level). Hydrogen atoms are omitted for clarity.

Table S3. Comparison of the selected bond distances (Å) and bond angles (°) obtained from the crystal structure and the optimized geometries of **1**.

	**Exp.**	**Opt.**
Fe1–Ge1/Ge2	2.1061(9)/ 2.4194(9)	2.101/ 2.441
Ge1/Ge2–C_{ipso}	1.928(2)/ 2.084(2)	1.956/ 2.058
Ge1···Ge2	2.8738(9)	2.866
Fe1–Ge1/Ge2–C_{ipso}	176.86(7)/ 95.77(6)	176.01/ 94.45

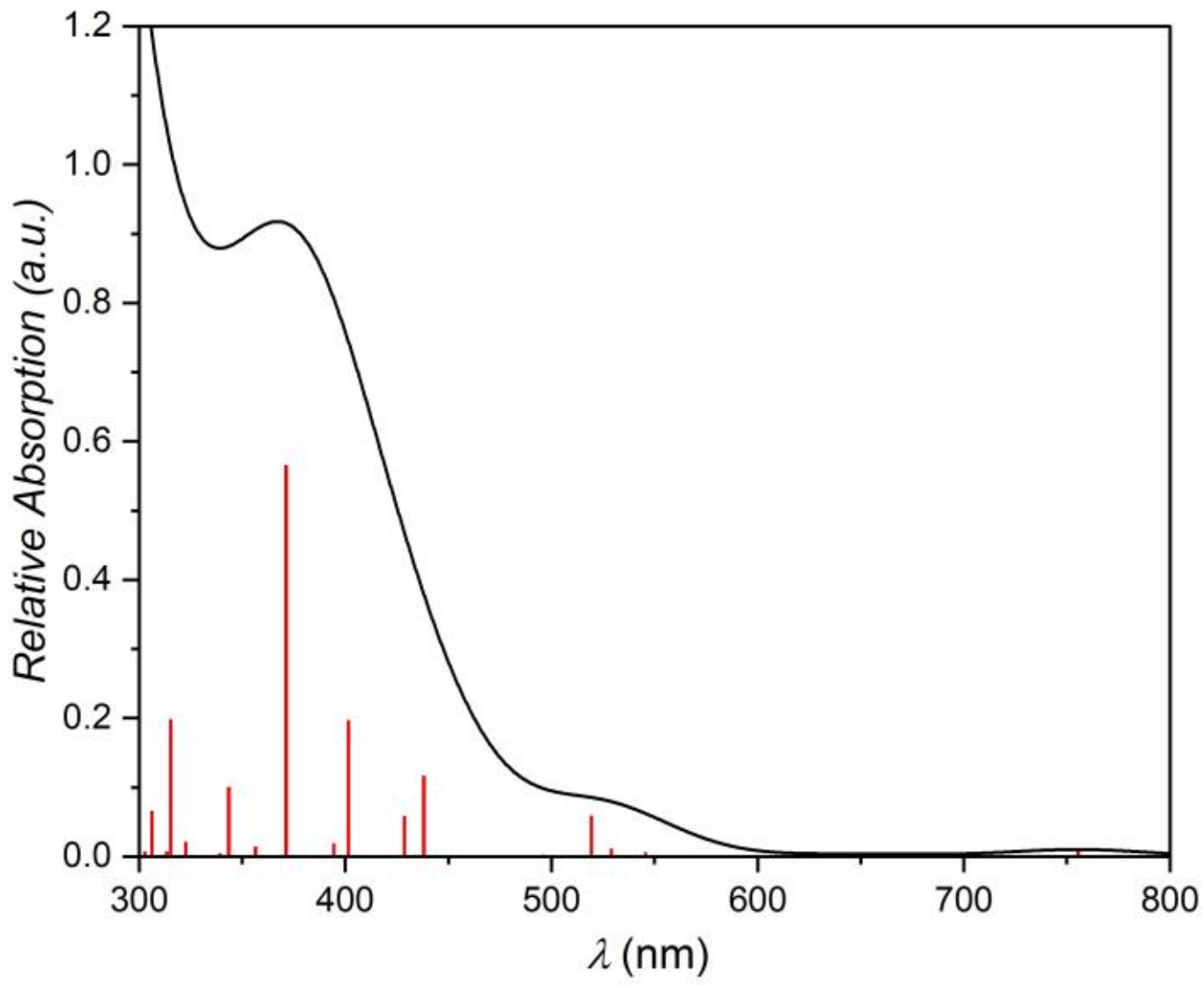

Figure S40. TD-DFT calculated UV-vis spectra for **1**. The calculated spectrum was convoluted using a Gaussian line shape function with a half-width of 80 nm. The principal contributions involved in the electronic transitions at 371 nm, 519 nm, 594 nm and 756 nm are HOMO-1 → LUMO (24%) and HOMO-4 → LUMO+2 (12%), HOMO-1 → LUMO+2 (32%) and HOMO → LUMO+2 (30%), HOMO → LUMO (26%) and HOMO-4 → LUMO+1 (15%), and HOMO → LUMO+1 (64%) and HOMO → LUMO (22%), respectively.

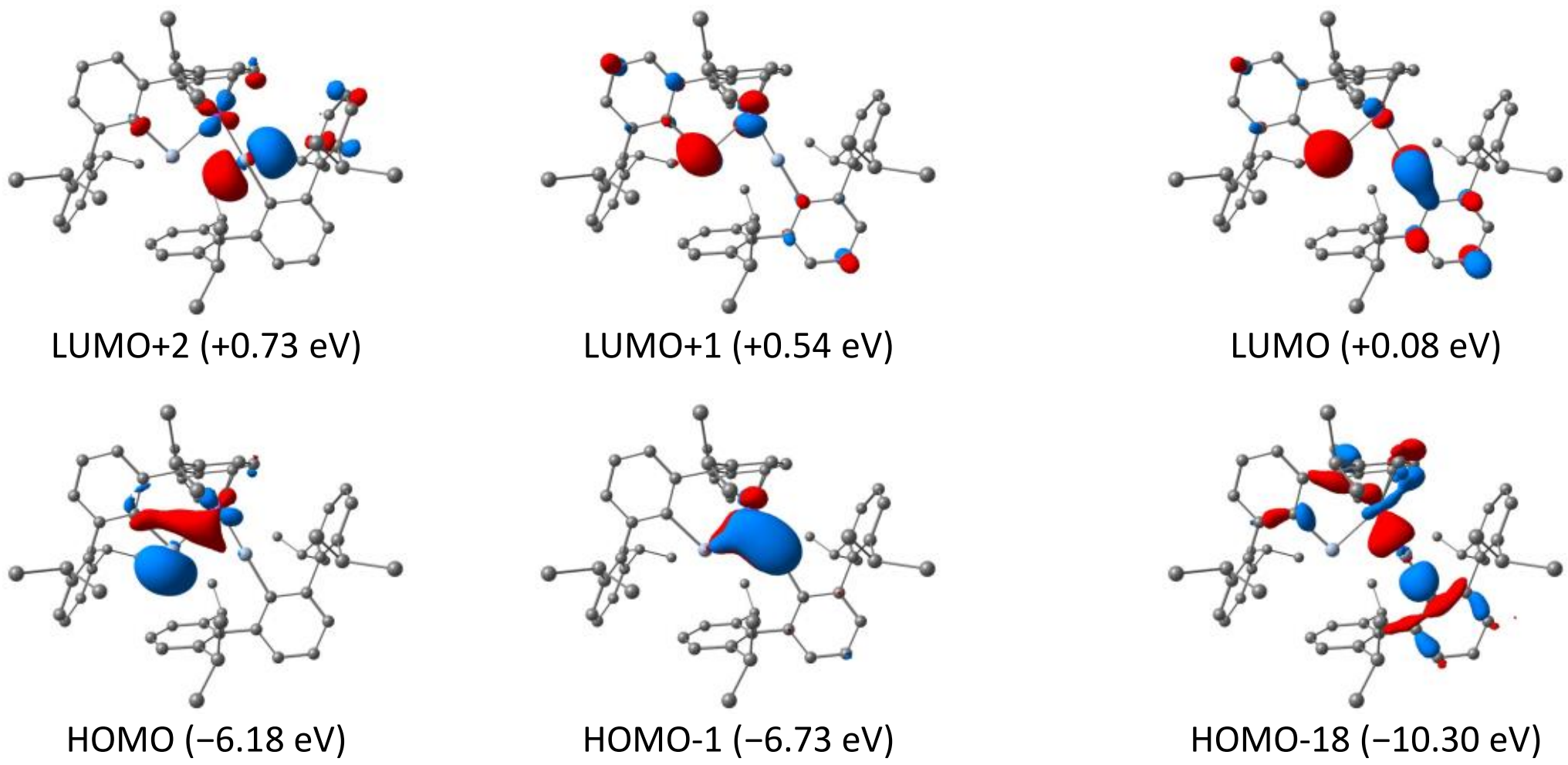

Figure S41. Selected molecular orbitals of **1** (Isovalue = 0.04; ωB97X-D3/def2-TZVP level).

MO and IBO analyses for complex 1: The short Fe1−Ge1 bond length, the nearly linear Fe1−Ge1−C1 unit, and the Mayer bond order (1.65) suggest a multiple bond between Fe1 and Ge1. The MO and IBO analyses are consistent with this assignment. The HOMO-18 and the related IBO (Figure 3b) reveal an Fe1−Ge1 σ-bonding interaction, while an out-of-plane Fe1−Ge1 π interaction in HOMO-1 and the corresponding IBO support a Fe=Ge double-bond assignment. The HOMO (Figure S41) is primarily characterized by σ bonding between Fe1 and Ge2, with contributions of 51% from Fe1, 24% from Ge2, and only 3.6% from Ge1. The related IBO (Figure 3b) also describes the Fe1−Ge2 σ-bond as a dominant interaction. Similar features are seen in complexes **3** and **6** (Figures S46 and S55). A significant in-plane π interaction between Fe1 and Ge1 was not found. These results support an Fe1=Ge1 double-bond description for compounds **1**, **3**, and **6**.

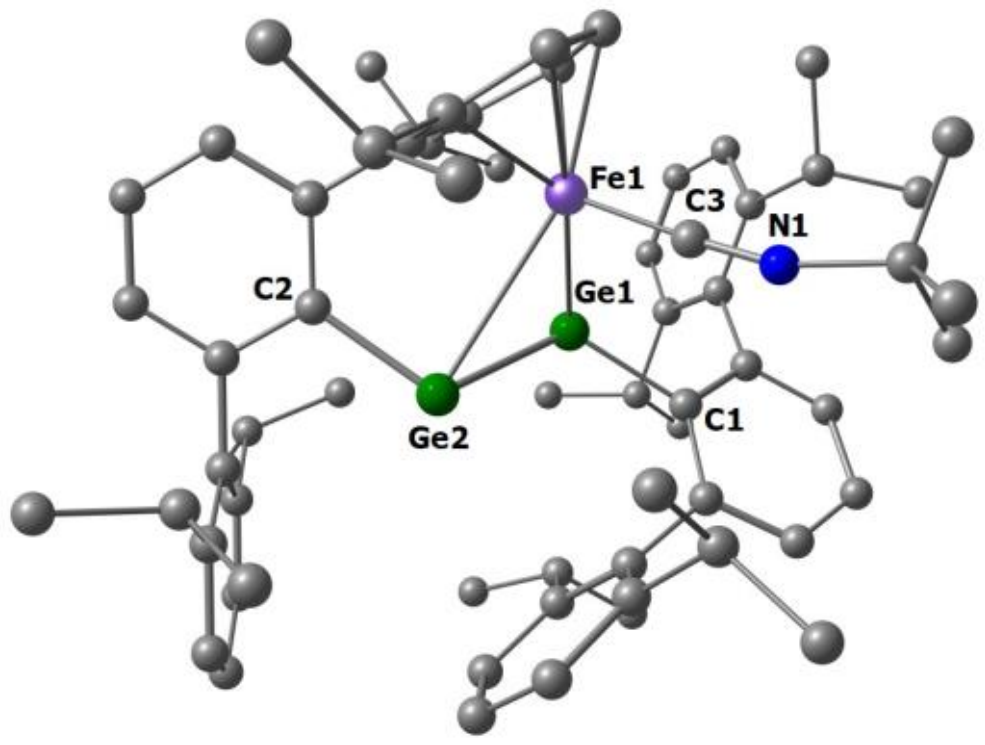

Figure S42. The optimized structure of **2** (BP86/def2-TZVP level). Hydrogen atoms are omitted for clarity.

Table S4. Comparison of the selected bond distances (Å) and bond angles (°) obtained from the crystal structure and the optimized geometries of **2**.

	**Exp.**	**Opt.**
Fe1–Ge1/Ge2	2.2916(5)/ 2.9513(6)	2.269/3.070
Ge1/Ge2–C*ipso*	1.993(3)/ 2.018(3)	1.991/2.037
Ge1–Ge2	2.3787(5)	2.412
Fe1–C3	1.831(3)	1.783
Fe1–Ge1/Ge2–C*ipso*	121.39(8)/ 91.03(8)	122.21/88.17
C1–Ge1–Ge2	119.09(8)	120.76
C2–Ge2–Ge1	113.66(8)	107.71

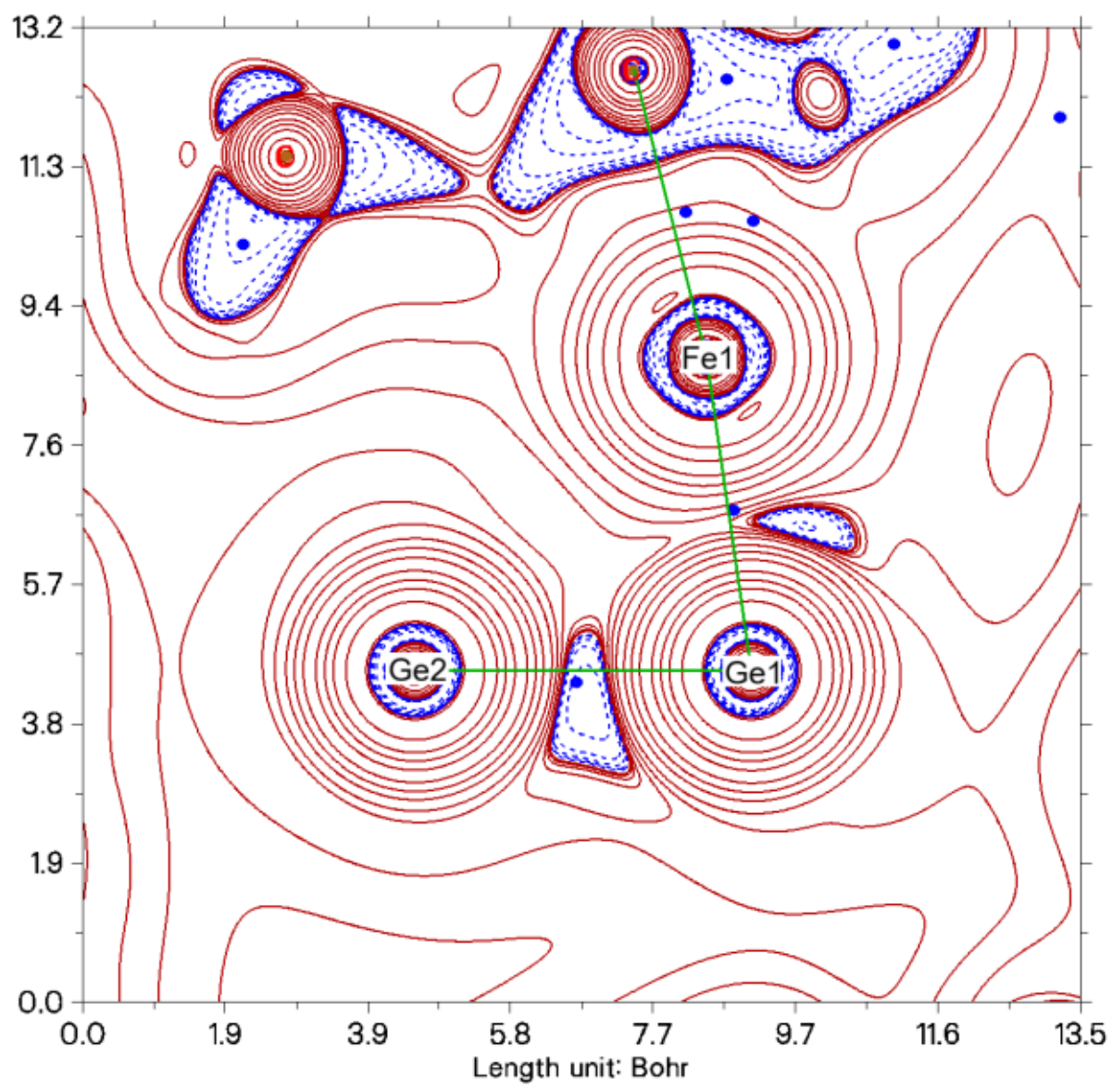

Figure S43. Plot of the Laplacian of the electron density on the $FeGe_2$ plane of **2** (BCP: blue dots, bond paths: green lines).

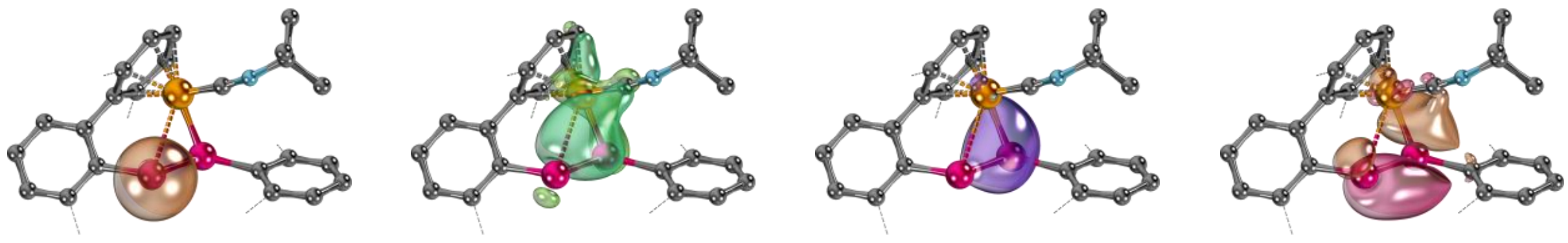

electron lone pair
0.84 Ge2/0.05 Ge1

Fe1–Ge2 σ-bond
Ge1–Ge2 σ-bond
0.47 Fe1/0.18 Ge1/0.23 Ge2

Fe1–Ge1 σ-bond
Ge1–Ge2 σ-bond
0.19 Fe1/0.67 Ge1

Fe1–Ge1 σ-bond
Ge1–Ge2 π-bond
0.21 Fe1/0.54 Ge1/0.25 Ge2

Figure S44. Selected IBOs of **2**. The bond type and percentage of the electron density on Fe and Ge are given. The Dipp substituents and ^{i}Pr substituents of the η^{6}-Dipp are omitted for clarity.

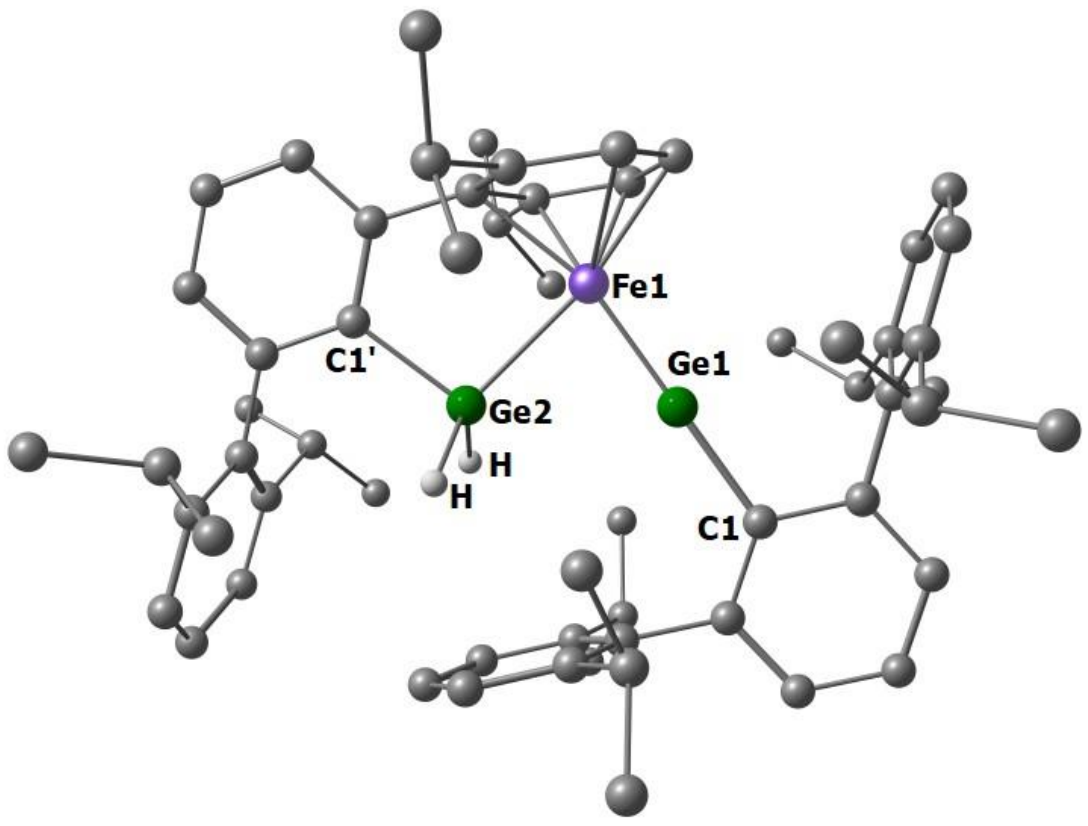

Figure S45. The optimized structure of **3** (BP86/def2-TZVP level). Hydrogen atoms except Ge–*H* are omitted for clarity.

Table S5. Comparison of the selected bond distances (Å) and bond angles (°) obtained from the crystal structure and the optimized geometries of **3**.

	**Exp.**	**Opt.**
Fe1–Ge1/Ge2	2.0994(6)/ 2.3968(7)	2.086/2.408
Ge1/Ge2–C_{ipso}	1.930(2)/ 2.006(2)	1.944/1.988
Ge1···Ge2	3.0161(6)	2.942
Ge2–H	1.59(2)/1.59(2)	1.558/1.556
Fe1–Ge1/Ge2–C_{ipso}	176.03 (6)/ 101.32(5)	175.29/99.35

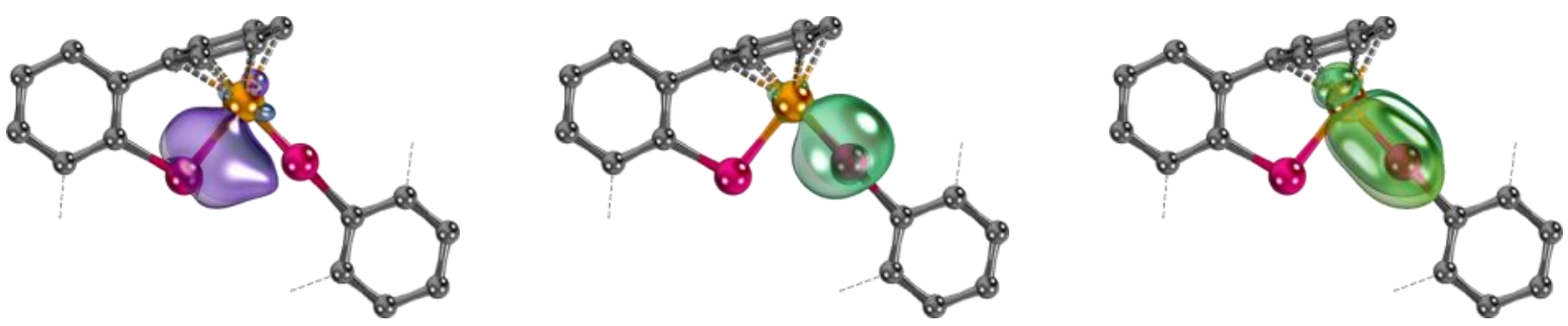

Fe1–Ge2 σ-bond
0.66 Fe1/0.23 Ge2

Fe1–Ge1 σ-bond
0.41 Fe1/0.47 Ge1

Fe1–Ge1 π-bond
0.48 Fe1/0.47 Ge1

Figure S46. Selected IBOs of **3**. The bond type and percentage of the electron density on Fe and Ge are given. The Dipp substituents and ^{i}Pr substituents of the η^{6}-Dipp are omitted for clarity.

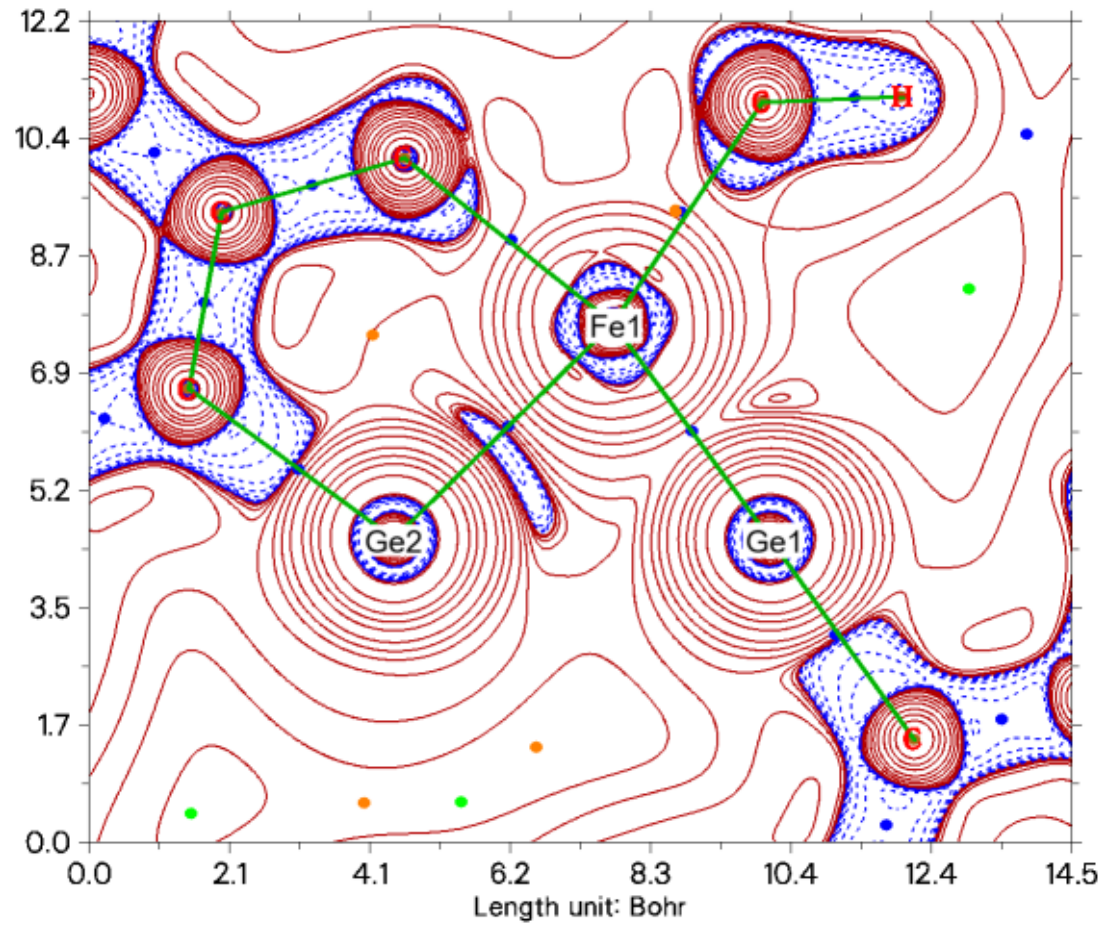

Figure S47. Plot of the Laplacian of the electron density on the $FeGe_2$ plane of **3** (BCP: blue dots, RCP: brown dots, Cage critical points (CCP): green dots, bond paths: green lines).

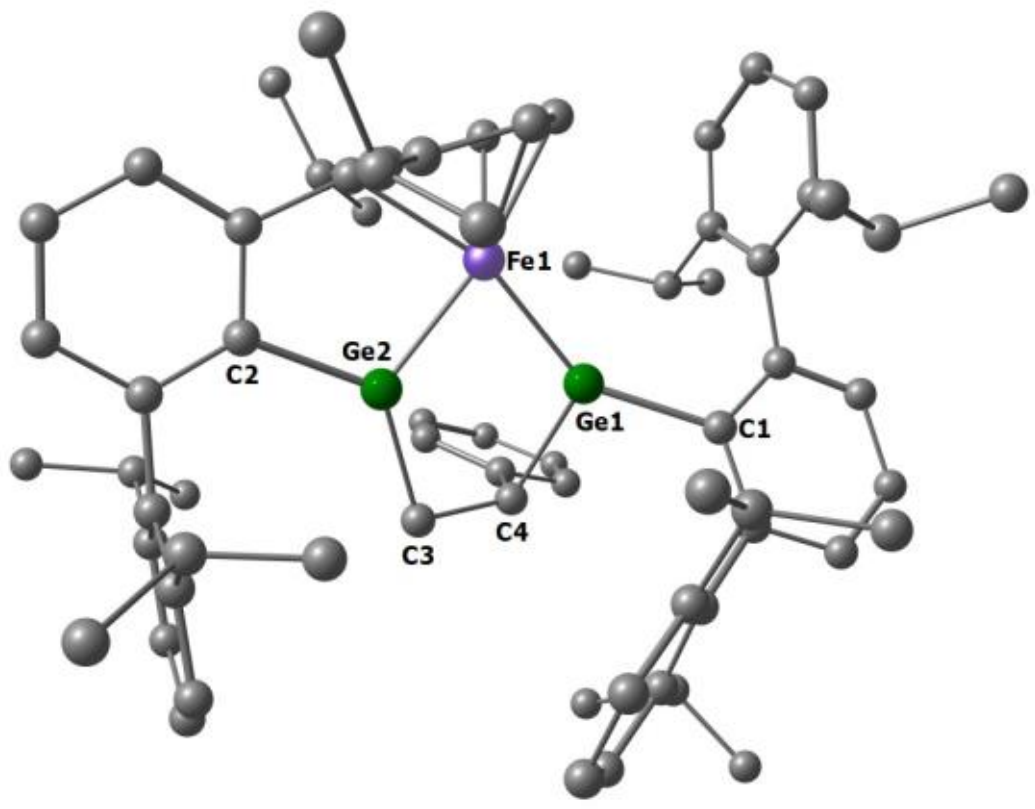

Figure S48. The optimized structure of **4a** (BP86/def2-TZVP level). Hydrogen atoms are omitted for clarity.

Table S6. Comparison of the selected bond distances (Å) and bond angles (°) obtained from the crystal structure and the optimized geometries of **4a**.

	**Exp.**	**Opt.**
Fe1–Ge1/Ge2	2.2441(4)/ 2.1857(4)	2.237/2.191
Ge1/Ge2–C_{ipso}	2.020(2)/ 1.955(2)	2.014/1.964
Ge1–C4/Ge2–C3	2.074(2)/ 1.970(2)	2.095/ 1.982
C3–C4	1.534(3)	1.524
Ge1···Ge2	2.8442(3)	2.799
C1–Ge1–C4	102.54(8)	99.42
C2–Ge2–C3	128.02(9)	128.43

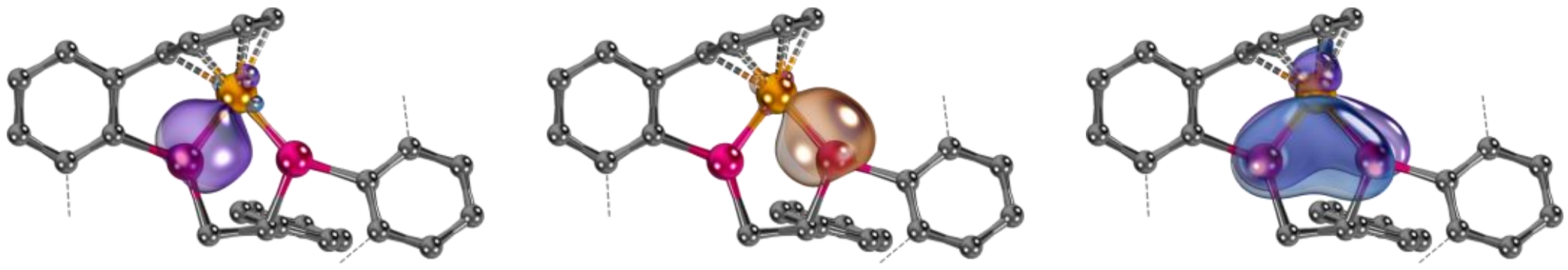

Figure S49. Selected IBOs of **4a**. The bond type and percentage of the electron density on Fe and Ge are given. The Dipp substituents and ^{i}Pr substituents of the η^6-Dipp are omitted for clarity.

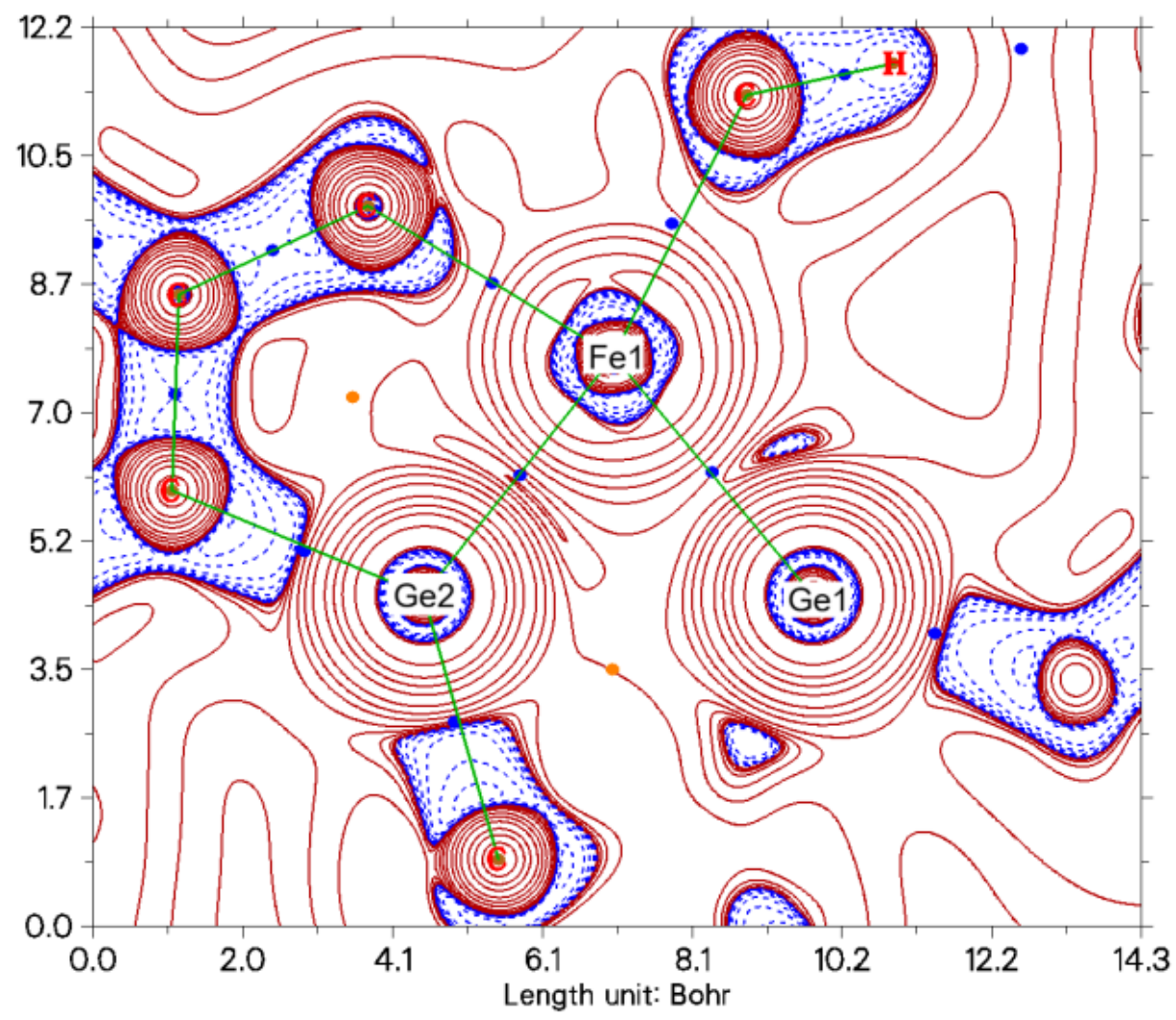

Figure S50. Plot of the Laplacian of the electron density on the $FeGe_2$ plane of **4a** (BCP: blue dots, RCP: brown dots, bond paths: green lines).

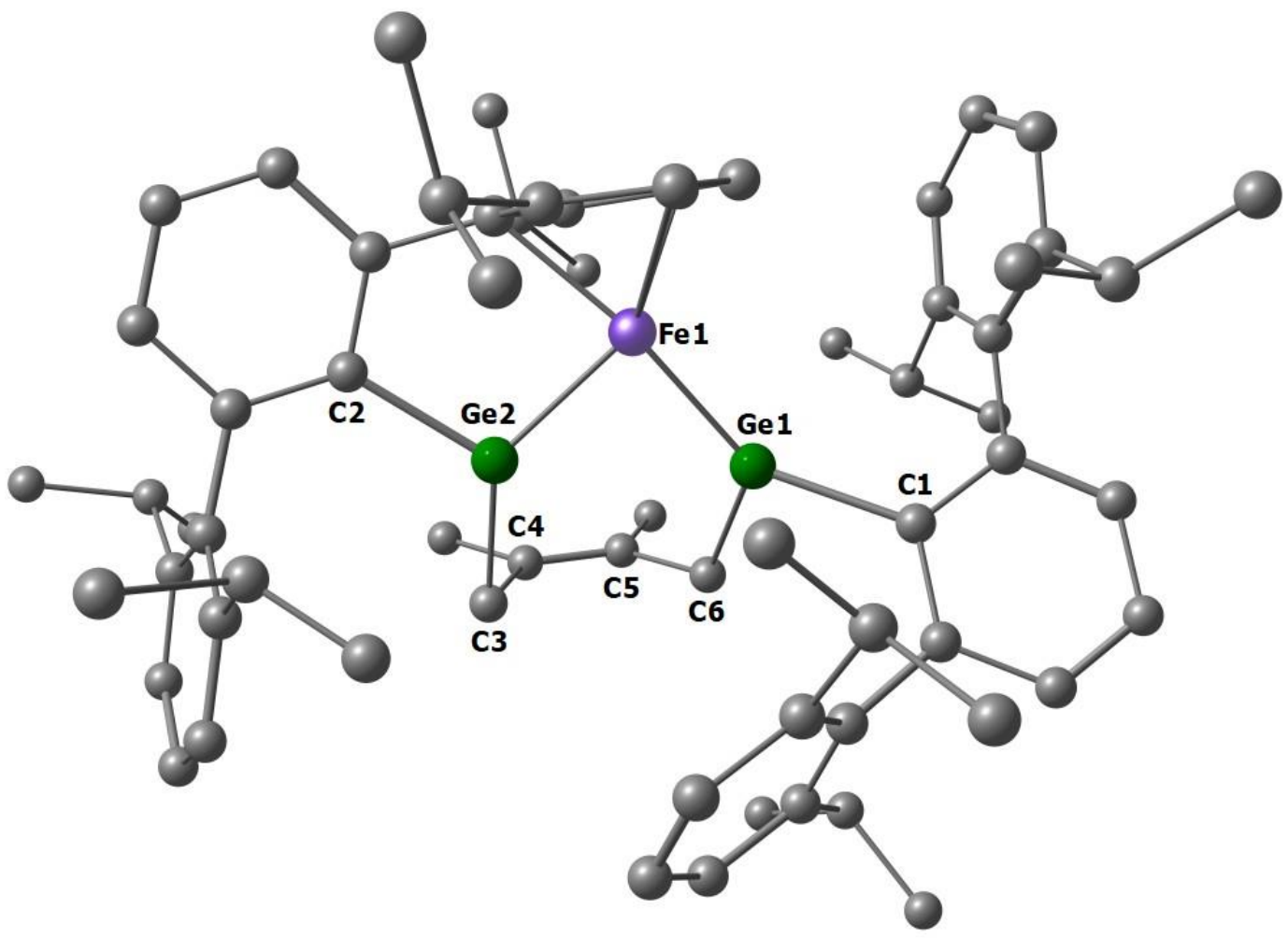

Figure S51. The optimized structure of **5** (BP86/def2-TZVP level). Hydrogen atoms are omitted for clarity.

Table S7. Comparison of the selected bond distances (Å) and bond angles (°) obtained from the crystal structure and the optimized geometries of **5**.

	**Exp.**	**Opt.**
Fe1–Ge1/Ge2	2.2260(5)/ 2.2150(5)	2.233/ 2.224
Ge1/Ge2–C_{ipso}	2.030(3)/ 1.990(3)	2.042/ 1.998
Ge1–C6/Ge2–C3	2.012(3)/ 1.987(3)	2.053/1.992
C4–C5	1.344(4)	1.361
Ge1···Ge2	3.0663(6)	3.100
C1–Ge1–C6	96.05(11)	94.88
C2–Ge2–C3	116.60(12)	115.80

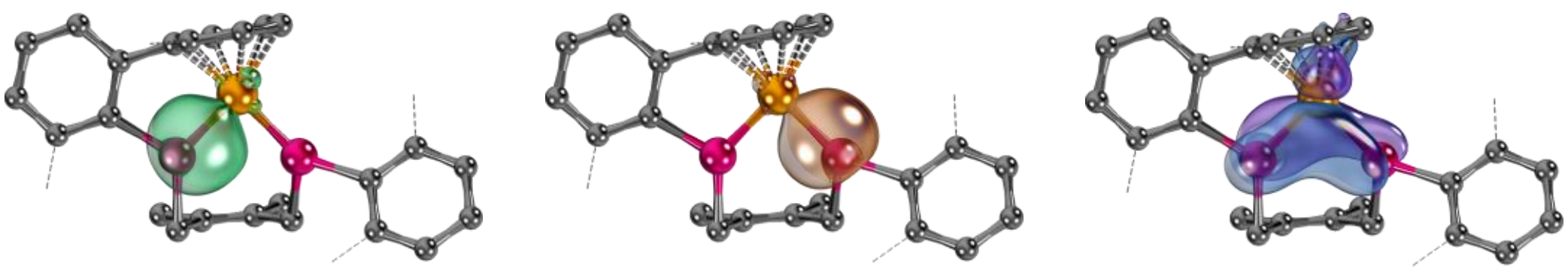

Fe1–Ge2 σ-bond
0.26 Fe1/0.78 Ge2

Fe1–Ge1 σ-bond
0.33 Fe1/0.70 Ge1

Ge2–Fe1–Ge1 π-bond
0.23 Ge2/0.57 Fe1/0.15 Ge1

Figure S52. Selected IBOs of **5**. The bond type and percentage of the electron density on Fe and Ge are given. The Dipp substituents and ^{i}Pr substituents of the η^6-Dipp are omitted for clarity.

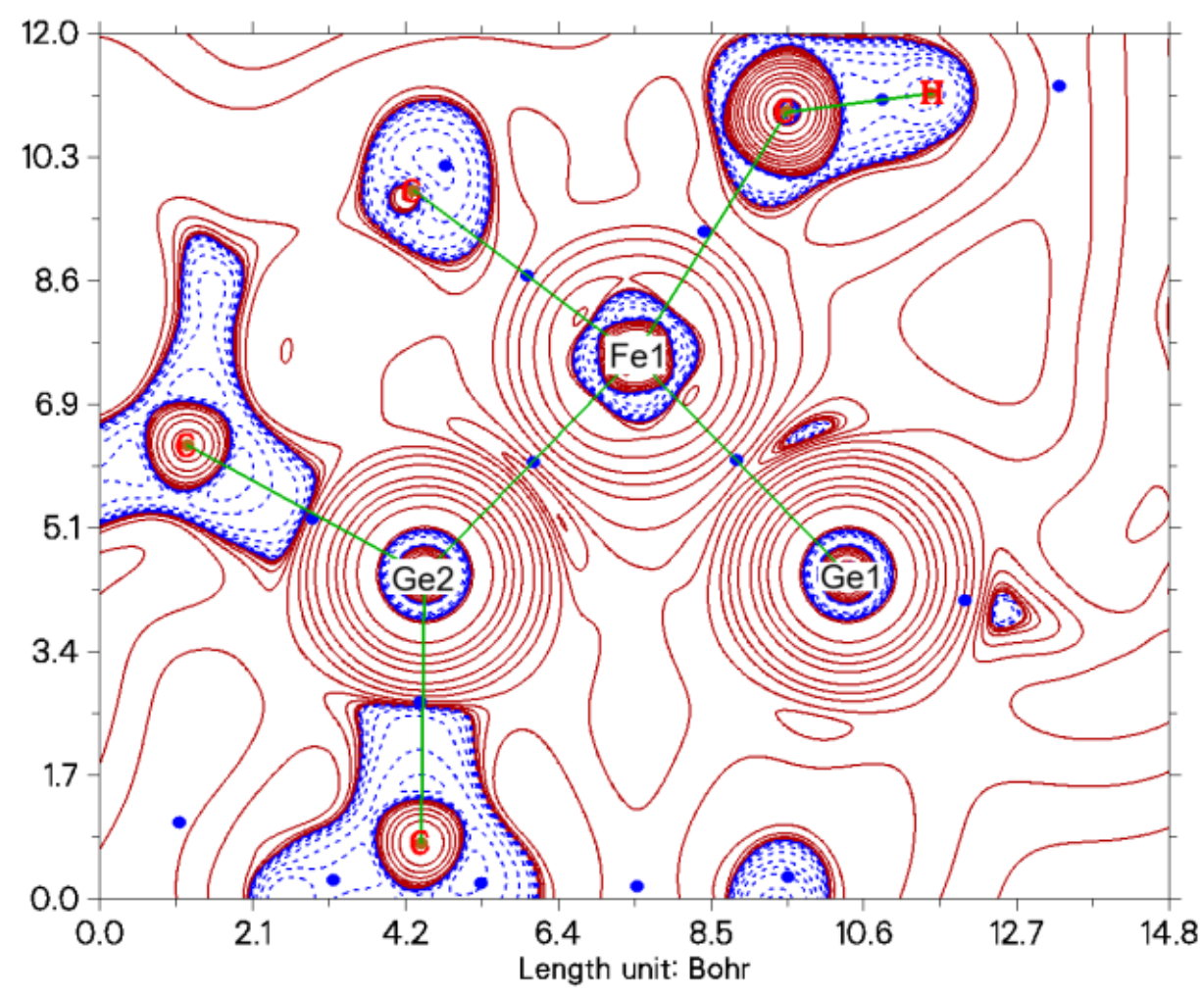

Figure S53. Plot of the Laplacian of the electron density on the $FeGe_2$ plane of **5** (BCP: blue dots, bond paths: green lines).

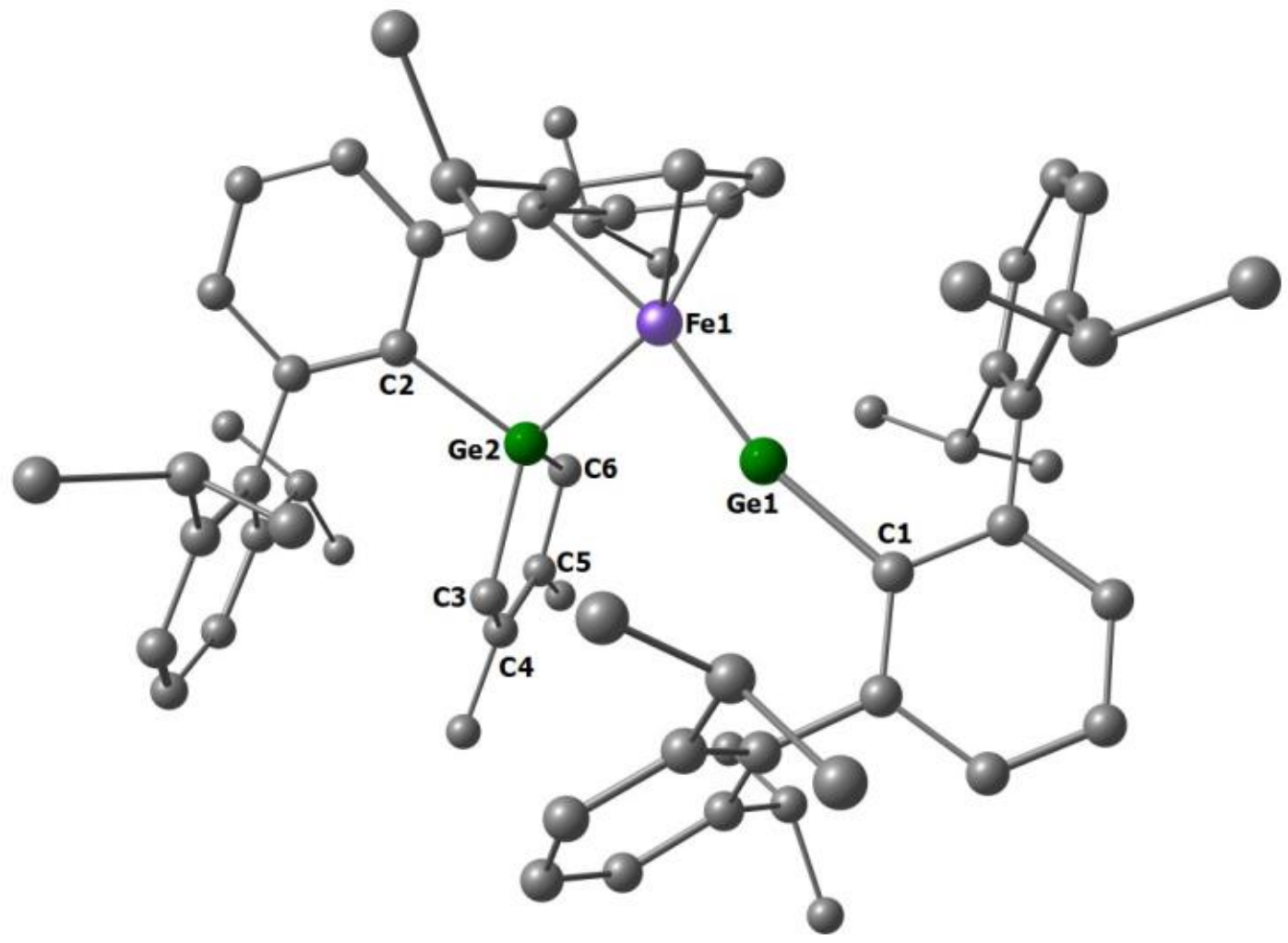

Figure S54. The optimized structure of **6** (BP86/def2-TZVP level). Hydrogen atoms are omitted for clarity.

Table S8. Comparison of the selected bond distances (Å) and bond angles (°) obtained from the crystal structure and the optimized geometries of **6**.

	**Exp.**	**Opt.**
Fe1–Ge1/Ge2	2.1069(2)/ 2.4021(2)	2.101/ 2.415
Ge1/Ge2–C_{ipso}	1.956(1)/ 2.010(1)	1.970/ 2.011
Ge2–C3/Ge2–C6	1.982(1)/ 1.993(1)	1.998/ 2.009
C4–C5	1.337(2)	1.354
Ge1···Ge2	3.2368(8)	3.216
Fe1–Ge1–C1	168.39(4)	167.75
Fe1–Ge2–C2	101.03(3)	100.09
C3–Ge2–C6	90.70(5)	90.02

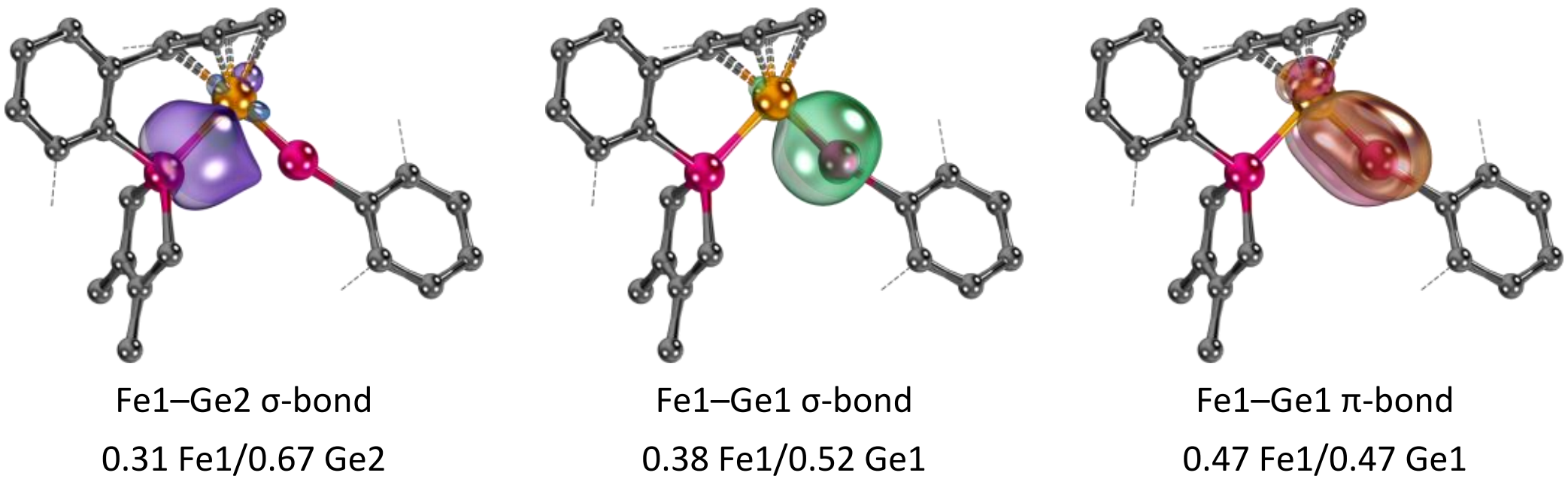

Figure S55. Selected IBOs of **6**. The bond type and percentage of the electron density on Fe and Ge are given. The Dipp substituents and ^{i}Pr substituents of the η^6-Dipp are omitted for clarity.

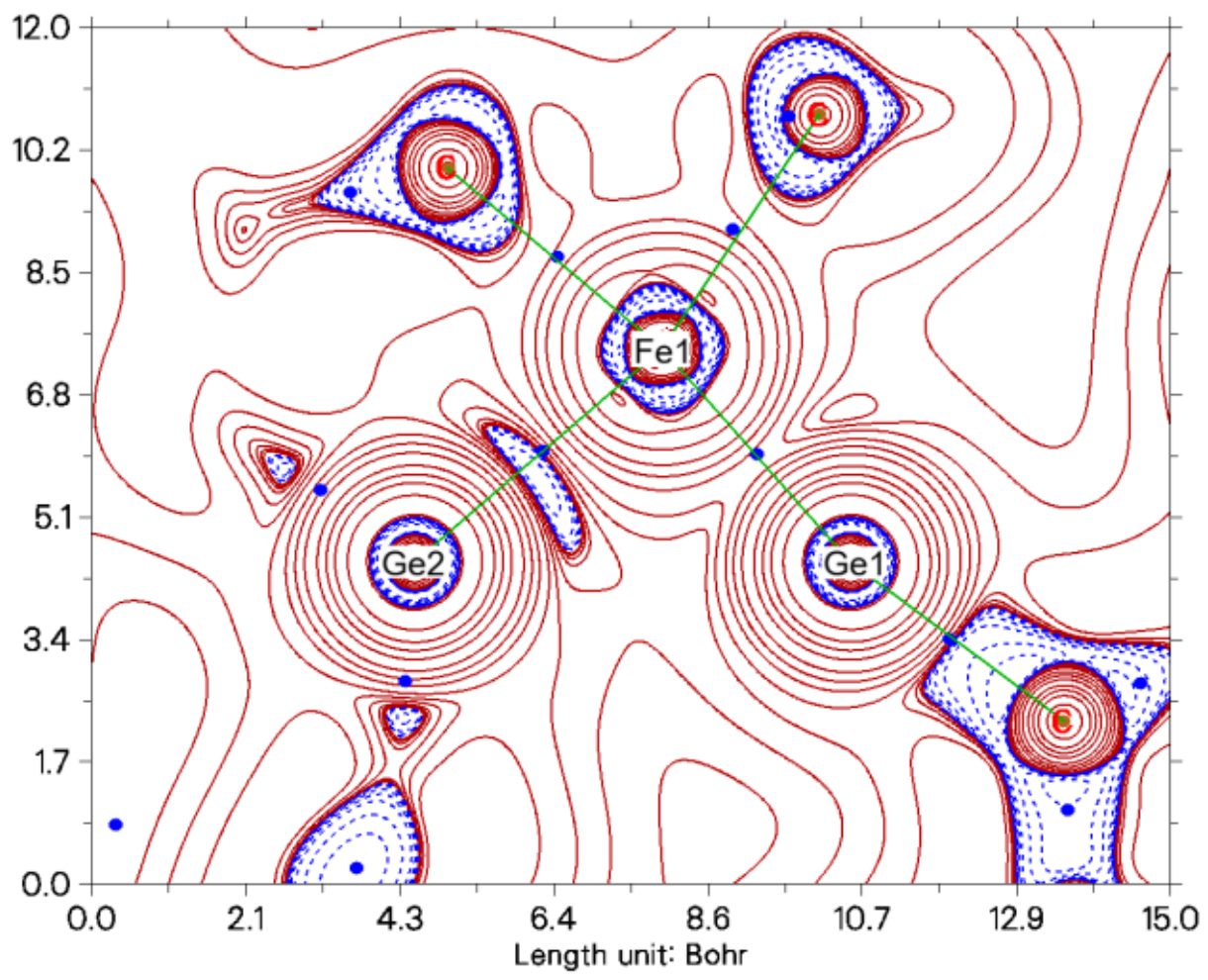

Figure S56. Plot of the Laplacian of the electron density on the $FeGe_2$ plane of **6** (BCP: blue dots, bond paths: green lines).

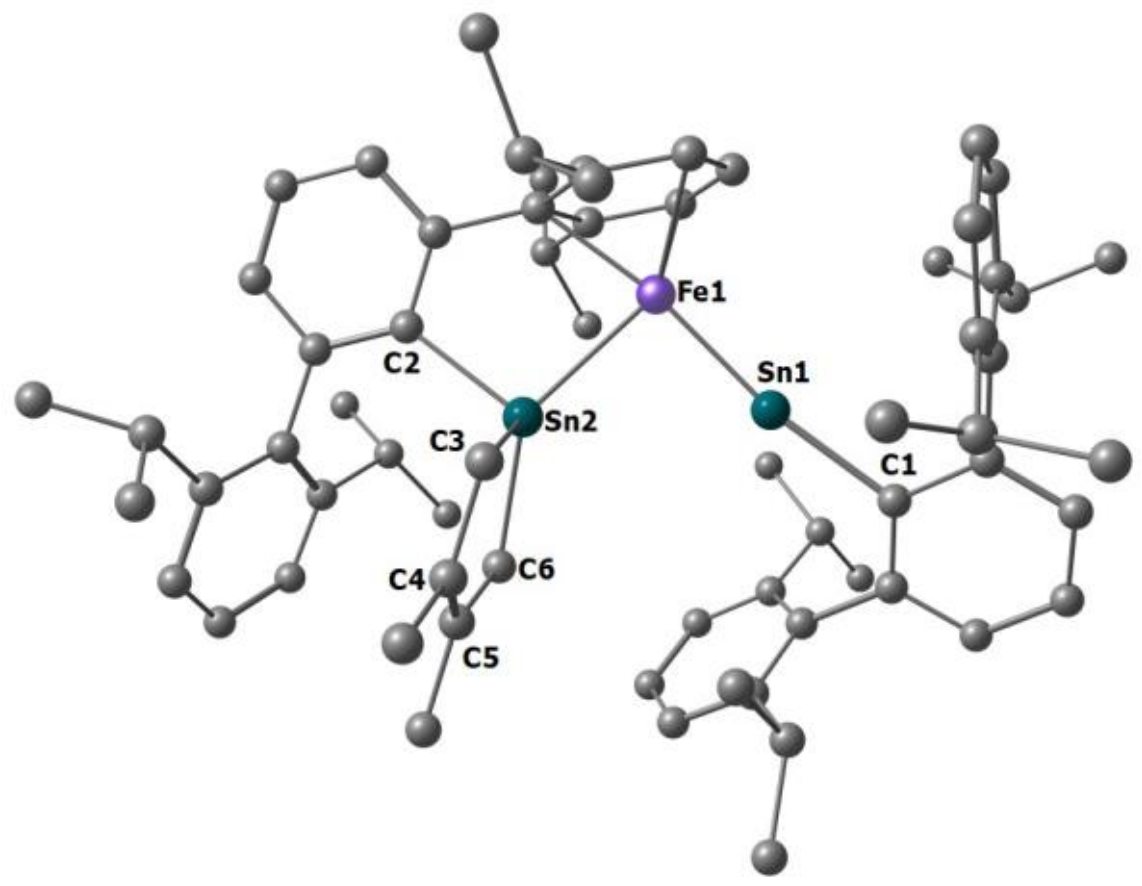

Figure S57. The optimized structure of **7** (BP86/def2-TZVP level). Hydrogen atoms are omitted for clarity.

Table S9. Comparison of the selected bond distances (Å) and bond angles (°) obtained from the crystal structure and the optimized geometries of **7**.

	**Exp.**	**Opt.**
Fe1–Sn1/Sn2	2.2904(3)/ 2.5778(3)	2.280/ 2.575
Sn1/Sn2–C_{ipso}	2.1428(16)/ 2.2016(15)	2.164/ 2.199
Sn2–C3/Sn2–C6	2.1849(17)/ 2.1648(16)	2.198/ 2.177
C4–C5	1.342(2)	1.357
Sn1···Sn2	3.4730(4)	3.327
Fe1–Sn1–C1	170.97(4)	170.98
Fe1–Sn2–C2	95.72(4)	94.00
C3–Sn2–C6	84.72(6)	84.58

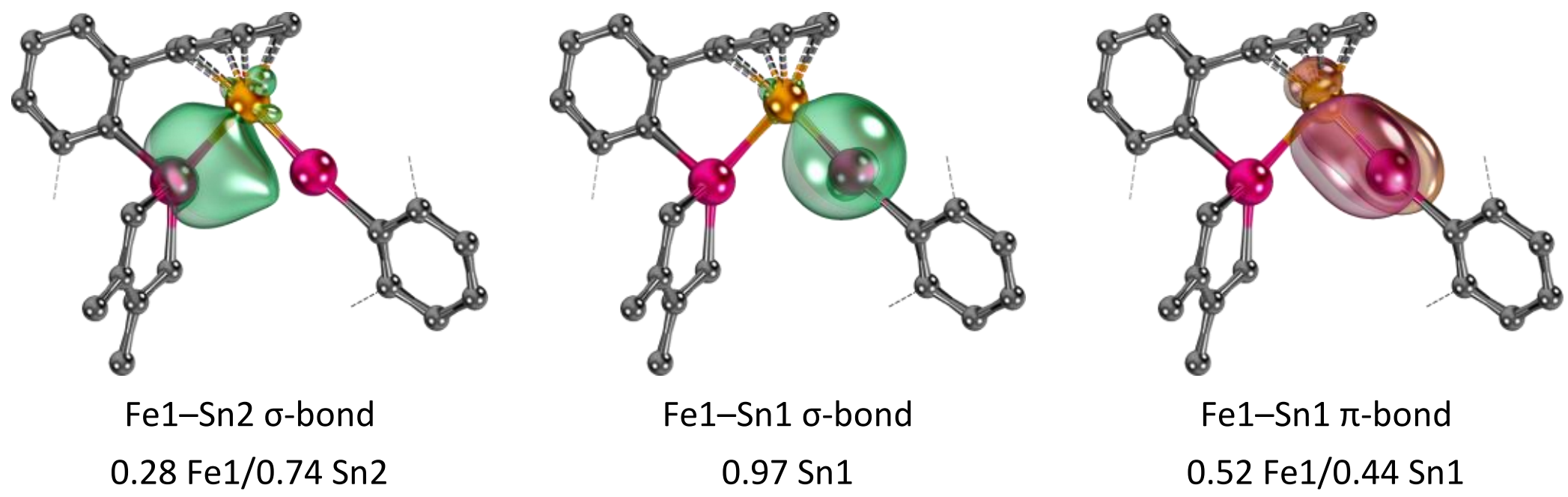

Figure S58. Selected IBOs of **7**. The bond type and percentage of the electron density on Fe and Sn are given. The Dipp substituents and ^{i}Pr substituents of the η^6-Dipp are omitted for clarity.

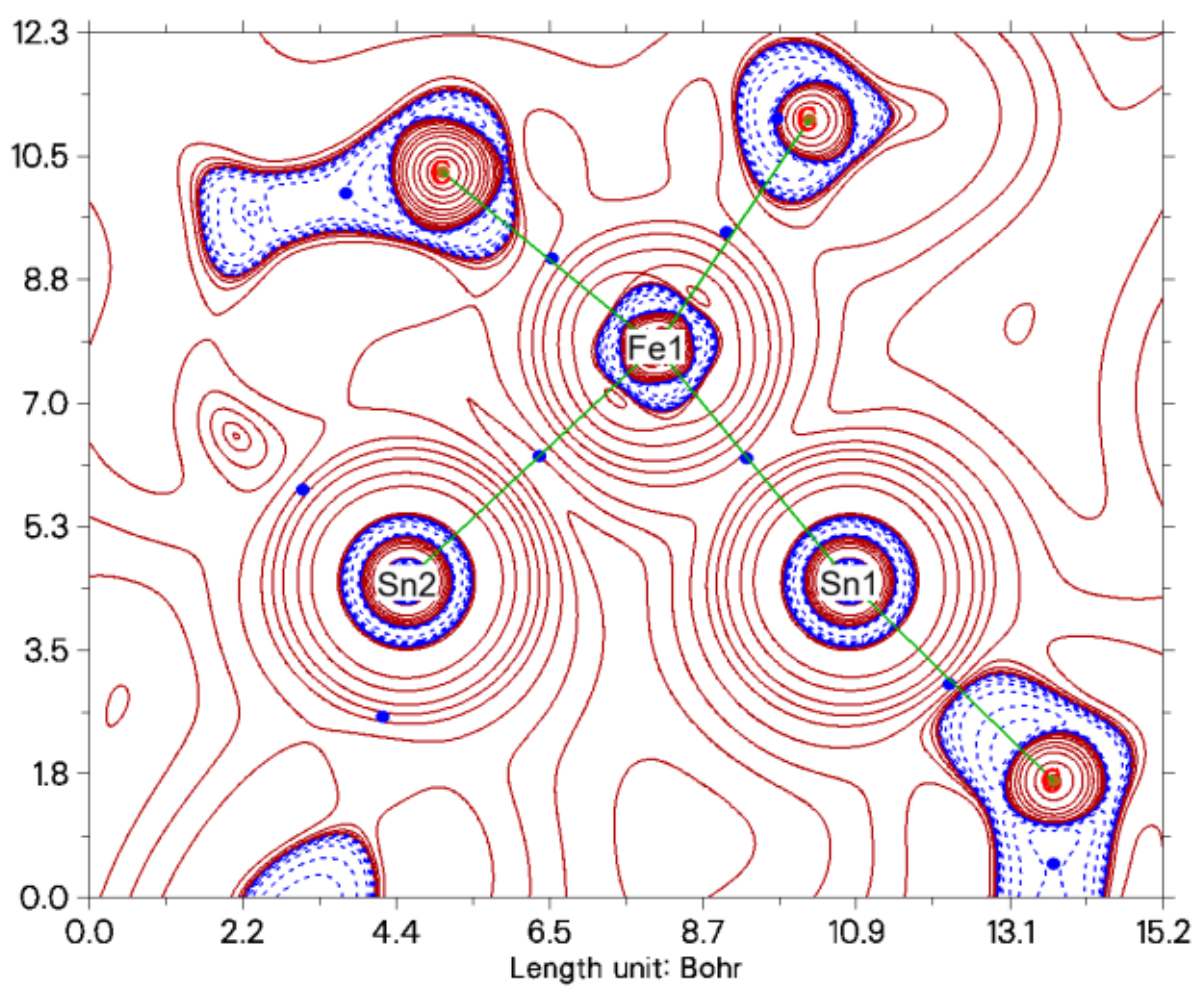

Figure S59. Plot of the Laplacian of the electron density on the $FeSn_2$ plane of **7** (BCP: blue dots, bond paths: green lines).

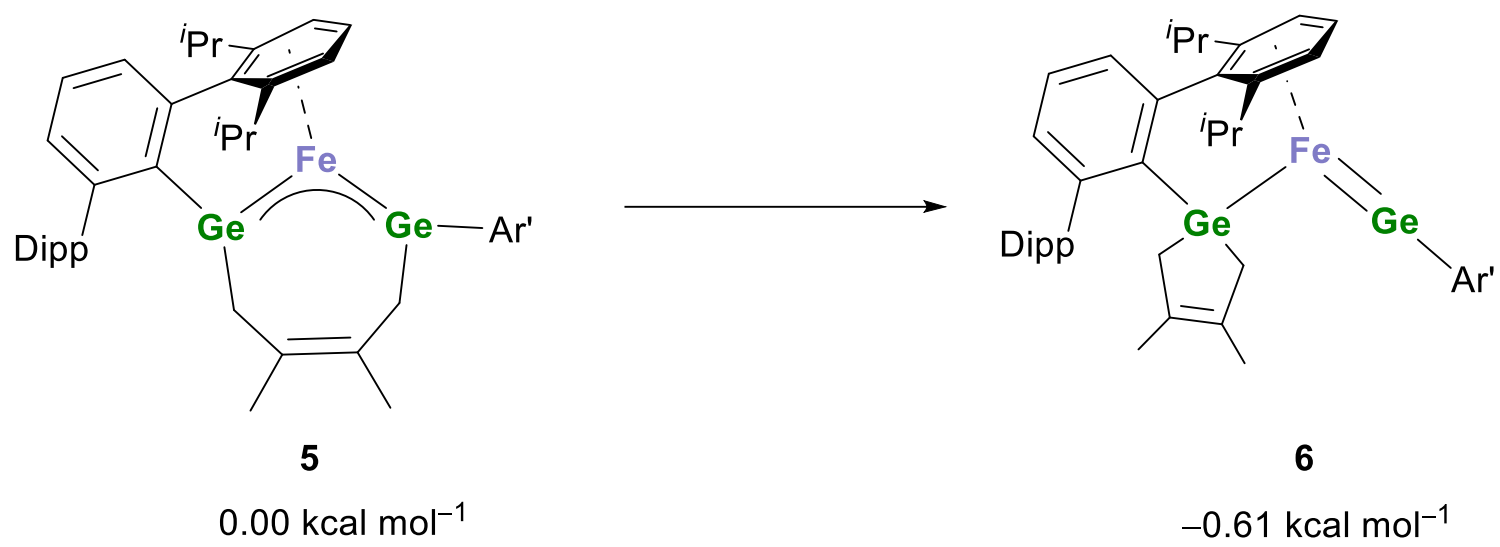

Figure S60. Relative Gibbs free energies (ΔG°, 298 K) of **5** and **6** (r^2SCAN-3c composite method[26]).

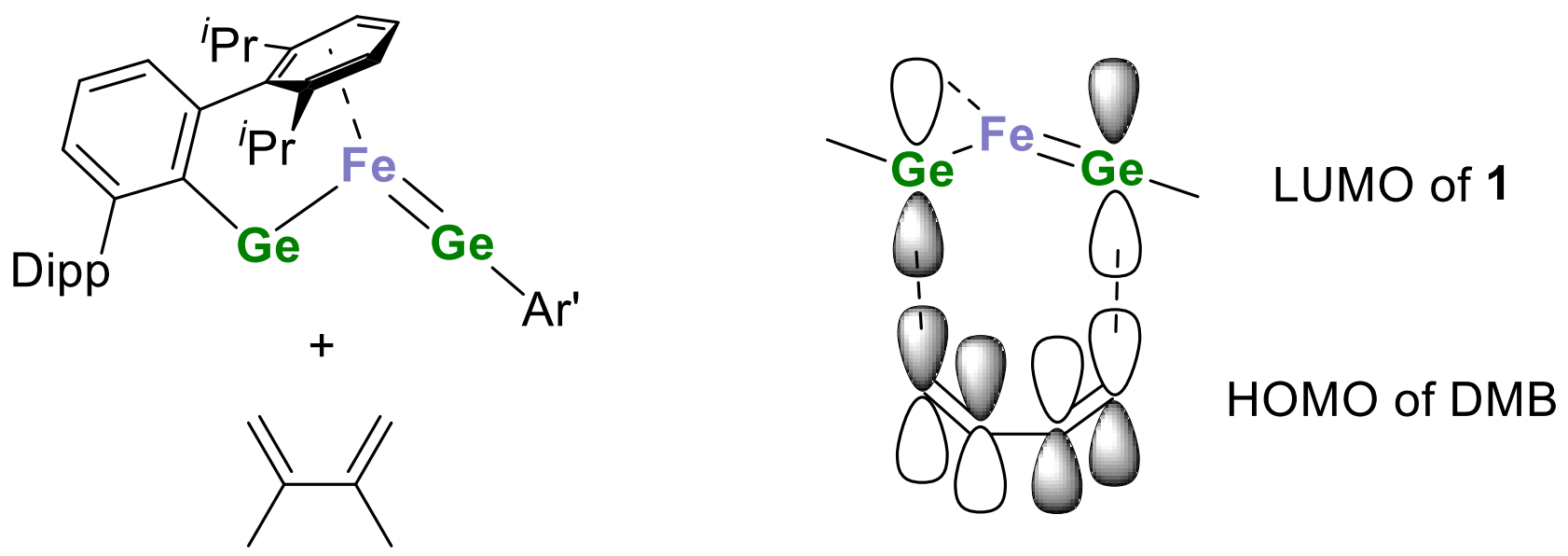

Figure S61. Interaction of the frontier orbitals between **1** and DMB.

Energy profile of the reaction of 1 with H_2: Using the full structure of complex **1**, the hydrogenation of **1** yielding **3** was found to be exergonic, with ΔG(298 K) = −22.1 kcal mol^{-1}. Two reaction pathways were identified, and their relative Gibbs free energies are illustrated in Figure S62. The cooperative dihydrogen activation pathway involving both the iron center and the ferriogermylene site (Ge2) is kinetically more favorable. In this route, the first transition state (**TS1**) shows the coordination of dihydrogen to the vacant 4p orbital of the Ge2 atom in **1**. Notably, the Fe center also interacts with the H_2 in **TS1**. This transition state features a short Ge2−H distance of 1.867 Å and an Fe···H separation of 2.149 Å. The imaginary mode of **TS1** involves H−H bond cleavage, Ge2−H bond formation, and motion of the second hydrogen atom toward Fe. Then, **TS1** relaxes to intermediate **INT**, bearing a Ge2−H (1.579 Å) bond and a Ge2···H−Fe (Ge···H: 1.843 Å and H−Fe: 1.571 Å) moiety. This intermediate is converted to the final product **3** via an Fe-to-Ge2 hydrogen migration process that involves **TS2**, which has a lower energy than **TS1**. This route thus suggests that the dihydrogen activation reaction is efficiently promoted by Fe−Ge cooperative action. The alternative pathway proceed via **TS**, which is 7.3 kcal mol^{-1} higher in energy than **TS1**. This route corresponds to oxidative addition of H_2 solely at the ferriogermylene Ge2 atom.

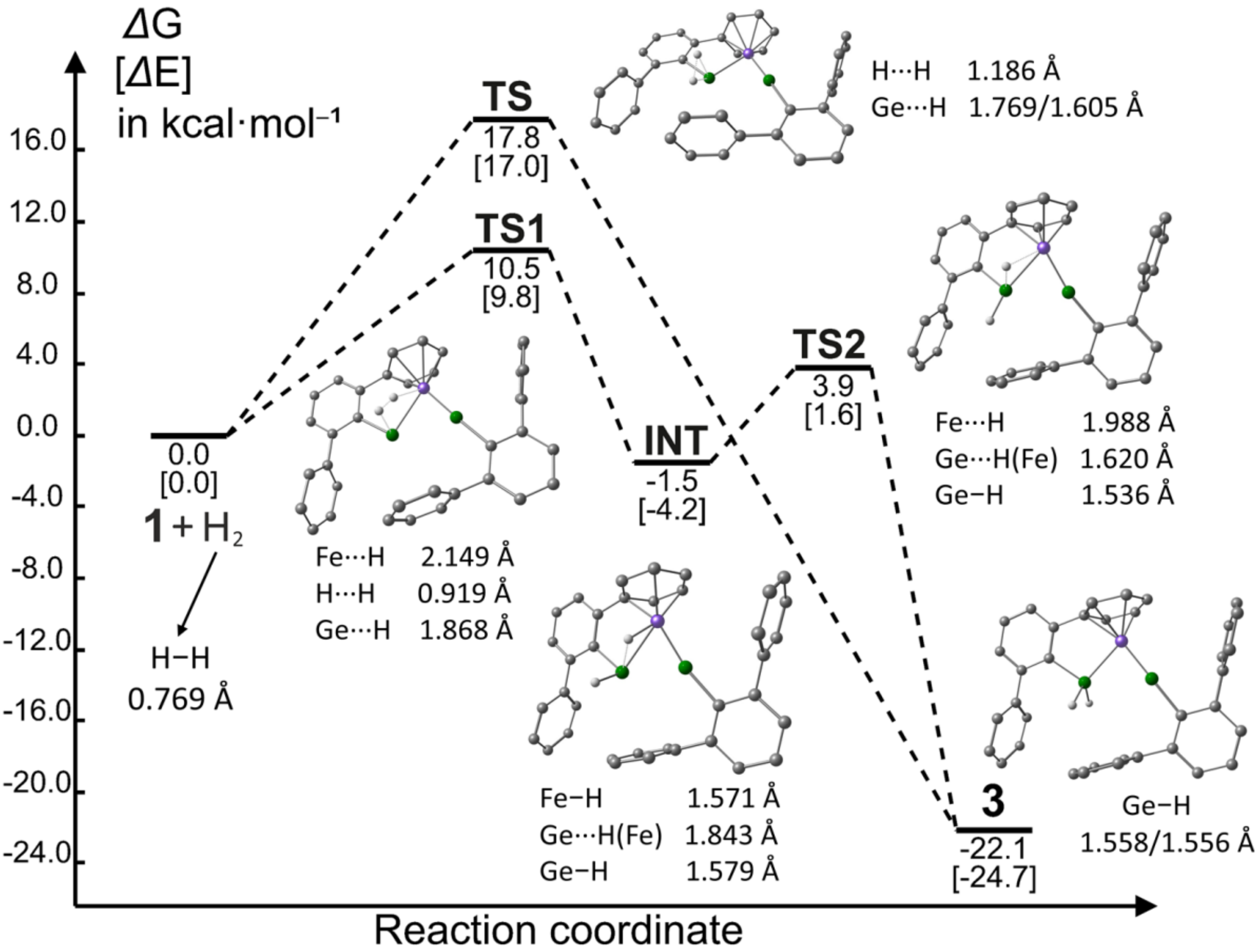

Figure S62. Computed energy profile for the reaction of **1** with H_2 at the BP86/def2-SVP(C, H)/def2-TZVP(Fe, Ge) level of theory. Key bond distances are given. ^{i}Pr substituents and non-hydride hydrogen atoms have been omitted for clarity. Color code: Fe, purple; Ge, green; C, gray; H, white.

Table S10. Cartesian coordinates of the optimized geometries of **1**–**7**, **TS**, **TS1**, **TS2**, and **INT**.

Complex 1

Fe	9.20294705204584	9.15459369108397	8.78445165848069
Ge	7.81002137675575	8.84703556735222	7.24232071298750
Ge	7.92434817080012	11.23306876119419	8.82672935366269
C	6.43646958462351	8.49191707772683	5.89551973312602
C	5.42224028965061	10.77703138460544	6.01915332129128
C	5.44818329038211	9.39474913240351	5.46570803409441
C	6.30190602802360	11.74210020136920	5.48224987420711
C	6.51461358622438	7.19434902278944	5.34737942937934
C	4.54356894436679	11.09879443905259	7.07979903045983
C	5.59268257868153	6.79900706067585	4.37464410210437
H	5.65550630439920	5.79489445659784	3.95170610059236
C	4.53168993011580	8.97686758807323	4.49098897705377
H	3.76603955649807	9.67573003706454	4.14948779279086
C	4.60164023691940	7.69017653341235	3.95125272234156
H	3.88321645260615	7.38190549431295	3.19029107714821
C	7.60177795162152	6.28816031471012	5.83274918986485
C	8.88350356624948	6.35262625741269	5.23556621218827
C	7.23733166578908	11.41667869457230	4.32700225420082
H	7.13977854040576	10.34393870988971	4.10540234440053
C	7.35543249454193	5.42374240747637	6.92444346074332
C	3.63940663092998	10.03406359249220	7.68571119666034
H	3.43633078816717	9.29067468139000	6.89879627009999
C	6.00881234992175	5.40001569475312	7.62884976926115
H	5.36822351716647	6.15233820978503	7.14626473032306
C	6.28104242524430	13.03557750638166	6.01724818332433
H	6.95277548898452	13.79581274038810	5.61638238154366
C	9.88981292988595	5.50433258741577	5.71388368619349
H	10.88118123416831	5.53653036346084	5.25918487667513
C	9.18257185317717	7.31505915683674	4.09679196348453
H	8.30730538682395	7.97052635896203	3.97730392149259
C	4.56146247763525	12.40288160477490	7.58402217437478
H	3.90267878239898	12.67421879983480	8.40829474492278
C	8.39071786401232	4.59460867254534	7.37273499109544
H	8.21296406184015	3.92078924120878	8.21253831611779
C	5.42203272503651	13.36450803509465	7.05945008110667
H	5.43783461998671	14.36670325666864	7.48395167503268
C	9.64459537737964	4.62345229622931	6.76543279133023
H	10.43945715843458	3.96659461500725	7.12317367351075
C	2.28819330172224	10.58495699409780	8.15854128186806
H	2.40467398303636	11.25420453604281	9.02360486552039
H	1.63170305055715	9.76035262575896	8.47194577384754
H	1.78111435210668	11.14636147291456	7.36119713689812
C	4.35144950685786	9.29810346703367	8.83379893049273
H	5.30299386197033	8.85872671716473	8.50381137212408
H	3.72045100545592	8.48837714891155	9.23021884309064
H	4.57889298687664	9.99225186491846	9.65478022126142
C	9.37317464326544	6.55679530825610	2.77365674159128
H	10.23107846904480	5.87018020540867	2.83235726136417
H	9.55621556014900	7.25911348362055	1.94718075080946
H	8.48245084394498	5.96105857134241	2.52886250009127
C	8.70509871195632	11.67867488863776	4.69158118038079
H	8.88673166709222	12.74566080242592	4.88345010834429
H	9.37062904260944	11.36519014688144	3.87413890834691
H	8.98807546984142	11.12518604970641	5.59934021513656
C	10.38808332627748	8.21198954583971	4.41589743972932
H	10.23452337995801	8.75221220605184	5.36169944113875
H	10.53982629710731	8.95188673267256	3.61702638238561
H	11.31464211880218	7.62573662571570	4.50679330930750
C	6.83093461991932	12.18394823385740	3.05880802806186
H	5.79258324213275	11.95906223869421	2.77652024153521
H	7.48438135099689	11.91431643251206	2.21560627007097

H	6.90906019946148	13.27036713661771	3.21381339004825
C	5.31874935738620	4.03625968522001	7.47295037509287
H	5.18455959497380	3.77961479288692	6.41262096791566
H	4.32920023414484	4.04729556499979	7.95282705430791
H	5.91117239734970	3.23547712365446	7.94033316052395
C	6.15285320405560	5.79745045879614	9.10640288018083
H	6.75650555746709	5.06169802442070	9.65956771258079
H	5.16722847555017	5.85552161032107	9.59010733562570
H	6.64331751480585	6.77688832166814	9.20108358039324
C	9.05451150075498	12.08518404675515	10.32072406709099
C	10.26875377754140	9.95244532078017	10.34831470233046
C	10.06197418645016	11.30637599671372	10.91983052551589
C	9.44936155090283	8.86624690576695	10.81805812890964
C	8.83453057261070	13.39003752728845	10.80551660067910
C	11.12720946420079	9.80203041297028	9.20318742999392
C	9.60392738086256	13.87058971508215	11.87935973328086
H	9.42555949481963	14.88401950052707	12.24522829926685
C	10.82116444442867	11.78518525012471	11.99066180108302
H	11.59070959261902	11.15445689202574	12.44013570041723
C	10.58492505285712	13.07516807539121	12.47311622788202
H	11.16971030885328	13.46418207926974	13.30811333970883
C	7.81817108440890	14.30503708660078	10.20047022058229
C	6.50984860343747	14.34776450512254	10.72507353266347
C	8.50782310780975	9.04729444361196	11.99421808766977
H	8.34935955627205	10.12944817864848	12.11183073152047
C	8.20690774858795	15.17341298019873	9.15554697478306
C	11.93608564154216	10.97318144772140	8.67862142540739
H	11.41758026730080	11.88681614030931	9.00537634387819
C	9.60377724965113	15.08046732833522	8.55912943848604
H	10.23970520510442	14.57780673965991	9.30295925155740
C	9.55858048141397	7.60933162175647	10.15789646096368
H	8.89323502387193	6.79463031963544	10.43872460737783
C	5.60224898814441	15.27758708641636	10.20210127862975
H	4.58676930969563	15.32209470514472	10.59981381980990
C	6.07864001322257	13.38429455626101	11.81903750663416
H	6.98541616054964	12.87894132554291	12.18282172525724
C	11.20431735170342	8.52066603349925	8.58764628856106
H	11.79219130824635	8.39579127323560	7.67968650305996
C	7.27323982761621	16.09134355196052	8.66077392036077
H	7.55802046646556	16.77401829351415	7.85880022209072
C	10.44640072929120	7.42922452530487	9.07123315572293
H	10.43419883928872	6.49275881031506	8.51685821756611
C	5.98180529598572	16.15179174198584	9.18533625670734
H	5.26737174184783	16.87825346295898	8.79407233208662
C	13.33170223813754	10.97304249142221	9.32457241446446
H	13.88525998718305	10.05905190217396	9.05979813989358
H	13.91874329001652	11.83882425500036	8.98446605160658
H	13.25732541709899	11.01962213130229	10.41978203402583
C	12.02408197690977	11.01165633361161	7.15101886971642
H	11.02317790717791	10.95666940515220	6.70127351250367
H	12.50260033471671	11.94591425613095	6.82435653483100
H	12.62552810984578	10.18170909435134	6.75075948879306
C	5.43575988385877	14.09651700857721	13.01666149329915
H	4.49346643981437	14.59085029033445	12.73708119788705
H	5.20589169616790	13.37362248623151	13.81334541209013
H	6.10783529033571	14.86182528654088	13.42998088846652
C	7.13540451352698	8.40318714913130	11.77678025056083
H	7.19618830027650	7.30575459598645	11.73320893981990
H	6.46124145673584	8.66058807869901	12.60623042978916
H	6.68826665883092	8.75626801388174	10.83747557489153
C	5.15411114295703	12.30154269208736	11.24320556922698
H	5.62998288804087	11.80594971291321	10.38187355314368
H	4.91681590453795	11.54114418264936	12.00219084177181
H	4.20764677841801	12.73957447297272	10.89017816091565

C	9.17988351195162	8.52853885206136	13.27597155430898
H	10.13636382607779	9.03925938358018	13.45605698370046
H	8.53345322357902	8.69341508439347	14.15039133105711
H	9.38263554009299	7.44921070895579	13.19975478510321
C	10.23470656711995	16.44694676055056	8.26568634142631
H	10.23576599751032	17.08650629277794	9.15966439390244
H	11.27522095098407	16.32040249544565	7.93249854102105
H	9.70016875360367	16.98288760146349	7.46724501389590
C	9.58696195381127	14.19760922259487	7.30216126496003
H	9.00104981518754	14.67550578789621	6.50268170048316
H	10.60496704514503	14.01925847283298	6.92582814073989
H	9.11699467321737	13.22211235580315	7.50713426643246

Complex 2

Fe	2.46073707953936	16.13159830340444	11.67902365374788
Ge	3.93595206996365	15.04468614446963	13.01775155847151
Ge	1.91603865635006	14.48060223976480	14.20983501771894
N	3.27202389240873	18.71255018975607	12.93750464491317
C	2.75496672094552	15.73210892986552	9.64669510347852
H	3.69810947569153	15.66460705813046	9.08884714762715
C	0.85693081609808	13.17955404008904	13.05410760077234
C	6.87100969532992	15.70248026901474	11.83557806079563
C	7.40421358337291	14.38063879734089	11.82223384490265
C	6.59283285778179	16.32378734989122	13.18177785366831
C	6.70661569581502	16.40819560154278	10.60748004945863
C	0.32615222621530	12.01983453077041	13.67949230899982
C	-0.57642444070190	11.18790711907656	12.98718287933898
H	-0.97836839762873	10.29566342988855	13.49308838847416
C	2.20314148202164	14.55539752789105	10.25045392251310
C	4.33622617094571	15.76414226553012	16.25730690049646
C	7.60216400994308	17.10541945308345	13.78041794727769
H	8.52252856635197	17.31639118958859	13.21508694724483
C	0.46632181534676	13.46722283567594	11.72686121014086
C	7.76568085315833	13.79112853076248	10.59429835931923
H	8.18081079454704	12.77177887810000	10.58720681129992
C	3.26542033732306	16.55338658437136	16.74698297347271
C	7.46045470954556	17.57601799120911	15.09503435128151
H	8.24326171568870	18.20655977933681	15.54342567564646
C	0.39050716458074	15.96611001583933	11.27093782965053
C	7.64640128759566	13.59096746853116	13.10256905421986
H	7.19807070977058	14.16022779799737	13.93804821469257
C	5.35500399069564	16.34261684291918	15.31127049626243
C	-0.43915950942993	12.63216520913893	11.04315549836550
H	-0.73989414091918	12.88573429633320	10.01458069393612
C	3.01940508702332	17.63739575798834	12.45494892365195
C	5.43036762290280	15.98760045333785	13.93524415590312
C	2.99087725728351	17.95726694131039	16.22792068313854
H	3.63800445775092	18.11255715160625	15.34158056433042
C	1.01629894833584	14.67949379747458	11.04894165342473
C	-0.83996987133989	16.09360209643112	12.15852802394157
H	-0.74852419497997	15.30353423496905	12.93310354362253
C	-0.96569534476976	11.49451671236185	11.67398459015154
H	-1.68271345999803	10.84960164518081	11.14360246909052
C	2.09120977589517	16.97963245935879	9.77607938444865
H	2.53832512805475	17.88391220490482	9.34165260321827
C	7.60713339124045	14.48096105263500	9.39079422376290
H	7.89201708705906	14.00981425411171	8.43743260846952
C	6.35378931546639	17.17951897420063	15.85769971148451
H	6.27864867988807	17.47023836079410	16.91702059958328
C	0.91878142130567	17.09102826579398	10.57692725204536
H	0.49087593126475	18.08675358710025	10.75478227420111
C	2.86786842182790	13.19597004532713	10.06155553604895
H	2.61449188628039	12.59104389859815	10.95678090336857

C	0.72937879865611	11.68810842000372	15.08235817406891
C	4.49540794994428	14.41937305267396	16.71900489531327
C	0.10004178150253	12.36423558948852	16.16290525879120
C	7.07593742849875	15.77688344767897	9.40414149672236
H	6.95087509358318	16.31136261941528	8.45226171884582
C	6.17567995462029	17.83680178535362	10.57037330376716
H	5.43763530616987	17.91764976006483	11.39712868497756
C	1.72474220350907	10.69663866632531	15.31518253216462
C	2.40039412589705	16.02042888356272	17.72443506901656
H	1.57966330342595	16.64112752443909	18.11387708418485
C	3.80064095268467	20.05237409642486	13.03542133386846
C	-2.12142444578560	15.81161781926984	11.35080123194222
H	-3.01740541904355	15.87738061918419	12.00183822473768
H	-2.10289055048867	14.79991792998166	10.90096833343681
H	-2.23986073708444	16.55022080410033	10.53026427880213
C	0.48505736500099	12.04052155898590	17.47796998623268
H	-0.00169488269708	12.54595656178920	18.32505470514164
C	4.39502367561889	13.25029579936902	9.95010554676899
H	4.73381041281704	13.75934983181104	9.02566189127053
H	4.81686616097566	12.22599117193375	9.93038980200823
H	4.85391709527500	13.78448397539359	10.80630523545998
C	5.62058438406736	13.52238676371068	16.20707928296115
H	5.65244516688661	13.66119587424286	15.10531612743678
C	1.47075625208876	11.07684233287518	17.71880776558800
H	1.76224999665573	10.83334389619290	18.75245271278179
C	2.08247552521737	10.41040044733948	16.64655152540597
H	2.84499471628670	9.64697710027145	16.85545000126220
C	2.37574219428887	9.96677245231812	14.13794459379668
H	1.57246654160037	9.79806099804906	13.38982834494659
C	5.45991713791745	18.17342881986887	9.25217593483544
H	6.17595189204272	18.28053804192736	8.41159580084236
H	4.92128987345428	19.13796089811906	9.33292330866759
H	4.72687626247328	17.39454982960062	8.97671547252495
C	3.58389261410650	13.91680425470705	17.66428422826366
H	3.67037862399377	12.87774804743120	18.00487039124545
C	2.26733695401157	12.50107366703426	8.82319191127804
H	1.17087992006401	12.38061333487319	8.91005765506691
H	2.71149850184791	11.49394825709337	8.68579132038272
H	2.47365652229876	13.09351116535193	7.90698209078913
C	2.55382026949716	14.71210633752231	18.18107992207254
H	1.85994230104362	14.29661744027704	18.92619121541498
C	3.35147645619067	19.01874189002808	17.28319256003458
H	3.14680593671997	20.04504555418904	16.91285011573860
H	4.42386000968741	18.96493853845339	17.55936810248803
H	2.75940456517601	18.87549326299885	18.21102857988121
C	2.85275292210927	20.91642181157449	13.89195120151762
H	1.82732601217897	20.90893629969222	13.47269782985450
H	3.21726942637297	21.96258687877616	13.91268151477996
H	2.81017757393637	20.54466534155425	14.93129901498100
C	-1.03714203337685	13.35141552230364	15.90955726574883
H	-0.95435275749713	13.68525599006035	14.85463832088279
C	5.19515935404651	19.98866491584645	13.68976572438572
H	5.13546708014245	19.54478132156283	14.70147079249898
H	5.62377539724880	21.00707909867253	13.77515653634933
H	5.88361711548699	19.36233368017596	13.09447174870075
C	5.37375194379858	12.03145315296232	16.46715033931984
H	5.45193813850762	11.78283891834628	17.54637905753464
H	6.13478331468084	11.42126519287639	15.94076213195494
H	4.37295403776051	11.71423585593627	16.11954182311037
C	9.15274092204065	13.47491443590304	13.39758395859758
H	9.68337715949093	12.92148511309077	12.59461586050415
H	9.32664762981746	12.93797791578517	14.35308604661371
H	9.61660346119598	14.47856672885450	13.47965071691419
C	7.30643761923495	18.85954276806333	10.80499871884431

H	7.79338862830605	18.72544966519613	11.78797484958485
H	6.91649895187340	19.89748325781039	10.75960201630090
H	8.08582193830085	18.75493526957014	10.02180712571361
C	1.53174776330800	18.08319883225116	15.75698787701298
H	0.81576141421366	17.99395871459345	16.59969154773496
H	1.29956273350123	17.28356948003682	15.02551097566518
H	1.34731147380792	19.05811493450830	15.26669913137164
C	2.95126214560584	8.59226008144415	14.50922322157354
H	3.83512590966402	8.68354941357627	15.17475421271272
H	3.28867022630322	8.06067592970843	13.59631503073557
H	2.20255020571868	7.95422525629770	15.02129557770055
C	6.99391481646794	13.92962810049599	16.77631111822514
H	7.26758405401957	14.96655521899184	16.50882099808949
H	7.78940522965472	13.25995757830574	16.38899589001189
H	6.99259300264302	13.84973957123464	17.88352483333080
C	6.95327856067084	12.22050918112218	13.05881561483050
H	5.86320085750829	12.33601629767129	12.89287171415845
H	7.09611372871426	11.67656534298011	14.01363233922978
H	7.35191625998335	11.57553977542268	12.24880664426786
C	-0.91975519303296	17.44444929382484	12.88097420090075
H	-1.15570329717218	18.27454330751346	12.18236800785238
H	0.03651903253364	17.67957597966257	13.38583032502647
H	-1.72258941972176	17.42498505766704	13.64490723454572
C	3.88609381398384	20.65580556399912	11.61848096879844
H	4.56318627123317	20.06051807049244	10.98168614956877
H	4.27313527055230	21.69310848809875	11.66786376941115
H	2.88529306080701	20.67571748782510	11.14302211188856
C	-2.39441624176478	12.63512767686636	16.04804080832566
H	-2.53860403540874	12.25432919480189	17.08106558514642
H	-2.46016430781373	11.77029181120164	15.35747851759852
H	-3.23248776164531	13.32527312279036	15.81655781769059
C	-0.95871937742435	14.60998413240201	16.78520390606727
H	-1.75346066232891	15.32870100672677	16.49788021535673
H	0.02251468320547	15.11062873317073	16.66326029212798
H	-1.09768617712700	14.38117493313828	17.86266939117645
C	3.45084299811954	10.83048231422095	13.44784995829964
H	3.06363989075374	11.82747932578956	13.16219375678090
H	3.82509178122576	10.32917489314021	12.53140573437352
H	4.31194183404557	10.99470307887269	14.12312247440327

Complex 3

Fe	6.42733980787412	11.49636371779398	7.45752032215033
Ge	7.58086070093436	9.38425096890828	7.37350834380611
Ge	7.77181937837965	11.81421602441391	9.02032051444723
C	6.55684641618715	8.51518195889107	5.90785657927078
C	6.79234667049998	7.21215144874630	5.42194012214414
C	5.34871050223538	10.67680695391449	5.87956578480302
C	4.47790313847080	10.85620234830470	7.01693367608890
C	5.99840523432470	6.71510322400675	4.36618229580840
H	6.18074891530680	5.69466816781004	3.99464818605499
C	5.53927154715646	9.30079041930550	5.33098738576326
C	4.98420283222149	7.50273595345862	3.79880201743636
H	4.37172457039093	7.10035799634146	2.97743251341462
C	4.75185516234579	8.80137076161014	4.27788412330421
H	3.95844798849007	9.42762414040495	3.84188135287735
C	7.17793513199971	11.49416228491246	4.24432970834211
H	7.30117335992916	10.39585720032398	4.15103789982905
C	6.21816301599223	11.73248685224081	5.40210614930878
C	9.19457677917266	6.45416469958962	5.53315183550352
C	7.52757606673287	5.42740055723588	7.03527753249846
C	7.86270277503954	6.35091384953644	6.01373732984511
C	6.12002161705282	5.37959851077615	7.61980463731522
H	5.55401778773287	6.22453407259849	7.17777469283836

C	3.62332907295717	9.71673132135879	7.55906488680859
H	3.84950341367961	8.82160167961501	6.94472103153094
C	6.15069576151612	12.99983307416507	6.05752351942671
H	6.84249050571615	13.80313838784500	5.77098340771566
C	4.46441295629763	12.13800293111412	7.64830737982449
H	3.87707796630983	12.28900413773109	8.56533987176815
C	9.55397668603023	7.48600840888270	4.46924534727660
H	8.78566643511441	8.28428185465263	4.52717237233222
C	8.52223509534654	4.54886916959878	7.50618155578186
H	8.27267667180532	3.81614619349335	8.28922084723152
C	10.16215825811669	5.56288736902590	6.03257183986411
H	11.19594800832115	5.61797676358868	5.65992996059630
C	5.26644791252816	13.20741608102860	7.15528094905394
H	5.32436084812187	14.14526976987766	7.72184391684837
C	2.12827033053471	10.03716182188704	7.37781785896214
H	1.82822451694931	10.91831907210764	7.98321311703657
H	1.49856946232194	9.18065225457742	7.69521112772363
H	1.89154764348880	10.26265830313314	6.31818804761198
C	9.82548917288801	4.60052102626602	6.99514316713656
H	10.59128666628061	3.90120977306321	7.36526137194968
C	3.96944673734064	9.36972994400509	9.01522892266358
H	5.02782599814076	9.05754822947020	9.09866622375186
H	3.32894190940837	8.54350466488958	9.38666566067930
H	3.82747091167827	10.23815782068228	9.69057719007594
C	9.47241237637037	6.86825223523403	3.06043101274781
H	10.19446065182414	6.03155623434838	2.95347462459734
H	9.70463994458264	7.62382557751775	2.28098576461420
H	8.45903764453755	6.46829412593932	2.85761433824242
C	6.54993873241054	12.00146447329183	2.93320538445735
H	5.57224369702445	11.51389321808398	2.74439721073507
H	7.21057044662379	11.79092223691013	2.06702601814608
H	6.37795344041829	13.09768565772040	2.97487765613992
C	8.57256145257307	12.08680134625383	4.48698160757799
H	8.55597181359046	13.19569003094165	4.52205814816016
H	9.26382802197536	11.79622826498519	3.67006753209609
H	8.98942552817985	11.72232134638220	5.44666389480021
C	10.91507256376897	8.15200948559793	4.71832030996733
H	10.96889926980003	8.55489232591055	5.74918825413083
H	11.06930495091022	8.99341535341163	4.01240271571256
H	11.76108370220171	7.44721765333709	4.57780162995336
C	5.39255761555579	4.08164032968351	7.22665264684323
H	5.34596420007016	3.96865977691788	6.12467710292265
H	4.35365885458614	4.07542686046777	7.61723838676614
H	5.91205258537224	3.18870213826549	7.63370937848499
C	6.13307455650239	5.59491094407889	9.14286553378690
H	6.65613617812236	4.77185242311114	9.67359707079255
H	5.09886318187555	5.64536435723799	9.54155463050324
H	6.64575263542429	6.54527429480802	9.39189520896422
H	7.51515148559992	8.37391467334724	8.55507239347770
H	9.06642442253613	9.32631508609341	6.90867376975912
C	9.12868526646527	12.16321354362083	10.36745928675657
C	9.04887063356782	13.47364249069021	10.89866573906106
C	10.15277447900044	9.86665269350235	10.28368519630773
C	11.05119950107247	9.53147272326117	9.23314481131815
C	9.96136488160772	13.88210684176024	11.88447156682003
H	9.89510807634197	14.90155483376992	12.29434630093244
C	10.11455572070804	11.25938572047264	10.81990907023331
C	10.94879808495418	12.98882248958969	12.33507484689105
H	11.66425785903630	13.30668505791724	13.10869597096516
C	11.02340485218243	11.68982373472254	11.80672149452662
H	11.78993882976704	10.98650620619603	12.16705144638352
C	8.32069009480675	9.24097207291748	11.97716427249995
H	8.47053723867559	10.31027311271705	12.23064836053770
C	9.29184912079777	8.89277368296327	10.85317825932105

C	6.67696886281153	14.33535612002205	10.93911973205111
C	8.26233503130977	15.21632252695812	9.25736933638243
C	7.97831970033873	14.37539971640046	10.36746203347595
C	9.62687744030243	15.19840511198815	8.57896940882396
H	10.23677886064930	14.42222978063228	9.08369624166514
C	11.94607074399412	10.59990727813810	8.60813611069946
H	12.15872508022403	11.34860602252859	9.39999658417306
C	9.34799248859709	7.57636036608046	10.35844771942627
H	8.68629190733636	6.80640882544118	10.78282963031574
C	11.06386646812421	8.20542244333440	8.76574979544410
H	11.73917275036807	7.91912626314253	7.94728036261386
C	6.34162023113068	13.39164340244133	12.08844448033623
H	7.20687187108549	12.71068999155098	12.22302510831911
C	7.24507116996805	16.05920717715230	8.77244864032146
H	7.45174809276270	16.72175056335013	7.91809026260592
C	5.68923834358757	15.19596115260621	10.42359013306027
H	4.67957755772103	15.18644642930731	10.86143272180154
C	10.22249091723558	7.23436141546938	9.32339131061305
H	10.23222850860993	6.21082129017172	8.92626162591306
C	13.29487520480307	10.05031735375256	8.11829345364142
H	13.16983988244217	9.37953935722314	7.24309307120283
H	13.95610008651889	10.88067407796956	7.79682633173273
H	13.81733009070030	9.47907276258783	8.91200966545506
C	5.97310958837808	16.06218793600154	9.35983604305708
H	5.19015841010989	16.73277276767732	8.97319289493246
C	11.21869095982988	11.34071444037988	7.46905182765819
H	10.26104518338111	11.78373382094163	7.80952931818431
H	11.84627130392319	12.16228615013384	7.06644955752128
H	10.98010341672598	10.64745888014007	6.63824929304856
C	6.16567769561783	14.16962915082650	13.40544844093487
H	5.31936828106153	14.88519734472224	13.33995152335023
H	5.96039290010063	13.47757188832473	14.24813873630019
H	7.07769360922476	14.75016111945773	13.65120943804209
C	8.61531729595582	8.42740499640828	13.24982246323607
H	9.66275581501543	8.57261833916521	13.58310788171814
H	7.94331611237079	8.73542989119145	14.07754427093555
H	8.46347602408948	7.34063793888355	13.08308186264215
C	6.86021666416979	9.08396701536067	11.52011541997502
H	6.63141840471587	8.03659754476122	11.23512351569524
H	6.15743407039138	9.37798611259530	12.32648905522364
H	6.65118943070572	9.72153524213170	10.63610574210123
C	5.11766756062958	12.51688692642606	11.76467236321941
H	5.27018207738000	11.96729345313617	10.81249947510325
H	4.94361167984895	11.77290250701130	12.56833604740139
H	4.19239830436579	13.12224919914331	11.66761861714171
C	10.35853832320205	16.54117059915620	8.75142262764023
H	10.48248215010737	16.79481349915537	9.82364537891649
H	11.36591682870224	16.50230388435451	8.28808559644678
H	9.79904312474872	17.37138394587442	8.27177389739539
C	9.50479170165292	14.78882818414164	7.10002065966145
H	8.94835526669297	15.54680988828771	6.50934029561693
H	10.50699240418462	14.67536366600398	6.63929719782705
H	8.97033819131121	13.82151942279275	7.00633437556185

Complex 4a

Fe	0.42964771212811	10.49671913870026	11.68092839669237
Ge	0.96286860477251	9.32225352035556	13.50856490707446
Ge	2.37095172401234	9.65459056413571	11.11205983597555
C	0.29548220004466	8.47232796394689	15.20765364480965
C	-1.78403629806836	12.75009145708284	15.36490264658641
H	-1.43970910319086	13.77700980941141	15.49989621408761
C	3.00594619595970	8.89788406408138	13.69259894965151
H	3.06605283179170	8.00821744608745	14.31975567078962

C	3.74231302189799	10.36659036417723	7.09308753223610
H	4.53415958106279	10.07226588026758	6.40213235303814
C	3.60925874178089	10.05204487213180	14.42142953555034
C	-1.53594741962801	10.60886989457129	10.89853932759067
H	-2.36928207116631	9.91326415074486	10.96854785106815
C	6.19388023038778	10.83072865992548	9.62222661293377
H	5.22251328129658	11.35114192065319	9.59643533904875
C	-1.39139016773054	10.36829477071843	15.52523758639516
C	4.72807645894552	8.87028221075869	8.91543503262412
C	0.45464473302503	11.97234483819337	16.24659336505180
H	0.98194303879709	11.01092381581127	16.33602549381596
C	-3.50858255319427	11.20381418405111	14.69403266464228
H	-4.50849056636145	11.02293422959695	14.29521791860431
C	-3.07137732004076	12.51407829969276	14.88731615295728
H	-3.72955600537557	13.35129714017336	14.64936780037556
C	3.57244750416436	8.62347940258748	12.30500357400824
H	3.46016378935232	7.56294352818758	12.04200420513263
H	4.62990525882916	8.89369773218550	12.17604564074525
C	-2.67979960534416	10.11648239617712	14.99836266198614
C	0.76486075071811	7.21152398818814	15.66586724481818
C	0.53582543676052	11.35604781717710	9.80218447972345
C	2.73923715468346	11.24770451378551	6.67499442563971
H	2.76505893042263	11.65123259461791	5.66166388958132
C	-0.60673566809211	9.19312556862341	16.01953990806515
C	2.67949010295680	10.21179954924739	9.25454232599762
C	-1.36711848398848	11.58866521132703	11.90150174199142
H	-2.05290083655592	11.63113397906483	12.74328615848520
C	4.42357166722242	7.49235981205630	8.88547277461850
C	-0.53497829015537	5.99359273900399	13.30960434369410
H	-0.71951785633958	7.07205849289546	13.42851524710828
C	-0.92961951910461	11.68837112243424	15.69381484401813
C	2.86387148493742	5.89047579204292	15.14479896505594
C	-0.90879664492905	8.74684113242599	17.31527547562580
H	-1.59114834512208	9.33638464918183	17.93014880793632
C	-0.77706579222713	9.40849219219660	8.77275920900717
H	0.22225923940592	9.17631252364938	8.37336703269634
C	-0.59120131533356	10.46339365474747	9.84675389932534
C	3.72304391300793	9.84291339522846	8.39230863281939
C	5.89673577925989	9.33999806485845	9.55799722599652
C	3.53130489426089	10.11646190173433	15.82725770640362
H	3.00121690805134	9.32621033103759	16.36185484777460
C	2.41667379921823	13.82052965751355	12.03643320178282
H	1.69479613857149	14.42774755490567	12.60125446119888
H	3.30942247240367	14.44231329577672	11.87672362002915
H	2.69991146293122	12.96320793727511	12.65852432409391
C	1.85273457453725	13.36286890607843	10.69239013455506
H	2.66096315319510	12.85464780883029	10.14338840672190
C	-0.25532780173289	12.46671417066691	11.84918337539632
H	-0.09775936223883	13.16712555348601	12.66697561837402
C	1.54314756395831	6.27206095365270	14.79735436642876
C	3.57077146468071	5.02849822917488	14.29569319401348
H	4.59534030490265	4.75231678379909	14.54804678378634
C	0.93417076857438	5.73459124616368	13.63108346078488
C	1.68340449971804	4.88093378147790	12.81179435737868
H	1.23263543641928	4.47098249529289	11.90962554917348
C	1.69401055589789	11.60585355310270	7.53488064323317
H	0.90926930339819	12.28261130203425	7.19284110054197
C	2.99576472392453	4.53769668240431	13.12963884278871
H	3.56486129420118	3.88040208193619	12.47016399800760
C	6.99313416566524	11.27901145253020	8.38575945590515
H	7.96246922056960	10.75930783069722	8.34982022655055
H	7.18474444796524	12.36199285747134	8.41698585747286
H	6.45310027177287	11.05347996689594	7.45705309110033
C	6.91335002064123	11.25466474780140	10.90891379427813

H	6.40481740085649	10.87409631770690	11.80501717787453
H	6.94933095128389	12.35146397033789	10.97585309767695
H	7.95239956722038	10.89417687031168	10.93306114252145
C	-3.17266922874469	8.69979944857854	14.74351394258242
H	-2.41801953741761	8.00547627048392	15.13755437858101
C	4.81047658470063	12.17317359205949	15.86613208236129
H	5.28187862906439	12.98410880251029	16.42253771350970
C	-3.28827759121290	8.43140735361885	13.23539693718756
H	-3.63232455866878	7.40299261594417	13.05006323432184
H	-2.31499534405501	8.56480010494847	12.74144562124887
H	-4.00863149836606	9.11743934283877	12.76406326030433
C	0.70293248374182	12.37971480049645	10.80267322103526
C	4.27780309029619	11.09781641383502	13.76144606180427
H	4.34163934196340	11.09109609719201	12.67224569323331
C	6.47467700944902	7.04375042219145	10.10897246750210
H	7.15447221602108	6.33215488449108	10.58009564305289
C	1.39733561361944	14.56164932423066	9.84171171084848
H	0.57021515638852	15.09274528249677	10.33677111283423
H	1.04704545854593	14.23896361063075	8.85143456411266
H	2.22433253954290	15.27245213266497	9.69840507833215
C	-0.38166437693809	7.54937609636907	17.79149201423931
H	-0.62483669124893	7.19929440001312	18.79572738222221
C	4.96603722976320	6.95050735025349	16.12816818220688
H	5.36733056051693	7.42581571400226	17.03453727103845
H	4.95788397769899	7.70250007391819	15.32962117314473
H	5.66455828306198	6.15237527412768	15.83685786888758
C	-1.37637368879396	8.11033636605903	9.31146472972946
H	-0.83009603932687	7.78947723544312	10.20542654896264
H	-1.31626787937802	7.31476397099174	8.55539711954840
H	-2.43698179038816	8.22401611460719	9.58117959831965
C	0.41356324490977	6.77216336464598	16.95210152588339
H	0.75517139609203	5.79155460624389	17.28509189389510
C	4.87280557451639	12.14211204595509	14.47140863522057
H	5.39078508759628	12.93431446557271	13.92850944882316
C	3.55979484712937	6.39273539262845	16.40406987144778
H	2.94809675345931	7.20716493305593	16.81844553465090
C	5.31206685469845	6.59309987564523	9.49046386843056
H	5.08376058881314	5.52583690089566	9.48465199598381
C	4.12689963142986	11.15446426930645	16.53967378209727
H	4.05940787585012	11.16863773125513	17.62890004092106
C	3.14505922598967	6.97352595755355	8.24256968438113
H	2.65597581449062	7.82245336814646	7.74269950851107
C	1.64858991595290	11.07876140013686	8.82846317332296
C	-1.62075615033016	9.98416157069424	7.62379880266088
H	-2.62299808475848	10.26208827324357	7.98387758956224
H	-1.73978235429092	9.24298883628166	6.81976860409997
H	-1.15524521684871	10.88222821152329	7.19489675048109
C	6.76355746075764	8.40799285927373	10.13972277826466
H	7.66578737506616	8.75294330151797	10.64525556929386
C	-4.49002167410089	8.40259984584162	15.47301080045410
H	-5.30916916150690	9.03931634379533	15.10714679866454
H	-4.38843148933958	8.57355586655269	16.55394354418129
H	-4.78690200013343	7.35496058191669	15.31709275536106
C	1.26726386691188	12.84530475312296	15.28584637420876
H	1.35891962504757	12.34634489992713	14.31224488051722
H	2.27879832026992	13.01574649336910	15.67526193598949
H	0.79210510659861	13.82565422889420	15.12922315224118
C	-0.92175434397841	5.62476391474894	11.87734881136669
H	-0.23544790599769	6.07236375138540	11.14750583639631
H	-1.93443382198241	5.99018169149169	11.65993419593430
H	-0.92316256956369	4.53482672615672	11.72378384303358
C	3.63925408972382	5.27862330014841	17.46312306225716
H	4.24058712772026	4.43637479574261	17.08951994371969
H	2.64618961645341	4.88692551239576	17.72143190940054

H	4.11194788899098	5.65265928731890	18.38333608473111
C	3.43845517221678	5.91533074074091	7.16771729383589
H	4.12717077044509	6.30687100118372	6.40598785467879
H	2.50848189957824	5.60803443467491	6.66717110252765
H	3.89591159248963	5.01454296771309	7.60292372602135
C	0.38287609793568	12.58585269471416	17.65259151797322
H	-0.12285222754557	13.56317763541218	17.63273688907145
H	1.39572418820790	12.73601628349885	18.05460985247083
H	-0.17069909387352	11.93403179317496	18.34353023614334
C	2.16726692545336	6.43132349230714	9.29737476138715
H	2.61922856419662	5.61330227536999	9.87694695073759
H	1.25174672521819	6.05137430984069	8.82096243772875
H	1.86840616637906	7.21335275337549	10.01272285650482
C	-1.44936083580659	5.25556081456119	14.30402583248827
H	-1.27987997933321	4.16964466336858	14.24479998891911
H	-2.50566693744672	5.44988978274001	14.06679338132762
H	-1.26885220011079	5.57435261868532	15.33771533911623

Complex 5

Fe	6.82148419665576	3.20674388048992	8.18098780089882
Ge	5.99627309073037	4.03914082465541	10.07115755887804
Ge	7.95460075177524	5.11284472605825	7.92069245516962
C	6.00485300822991	1.44944717689548	8.90268589269928
C	9.67249555220288	2.59299646970155	3.86642003295460
H	9.22604843804497	2.07908832884547	3.01423554163248
C	8.79091977890349	8.68897236472920	5.63648398079505
H	8.44453636297781	9.72280379758862	5.66174326442064
C	4.70328559793998	0.12016837356539	10.61006338189172
H	4.88225634832502	-0.78573690319499	10.02853223259685
C	11.15172648572158	2.56022591375040	5.77118395189068
H	11.86811917730311	2.02230671201144	6.39141161552286
C	4.20705621211289	2.47850205903454	12.08168154903875
C	3.62405802055642	1.25680472442574	12.45336004960015
H	2.94149924521249	1.22877751795815	13.30284560821518
C	2.99429287041029	5.98804641768786	12.82121593636554
H	2.45264857532128	6.78908644506830	12.31643906990459
C	11.57248033471621	4.63247426810229	7.18028890898370
H	10.84797351891079	5.30525208272632	7.66877060686490
C	5.25956362518067	1.33383512007441	10.19734922821117
C	5.97123369663283	2.15447203554827	6.54634355100611
H	5.43363470740237	2.50378242543517	5.66628960233509
C	9.74722468089296	8.29444352238665	4.70102757252367
H	10.17928722616709	9.02269821478273	4.01337109577204
C	5.05010595468528	2.51339809118896	10.94896597940382
C	9.31642639802808	3.92085884422449	4.13918008487392
C	10.11560019850915	6.95553721851649	4.63024518329989
H	10.83022334101718	6.62038152054481	3.87563459110471
C	3.19067092968007	4.76896623341929	12.16207271987679
C	8.81418924409058	9.60634546151627	8.84192260242007
H	9.52416942864796	8.98774257241865	8.27197893734122
C	3.88619088618742	0.08465173148502	11.74230730995128
H	3.42894495020109	-0.85456960684100	12.05683769945476
C	3.48052576164893	6.19470560620581	14.10947438793501
H	3.33699925379942	7.15757286271289	14.60230130407285
C	10.57503742931897	1.91278201141034	4.67956327384599
H	10.83306175973058	0.87520183234329	4.46200525897378
C	8.85211599250134	5.53048386015305	10.81374829101081
C	10.83954923590563	3.89312710592924	6.06879827353956
C	7.43850772536898	1.34801031406708	8.88128255413130
C	8.24227018506500	7.76849660168910	6.53741579899478
C	8.63904281233462	6.40344988973403	6.49423083696987
C	7.79934275979884	5.25419444817836	11.63126980317110
C	5.25912625381414	1.83484411389442	7.73220644240206

C	8.19381586763487	0.91021110671687	10.12034258021298
H	7.55764617341066	1.16080847138180	10.98326761284104
C	9.55394169149669	6.00245582598155	5.49963483085098
C	5.07026699347026	8.93713446522354	9.19048612764328
H	4.26585993169615	9.19459835117618	9.88131082012907
C	3.90047962122092	3.73605545810120	12.82442162559068
C	4.96329641078999	2.78517690509338	14.96673454552440
H	5.31267474101945	2.02034902849087	14.25711821082017
C	2.54928097437444	4.55964859584893	10.79603018050796
H	2.97291835096385	3.64646342930238	10.35677345626873
C	7.41866744321874	9.06573425825839	8.56268661252596
C	5.48986105240839	7.06853384358568	5.89255523409792
H	6.41100465899846	6.60567038491870	5.51559612383318
C	6.38742121109211	5.60416105274218	11.23960554765377
H	6.30223463968770	6.52182358990204	10.64337391453621
H	5.71330144787550	5.66140990619581	12.09978624604815
C	7.15232223673238	8.22420280258334	7.45491320459132
C	8.26799764735214	4.61563140276504	3.28040071582148
H	8.38192540849329	5.69791748979651	3.42812405794059
C	3.88949912931254	2.14290039520457	15.86755352659672
H	3.54316325958000	2.86610285988436	16.62140413459185
H	4.29654862300120	1.26741676691686	16.39506503302222
H	3.01072362356696	1.82206723452965	15.29340383133576
C	10.27904788627910	5.18878922304096	11.13573556831296
H	10.88906862395211	6.10604116943305	11.19938974422633
H	10.71540998445903	4.58451077703778	10.32581307117657
H	10.40377528368894	4.63574234412583	12.07265143764908
C	8.10284217106122	1.63734535852157	7.66071136164578
H	9.19038788917413	1.64749081479112	7.62011913965671
C	9.21247071059804	9.53837377329743	10.32482439048510
H	8.61457470392327	10.23010895775804	10.93611847087172
H	10.26627554548730	9.83064513282998	10.44192590399234
H	9.08767192739067	8.52787632676653	10.73451886551126
C	1.03893757511107	4.31743739831704	10.96572061263174
H	0.54505435603262	5.20048817948508	11.39855605792936
H	0.85158304550739	3.46508142979110	11.63375421554919
H	0.56994916179108	4.10452448848632	9.99352130260111
C	7.38059284335633	2.05010694869744	6.50980671176767
H	7.93132951532130	2.35934843973229	5.62631988177418
C	9.90039412139971	4.56882445596511	5.25463405508839
C	3.74023797513645	1.80614939307786	7.76183840027154
H	3.43178699267035	1.88751213536227	8.81436931753183
C	8.66408782058898	6.20984863853149	9.50378522255789
H	9.58848065187307	6.69328120660755	9.15878224716512
H	7.89740723400663	6.99355235769417	9.57440788926085
C	12.68955423965925	5.51192284931019	6.58842210452101
H	13.42868354747685	4.88527457441720	6.06675578887603
H	13.20799226617549	6.06229715318070	7.38753731563423
H	12.29222771098354	6.24291392301768	5.87378552497047
C	4.80610734897495	8.15191219654890	8.07207153851755
H	3.78745806848519	7.81683219635034	7.87778088126197
C	4.13338223583698	5.15964086245363	14.77433338389426
H	4.48921994200922	5.31872088025876	15.79297944781763
C	5.83040333230843	7.79347856188656	7.18586604514432
C	8.93855351583220	11.05606633062271	8.33763627547453
H	8.71254757618249	11.13676025624589	7.26635123889274
H	9.95717924567568	11.43737813931962	8.50318310629247
H	8.23705034385358	11.71066926656242	8.87666573659052
C	6.36303344722161	9.40527047452263	9.41863294849431
H	6.55786658286518	10.03169069352154	10.28994815554918
C	12.15213029194470	3.71181585474122	8.25635446459439
H	11.38755071163847	3.04714837965184	8.67914398304703
H	12.57349410696276	4.31128505179621	9.07501770955729
H	12.96874501055964	3.08882965345568	7.86176608189633

C	9.52585214516811	1.63692612107639	10.29772541475136
H	10.25463750970080	1.33764749398177	9.52988514638431
H	9.96626723900271	1.39695939922974	11.27584753790655
H	9.37531665317299	2.72250748562942	10.23555874131658
C	4.47349933733962	5.94633436888055	6.08333111689761
H	4.84591394630060	5.23342730495105	6.83232454765464
H	4.32577941586174	5.40160878185109	5.14008624987421
H	3.48988147923660	6.32037547012707	6.40404526824284
C	7.89523389201455	4.53466128297482	12.94783159527455
H	8.88656846497795	4.11727315579958	13.15495887891968
H	7.16732244643798	3.70822118875503	12.98322226287341
H	7.62793810574185	5.21491028819483	13.77387357429672
C	4.34092345665467	3.91794177564616	14.15985970169989
C	8.43416254951167	4.34270532987674	1.78054274320948
H	9.45038232265291	4.58654003769379	1.43971445224509
H	7.72364947281666	4.95433644876752	1.20579125294801
H	8.23648453952444	3.29065684815737	1.52680612135921
C	8.37837846476063	-0.61614826895878	10.09938831690970
H	7.40973663075983	-1.13025658182112	10.02971468826011
H	8.88481003376643	-0.96074979368805	11.01294528206856
H	8.98649419974103	-0.92086822615251	9.23395606404124
C	5.02944376681656	8.09013292118715	4.83845428597333
H	4.11294742431803	8.60324170823390	5.16700935236987
H	4.81813708918853	7.58911337283588	3.88175249950869
H	5.80157636362693	8.85296852784968	4.66427731406148
C	6.16041079723720	3.20608593976269	15.83128174152019
H	5.86175738095511	3.90694431029367	16.62448623228961
H	6.94779063209000	3.68301691127726	15.23709204287840
H	6.59168185668628	2.32310507306847	16.32486541020367
C	2.81796759863521	5.70621480835876	9.81378943771840
H	2.36860249222609	5.47928322979427	8.83662451695025
H	3.89503690293268	5.86528964514037	9.65470232982214
H	2.39009837429621	6.65732301520045	10.16341507310157
C	6.85626275151027	4.24293007717895	3.76137341470987
H	6.66610671075290	3.16890225627704	3.61081855267951
H	6.09397169792217	4.80467268067362	3.20133192315790
H	6.73351320338663	4.46387473680196	4.83059507482450
C	3.27184030167033	0.43237093257666	7.24860130725980
H	3.55812147295376	0.29556959403060	6.19477253304926
H	2.17804717150353	0.34386893594947	7.32211190333425
H	3.72217870384677	-0.38497852064802	7.82836456477255
C	3.06211329306012	2.94117598515288	6.99845950453880
H	3.37559369779077	3.91330883013732	7.39316152972504
H	1.97008223839930	2.86008416717840	7.09900669683696
H	3.29463008953578	2.91999086778289	5.92402120896291

Complex 6

Fe	0.78547624616341	8.79381827163411	6.80469960296017
Ge	0.14286280032169	7.43649897197076	4.91297410882873
Ge	0.51951498512969	10.54026860861234	5.66662446719581
C	1.15228091686448	4.55264792543850	3.17860009650534
C	1.20024893302069	6.91342518616541	7.59810311229047
C	2.39007290027933	7.71980162751953	7.62283205223943
C	-1.30593913042940	12.48963951642869	7.92717617562588
C	-0.08062242529334	8.68297790439194	8.71652938089700
H	-1.02267064358662	9.09821838151636	9.06907847818801
C	-0.04101607723676	7.37643839341492	8.16724663225950
C	1.22051721390218	4.53536998571284	4.67179826433078
C	0.94798993843035	5.67504703469619	5.45373853777802
C	2.30205178398664	9.00360285133429	8.22329384686182
H	3.16082520845308	9.67037808665205	8.19001441270171
C	2.30360926283608	4.95791844672928	2.46711941559728
C	0.18389551531081	7.75214546877338	2.94001466779300

H	0.45616161680747	8.79940673055433	2.73192052959478
H	0.91935410456875	7.13529962003418	2.40846621397267
C	-0.01151725319831	4.13778585428875	2.49406592157854
C	0.28780048737228	12.36956970316834	4.97340217298212
C	2.26852119925994	4.95441995304971	1.06629341715965
H	3.14574707466176	5.27985261236693	0.50469022456772
C	-1.38020520059228	12.24434334381599	9.30439368466585
H	-2.30704459208393	11.85849984615659	9.73192122580532
C	-1.24316596685699	6.45499494960417	8.23164806344056
H	-1.22216631801266	5.82082815609634	7.33198064204622
C	1.08644205726676	9.48205010618260	8.76830879509576
H	1.02025805936046	10.50884414498639	9.12013847678573
C	-1.22288054649078	7.46807897050820	2.46644382280328
C	-0.01294308004216	13.35474741428763	5.94448854655753
C	-2.20713621467337	7.27961624042704	3.37713003987151
C	1.91467155929835	3.31649614480690	6.66820273143513
H	2.28490448578160	2.40090068951198	7.13147979623800
C	-0.19479134770349	14.68555221268756	5.55398892532721
H	-0.43326677914227	15.43123719646582	6.31452145013739
C	0.90028904503872	12.98192745647310	9.59835467102417
H	1.75338169159072	13.15830694110327	10.25532929670197
C	-1.85388736992746	7.23239333079730	4.83943275821248
H	-2.21784871906736	6.30159934641893	5.30590696975053
H	-2.32137581424848	8.06011794785112	5.39644162894556
C	1.02074779932119	13.23080220709944	8.22533814304531
C	1.69668954803370	4.45747928361437	7.44098536109672
H	1.91295324303104	4.44823959111484	8.51036832513731
C	-0.10152675881395	13.00200135501808	7.39453619995066
C	1.70739207614650	3.37064435491376	5.29131640841432
H	1.95184461893630	2.50851734794091	4.66821602672824
C	1.24851570524053	5.63367960586103	6.83117833873138
C	0.43381063938717	12.74389960056146	3.62276770748204
C	0.00164620913363	4.13141369826358	1.09397702661819
H	-0.88772379083055	3.81632080873561	0.54875415474986
C	-0.05785272411567	15.05436574761570	4.21481501616505
H	-0.19651404486947	16.09391459196920	3.91514049609212
C	-2.58715930370332	7.18433838090352	8.27496932568249
H	-2.71527104473866	7.73904401243463	9.21659794155908
H	-3.40952606349629	6.45808192820006	8.21036060209038
H	-2.69040463452601	7.89023955968932	7.44320624238009
C	3.70192101276929	7.22535184751666	7.04583481841601
H	3.45138575992727	6.51723170029265	6.24175225726596
C	-1.10121749823761	5.52966402788871	9.45479660813339
H	-0.17692399106830	4.94039586463071	9.40639246175044
H	-1.94924384698580	4.83126983663737	9.50774337892399
H	-1.08277034974188	6.11981145411390	10.38353449115721
C	-0.29338241912807	12.50242083875813	10.13742592980129
H	-0.37038272894945	12.31476402363551	11.20956410278305
C	-0.26709315891704	11.40933374196488	1.63203669887609
C	1.12939593407976	4.53978213738129	0.38261337149152
H	1.11594848220811	4.53750172113526	-0.70868959037318
C	2.34956944826301	13.68675728986943	7.64099331261778
H	2.15969688683612	14.02752610791536	6.61330610676372
C	-1.40774799202372	7.42124873446005	0.97816552030704
H	-1.09007565758746	8.36645961408007	0.51646983500311
H	-2.44014096411857	7.21411574379964	0.66947990165298
H	-0.76012449414211	6.63609287615279	0.55698574284148
C	0.26399236366480	14.08891832311138	3.26208492463839
H	0.38141473033821	14.36821384104689	2.21360687084406
C	-2.51857331219239	12.24312704356162	7.04407574611815
H	-2.19620532359274	12.36584618053918	5.99962810199876
C	0.74679027875384	11.75551867619748	2.55242359534321
C	2.06517597274229	11.25867949863428	2.40527720284492
C	-1.24221531020258	3.67949474304708	3.26257034086881

H	-1.28666395615618	4.28686414542088	4.18170246860409
C	-3.67305471373375	7.15400289794204	3.06598840896873
H	-4.22013022462189	7.99895165550840	3.51854725916483
H	-4.09831263006870	6.23952768288890	3.50952138816901
H	-3.90018896126651	7.15003243865446	1.99243630891325
C	3.56213547156640	5.42422593041049	3.18610048651107
H	3.46010640227278	5.15016835295044	4.24591390010812
C	0.06847522079397	10.60189207264277	0.53870877643478
H	-0.69747008056408	10.35299215882071	-0.19717450735194
C	2.34358983419545	10.41898743270915	1.32054289196971
H	3.34757594479504	10.01436758925729	1.19706403269701
C	4.50044517024363	6.46489727630641	8.11887864740073
H	4.74143813057724	7.12920130724363	8.96283926291764
H	5.44480143161614	6.08487499622210	7.70206061390384
H	3.93278267507479	5.61080149886337	8.50925528371543
C	1.35929741578462	10.10500763500487	0.38447576310797
H	1.59791907087818	9.45760695334332	-0.46041353235489
C	-1.70232060505040	11.87882647704513	1.81558884148228
H	-1.73374535339578	12.50543597934819	2.71804481489637
C	4.54408901682741	8.35111174437298	6.44142344359065
H	3.93653257044179	8.97358354544942	5.77303926666457
H	5.38285469981589	7.93343098166284	5.86737087274318
H	4.97545848431063	9.00080595143579	7.21798772823491
C	3.17376747390826	11.70093190138449	3.35050498338933
H	2.72596726775662	11.83269697764371	4.34862210708246
C	-2.54869889206546	3.88879443692626	2.49070159024162
H	-2.61583073327439	4.90701850668420	2.09118487852473
H	-3.40948015926419	3.71785978890801	3.15272992241941
H	-2.64033402621313	3.18037709582055	1.65326195007507
C	3.70353228524030	6.95300625268046	3.11982345153059
H	3.72768875933826	7.30207541229721	2.07657100186576
H	4.63390173673896	7.27573957310247	3.60887751333922
H	2.86297385085484	7.45398721717983	3.61893031242918
C	-1.11789588002081	2.20188608080603	3.67830651076806
H	-1.00546879861526	1.56489884565003	2.78776868602094
H	-2.01955938346249	1.87750913923682	4.21942299907254
H	-0.25199887117335	2.03448497613237	4.32977936933139
C	4.82520747754206	4.73015702679813	2.65603877775679
H	4.72236626382002	3.63653865512621	2.69345971903057
H	5.69853630377550	5.01586756899394	3.26064237328985
H	5.03828914938389	5.01314907252495	1.61464899652852
C	2.96489547287737	14.86395857364210	8.40928353329659
H	2.26666205357822	15.71080544499223	8.46536867889166
H	3.88194862221642	15.20629207562222	7.90861381422005
H	3.23611199006467	14.58189816148113	9.43727683561173
C	-3.05006131579313	10.81117079783506	7.19046256960256
H	-2.24558515075617	10.08804833038129	6.99224731151922
H	-3.86579706390331	10.62877317107468	6.47595122207432
H	-3.44371987537939	10.62170758895321	8.20030101002681
C	-3.61153489361230	13.28925349122269	7.31588296400219
H	-3.97071997983004	13.22222713663596	8.35381487305651
H	-4.47123778242221	13.13234290192314	6.64789559221679
H	-3.23263765733389	14.30866001987415	7.15582872597882
C	4.29683369704557	10.67231034237145	3.49077727635568
H	4.90023751521680	10.59513323991472	2.57394073009890
H	3.89575425613973	9.67703567117521	3.71860659161347
H	4.97572128323722	10.96332782715829	4.30448577806689
C	3.33013610357492	12.50457497107650	7.55466927733481
H	3.56965561053412	12.12286088812871	8.55901562743286
H	4.27050297671494	12.81232071340117	7.07410897874432
H	2.90256960891908	11.67474933993468	6.97092424785662
C	3.73468517646808	13.06755070084767	2.91806199382493
H	4.52145962743449	13.39830587822058	3.61254955919424
H	2.95022575030473	13.83547265250841	2.90119896590232

H	4.17120842210912	13.00165620657115	1.91002272265971
C	-2.17089367795586	12.74517188238316	0.63686500798623
H	-2.17238703252003	12.17305165247164	-0.30282807882885
H	-1.51448404199363	13.61594778035763	0.49839221564554
H	-3.19438696411940	13.10961485379624	0.80838936546071
C	-2.65216690814330	10.69490143990797	2.05277046050571
H	-3.66911742130013	11.05769973239319	2.26339894319413
H	-2.32035151000803	10.07502855606386	2.89676002963502
H	-2.70555063392180	10.03807223333827	1.17279431088648

Complex 7

Fe	12.53193408771519	10.38956931731244	6.05831157282100
Sn	10.15505999956311	11.25036059323907	5.57006111677620
Sn	12.03534121353855	8.66162810969400	4.65682411107896
C	10.58118148122614	13.28821804816741	6.27888364606201
C	8.54517191221236	10.45060956832334	6.83572442223643
H	8.80787433823969	9.44842676506901	7.21043962539789
H	8.35942623769170	11.07449272857094	7.72613564941803
C	7.32138369110084	10.40732798652049	5.95719998308696
C	7.36617257005170	10.80459449363570	4.66016578098151
C	8.66403767795011	11.17806401970231	3.98528124689505
H	8.57712581782809	12.12389474640269	3.43749504786373
H	8.94571921218215	10.41072757960984	3.24953924196185
C	6.09483448207393	9.85162650543037	6.63611682999927
H	6.25819111286031	8.78618609681049	6.87376562579668
H	5.17974163574736	9.92066277901186	6.03516745609052
H	5.91220209970322	10.35585803228495	7.59850078607091
C	6.16606344960065	10.87638132761650	3.75370491745320
H	6.26365689568370	10.16221105143462	2.92043239289494
H	6.11055820205477	11.87802300148942	3.29958178920964
H	5.21442135586761	10.67958547454551	4.26140063700181
C	11.69271443183588	6.82212843333565	3.57059643247883
C	12.34110259590052	5.69588561189765	4.11586310061445
C	12.25875844914520	4.46179234062887	3.46174486258241
H	12.75910583400456	3.59214150878112	3.89156260484711
C	11.54582674414627	4.35264427689540	2.26491227477197
H	11.48764092727336	3.39224256142875	1.75093676935241
C	10.91395778374280	5.47268330120461	1.72027674288559
H	10.36422442560916	5.39218023167970	0.78057426370748
C	10.97767865765753	6.71692451956368	2.36555739386230
C	11.73807965067627	13.35023771926780	7.07729377184528
C	12.09676336264066	14.54139999598672	7.72266838354311
H	12.99633399913509	14.57452400354774	8.34043393198499
C	11.33402201503270	15.69352423477063	7.52665355831192
H	11.61843047917072	16.62753224586726	8.01342730374415
C	10.23734203231152	15.66082299618430	6.66374449183937
H	9.68237439032607	16.57612106149617	6.45026642536708
C	9.84826053649681	14.46449640127270	6.03884935855736
C	8.74003023014092	14.49505312383876	5.03691534763562
C	9.06840571041755	14.55275673271403	3.66064155206374
C	8.03165640388704	14.66279429946838	2.72644382203583
H	8.26815196971622	14.71495023470409	1.66322991382993
C	6.69867427513842	14.68634730134361	3.13388485374290
H	5.90208529874378	14.76818337541761	2.39228484506559
C	6.38461027823674	14.58306220485406	4.48665578580421
H	5.33893936436753	14.56971288946037	4.79636435941612
C	7.39323001064242	14.48569183332838	5.45437716139608
C	7.03488978115658	14.38077305692955	6.92791755482158
H	7.91411643474051	13.96372837393847	7.44409992779212
C	6.75143011350263	15.76702279352693	7.53096632551836
H	7.61732270170197	16.43504600330084	7.43508246180237
H	6.50345903889193	15.68093617066146	8.59969539607800
H	5.89968944169227	16.24193861217519	7.02036276705580

C	5.85360271186368	13.43436138800099	7.17564635711369
H	4.90519272002320	13.86820635282899	6.82446122433320
H	5.74202263053019	13.23660335733161	8.25165513113073
H	6.00837949152819	12.48165861218528	6.65523160765413
C	10.51813626915953	14.51282966894833	3.19778072359755
H	11.09489272159056	14.01067036395156	3.98793535802927
C	10.72632982322124	13.70444251101601	1.91151629566102
H	10.24004935147460	14.17331596862587	1.04322268846026
H	10.33492084628885	12.68234290650132	2.01224050827296
H	11.79969070737193	13.63467083864635	1.68635776577511
C	11.08252612402112	15.93700262239497	3.06115162215290
H	12.14287260958279	15.90821388259061	2.76781376261976
H	11.00436193401870	16.48083208067348	4.01238985843748
H	10.52966498512026	16.50398866925390	2.29670195907058
C	12.66829884412184	12.18061136243188	7.10864550307041
C	12.46447898867475	11.09252004700306	8.03376860047117
C	13.31014164842054	9.95517488048084	7.93933247699694
H	13.13209921663768	9.08629135441040	8.56946827835116
C	14.34633913512452	9.90736235212950	6.97347437868910
H	14.92411451193463	8.99298663000933	6.85501182105547
C	14.52434148318103	10.97724656753784	6.05946876454330
H	15.26334353083802	10.87679883459380	5.26515075286603
C	13.69440559095190	12.12975158620453	6.10249884855263
C	13.90885518695882	13.26260445705679	5.11627044368662
H	13.05573976133009	13.94873271995374	5.21293962822298
C	13.94017461888816	12.77876926500020	3.66414338565681
H	14.77162804021482	12.08334400807596	3.47760442334292
H	14.05987444618836	13.63114877487854	2.97923956194440
H	13.00613385534462	12.25619709858946	3.41987961561552
C	15.17890785564569	14.04691758854488	5.48247504220819
H	15.12616401176772	14.42615284950705	6.51266163608716
H	15.31301359717949	14.90480553589562	4.80758936210605
H	16.07135997022944	13.40740540887177	5.40344770089859
C	11.37932039420227	11.18080080346821	9.08867904540505
H	10.56082678021285	11.78006419411622	8.66017520974815
C	11.91327741485389	11.93495263586842	10.31887415848195
H	11.12991536303707	12.02811066396035	11.08553128952050
H	12.25020441805545	12.94554309628293	10.05274528024845
H	12.76439205896470	11.39580469091402	10.76224121116437
C	10.80967405603684	9.81757568303090	9.48692549758553
H	10.53594761377420	9.23336867234608	8.59994147662084
H	9.91134510336471	9.94902116319020	10.10577596635282
H	11.53194517895017	9.23334476129720	10.07706508614437
C	13.11803440151982	5.87434305489973	5.38105840789205
C	14.48906225512253	6.21711742774507	5.31228278420696
C	15.19026134817363	6.42213831998379	6.50804185437909
H	16.25141439837477	6.67601127959141	6.47176832409987
C	14.55020340448242	6.31448461101179	7.74117475656722
H	15.11045239741060	6.48219574340897	8.66246626436775
C	13.19215847592397	6.00702802881612	7.79780235040832
H	12.69544990323288	5.93991136397959	8.76708900145136
C	12.45495811306736	5.78445665933484	6.62818675087092
C	10.97525841379420	5.44489682720548	6.71316696389478
H	10.58488836232274	5.40735850509229	5.68558700461047
C	10.18250777911332	6.52232482960004	7.46727337355417
H	10.48960236892317	6.58781112158625	8.52136267022570
H	9.10695446210159	6.29632530868296	7.44377577797722
H	10.33620039234764	7.51319643031791	7.01374546832997
C	10.76974639690382	4.05433983237172	7.33531936070336
H	11.30604601456872	3.28298098418385	6.76461507846380
H	9.70177847843801	3.79195168809247	7.35080368434171
H	11.14082269150834	4.02639369427216	8.37075267370172
C	15.20248603524782	6.38061041266741	3.97863520375784
H	14.46692438994904	6.18837524589368	3.18373837009228

C	15.72093473178101	7.81541529187988	3.78914630260551
H	14.89939573553413	8.54731826606143	3.84439815602530
H	16.20703301921667	7.92302311522962	2.80861791815031
H	16.45737802849325	8.08277137366008	4.56157571756261
C	16.33129938046807	5.35069874982190	3.81840250474456
H	17.11062776793718	5.48861956537003	4.58265075533905
H	16.80686322963651	5.44907156126453	2.83161139256476
H	15.94547661336222	4.32612930315758	3.91423378389580
C	10.30593667875282	7.90681886078933	1.77054581016034
C	8.89563360309299	8.02130731196782	1.84568773483430
C	8.28290620908240	9.10102344521031	1.20219059847769
H	7.19876475309461	9.19863371138855	1.22602517441910
C	9.03894549136199	10.07376537329596	0.54677641817593
H	8.53998375160203	10.92084151712269	0.07431734230491
C	10.42542117571088	9.97231407073724	0.51194857308761
H	11.00977771805115	10.73659530983744	-0.00140888035521
C	11.08091960822603	8.88208715733357	1.10293027051989
C	12.58157408133299	8.70811189509160	0.91630100343955
H	12.92295014043082	7.95382812874936	1.64070771913762
C	13.37112729364382	9.99284561499722	1.18764762224165
H	13.13261923456627	10.78609392552372	0.46431228968118
H	14.45107570891907	9.79749227500702	1.12009273007428
H	13.15709116583976	10.37568307522404	2.19522073360897
C	12.87195675220929	8.15183417240834	-0.48806148583891
H	12.35016278065062	7.19805411241488	-0.65019353056129
H	13.95060000833901	7.98220350378043	-0.62336228175669
H	12.53587159351791	8.85709522148675	-1.26292565202061
C	8.08944089077256	7.02169518011310	2.66408061207303
H	8.53312111502041	6.02779065449584	2.49557129816004
C	8.22142003323799	7.33241334796298	4.16787422272627
H	9.27243275961587	7.31925806905842	4.48836574548961
H	7.68218453944158	6.58211452103274	4.76502700365565
H	7.80853583413272	8.32388592165742	4.40412631997423
C	6.61317052884748	6.93823803881821	2.26446087507042
H	6.07000141174999	7.86038543353477	2.51848146335438
H	6.12515513184650	6.11648352259356	2.80738288504701
H	6.49280357609927	6.75529845024022	1.18705079863630

TS

Fe	-1.42750891989745	1.24939578737510	-0.63484460303550
Ge	-0.15971403665233	-0.78971936046079	-0.76596368455846
Ge	0.02466456610002	1.60815245969665	0.82987002265839
C	-1.21014211587273	-1.74828030938669	-2.17505256145228
C	-0.97847099808035	-3.05389436868390	-2.65964395566370
C	-2.49096347515992	0.38855192038849	-2.18098405682252
C	-3.33000989291278	0.47741453447979	-1.00223184136895
C	-1.74569049925258	-3.53608191612333	-3.74250003830064
H	-1.55365567280540	-4.55410297290740	-4.11605735706896
C	-2.22410423578103	-0.96126525529555	-2.76576938936373
C	-2.72926669512465	-2.73703836659461	-4.34435826661318
H	-3.31199215286975	-3.12563924361769	-5.19328663319945
C	-2.97538580411782	-1.44659838188086	-3.85072909014232
H	-3.75658650956249	-0.81355833644716	-4.29909813060298
C	-0.80900767620107	1.38630737172796	-3.86934435727894
H	-0.63670408344359	0.30024764399758	-4.01499321950424
C	-1.74060671197673	1.52289767406509	-2.67300708928564
C	1.34039409366455	-4.04694048794144	-2.59010009875348
C	-0.34410074311768	-4.77626362806943	-0.94035814980452
C	0.03697195145844	-3.96060109120360	-2.03823978453294
C	-1.75020392704320	-4.68014766271157	-0.35592909045237
H	-2.16695583840184	-3.70469601660650	-0.68066859377086
C	-4.05143786393901	-0.75084381521413	-0.46784974819758
H	-3.44722551226442	-1.62997712896073	-0.77299699152871

C	-1.87111062404023	2.76348644243910	-1.97504227227344
H	-1.24817258477802	3.62137364585236	-2.26264514974705
C	-3.43284255653809	1.74214108249338	-0.34964409896745
H	-3.99934812486401	1.82915160676167	0.58715565981506
C	1.77460733665883	-3.11012337795189	-3.71146297777204
H	0.86231615012732	-2.59522437781581	-4.07550304500084
C	0.57878195143545	-5.71598069307221	-0.44436745109908
H	0.29308982052431	-6.36965181957092	0.39383573393077
C	2.23942332245048	-4.99157508023474	-2.05964207864005
H	3.25345448334707	-5.07305454539392	-2.48057771776783
C	-2.72399939412869	2.87701572731225	-0.83916002881898
H	-2.71530902946403	3.80406802362074	-0.25146304376699
C	-5.43623713651737	-0.87704749987565	-1.13015837495124
H	-6.07523653642843	-0.00446485990697	-0.87881872045105
H	-5.95714982169998	-1.79433832877261	-0.78615989194140
H	-5.34938853429257	-0.92782270031352	-2.23363600070557
C	1.85746507135740	-5.83430684361277	-1.00720858390722
H	2.56615789672217	-6.57876314232679	-0.61226596566190
C	-4.14573248631298	-0.77806329812115	1.06264069714249
H	-3.14269921164324	-0.65805578914503	1.51799820332352
H	-4.56729404869080	-1.74537153693007	1.40298711190522
H	-4.80651717890647	0.02185360813946	1.45803268066176
C	2.39184995435348	-3.85495725528527	-4.90594354756799
H	3.34234512398654	-4.35922944946924	-4.63242852228626
H	2.62100695702862	-3.14796571848260	-5.72990852701945
H	1.70136324340863	-4.62849692073894	-5.29899062418237
C	-1.50067548074492	1.92243954477294	-5.13624688289350
H	-2.46137687818440	1.40077495722598	-5.32056403726584
H	-0.85851367339990	1.78339499732676	-6.03032141923596
H	-1.72009949468778	3.00667464970690	-5.03863153926178
C	0.56210134529379	2.03870195866119	-3.64312407216607
H	0.49097654365305	3.14326207905600	-3.56583696503052
H	1.24373133930848	1.81220517887159	-4.48792653508709
H	1.02181909926955	1.66268595333111	-2.70744719665124
C	2.71683475307248	-2.02268498233871	-3.16305515276439
H	2.24693398824078	-1.50248250443216	-2.30300412375455
H	2.95699778402443	-1.26992470540725	-3.94252467378935
H	3.67324410909251	-2.46283507696250	-2.80914096808685
C	-2.65283756049712	-5.78484212541769	-0.93659479187562
H	-2.69533660931174	-5.72363281329444	-2.04214066353802
H	-3.68888865936175	-5.69669562989195	-0.54804603874715
H	-2.26989248544780	-6.79186108316984	-0.66770237673244
C	-1.76494066114670	-4.68653546958144	1.17959346278936
H	-1.41782895632646	-5.65300107559684	1.60103918790373
H	-2.79365389056634	-4.51311194861079	1.55631681037774
H	-1.11816752173805	-3.88187285912974	1.58191632248531
C	1.39317603365579	1.87165316183692	2.18862206553866
C	1.31773586507173	3.16860669530822	2.75568529211942
C	2.40293621490160	-0.43432436030199	2.07287027827624
C	3.26834850923070	-0.73431894904241	0.98626776008243
C	2.21412866790156	3.54311507627608	3.76843281037454
H	2.14723023945740	4.55110351693996	4.20558041555443
C	2.36321361803403	0.94799921064461	2.63532699743240
C	3.18838644281037	2.63062534647861	4.20945398115951
H	3.89447259664609	2.92144169077609	5.00206950636594
C	3.26095082087001	1.34664563195547	3.64631090407186
H	4.01650722908414	0.62749614443978	3.99841254210682
C	0.65479737626126	-1.11690531553840	3.82717867212449
H	0.88563254346308	-0.08513319605105	4.16134715799895
C	1.57699461745493	-1.43162957617043	2.65303256535513
C	-1.04204695824435	4.06944949140363	2.78017609680228
C	0.57523615690883	4.93152452806162	1.11812572135911
C	0.26629554246207	4.09274538982816	2.22373028191438
C	1.95222163313105	4.90155988967894	0.46666960929451

H	2.51133282111273	4.05707463889296	0.91810212459353
C	4.12495762499531	0.35835455269665	0.35108696071677
H	4.32917242079952	1.11235890521714	1.14042911720899
C	1.62264499320431	-2.73379452684358	2.11739723206671
H	0.98919510696842	-3.52449581892154	2.54713992655008
C	3.28787174965585	-2.05004985916073	0.49249958845193
H	3.94162617354027	-2.30812994172317	-0.35143898152157
C	-1.40465431163180	3.12504700836762	3.92046176921009
H	-0.55269032296593	2.42791843211271	4.05785921436856
C	-0.42403043296257	5.78870959558443	0.62225859455212
H	-0.19794083387314	6.45034810548490	-0.22765686669834
C	-2.01089847927565	4.94554460932215	2.25469274555934
H	-3.02597517008223	4.94864847600004	2.67990663270469
C	2.47117754244697	-3.04146466996963	1.04806403554330
H	2.48158182812542	-4.05599335098612	0.62931735613698
C	5.48096652429247	-0.15640783432171	-0.15723466616118
H	5.36139416618223	-0.82895171410911	-1.03170973473265
H	6.11711257120830	0.69078143957352	-0.48530470267301
H	6.02727779018595	-0.71533353734434	0.62916007211519
C	-1.70245406068456	5.80823719432779	1.19506249979097
H	-2.47127788470611	6.49048503160132	0.80040547792393
C	3.36108651654885	1.07666195854834	-0.77826528364257
H	2.41388384811785	1.52572228637097	-0.41683055511441
H	3.97372452876734	1.89266097794379	-1.21374769152546
H	3.09727142401733	0.36802296980184	-1.58792638195842
C	-1.57811852353243	3.89887553477443	5.24033410318667
H	-2.41195998587995	4.62863817013891	5.17133121432712
H	-1.80245305556065	3.20583013039711	6.07733412304829
H	-0.65884439435289	4.46288481341312	5.49735297773802
C	0.90934126513010	-2.05222388328126	5.02206040694389
H	1.97188652581342	-2.02275141114557	5.33746269079600
H	0.28295997919908	-1.75794925522746	5.88938078145906
H	0.66019569767970	-3.10532679730545	4.77594170988867
C	-0.82324724270925	-1.13515049794629	3.40008414223907
H	-1.13623945590395	-2.14439271288423	3.06222847345781
H	-1.48353222483065	-0.83881998032452	4.24079352908221
H	-1.00777980540611	-0.43558926106365	2.55792076463110
C	-2.64085728498633	2.27359059979981	3.58189846534239
H	-2.49041865803746	1.72634182096134	2.62846173967305
H	-2.83471262437393	1.52961834050722	4.38094485700606
H	-3.55472405240419	2.89539701484651	3.48035299992169
C	2.74018335194804	6.18751188399477	0.77454129328839
H	2.84835192420781	6.33750490212307	1.86774743428600
H	3.75631229575052	6.14299689403908	0.33104713086869
H	2.22885121654283	7.08209293161152	0.36119697474145
C	1.85345621520407	4.62528707632202	-1.04326041760661
H	1.37088056044216	5.46425021706528	-1.58715053036670
H	2.86154537430998	4.48094657075399	-1.48200177940998
H	1.26089643062385	3.70749999150947	-1.23277172401051
H	-1.08780907312120	-1.72108929747935	0.41814457160143
H	0.08371388077635	-1.90215809493321	0.36555782418726

TS1

Fe	-1.47632514539627	1.17175095498578	-0.56003389190837
Ge	-0.20669177721749	-1.08667275452930	-0.48038441334552
Ge	0.04016470816797	1.45996352377692	0.85344510030031
C	-1.27819139326756	-1.77325919681165	-2.09037754426169
C	-1.00764581817384	-3.05330688885306	-2.62668738590669
C	-2.53447936119325	0.37361855453654	-2.12658032747554
C	-3.43272582586346	0.57661695778193	-1.00842381215808
C	-1.78971349863010	-3.54754263056161	-3.69512398945309
H	-1.56633869263442	-4.54685956894232	-4.10037823022807
C	-2.31937639934488	-1.00395312355307	-2.65889197986763

C	-2.83086108093527	-2.78151104577215	-4.23599238465449
H	-3.43093043736248	-3.17690102984132	-5.06986889637173
C	-3.09593703371634	-1.50375772196062	-3.71955847275679
H	-3.89656724522634	-0.88210734974675	-4.14907582238918
C	-0.71730184913592	1.18573614634354	-3.77237620343723
H	-0.52259796556762	0.09474329304935	-3.80342421341250
C	-1.66928884444410	1.42707356265748	-2.60845719237769
C	1.37775423327020	-3.86002106329389	-2.71270523359228
C	-0.17592731089514	-4.86343377571898	-1.08003349403713
C	0.09547084408336	-3.92394054608171	-2.10605559929054
C	-1.54895919596206	-4.93238385145818	-0.42097946927723
H	-2.15250729763593	-4.09887491776718	-0.83466603083212
C	-4.31570399289683	-0.55710263805231	-0.50431424397524
H	-3.77352021379262	-1.50473729558983	-0.71357300051843
C	-1.73639057031858	2.69696494075840	-1.95540983779105
H	-1.03075275381516	3.49104029444660	-2.23359463536781
C	-3.43942036669135	1.85797949457893	-0.37538949349633
H	-4.04041441848656	2.01856584155256	0.52953391479979
C	1.67342774768955	-2.80642341238141	-3.77538126443658
H	0.69739798989054	-2.48151605972418	-4.19079055532150
C	0.84143736760560	-5.75638372861715	-0.69270571419809
H	0.64077248641436	-6.50046161646961	0.09379187497750
C	2.37535087175743	-4.75499806566428	-2.28411285756961
H	3.37475957418863	-4.71846926956538	-2.74328615700596
C	-2.60883917016467	2.91087969761170	-0.85131655387281
H	-2.54331122426062	3.84942303595811	-0.28670399865803
C	-5.63584207591944	-0.59261259100844	-1.29744860744698
H	-6.20522223946415	0.34828261756755	-1.14697209272136
H	-6.27479162412770	-1.43786986536947	-0.96788586190768
H	-5.45311541824278	-0.70775601298138	-2.38334294846213
C	2.10677214570832	-5.70885844961165	-1.29233159717911
H	2.89235449805697	-6.41412139092798	-0.97984313987779
C	-4.58331736549187	-0.49403987897240	1.00677516204861
H	-3.64232594727136	-0.36910572191190	1.57918061120980
H	-5.07753861858033	-1.42450328612493	1.35118525116162
H	-5.25388757486524	0.34947417975373	1.27223214179972
C	2.51670139068507	-3.33369974572642	-4.94596130562970
H	3.54404999160830	-3.60863086908960	-4.62804356449709
H	2.61761966374824	-2.55678660329628	-5.73148104260494
H	2.05399058601388	-4.23003077546242	-5.40624462558632
C	-1.40592817236050	1.57479634113500	-5.09415895375718
H	-2.35154406971506	1.01340387359732	-5.23203931482809
H	-0.75047103187792	1.36065079144224	-5.96355467062209
H	-1.65101327840183	2.65770733254935	-5.10730089467614
C	0.63713926501469	1.88495104422375	-3.60760679694224
H	0.54638228551640	2.98968596356582	-3.65498097822962
H	1.33038473154865	1.58182025124200	-4.41825947035719
H	1.09681764925645	1.62120509388594	-2.63488374291743
C	2.32355306825543	-1.57286688969159	-3.12618828474382
H	1.71858184648835	-1.20691780039799	-2.26980884158411
H	2.43488127149708	-0.73957232057477	-3.84931630810253
H	3.32722583371427	-1.82019501114568	-2.72273921025189
C	-2.27143131319495	-6.24410231846441	-0.77649571127315
H	-2.36704293546680	-6.36092438482445	-1.87493396979271
H	-3.29061683792782	-6.26624919780176	-0.33774308444216
H	-1.71900117237138	-7.12704200572935	-0.39181279193863
C	-1.45858297594116	-4.71550692798615	1.09862162839592
H	-0.86685505796985	-5.51360994748136	1.59433552444176
H	-2.46865310722387	-4.71313496550896	1.55808200284588
H	-0.97496684272949	-3.74242075306626	1.31976646669093
C	1.41550749192667	1.79449654022811	2.19444531735866
C	1.34605938589993	3.10876650753498	2.72116245978224
C	2.41424000229303	-0.50872505754082	2.13587949722924
C	3.25350852321065	-0.82602218679384	1.03449249241315

C	2.24826299074892	3.50938336620060	3.72011254959506
H	2.18658173939333	4.53161074156352	4.12385467774598
C	2.38499315529554	0.88392771088295	2.67032685681922
C	3.21848157306922	2.60834394315780	4.19077561210405
H	3.92615183708314	2.92211699383168	4.97320829798984
C	3.28494854464253	1.30703632651232	3.66856312621125
H	4.03718915824989	0.59531010005933	4.04227642147888
C	0.68622198656256	-1.15400504480157	3.92329747840332
H	0.94810414067322	-0.13004521858995	4.25905721634878
C	1.58334581772811	-1.48949635859664	2.73554352564160
C	-0.99471863582563	4.05642296789442	2.76001393104125
C	0.61657723036597	4.86001187261775	1.06586634511271
C	0.30319116903031	4.03792286065434	2.18127618954127
C	1.97663401457967	4.77933503429448	0.38360957468499
H	2.52957270429697	3.93893259125224	0.85006979123172
C	4.15138610982724	0.24253020286980	0.41416799419302
H	4.39501388422458	0.96544262442612	1.22103662811709
C	1.59773660622959	-2.79304224662942	2.20714389554965
H	0.96384767655298	-3.57032880654566	2.65821609193890
C	3.21925617231626	-2.13730628283722	0.52794362873614
H	3.84236965252058	-2.40990545754765	-0.33530643547698
C	-1.36598589341117	3.12801595773231	3.91074785173517
H	-0.53184369744717	2.40811778598136	4.04060318730967
C	-0.36418576164145	5.74649921947183	0.58358947959298
H	-0.13296986984631	6.39715876550297	-0.27356118463975
C	-1.94530646136334	4.95996957024603	2.24873328327274
H	-2.95212055066285	4.99548050532466	2.69182295885630
C	2.39900291163420	-3.11298885157277	1.10769850261474
H	2.37414422505928	-4.12488686273538	0.68345515647484
C	5.47826621217383	-0.32028400543588	-0.11881352898363
H	5.32057758640061	-0.96662533468294	-1.00719471629304
H	6.14957344450249	0.50439795857216	-0.43403488621690
H	6.00529726998154	-0.92219446255356	0.64869938823997
C	-1.62981046047429	5.81016258676536	1.18068628370687
H	-2.38395036276460	6.51512380553175	0.79784037620799
C	3.41666416793704	1.02884514935141	-0.68743939679860
H	2.48323443987987	1.49285230276482	-0.31032982748111
H	4.05551238548609	1.84118016379510	-1.09098799056628
H	3.13597600621876	0.36209054981869	-1.52484855308522
C	-1.50004785067106	3.91332557991898	5.22843823660664
H	-2.31689795858517	4.66272484004130	5.16705303531921
H	-1.72843204803954	3.23119573066350	6.07334132469906
H	-0.56336536160356	4.45614335878948	5.46761159882962
C	0.92850741411461	-2.10080210007001	5.11166896648680
H	1.99580759802388	-2.10439130205592	5.41192813863331
H	0.32370764311459	-1.79057832401036	5.98873901847609
H	0.64428612205999	-3.14515821516810	4.86636989848313
C	-0.79747001067686	-1.13233909779420	3.51458730565413
H	-1.12888351042952	-2.12499769599609	3.14670818451385
H	-1.44262927446172	-0.85149698422825	4.37237390157469
H	-0.97694706964967	-0.39570980277387	2.70266691370255
C	-2.62949064947468	2.30821110175980	3.59462031964915
H	-2.50515649195907	1.75411174898876	2.64126951769568
H	-2.83094914979512	1.57339211889945	4.40046819248380
H	-3.52807563178446	2.95343665497101	3.50310520794030
C	2.80024218350635	6.05715536776954	0.62134525853652
H	2.94335001404112	6.24362742957660	1.70489056262384
H	3.80199904250384	5.97420662158461	0.15129805912029
H	2.29727390371745	6.94806438844431	0.19017095057163
C	1.82821176889343	4.44881516157028	-1.11127085618303
H	1.34264395559152	5.27534996855227	-1.67147838701557
H	2.81857135964212	4.26781650318217	-1.57591364242426
H	1.21308751782560	3.53654727164420	-1.24629768997694
H	-1.82889662216674	-0.71868263379111	0.39953964910341

H	-1.62710892430255	-1.58675474133294	0.62414781810353

TS2

Fe	-1.69799706764977	1.10273523787264	-0.40486067192111
Ge	-0.54931034443151	-1.04017694289841	-0.33725277496925
Ge	0.02359167211207	1.29835886526269	0.84978107860666
C	-1.19302334324592	-1.71836093538779	-2.09524916382006
C	-0.89210653261440	-2.98859276768018	-2.63952656722427
C	-2.53064431600377	0.43100125243867	-2.14895015497076
C	-3.59617026617623	0.47686309768253	-1.16597016263176
C	-1.51651777630190	-3.39933398174318	-3.83800340318957
H	-1.27755681675621	-4.39491088016409	-4.24357008971684
C	-2.14436022038837	-0.88894600240174	-2.75239886851711
C	-2.42871740148622	-2.56683152150708	-4.49842519012666
H	-2.90199498033479	-2.89637865851909	-5.43564039757841
C	-2.74745778804204	-1.31585225190653	-3.94852293571589
H	-3.47594638723864	-0.65917625375888	-4.44791352222449
C	-0.62998422926948	1.51367840269514	-3.49606255957203
H	-0.35332048531994	0.44464358846455	-3.60296583304877
C	-1.69643223058841	1.58094101231303	-2.41281725188159
C	1.33417305731623	-4.16607009503778	-2.48537367805298
C	-0.44303190566372	-4.69881806929205	-0.85032131991638
C	0.03048139612831	-3.95050155797500	-1.96067442567109
C	-1.86109236365479	-4.50517878669500	-0.32000488392980
H	-2.17621480845745	-3.47469994116080	-0.58316226438137
C	-4.42899183287139	-0.76792197084092	-0.88799026531360
H	-3.77699091065790	-1.63987755034956	-1.10801121975629
C	-1.89207824919857	2.74852050181490	-1.60593212578848
H	-1.20111336495850	3.59699603346888	-1.69573055205543
C	-3.73736769924900	1.65217022205342	-0.37110469408799
H	-4.47061970048780	1.67559095481273	0.44684348937570
C	1.84209316446657	-3.31102911555717	-3.64460650362348
H	0.98812855514404	-3.16996767225041	-4.33959507656833
C	0.39479738423140	-5.68599556251403	-0.29718474305454
H	0.03967084608908	-6.28579773759811	0.55365654743641
C	2.14160699185983	-5.15745259304533	-1.89769998408976
H	3.15019881944983	-5.34637823164020	-2.29165986149482
C	-2.88326452858524	2.77430180845445	-0.57741519387738
H	-2.92142925946471	3.63019478104876	0.10916249159488
C	-5.62856950056915	-0.82858654387318	-1.85267745429921
H	-6.31124301922872	0.03069697716489	-1.68589246671430
H	-6.20813837773925	-1.76250206591756	-1.70089137407826
H	-5.30013236526480	-0.80031671704722	-2.90977033292579
C	1.67127976049597	-5.92287280383080	-0.82163626979262
H	2.31046839816903	-6.70489926758682	-0.38308711873213
C	-4.88240676694271	-0.89645368237386	0.57263720927685
H	-4.02432851217156	-0.79927256090916	1.26755862862013
H	-5.35152764055168	-1.88670652379303	0.74106880971839
H	-5.63622630303189	-0.12795828832590	0.84315395543287
C	2.98455466538692	-3.95323102085538	-4.44242640572047
H	3.91679279083661	-4.02407314215148	-3.84365276479862
H	3.22097258867748	-3.33958777228222	-5.33530436221229
H	2.72375273524997	-4.97410294237722	-4.78810793483757
C	-1.23648245417596	1.97704726711976	-4.83454261081110
H	-2.12001617944305	1.36752902754442	-5.11010822757947
H	-0.49472403898745	1.89622075846723	-5.65562755382082
H	-1.56367779463698	3.03615523132303	-4.77090021151412
C	0.64755788973745	2.28381012111891	-3.15742833478731
H	0.48332252514961	3.38027697639139	-3.14073993064750
H	1.43569064721279	2.08118694829629	-3.91011726857522
H	1.02854417599167	1.97935701969850	-2.16422686139028
C	2.24628666895236	-1.91234528468542	-3.14237304472030
H	1.41095474316449	-1.41343478354333	-2.61238742214999

H	2.56063136316384	-1.25968650926465	-3.98266845306193
H	3.09013665793708	-1.98707397352436	-2.42866868850232
C	-2.83191572762293	-5.47222556137377	-1.02538786383874
H	-2.81166183911195	-5.32768470239568	-2.12388689501122
H	-3.87359414442377	-5.31021607835413	-0.67781676076069
H	-2.56015983788620	-6.52820260105212	-0.81638058048581
C	-1.95867275516311	-4.62075701650614	1.20769738053750
H	-1.76589675038234	-5.65382559457563	1.56497835339523
H	-2.97572408176978	-4.34025003312633	1.54950575434052
H	-1.23582490638020	-3.94192891139890	1.70266153915279
C	1.53790474079617	1.84694115084432	1.95555551763773
C	1.44828280844376	3.14341429710043	2.52804177499340
C	2.52791867334171	-0.47277491818602	1.93392390975309
C	3.31337188160374	-0.90921253840837	0.83461469794001
C	2.41203534814750	3.55191990777596	3.46855106460255
H	2.33558241453086	4.55861962376531	3.90804969602271
C	2.55541908196039	0.95416472811725	2.37594721138429
C	3.45585427291114	2.68974181312734	3.83961255485169
H	4.20908755660923	3.01982820242960	4.57096698437723
C	3.51910724257981	1.39397744967755	3.30182432012286
H	4.30705449876045	0.69520195971628	3.62276431176931
C	0.86637392007151	-0.92489080475081	3.84610279477173
H	0.90610673759419	0.18217737311126	3.88048113848364
C	1.70856525775934	-1.37891659208598	2.65803914973914
C	-0.97437155126001	3.82545740848431	2.72299374325676
C	0.52858213518288	5.10650786470628	1.23570294590176
C	0.31247883765754	4.04526579136628	2.15536773107734
C	1.88946166094016	5.30644552525127	0.57901481395318
H	2.64131109992589	4.79863226300893	1.21586402956530
C	4.17204737412090	0.08988385164153	0.06286799307045
H	4.56746050579136	0.81510230908886	0.80620986092829
C	1.70152251520427	-2.73284972100676	2.27322435414559
H	1.08066614641636	-3.45399146888695	2.82630341743416
C	3.25181458003148	-2.26602547528186	0.46826418834121
H	3.84090125883475	-2.63503071829439	-0.38242848836808
C	-1.20941328290309	2.73330318081244	3.76361612498882
H	-0.43505610243361	1.95339356193047	3.61075726583609
C	-0.55592486876211	5.93278136748845	0.88619830214680
H	-0.40551097682693	6.75404642109048	0.16966520751988
C	-2.02664943819927	4.68592530183278	2.35710059807367
H	-3.02467235154444	4.53428981135493	2.79298370911790
C	2.46097805896584	-3.17290915730004	1.18508694312912
H	2.42356004184382	-4.22532289035577	0.87572306044575
C	5.37743080740598	-0.55096976383324	-0.64060928221728
H	5.06329476004852	-1.20381456003203	-1.48114541634601
H	6.03437969497718	0.23307975499840	-1.06859108561795
H	5.98167252911995	-1.16479465026762	0.05745860767658
C	-1.82307330298744	5.73034221679356	1.44653475724338
H	-2.65956045753210	6.39054956809428	1.16953093436179
C	3.31817820450472	0.89054723846648	-0.93859048316948
H	2.51981189743081	1.45652209899473	-0.42093907617876
H	3.93594953164442	1.62402563913729	-1.49628162676317
H	2.83028889115614	0.21912723283535	-1.66978102579017
C	-0.99901924015786	3.29963735584765	5.18126158476566
H	-1.73420395327757	4.10260336900334	5.39820010660030
H	-1.12138273840308	2.50467569256167	5.94586275801152
H	0.01645714226418	3.72975187727504	5.29253630364599
C	1.46494556848301	-1.43869250434776	5.16795278421014
H	2.51132094456859	-1.09325342456285	5.29038547209029
H	0.87788502120003	-1.07401378092343	6.03643347306839
H	1.47011672552972	-2.54830982471218	5.20295290885656
C	-0.61290330636039	-1.31688132017453	3.69334087314148
H	-0.75069286875087	-2.41745068749058	3.66912933536350
H	-1.20783681825818	-0.92583347660552	4.54395463429236

H	-1.04114623113135	-0.90030386286537	2.75816048876110
C	-2.57276163276272	2.04272152236865	3.61454018158303
H	-2.69831508174992	1.65815469869269	2.58106386124337
H	-2.64722437980918	1.18421548199574	4.31182456603747
H	-3.41685948030641	2.72495235075024	3.84615848091680
C	2.30132682458168	6.78232801360614	0.46621439318984
H	2.24994536599293	7.29379135405134	1.44867290571613
H	3.34084645755814	6.86559495102868	0.08835575357838
H	1.65386092427039	7.34200424006379	-0.24050166346785
C	1.90714358091346	4.60340406625626	-0.78876440116017
H	1.16937214955503	5.06567707400256	-1.47744299513143
H	2.90745208413342	4.66281061862500	-1.26506293594533
H	1.63914516837794	3.53414652074531	-0.68514054866728
H	-1.99691278713000	-0.72467242267806	0.31805022945797
H	-0.11181176553713	-2.16860216132557	0.60788251267990

INT

Fe	-1.64039678031870	1.17936005270082	-0.47412887341340
Ge	-0.40634608933392	-1.02561501420819	-0.27819312167410
Ge	0.12984948962916	1.33001281122302	0.72047174411009
C	-1.24038884365333	-1.72207003133265	-1.99335158500907
C	-0.95541455599831	-3.00938712242089	-2.50448298044222
C	-2.53100888576598	0.43817543542013	-2.13744872034618
C	-3.54314401945063	0.63637221974723	-1.11969724626380
C	-1.66121880564303	-3.49149100331515	-3.63113207390641
H	-1.42324228997660	-4.49754512979782	-4.01049838510511
C	-2.23045306966780	-0.93787467358318	-2.64389610463336
C	-2.63491998555671	-2.71050670213506	-4.26436626370234
H	-3.17269283682232	-3.09814204973359	-5.14281188330774
C	-2.91517182224603	-1.42874228062386	-3.77018810898882
H	-3.67303459396935	-0.79614154944694	-4.25730778060100
C	-0.61483513742731	1.29914257374390	-3.62063713947058
H	-0.41656546074576	0.20928825673822	-3.66480763400121
C	-1.64203388480505	1.51040459149460	-2.51655177468395
C	1.36028725618962	-3.99549065943787	-2.52746819508934
C	-0.27746009729882	-4.79483571364845	-0.85823019388328
C	0.07804955367748	-3.92024501822498	-1.91670641447205
C	-1.67381041843439	-4.75149720595466	-0.24581510167155
H	-2.10779304251871	-3.76225440710049	-0.49608506983336
C	-4.42058471897648	-0.53385104128535	-0.69159097100859
H	-3.85541149062554	-1.45658548429891	-0.93967307565683
C	-1.77461310053872	2.76474779828108	-1.83536329676406
H	-1.03830484988540	3.55873249927935	-2.01998426058613
C	-3.61482850927582	1.89652775899511	-0.44389802661578
H	-4.31733892676929	2.03239596494127	0.38945140972886
C	1.72861047084109	-3.02978110358914	-3.65106954187379
H	0.78977429266597	-2.80870147013176	-4.19965594007819
C	0.65558511322420	-5.76365269963464	-0.43971033258874
H	0.38836133437946	-6.45969660210350	0.36952843784310
C	2.26865640230532	-4.96788727710706	-2.07062430765468
H	3.26328108654598	-5.04457989060167	-2.53300484825261
C	-2.72510975497096	2.95009783738798	-0.79288524335288
H	-2.71380657640801	3.88019164195860	-0.21026905392257
C	-5.71462746120505	-0.53436389022386	-1.52702165460717
H	-6.30977339529896	0.38195878006976	-1.33041714909774
H	-6.34500394575974	-1.41275817839029	-1.27882130072837
H	-5.49400601383956	-0.56756227462970	-2.61216919986462
C	1.91523698482767	-5.85668578497339	-1.04458643797615
H	2.63247241097082	-6.62176984711890	-0.70943456224035
C	-4.71870206764057	-0.57331181605652	0.81341470495723
H	-3.78095141510703	-0.54103745505641	1.40280398083752
H	-5.25690548796559	-1.50747671687813	1.07167276404576
H	-5.36017647670061	0.27248272360859	1.13760539050616

C	2.72783580213634	-3.60667111389654	-4.66381552320747
H	3.73375924570505	-3.75161328763845	-4.21699191685326
H	2.85246264086231	-2.91045264471743	-5.51810188738648
H	2.38909471696369	-4.58407396127807	-5.06322071108819
C	-1.23144779925480	1.71645210878056	-4.96932446966000
H	-2.16717936450171	1.15811056451756	-5.17193496650047
H	-0.52797202726161	1.52082071745349	-5.80463573014040
H	-1.47671131016545	2.79939473734025	-4.97308946178151
C	0.72696604016380	1.99164686825235	-3.37034181438268
H	0.64132885736887	3.09706312948772	-3.39095267902601
H	1.45937265606886	1.70560585983720	-4.15185525825328
H	1.13828123921720	1.69490455337636	-2.38678334599630
C	2.23749633429022	-1.69495563447047	-3.07714813171596
H	1.52591658520894	-1.26668372441119	-2.34239654363800
H	2.39813396582587	-0.94920524250685	-3.88285257462506
H	3.20122983011704	-1.84300099662165	-2.54959741961318
C	-2.57255023812004	-5.82718755223445	-0.88537477342703
H	-2.63678812136492	-5.69205362592554	-1.98340393630846
H	-3.60215760208497	-5.77949870238488	-0.47340265008024
H	-2.17255904750893	-6.84478337476225	-0.69213205622072
C	-1.66033498035350	-4.86299032639122	1.28596908475277
H	-1.30279992298008	-5.85481190782320	1.63330303250313
H	-2.68293855238737	-4.72175135734451	1.69150784584682
H	-1.00930813669919	-4.08307518667243	1.72848047553588
C	1.50893020403981	1.79567573398863	2.02531240818500
C	1.36310824126521	3.09565723476940	2.57557741100203
C	2.54271875063877	-0.48377910213728	2.02407439625214
C	3.34390762880221	-0.82450592240386	0.90449315598239
C	2.24712324068331	3.52228061682729	3.58325536947348
H	2.13013545391905	4.53369233969991	4.00162093567811
C	2.50700692618692	0.92179203331047	2.51734077870447
C	3.26650627382865	2.67225683713597	4.04059272561737
H	3.95876731285008	3.01624815520435	4.82411387897860
C	3.38931492318914	1.37679401815619	3.51531945044951
H	4.16362054585586	0.69180416995851	3.89338799297061
C	0.87896772464970	-1.08439431997386	3.89108272135026
H	1.16697702776740	-0.06077343271131	4.20597376913887
C	1.71323546510624	-1.44041434308318	2.66362866121457
C	-1.03510039698358	3.87247094282270	2.67359353928664
C	0.53533279022339	4.97990291143448	1.12018643067610
C	0.26390226254754	4.00012416654979	2.10906467651898
C	1.89450499792592	5.04769670875399	0.43555817339456
H	2.57227169728704	4.35519409010330	0.97487029405547
C	4.23253772297010	0.22724977330916	0.24575414298065
H	4.50194368112573	0.96027542439688	1.03522501326664
C	1.66450189262135	-2.74006326717821	2.13104462872604
H	1.01773537169782	-3.49880631263931	2.59670635159454
C	3.25749050453084	-2.13379188462113	0.40364234744845
H	3.85316942413310	-2.43068652319735	-0.47069034360888
C	-1.34092617206173	2.80336110750609	3.71691435251455
H	-0.55237544584393	2.02778805965175	3.63308828259985
C	-0.49667380273263	5.85597549142478	0.73292734875923
H	-0.30134728403562	6.62198862523483	-0.03319990336413
C	-2.03365749719552	4.77669175474876	2.26978240684775
H	-3.04129539799748	4.69969136763424	2.70423413386077
C	2.41286604036937	-3.07893561331828	1.00020859401256
H	2.34245731534927	-4.08839410714539	0.57755881701665
C	5.54097847943963	-0.35168999418083	-0.31478707808523
H	5.35667047461376	-1.01202295941801	-1.18759376594584
H	6.20838326629688	0.46344143582412	-0.66170069818136
H	6.08486952531248	-0.94376834765711	0.44852281438021
C	-1.76690187419143	5.76857838266119	1.31593427053383
H	-2.56067031258951	6.46948809233609	1.01414904612652
C	3.46914722490802	1.00105380523954	-0.84532329384615

H	2.55293662494029	1.47566605461044	-0.43895780390895
H	4.09576707546107	1.80851840734429	-1.27692966434233
H	3.15581482720130	0.32765038287168	-1.66550762139661
C	-1.24523288269350	3.39122120219876	5.13745242650285
H	-1.99210407034969	4.19962872964446	5.28308553619293
H	-1.43286401409946	2.60886210983416	5.90180764576321
H	-0.24078202289446	3.82100989099054	5.32550586224873
C	1.18277792569465	-2.02456520557014	5.07130544755011
H	2.26535390197995	-2.03242619197019	5.31063157590251
H	0.62979853721430	-1.70551358107911	5.97894084668811
H	0.88036532656223	-3.06883432048925	4.84845832216079
C	-0.62695246576552	-1.05219483820146	3.57541803959438
H	-0.99300393551381	-2.03886511324597	3.22628995428265
H	-1.20906702144851	-0.76931794671123	4.47661184167483
H	-0.86289972873190	-0.31692245535632	2.77723713530634
C	-2.68685383406592	2.10538759881182	3.46783167201139
H	-2.73026170722612	1.71259561629537	2.43097140552712
H	-2.81401960502435	1.25395261039280	4.16605967888853
H	-3.54770170478234	2.78833755484785	3.62410034401186
C	2.52151745620444	6.44989451742736	0.49674485486031
H	2.62002724163025	6.80133932552002	1.54368170692607
H	3.53180769090659	6.44575164397550	0.03842040587122
H	1.91231761616494	7.19738034796640	-0.05309103948523
C	1.77576459122165	4.52903101423312	-1.00778574198106
H	1.12386010441469	5.18753434460697	-1.61980592520977
H	2.76756434008589	4.47567279152873	-1.50152693434200
H	1.33028967428121	3.51464231286793	-1.01995107488862
H	-1.89962380080520	-0.13897274565915	0.34013686289494
H	-1.11034280692752	-1.99622940060800	0.74923359495906

References

1 L. Pu, A. D. Phillips, A. F. Richards, M. Stender, R. S. Simons, M. M. Olmstead, P. P. Power, Germanium and Tin Analogues of Alkynes and Their Reduction Products. *J. Am. Chem. Soc.* 2003**, *125***, 11626-11636.

2 K. Jonas, L. Schieferstein, C. Krüger, Y. H. Tsay, Tetrakis(Ethylene)Irondilithium and Bis(η^4-1,5-Cyclooctadiene)Irondilithium. *Angew. Chem., Int. Ed.* 1979, ***18***, 550-551.

3 G. M. Sheldrick, Shelxt - Integrated Space-Group and Crystal-Structure Determination. *Acta Cryst. A* 2015, ***71***, 3-8.

4 O. V. Dolomanov, L. J. Bourhis, R. J. Gildea, J. A. K. Howard, H. Puschmann, Olex2: A Complete Structure Solution, Refinement and Analysis Program. *J. Appl. Crystallogr.* 2009, ***42***, 339-341.

5 G. M. Sheldrick, Crystal Structure Refinement with Shelxl. *Acta Cryst. C* 2015, ***71***, 3-8.

6 (a) *Sadabs*, BRUKER AXS GmbH: Karlsruhe, Germany, 2016. (b) *Crysalispro, Scale3 Abspack*, Rigaku Oxford Diffraction: 2019.

7 R. C. Clark, J. S. Reid, The Analytical Calculation of Absorption in Multifaceted Crystals. *Acta Cryst. A* 1995, ***51***, 887-897.

8 *Crysalispro Software System*, Rigaku Oxford Diffraction: 2023.

9 (a) F. Neese, The Orca Program System. *WIREs Computat. Mol. Sci.* 2012, ***2***, 73-78. (b) F. Neese, Software Update: The Orca Program System—Version 5.0. *WIREs Computat. Mol. Sci.* 2022, 12:e1606. (c) F. Neese, Software Update: The ORCA Program System—Version 6.0. WIREs Computat. *Mol. Sci.* 2025, 15:e70019.

10 (a) A. D. Becke, A New Mixing of Hartree–Fock and Local Density-Functional Theories. *J. Chem. Phys.* 1993, ***98***, 1372-1377. (b) C. Lee, W. Yang, R. G. Parr, Development of the Colle-Salvetti Correlation-Energy Formula into a Functional of the Electron Density. *Phys. Rev. B* 1988, ***37***, 785-789. (c) M. Orio, D. A. Pantazis, F. Neese, Density Functional Theory. *Photosynth. Res.* 2009, ***102***, 443-453.

11 (a) A. D. Becke, Density-Functional Exchange-Energy Approximation with Correct Asymptotic Behavior. *Phys. Rev. A* 1988, ***38***, 3098-3100. (b) A. D. Becke, Density-Functional Thermochemistry. III. The Role of Exact Exchange. *J. Chem. Phys.* 1993, ***98***, 5648-5652.

12 (a) A. Schäfer, H. Horn, R. Ahlrichs, Fully Optimized Contracted Gaussian Basis Sets for Atoms Li to Kr. *J. Chem. Phys.* 1992, ***97***, 2571-2577. (b) F. Weigend and R. Ahlrichs, Balanced Basis Sets of Split Valence, Triple Zeta Valence and Quadruple Zeta Valence Quality for H to Rn: Design and Assessment of Accuracy. *Phys. Chem. Chem. Phys.* 2005, ***7***, 3297-3305.

13 F. Weigend, Accurate Coulomb-Fitting Basis Sets for H to Rn. *Phys. Chem. Chem. Phys.* 2006, ***8***, 1057-1065.

14 (a) B. Metz, H. Stoll, M. Dolg, Small-Core Multiconfiguration-Dirac–Hartree–Fock-Adjusted Pseudopotentials for Post-*d* Main Group Elements: Application to PbH and PbO. *J. Chem. Phys.* 2000, ***113***, 2563-2569. (b) K. A. Peterson, D. Figgen, E. Goll, H. Stoll, M. Dolg, Systematically Convergent Basis Sets with Relativistic Pseudopotentials. II. Small-Core Pseudopotentials and Correlation Consistent Basis Sets for the Post-*d* Group 16–18 Elements. *J. Chem. Phys.* 2003, ***119***, 11113-11123.

15 K. Eichkorn, O. Treutler, H. Öhm, M. Häser, R. Ahlrichs, Auxiliary Basis Sets to Approximate Coulomb Potentials. *Chem. Phys. Lett.* 1995, ***242***, 652-660.

16 (a) S. Grimme, S. Ehrlich, L. Goerigk, Effect of the Damping Function in Dispersion Corrected Density Functional Theory. *J. Comput. Chem.* 2011, ***32***, 1456-1465. (b) S. Grimme, J. Antony, S. Ehrlich, H. Krieg, A Consistent and Accurate Ab Initio Parametrization of Density Functional Dispersion Correction (Dft-D) for the 94 Elements H-Pu. *J. Chem. Phys.* 2010, ***132***, 154104.

17 G. Knizia, Intrinsic Atomic Orbitals: An Unbiased Bridge between Quantum Theory and Chemical Concepts. *J. Chem. Theory Comput.* 2013, ***9***, 4834-4843.

18 Chemcraft - Graphical Software for Visualization of Quantum Chemistry Computations. https://www.chemcraftprog.com.

19 Y. S. Lin, G. D. Li, S. P. Mao, J. D. Chai, Long-Range Corrected Hybrid Density Functionals with Improved Dispersion Corrections. *J. Chem. Theory Comput.* 2013, ***9***, 263-272.

20 F. Neese, F. Wennmohs, A. Hansen, U. Becker, Efficient, Approximate and Parallel Hartree–Fock and Hybrid DFT Calculations. A 'Chain-of-Spheres' Algorithm for the Hartree–Fock Exchange. *Chem. Phys.* 2009, ***356***, 98-109.

21 J. D. Rolfes, F. Neese, D. A. Pantazis, All-Electron Scalar Relativistic Basis Sets for the Elements Rb-Xe. *J. Comput. Chem.* 2020, ***41***, 1842-1849.

22 K. Ishida, K. Morokuma and A. Komornicki, *J. Chem. Phys.*, 1977, **66**, 2153-2156.

23 (a) A. Klamt, G. Schüürmann, Cosmo: A New Approach to Dielectric Screening in Solvents with Explicit Expressions for the Screening Energy and Its Gradient. *J. Chem. Soc., Perkin Trans. 2* 1993, 799-805. (b) V. Barone, M. Cossi, Quantum Calculation of Molecular Energies and Energy Gradients in Solution by a Conductor Solvent Model. *J. Phys. Chem. A* 1998, ***102***, 1995-2001.

24 E. D. Glendening, C. R. Landis, F. Weinhold, Nbo 6.0: Natural Bond Orbital Analysis Program. *J. Comput. Chem.* 2013, ***34***, 1429-1437.

25 (a) T. Lu, F. Chen, Multiwfn: A Multifunctional Wavefunction Analyzer. *J. Comput. Chem.* 2012, ***33***, 580-592. (b) T. Lu, A Comprehensive Electron Wavefunction Analysis Toolbox for Chemists, Multiwfn. *J. Chem. Phys.* 2024, ***161***, 082503.

26 S. Grimme, A. Hansen, S. Ehlert, J. M. Mewes, r2SCAN-3c: A "Swiss Army Knife" Composite Electronic-Structure Method. *J. Chem. Phys.* 2021, ***154***, 064103.
